# Supplementary material for: A unified framework for analysis of individual-based models in ecology and beyond
Source: Nat Commun. 2019 Oct 17;10:4716. doi: 10.1038/s41467-019-12172-y (PMC6797757; doi:10.1038/s41467-019-12172-y)
Supplement: Supplementary file 1 — Supplementary Information [file 41467_2019_12172_MOESM1_ESM.pdf]

Supplementary Notes for "A unified framework for analysis of individual-based models in ecology and beyond" by Cornell et al.

In this Supplementary Notes, we provide:

1. The detailed mathematical derivation of exact expressions for the moment equations to all orders and expressions for equations in Fig. 1E for few most used particular processes and for a general model (Supplementary Note 1),

2. The tutorial for the toolboxes for mathematical analysis and simulations (Supplementary Note 2),

3. Derivation of main results in the case study 1 "Optimal landscape connectivity" and demonstration of application of toolboxes presented in this paper (Supplementary Note 3),

4. Derivation of main results in the case study 2 "Genetic similarity" and demonstration of application of toolboxes presented in this paper (Supplementary Note 4),

5. Derivation of main results in the case study 3 "Optimal foraging" and demonstration of application of toolboxes presented in this paper (Supplementary Note 5).

CONTENTS

Supplementary Note 1 2
Supplementary Note 2 98
Supplementary Note 3 131
Supplementary Note 4 148
Supplementary Note 5 164

Supplementary Note 1

The mathematical derivations of main results

This Supplementary Note 1 contains the derivations of the main mathematical results that underpin our framework for studying spatial stochastic systems. First, for a broad class of spatial stochastic point processes we derive the full hierarchy of equations for evolution of the correlation functions and cumulants. Second, we present the perturbation expansion of these equations in the approximation of long-ranged interactions, and obtain the equations for the mean field solution and its corrections. Our results are based on the approach established in the publication *Ovaskainen O., Cornell S. J., Proc. Natl Acad. Sci. USA* ***103****, 12781 – 12786 (2006)* (Ref. [9] in the main text), and on the mathematical formalism developed in the publication *Ovaskainen O., Finkelshtein D., Kutoviy O., Cornell S., Bolker B., Kondratiev Y. Theor. Ecol.* ***7****, 101 – 113 (2014)* (Ref. [10] in the main text).

The structure of this Supplementary Note 1 is as follows. The mathematical framework from Ref. [10] is briefly reviewed in section 1.1. In section 1.2 we present a derivation scheme and some useful relations and equalities, which will be used in section 1.3. Section 1.3 considers several example spatial stochastic point processes, and derives the equations for the moment and cumulant hierarchy, and the perturbation expansion, for each process separately. In section 1.4 we present a generalized derivation of these results which is valid for the whole class of point processes we consider. The resulting equations of evolution of the mean field solution, its corrections and the leading contribution to the cumulants are used to write the code of the Model Constructor, explained in section 1.5.

Contents

1.1 Mathematical framework 5

1.1.1 Locally finite configuration 5

1.1.2 Correlation functions 5

1.1.3 Cumulants 7

1.1.4 Perturbation expansion in the approximation of long-ranged interactions 8

1.2 Derivation scheme, some details of the derivation 10

1.2.1 Notations. 10

1.2.2 Derivation of the operator $\widehat{L}$ 10

1.2.3 Derivation of the operator L^{Δ} 11

1.2.4 Derivation of the operator Q^{Δ} 12

1.2.5 Derivations of operators $L^{\Delta}_{\epsilon,\text{ren}}$, $Q^{\Delta}_{\epsilon,\text{ren}}$ . . . 13
1.2.6 Derivation of functions H_q, H_p and H_g . . . 13
1.3 Derivations for selected basic processes . . . **14**
1.3.1 Processes defined by a single birth event by an operator $L_i^{x^+}$ . . . 14
Immigration, $L = L_i^{\text{IM}}(r)$ . . . 14
Birth to another type, $L = L_{ij}^{\text{BT}}(a)$ . . . 15
Birth, $L = L_i^{\text{B}}(a)$ . . . 19
Birth to another type by facilitation, $L = L_{kji}^{\text{BTF}}(a,b)$ . . . 19
Birth by facilitation, $L = L_{ji}^{\text{BF}}(a,b)$ . . . 26
1.3.2 Processes defined by a single death event by an operator $L_i^{x^-}$ . . . 27
Density independent death, $L = L_i^{\text{D}}(r)$ . . . 27
Death by external factor, $L = L_{ji}^{\text{DE}}(a)$ . . . 28
Death by competition, $L = L_i^{\text{C}}(a)$ . . . 32
1.3.3 Processes defined by one birth and one death events by an operator $L_{ij}^{x^- y^+}$ 33
Change in type, $L = L_{ji}^{\text{CT}}(r)$ . . . 33
Jump with a change in type $L = L_{ji}^{\text{JCT}}(a)$ . . . 35
Jump $L = L_i^{\text{J}}(a)$ . . . 38
Infection $L = L_{ji}^{\text{I}}(a)$ . . . 39
Birth to another type by consumption, $L = L_{kij}^{\text{BTC}}(a,b)$ . . . 46
Birth by consumption, $L = L_{ji}^{\text{BC}}(a,b)$ . . . 55
1.3.4 Processes defined by three birth-death events by an operator $L_{ijk}^{x^- y^- y^+}$. 56
Change in type by consumption, $L = L_{ijk}^{\text{CTC}}(a)$ . . . 56
1.4 Derivations for a general process . . . **65**
1.4.1 Formulation of a general process . . . 65
1.4.2 Derivation of the operator $\widehat{L}$ . . . 69
1.4.3 Derivation of the operator L^{Δ} . . . 71
1.4.4 Derivation of the operator Q^{Δ} . . . 73
1.4.5 Derivation of functions H_q, H_p and H_g . . . 75
1.4.6 The general case of spatially heterogeneous systems . . . 82
1.4.7 List of expressions for H_q, H_p and H_g for selected basic processes . . . 82
1.5 Transformation of equations for H_q, H_p and H_g for the use in toolboxes . . . **89**
1.5.1 Simplifications based on symmetry properties of the interaction function. 89
1.5.2 Equations in the real space . . . 94
1.5.3 Equations in the Fourier space . . . 95
1.5.4 The structure of the interaction function $r(\mathbf{P},\mathbf{R},\mathbf{C})$ . . . 96

1.1 Mathematical framework

In this section a brief review of the general mathematical framework for the analysis of stochastic spatial point processes developed in Ref.[10] is presented.

1.1.1 Locally finite configuration

First, we recall the notion of locally finite configuration γ, defined as a discrete subset $\gamma \subset \mathbb{R}^d$, such that in any bounded region $\Lambda \subset \mathbb{R}^d$ there is only a finite number of points belonging to γ, i.e. $|\gamma \cap \Lambda| < \infty$, where $|.|$ denotes a number of points in a discrete set. The space of locally finite configurations of the space $\mathbb{R}^d$ consists of all possible γ defined above, $\Gamma := \{\gamma\}$. Let μ be a probability measure on Γ, i.e. $\mu(\Gamma) = 1$. Consider a real-valued function F on Γ. The value of the function $F(\gamma)$ averaged over all possible $\gamma \in \Gamma$ is given by a pairing $\langle F, \mu \rangle$, where

$$\langle F, \mu \rangle := \int_\Gamma F(\gamma) d\mu(\gamma), \tag{1}$$

provided that the integral is well-defined. The evolution $t \mapsto \langle F, \mu(t) \rangle$ represents the evolution of probability measure $t \mapsto \mu(t)$ and it is given by the following equation

$$\frac{d}{dt} \langle F, \mu(t) \rangle = \langle LF, \mu(t) \rangle, \tag{2}$$

where L is a Markov operator on functions on Γ (see numerous examples below), and the initial condition is $\mu(0) = \mu_0$, where μ_0 is a probability measure on Γ.

1.1.2 Correlation functions

As shown in Ref. [10], the evolution of probability measure $\mu(t)$ can be reformulated as the evolution of their correlation functions $k^{(n)}(t)$. Correlation functions include the same description of the state of the system as the measure μ. Objects $k^{(n)}$ are called correlation functions (e.g. in Mathematical Physics), the n-th order spatial moments (e.g. in Theoretical Ecology),or factorial moments (in Probability Theory). The definition of $k^{(n)}$ functions is the following. Using symmetric functions $G^{(n)}(x_1, ..., x_n)$ with a compact support on $(\mathbb{R}^d)^n$, $n \geq 1$, and a constant $G^{(0)}$, the symmetric functions $k^{(n)}$ are said to be the correlation functions corresponding to measure μ iff

$$\langle \sum_{\{x_1,...,x_n\} \subset \gamma} G^{(n)}(x_1, ..., x_n), \mu \rangle = \frac{1}{n!} \int_{(\mathbb{R}^d)^n} G^{(n)}(x_1, ..., x_n) k^{(n)}(x_1, ..., x_n) dx_1 ... dx_n, \tag{3}$$

for any $n \in \mathbb{N}$, and setting $k^{(0)} := 1$.

To explain the meaning for correlation functions of orders $n = 1, 2$, consider an indicator function $\chi_\Lambda(x) = \begin{cases} 1, & x \in \Lambda \\ 0, & x \notin \Lambda \end{cases}$ for some bounded region $\Lambda \subset \mathbb{R}^d$, and take $G^{(1)}(x) = \chi_\Lambda(x)$. Then (3) is read as

$$\langle \sum_{x \in \Gamma} \chi_\Lambda(x), \mu \rangle = \int_{\mathbb{R}^d} \chi_\Lambda(x) k^{(1)}(x) dx,$$

or

$$\langle |\gamma \cap \Lambda|, \mu \rangle = \int_\Lambda k^{(1)}(x) dx.$$

Note that $\langle|\gamma \cap \Lambda|, \mu\rangle$ is just the expected value for the random number of points of γ in the given bounded region Λ. Then, by the well-known Markov's inequality, one can estimate the probability to find in Λ more than N points

$$\mathrm{Prob}\big\{|\gamma \cap \Lambda| > N\big\} \leq \frac{1}{N} \int_{\Lambda} k^{(1)}(x) dx.$$

On the other hand, by the Lebesgue theorem, if $B_r(x)$ is the ball centered at x and the radius $r > 0$, and if V_r is its volume, then

$$k^{(1)}(x) = \lim_{r \to 0} \frac{1}{V_r} \int_{B_r(x)} k^{(1)}(y) dy,$$

i.e. $k^{(1)}(x)$ is indeed the local density.

Similarly, for any bounded regions $\Lambda_1, \Lambda_2 \subset \mathbb{R}^d$, one can calculate the expected value of the product of random numbers $|\gamma \cap \Lambda_1|$ and $|\gamma \cap \Lambda_2|$:

$$\langle|\gamma \cap \Lambda_1|\,|\gamma \cap \Lambda_2|, \mu\rangle = \int_{\Lambda_1} \int_{\Lambda_2} k^{(2)}(x, y) dx dy + \int_{\Lambda_1 \cap \Lambda_2} k^{(1)}(x) dx.$$

In particular, one can get from this the corresponding covariance, correlation, variance and so on.

It is convenient to summarize (3) in n and to rewrite the result in a compact form. For this, let η be any finite subset of a configuration $\gamma \in \Gamma$, e.g. $\eta = \{x_1, ..., x_n\} \subset \gamma$ for $|\eta| = n$. In symbol this is denoted as $\eta \Subset \gamma$. Using this notation, one obtains:

$$G^{(0)} + \sum_{n=1}^{\infty} \sum_{\{x_1,...,x_n\} \subset \gamma} G^{(n)}(x_1, ..., x_n) = \sum_{\eta \Subset \gamma} G(\eta), \tag{4}$$

where $G(\eta) = G^{(n)}(x_1, ..., x_n)$ for $\eta = \{x_1, ..., x_n\}$. Introducing the notation of K-transform,

$$(KG)(\gamma) := \sum_{\eta \Subset \gamma} G(\eta), \tag{5}$$

the compact form of the expression (3) summarized in n becomes:

$$\langle KG, \mu\rangle = \langle\!\langle G, k \rangle\!\rangle, \tag{6}$$

where

$$\langle\!\langle G, k \rangle\!\rangle := G^{(0)} + \sum_{n=1}^{\infty} \frac{1}{n!} \int_{(\mathbb{R}^d)^n} G^{(n)}(x_1, ..., x_n) k^{(n)}(x_1, ..., x_n) dx_1 ... dx_n \tag{7}$$

$$= \int_{\Gamma_0} G(\eta) k(\eta) d\lambda(\eta). \tag{8}$$

In the latter expression Γ_0 denotes the set of all finite subsetes of $\mathbb{R}^d$, λ is a measure on Γ_0, i.e. λ is a Lebesgue measure on each $(\mathbb{R}^d)^n$ symmetrized by the factor $1/(n!)$.

Consider now

$$G^{(n)}(x_1, \ldots, x_n) = \big(\mathrm{e}^{\chi_\Lambda(x_1)} - 1\big) \ldots \big(\mathrm{e}^{\chi_\Lambda(x_n)} - 1\big)$$
$$= (\mathrm{e} - 1)^n \chi_\Lambda(x_1) \ldots \chi_\Lambda(x_n),$$

$G^{(0)} = 1$. It is straightforward to check then that

$$(KG)(\gamma) = \mathrm{e}^{|\gamma\cap\Lambda|};$$

therefore, (6) allows to find the characteristic function of the random number $|\gamma \cap \Lambda|$:

$$\langle \mathrm{e}^{|\gamma\cap\Lambda|}, \mu\rangle = 1 + \sum_{n=1}^{\infty} \frac{(\mathrm{e}-1)^n}{n!} \int_{\Lambda^n} k^{(n)}(x_1, \ldots, x_n) dx_1 \ldots dx_n. \tag{9}$$

For the function $F(\gamma) = (KG)(\gamma)$ using Eq.(6) the equation of the evolution (2) can be rewritten in terms of correlation functions. For the left and right hand sides of (2) one obtains:

$$\langle KG, \frac{\partial}{\partial t}\mu\rangle = \langle\!\langle G, \frac{\partial}{\partial t}k\rangle\!\rangle; \tag{10}$$

$$\langle LKG, \mu\rangle = \langle K(\widehat{L}G), \mu\rangle = \langle\!\langle (\widehat{L}G), k\rangle\!\rangle = \langle\!\langle G, L^{\Delta}k\rangle\!\rangle, \tag{11}$$

where

$$(\widehat{L}G)(\eta) := (K^{-1}LKG)(\eta), \tag{12}$$

where the inverse mapping K^{-1} is used, the operator L^{Δ} is determined by the last equality in (11), for details see Ref. [10]. As a result, the evolution of correlation functions of all orders is given by the following equation:

$$\frac{\partial}{\partial t}k(t,\eta) = \left(L^{\Delta}k\right)(t,\eta). \tag{13}$$

1.1.3 Cumulants

For a given Markov operator L, the equation (13) on the correlation functions is one of the main object of interest. Often, instead of correlation functions it is convenient to consider cumulants, also known as truncated correlation functions or semi-invariants (second cumulant is also called "spatial covariance", or "truncated 2-point correlation function", or the "second central moment").

By (9), we see that the correlation functions k^n can be interpreted as (factorial) moments for the generating function of $\mathrm{e}^{|\gamma\cap\Lambda|}$. By the definition, cumulants $u^{(n)}$ are the similar moments for the logarithm of the generating function of $\mathrm{e}^{|\gamma\cap\Lambda|}$, i.e.

$$\log\langle \mathrm{e}^{|\gamma\cap\Lambda|}, \mu\rangle = \sum_{n=1}^{\infty} \frac{(\mathrm{e}-1)^n}{n!} \int_{\Lambda^n} u^{(n)}(x_1, \ldots, x_n) dx_1 \ldots dx_n; \tag{14}$$

note that $u^{(0)} := 0$.

There exists one-to-one correspondence between correlation functions with $k^{(0)} = 1$ and cumulants with $u^{(0)} = 0$. In particular, cumulants may be expressed through the correlation functions, see Ref. [10]:

$$\begin{aligned} u^{(0)} &:= 0; & (15)\\ u^{(1)}(x) &:= k^{(1)}(x); & (16)\\ u^{(2)}(x_1,x_2) &:= k^{(2)}(x_1,x_2) - k^{(1)}(x_1)k^{(1)}(x_2), \end{aligned}$$

The opposite relation is given by

$$k(t,\eta) = (\exp^{*} u)(t,\eta), \tag{17}$$

where

$$(\exp^* u)(\eta) = 1^*(\eta) + \sum_{n=1}^{\infty} \frac{1}{n!} \sum_{\eta_1 \sqcup \ldots \sqcup \eta_n = \eta} u(\eta_1)\ldots u(\eta_n), \tag{18}$$

$$1^*(\eta) = 0^{|\eta|} = \mathbb{1}_{|\eta|=0} = \begin{cases} 1, & \eta = \varnothing; \\ 0, & \eta \neq \varnothing, \end{cases} \tag{19}$$

the symbol $\sqcup$ denotes a disjoint union. For any functions u, v on Γ_0 a disjoint union is determined as follows:

$$\sum_{\eta_1 \sqcup \eta_2 = \eta} u(\eta_1)v(\eta_2) = \sum_{\xi \subset \eta} u(\xi)v(\eta \backslash \xi) =: (u * v)(\eta), \tag{20}$$

where the symbol $*$ defines a convolution. It has been shown [10] that the evolution of cumulants is described by the following equation:

$$\frac{\partial}{\partial t} u(t, \eta) = (Q^{\Delta} u)(t, \eta), \tag{21}$$

where the operator Q^{Δ} can be found from the operator L^{Δ} :

$$Q^{\Delta} u := (\exp^{*-1} u) * \left(L^{\Delta}(\exp^* u)\right). \tag{22}$$

1.1.4 Perturbation expansion in the approximation of long-ranged interactions

In general, equations (13) and (22) for $k^{(n)}$ and $u^{(n)}$ functions depend on $k^{(n+1)}$ and $u^{(n+1)}$ correspondingly. Therefore, the solution in closed form does not exist. In Ref. [9] it has been shown, that in systems where the Markov operator L includes pairwise interaction between points in locally finite configuration it is possible to identify a small parameter, and to find a solution of equations (13) and (21) in a closed form using perturbation theory in the approximation of long-ranged interactions.

Let a be an interaction kernel which describes pairwise interactions between points in the system, $a : \mathbb{R}^d \to \mathbb{R}_+ := [0, \infty)$, $a \in L^1(\mathbb{R}^d)$. The kernel is a symmetric function, $a(-x) = a(x)$. The kernel can be made more long-ranged by the following scaling:

$$a_{\epsilon}(x) := \epsilon^d a(\epsilon x), \tag{23}$$

where $\epsilon > 0$ and $\epsilon \to 0$. After rescaling, L^{Δ} changes into L^{Δ}_{ϵ} which is the same as L^{Δ} with an additional ϵ in each kernels' subscript. In order to have a reasonable limit as $\epsilon \to 0$, the operator L^{Δ}_{ϵ} is renormalized, and denoted as $L^{\Delta}_{\epsilon,\mathrm{ren}}$,

$$L^{\Delta}_{\epsilon,\mathrm{ren}} := S_{\epsilon^{-1}} L^{\Delta}_{\epsilon} S_{\epsilon}, \tag{24}$$

where $(S_{\epsilon} k)(\eta) := k(\epsilon \eta)$, and $\epsilon \eta := \{\epsilon x | x \in \eta\}$. Correlation functions in the rescaled and renormalized system, denoted as k_{ϵ}, satisfy the following equation of the evolution:

$$\frac{\partial}{\partial t} k_{\epsilon}(t, \eta) = (L^{\Delta}_{\epsilon,\mathrm{ren}} k_{\epsilon})(t, \eta). \tag{25}$$

Cumulants that corresponds to k_{ϵ} are denoted by u_{ϵ}. The evolution of u_{ϵ} is governed by the

operator $Q^{\Delta}_{\epsilon,\mathrm{ren}}$ obtained from the operator Q^{Δ} in the same way as described above:

$$Q^{\Delta}_{\epsilon,\mathrm{ren}} := S_{\epsilon^{-1}} Q^{\Delta}_{\epsilon,\mathrm{ren}} S_{\epsilon}, \tag{26}$$

$$\frac{\partial}{\partial t} u_{\epsilon}(t) = Q^{\Delta}_{\epsilon,\mathrm{ren}} u_{\epsilon}(t). \tag{27}$$

Considering ϵ^d as a small parameter, $\epsilon^d \to 0$, a perturbation expansion can be developed [10]:

$$\begin{aligned} L^{\Delta}_{\epsilon,\mathrm{ren}} &= A + \epsilon^d B + o(\epsilon^d); \\ Q^{\Delta}_{\epsilon,\mathrm{ren}} &= \widetilde{A} + \epsilon^d \widetilde{B} + o(\epsilon^d). \end{aligned} \tag{28}$$

Solution for cumulants is considered as a perturbation series in powers of ϵ^d,

$$u_{\epsilon}(t,\eta) = v(t,\eta) + \epsilon^d w(t,\eta) + o(\epsilon^d). \tag{29}$$

It has been shown [10], that $v(t,\eta)$ is non-zero only on one-point configuration:

$$v(t,\eta) = \mathbb{1}_{|\eta|=1} q(t,\eta). \tag{30}$$

In a pointwise limit, as $\epsilon^d \to 0$, the interactions become global, therefore one would recover a mean-field limit [10], also known as mesoscopic limit, where $q(t,x)$ describes the density of individuals at time t.

The correction w in (29) consists of two components w_1 and w_2, where w_1 is non-zero only on one-point configuration, and w_2 is non-zero only on two-point configurations:

$$w(t,\eta) := w_1 + w_2; \quad w_1(t,\eta) := \mathbb{1}_{|\eta|=1}\, p(t,\eta); \quad w_2(t,\eta) := \mathbb{1}_{|\eta|=2}\, g(t,\eta), \tag{31}$$

$$\mathbb{1}_{|\eta|=1} = \begin{cases} 1, & |\eta| = 1; \\ 0, & |\eta| \neq 1; \end{cases} \qquad \mathbb{1}_{|\eta|=2} = \begin{cases} 1, & |\eta| = 2; \\ 0, & |\eta| \neq 2. \end{cases} \tag{32}$$

Substituting (28)-(31) into (27), and considering separately equations for $|\eta| = 1$ and $|\eta| = 2$ for leading and subleading order of small parameter ϵ^d one obtains equations for the mean field q, its correction p, and for a second order cumulant g:

$$\begin{aligned} \frac{\partial}{\partial t} q(t,x) &= H_q(t,x); \\ \frac{\partial}{\partial t} p(t,x) &= H_p(t,x); \\ \frac{\partial}{\partial t} g(t,x,y) &= H_g(t,x,y). \end{aligned} \tag{33}$$

Thus, one obtains the expansion for the first and second order cumulants in approximation of long-ranged interactions,

$$\begin{aligned} u^{(1)}_{\epsilon}(t,x) &= q(t,x) + \epsilon^d p(t,x) + o(\epsilon^d), \\ u^{(2)}_{\epsilon}(t,x,y) &= \epsilon^d g(t,x,y) + o(\epsilon^d). \end{aligned} \tag{34}$$

1.2 Derivation scheme, some details of the derivation

This section presents a scheme of the derivation used in the next section. Also some useful transformations and equalities used during the derivation are presented. Some more detailed derivations and some mathematical proofs related to the material in this section can be found in Ref. [10].

1.2.1 Notations.

Throughout, we consider that the configuration γ describes N types of individuals or particles. Thus, the full configuration consists of configurations for each type, $\gamma = (\gamma_1, ..., \gamma_N)$. Analogously, the finite subset η of a configuration γ is represented as $\eta = (\eta_1, ..., \eta_N)$. Coordinate x of a particle of type i is denoted as $x^{(i)}$,

$$x^{(i)} := (\xi_1, ..., \xi_i, ..., \xi_N), \qquad \xi_j = \begin{cases} \varnothing, & j \neq i; \\ \{x\}, & j = i; \end{cases} \quad j = 1, ..., N. \tag{35}$$

The notation $x \in \eta_i$ is equivalent to $x^{(i)} \in \eta$. When a particle of a certain type is added or subtracted from a configuration γ, this is denoted by the following notations:

$$\begin{aligned} (\gamma_1, ..., \gamma_i \backslash x, ..., \gamma_N) &= \gamma \backslash x^{(i)}; \\ (\gamma_1, ..., \gamma_i \cup x, ..., \gamma_N) &= \gamma \cup x^{(i)}; \\ (\gamma_1, ..., \gamma_i \backslash x, ..., \gamma_j \cup y, ..., \gamma_N) &= (\gamma \backslash x^{(i)}) \cup y^{(j)}. \end{aligned}$$

For the sake of brevity, the following notations are introduced:

$$\begin{aligned} (L_i^{x^+} F)(\gamma) &= F(\gamma \cup x^{(i)}) - F(\gamma); \\ (L_i^{x^-} F)(\gamma) &= F(\gamma \backslash x^{(i)}) - F(\gamma); \\ (L_{ii}^{x^+ y^-} F)(\gamma) &= F(\gamma \cup x^{(i)} \backslash y^{(i)}) - F(\gamma); \\ (L_{ij}^{x^+ y^-} F)(\gamma) &= F(\gamma \cup x^{(i)} \backslash y^{(j)}) - F(\gamma); \\ (L_{ijk}^{x^- y^- z^+} F)(\gamma) &= F(\gamma \backslash x^{(i)} \backslash y^{(j)} \cup z^{(k)}) - F(\gamma). \end{aligned} \tag{36}$$

1.2.2 Derivation of the operator $\widehat{L}$

The operator $\widehat{L}$ is defined as $(\widehat{L}G)(\eta) := (K^{-1}LKG)(\eta)$, where

$$(K^{-1}F)(\eta) := \sum_{\xi \subset \eta} (-1)^{|\eta \backslash \xi|} (LF)(\xi), \tag{37}$$

$$(KG)(\gamma) := \sum_{\eta \Subset \gamma} G(\eta), \tag{38}$$

where $\eta \Subset \gamma$ means $\eta \subset \gamma$ and $|\eta| < \infty$. The expression for $(\widehat{L}G)(\eta)$ is then transformed using the following equalities. For a given $x \in \gamma$, we have

$$\sum_{\eta \Subset \gamma} G(\eta) = \sum_{\eta \Subset \gamma \backslash x} G(\eta) + \sum_{\eta \Subset \gamma \backslash x} G(\eta \cup x).$$

It is convenient to introduce the operation $D_x^{(i)}$ which adds a particle of type i at coordinate x :

$$(D_x^{(i)} u)(\eta) = u(\eta \cup x^{(i)}). \tag{39}$$

Therefore, using $F = KG$, first two equations from (36) become:

$$\begin{aligned}(L_i^{x^-} KG)(\xi) = KG(\xi \backslash x^{(i)}) - KG(\xi) &= \sum_{\eta \Subset \xi \backslash x^{(i)}} G(\eta) - \sum_{\eta \Subset \xi \backslash x^{(i)}} G(\eta) - \sum_{\eta \Subset \xi \backslash x^{(i)}} G(\eta \cup x^{(i)}) \\ &= - \sum_{\eta \Subset \xi \backslash x^{(i)}} (D_x^{(i)} G)(\eta) \\ &= -(K D_x^{(i)} G)(\xi \backslash x^{(i)}), \end{aligned} \tag{40}$$

$$\begin{aligned}(L_i^{x^+} KG)(\xi) = KG(\xi \cup x^{(i)}) - KG(\xi) &= \sum_{\eta \Subset \xi} G(\eta) + \sum_{\eta \Subset \xi} G(\eta \cup x^{(i)}) - \sum_{\eta \Subset \xi} G(\eta) \\ &= \sum_{\eta \Subset \xi} (D_x^{(i)} G)(\eta) \\ &= (K D_x^{(i)} G)(\xi). \end{aligned} \tag{41}$$

Also, during the derivation of $\widehat{L}$ we use that $\eta \backslash \xi \backslash x = \eta \backslash x \backslash \xi$; and $\sum_{x \in \eta} 1 = |\eta|$; and the following equalities: for $x \neq y$,

$$\sum_{y^{(j)} \in \eta} \sum_{x^{(i)} \in \eta \backslash y^{(j)}} = \sum_{x^{(i)} \in \eta} \sum_{y^{(j)} \in \eta \backslash x^{(i)}} ; \tag{42}$$

for any function $F(\xi, x)$ on $\Gamma_0 \times \mathbb{R}^d$,

$$\sum_{\xi \subset \eta} \sum_{x \in \xi} F(\xi, x) = \sum_{x \in \eta} \sum_{\xi \subset \eta \backslash x} F(\xi \cup x, x). \tag{43}$$

1.2.3 Derivation of the operator L^Δ

As follows from (8) and (11), the operator L^Δ is derived from the following equality,

$$\int_{\Gamma_0} (\widehat{L} G)(\eta) k(\eta) d\lambda(\eta) = \int_{\Gamma_0} G(\eta)(L^\Delta k)(\eta) d\lambda(\eta), \tag{44}$$

by transforming the integral in the left hand side to the same structure as the integral in the right hand side. In some integrals one can change the variable of integration by using the substitution $\eta = \eta' \cup x^{(i)}$ leading to the following transformation:

$$\int_{\Gamma_0} \sum_{x^{(i)} \in \eta} G(\eta \backslash x^{(i)}) k(\eta) d\lambda(\eta) = \int_{\Gamma_0} \int_{\mathbb{R}^d} dx G(\eta') k(\eta' \cup x^{(i)}) d\lambda(\eta'). \tag{45}$$

Correspondingly, the change of variable $\eta = \eta' \backslash x^{(i)}$ leads to the following transformation:

$$\int_{\Gamma_0} \int_{\mathbb{R}^d} dx G(\eta \cup x^{(i)}) k(\eta) d\lambda(\eta) = \int_{\Gamma_0} \sum_{x^{(i)} \in \eta'} G(\eta') k(\eta' \backslash x^{(i)}) d\lambda(\eta'). \tag{46}$$

1.2.4 Derivation of the operator Q^{Δ}

From calculations it follows, that in all processes considered in this work and specified in next sections the operators Q^{Δ} and $Q^{\Delta}_{\epsilon,\text{ren}}$ have the following structure:

$$Q^{\Delta} = L^{\Delta} + M^{\Delta}; \tag{47}$$

$$Q^{\Delta}_{\epsilon,\text{ren}} = L^{\Delta}_{\epsilon,\text{ren}} + M^{\Delta}_{\epsilon,\text{ren}} + o(\epsilon^d), \tag{48}$$

where operators L^{Δ} and M^{Δ} represent linear and nonlinear parts correspondingly, as explained below. In order to find Q^{Δ}, one should substitute $\widehat{L}$ into the left hand side of the following equation:

$$\int_{\Gamma_0} \left(\widehat{L} e_{\lambda}(\theta)\right)(\eta)(\exp^{*} u)(\eta) d\lambda(\eta) = \int_{\Gamma_0} e_{\lambda}(\theta,\eta) \left(L^{\Delta}(\exp^{*} u)\right)(\eta) d\lambda(\eta), \tag{49}$$

where

$$e_{\lambda}(\theta,\eta) := \prod_{x\in\eta} \theta(x), \qquad e_{\lambda}(\theta,\varnothing) := 1. \tag{50}$$

For simplicity, it is assumed that θ has a compact support $\lambda \subset \mathbb{R}^d$. Then, the following definition is used:

$$Q^{\Delta} u := (\exp^{*-1} u) * \left(L^{\Delta}(\exp^{*} u)\right). \tag{51}$$

Using (47) and the following equalities derived in Ref. [10],

$$1^{*} * v \;=\; v, \tag{52}$$

$$(\exp^{*} u) * (\exp^{*-1} u) \;=\; 1^{*}, \tag{53}$$

the right hand side of the equation (49) becomes

$$\int_{\Gamma_0} e_{\lambda}(\theta,\eta) \left((\exp^{*} u) * (L^{\Delta} + M^{\Delta})u\right)(\eta) d\lambda(\eta) = $$

$$= \int_{\Gamma_0} e_{\lambda}(\theta,\xi)(\exp^{*} u)(\xi) d\lambda(\xi) \int_{\Gamma_0} e_{\lambda}(\theta,\eta)((L^{\Delta} + M^{\Delta})u)(\eta) d\lambda(\eta). \tag{54}$$

Changing variables where it is needed according to (45) and (46), we transform the left hand side of the equation (49) so that the function $e_{\lambda}(\theta,\eta)$ enters the expression in the same way as it does in the right hand side of the equation (49). If needed, one can use $e_{\lambda}(\theta,\eta\cup x) = e_{\lambda}(\theta,\eta)\theta(x)$. All sums over coordinates in η should be transformed into integrals over all space $\mathbb{R}^d$ using the change of variables (45). The addition of particles should be represented in terms of corresponding operators $D_x^{(i)}$ using their definition (39). Resulting expressions of most of processes considered here will contain either one D_x operation in the integrand function, $D_x(\exp^{*} u)$, or two, $D_x D_y(\exp^{*} u)$. Those processes where the resulting expressions do not contain D_x operations are considered separately. According to Ref. [10] one gets:

$$D_y(u * v) = (D_y u) * v + u * (D_y v), \tag{55}$$

and for $u(\varnothing)=0$, as is the case here shown in (15), this means

$$D_x(\exp^* u) = D_x u * (\exp^* u), \tag{56}$$

$$D_x^{(i)} D_y^{(j)}(\exp^* u) = D_x^{(i)} D_y^{(j)} u * (\exp^* u) + D_y^{(j)} u * D_x^{(i)} u * (\exp^* u). \tag{57}$$

As shown in Ref. [10], only those terms contribute to L^Δ which contain only a single convolution, e.g. $D_x u * (\exp^* u)$ and $D_x^{(i)} D_y^{(j)} u * (\exp^* u)$. This is due to the fact that the inner structure of the function $(\exp^* u)$ has not been revealed in these terms, thus the function $(\exp^* u)$ acts as a k function in equation (44) determining L^Δ. In this cases the expression for $(L^\Delta u)(\eta)$ is given by the expression for $(L^\Delta k)(\eta)$ after using u instead of k. Terms containing more than one convolution are generated due to particular properties of the function $(\exp^* u)$, therefore they determine nonlinear part denoted as M^Δ. To find M^Δ one can use the following substitution rule:

$$D_x^{(i)} D_y^{(j)} \exp^* u \longrightarrow D_y^{(j)} u * D_x^{(i)} u. \tag{58}$$

The resulting expression will contain the operator M^Δ,

$$\int_{\Gamma_0} e_\lambda(\theta,\eta)\left(M^\Delta u\right)(\eta) d\lambda(\eta). \tag{59}$$

1.2.5 Derivations of operators $L^\Delta_{\epsilon,\mathrm{ren}}$, $Q^\Delta_{\epsilon,\mathrm{ren}}$

In order to find $L^\Delta_{\epsilon,\mathrm{ren}}$ and $Q^\Delta_{\epsilon,\mathrm{ren}} = L^\Delta_{\epsilon,\mathrm{ren}} + M^\Delta_{\epsilon,\mathrm{ren}}$ one should obtain L^Δ_ϵ and M^Δ_ϵ from L^Δ and M^Δ by introducing a subscript ϵ to all kernels, e.g. replacing a by a_ϵ. From direct computations, using $a_\epsilon(\epsilon^{-1}x - \epsilon^{-1}y) = \epsilon^d a(x-y)$, and $S_{\epsilon^{-1}} \int a(x-y)dx = \int a(x - \epsilon^{-1}y)dx$ (i.e. that the operator $S_{\epsilon^{-1}}$ does not affect the variable of integration), the following expressions are obtained:

$$(L^\Delta_{\epsilon,\mathrm{ren}} k)(\eta) = (S_{\epsilon^{-1}} L^\Delta_\epsilon S_\epsilon k)(\eta) = \left((A + \epsilon^d B)k\right)(\eta);$$
$$(M^\Delta_{\epsilon,\mathrm{ren}} k)(\eta) = (S_{\epsilon^{-1}} M^\Delta_\epsilon S_\epsilon k)(\eta) = \left((C + \epsilon^d D)k\right)(\eta). \tag{60}$$

Thus, operators A, B, C, D determine the perturbation expansion for operators $L^\Delta_{\epsilon,\mathrm{ren}}$ and $Q^\Delta_{\epsilon,\mathrm{ren}}$.

1.2.6 Derivation of functions H_q, H_p and H_g

Applying perturbation expansion for u_ϵ, Eq. (29), and for $Q^\Delta_{\epsilon,\mathrm{ren}}$, Eq.(60), one obtains:

$$\frac{\partial}{\partial t} v(\eta) = (Av + Cv)(\eta); \tag{61}$$

$$\frac{\partial}{\partial t} w(\eta) = (Aw + Bv + Dv + C(v,w) + C(w,v))(\eta). \tag{62}$$

where in (62) it is taken into account that the cumulant u enters the function $C(u)$ twice, therefore the notation $C(v,w)$ denotes that the first occurrence of u is given by v and the second occurrence of u is given by w, the analogous for $C(w,v)$.

Considering some species m and n, and using $\eta = \{x^{(m)}\}$ for one-point configuration, and $\eta = \{x_1^{(m)}, x_2^{(n)}\}$ for two-point configuration, one can use:

$$v(\eta) = \mathbb{1}_{\eta=\{x^{(m)}\}} q_m(x); \tag{63}$$

$$w(\eta) = \mathbb{1}_{\eta=\{x^{(m)}\}} p_m(x) + \mathbb{1}_{\eta=\{x_1^{(m)}, x_2^{(n)}\}} g_{mn}(x_1, x_2), \tag{64}$$

where $q_m(x) := q(x^{(m)})$, $p_m(x) := p(x^{(m)})$; $g_{mn}(x_1,x_2) := g(x_1^{(m)}, x_2^{(n)})$. Substituting these expressions into (61) and (62) one obtains H functions from (33):

$$\begin{aligned} H_{q_m}(x) &= (Av + Cv)(\eta)\big|_{\eta=\{x^{(m)}\}}\,; && (65)\\ H_{p_m}(x) &= (Aw + Bv + Dv + C(v,w) + C(w,v))(\eta)\big|_{\eta=\{x^{(m)}\}}\,; && (66)\\ H_{g_{mn}}(x_1,x_2) &= (Aw + Bv + Dv + C(v,w) + C(w,v))(\eta)\big|_{\eta=\{x_1^{(m)},x_2^{(n)}\}}\,. && (67)\end{aligned}$$

In the translationally invariant case functions q and p are constants: $q_m(x) = q_m$, $p_m(x) = p_m$; the function g has the following properties: $g_{mn}(x_1,x_2) = g_{mn}(x_1 - x_2)$, and $g_{mn}(x) = g_{mn}(-x)$, and $g_{mn}(x) = g_{nm}(x)$. The same applies to H functions: $H_{q_m}(x) = H_{q_m}$, $H_{p_m}(x) = H_{p_m}$; $H_{g_{mn}}(x_1,x_2) = H_{g_{mn}}(x_1 - x_2)$.

1.3 Derivations for selected basic processes

In this section several different basic spatial stochastic point processes are considered. The derivation of hierarchy of equations for correlation functions and cumulants is presented. The perturbation expansion in the approximation of long-ranged interactions is applied, and the equations of evolution of the mean field solution, its corrections and the leading contribution to the cumulants are derived.

1.3.1 Processes defined by a single birth event by an operator $L_i^{x^+}$.

Immigration, $L = L_i^{\mathrm{IM}}(r)$.

Process definition: an immigrant of type i appears spontaneously at location x with rate per unit area r,

$$(L_i^{\mathrm{IM}}(r)F)(\gamma) = r\int_{\mathbb{R}^d}(L_i^{x^+}F)(\gamma)dx. \qquad (68)$$

Using $F = KG$, the expression for $\widehat{L}G$ follows from its definition:

$$\begin{aligned}(\widehat{L}G)(\eta) &= (K^{-1}L_i^{\mathrm{IM}}(r)KG)(\eta)\\ &= r\sum_{\xi\subset\eta}(-1)^{|\eta\setminus\xi|}\int_{\mathbb{R}^d}dx(KD_x^{(i)}G)(\xi)\\ &= r\int_{\mathbb{R}^d}dx(D_x^{(i)}G)(\eta).\end{aligned}$$

Substituting $\widehat{L}G$ and using a change of variables $\eta\to\eta\setminus x^{(i)}$ one can derive L^{Δ} :

$$\begin{aligned}\int_{\Gamma_0}(\widehat{L}G)(\eta)k(\eta)d\lambda(\eta) &= r\int_{\Gamma_0}\int_{\mathbb{R}^d}dxG(\eta\cup x^{(i)})k(\eta)d\lambda(\eta)\\ &= \int_{\Gamma_0}G(\eta)\left(r\sum_{x^{(i)}\in\eta}k(\eta\setminus x^{(i)})\right)d\lambda(\eta),\\ (L^{\Delta}k)(\eta) &= r\sum_{x^{(i)}\in\eta}k(\eta\setminus x^{(i)}).\end{aligned}$$

In order to find Q^Δ, one substitutes $\widehat{L}$ into the following expression, and transforms this expression so that it contains $e_\lambda(\theta,\eta)$ explicitly. Using the equality $e_\lambda(\theta,\eta\cup x) = e_\lambda(\theta)\theta(x)$, one obtains:

$$\int_{\Gamma_0}\left(\widehat{L}e_\lambda(\theta)\right)(\eta)(\exp^* u)(\eta)d\lambda(\eta) = \int_{\Gamma_0} r\int_{\mathbb{R}^d} dx e_\lambda(\theta,\eta\cup x^{(i)})(\exp^* u)(\eta)d\lambda(\eta)$$
$$= \int_{\Gamma_0} r\int_{\mathbb{R}^d} dx e_\lambda(\theta,\eta)\theta(x^{(i)})(1^* \star \exp^* u)(\eta)d\lambda(\eta)$$
$$= \int_{\Gamma_0} r\int_{\mathbb{R}^d} dx e_\lambda(\theta,\eta\cup x^{(i)})1^*(\eta)d\lambda(\eta)\int_{\Gamma_0} e_\lambda(\theta,\xi)(\exp^* u)(\xi)d\lambda(\xi)$$
$$= \int_{\Gamma_0} e_\lambda(\theta,\eta)\left(r\sum_{x^{(i)}\in\eta} 1^*(\eta\backslash x^{(i)})\right)d\lambda(\eta)\int_{\Gamma_0} e_\lambda(\theta,\xi)(\exp^* u)(\xi)d\lambda(\xi).$$

This leads to the following:

$$\begin{aligned}(Q^\Delta u)(\eta) &= r\sum_{x^{(i)}\in\eta} 1^*(\eta\backslash x^{(i)}) = r\mathbb{1}_{\eta=\{x^{(i)}\}};\\ \frac{\partial u}{\partial t} &= r\mathbb{1}_{\eta=\{x^{(i)}\}}.\end{aligned} \tag{69}$$

Due to the absence of spatial kernels, $Q^\Delta_{\epsilon,\mathrm{ren}} = Q^\Delta$. Applying perturbation expansion in the limit of long-ranged interactions, one obtains:

$$\begin{aligned}H^{\mathrm{IM}}_{q_m}(x) &= r\delta_{im};\\ H^{\mathrm{IM}}_{p_m}(x) &= 0;\\ H^{\mathrm{IM}}_{g_{mn}}(x_1,x_2) &= 0.\end{aligned} \tag{70}$$

Birth to another type, $L = L^{\mathrm{BT}}_{ij}(a)$

Process definition: existing individuals of type j produce new individuals of type i with the kernel a, which incorporates both fecundity and dispersal,

$$(L^{\mathrm{BT}}_{ij}(a)F)(\gamma) = \sum_{y\in\gamma_j}\int_{\mathbb{R}^d} a(x-y)(L^{x^+}_i F)(\gamma)dx. \tag{71}$$

Using $F = KG$, the expression for $\widehat{L}G$ is the following,

$$\begin{aligned}(\widehat{L}G)(\eta) &= (K^{-1}L^{\mathrm{BT}}_{ij}(a)KG)(\eta)\\ &= \sum_{\xi\subset\eta}(-1)^{|\eta\backslash\xi|}\sum_{y^{(j)}\in\xi}\int_{\mathbb{R}^d} a(x-y)(KD^{(i)}_x G)(\xi)dx.\end{aligned}$$

First, the change of variables $\xi\to\xi\cup y^{(j)}$ is used, and the sequence of summations is changed as shown in (43),

$$\begin{aligned}\widehat{L}G(\eta) &= \sum_{y^{(j)}\in\eta}\sum_{\xi\subset\eta\backslash y^{(j)}}(-1)^{|(\eta\backslash y^{(j)})\backslash\xi|}\int_{\mathbb{R}^d} a(x-y)(KD^{(i)}_x G)(\xi\cup y^{(j)})dx\\ &= \sum_{y^{(j)}\in\eta}\sum_{\xi\subset\eta\backslash y^{(j)}}(-1)^{|(\eta\backslash y^{(j)})\backslash\xi|}\int_{\mathbb{R}^d} a(x-y)(D^{(j)}_y(KD^{(i)}_x G))(\xi)dx.\end{aligned}$$

Then, changing the sequence of D and K operators according to (41),

$$(D_y^{(j)}(KD_x^{(i)}G))(\eta\backslash y^{(j)}) = (KD_x^{(i)}G)(\eta\backslash y^{(j)}) + (KD_y^{(j)}D_x^{(i)}G)(\eta\backslash y^{(j)}),$$

and applying the inverse K−transform one obtains

$$\widehat{L}G(\eta) = \sum_{y^{(j)}\in\eta}\int_{\mathbb{R}^d} a(x-y)\left[(D_x^{(i)}G)(\eta\backslash y^{(j)}) + (D_y^{(j)}D_x^{(i)}G)(\eta\backslash y^{(j)})\right]dx.$$

Using $\widehat{L}G$ one can derive L^Δ, using the change of variables $\eta\to\eta\cup y^{(j)}$ in the first term, and then changing variables $\eta\to\eta\backslash x^{(i)}$ in both terms:

$$\begin{aligned}\int_{\Gamma_0}(\widehat{L}G)(\eta)k(\eta)d\lambda(\eta) &= \int_{\Gamma_0}\sum_{y^{(j)}\in\eta}\int_{\mathbb{R}^d} a(x-y)\left[G((\eta\cup x^{(i)})\backslash y^{(j)}) + G(\eta\cup x^{(i)})\right]dxk(\eta)d\lambda(\eta)\\ &= \int_{\Gamma_0}\int_{\mathbb{R}^d}\int_{\mathbb{R}^d} a(x-y)G(\eta\cup x^{(i)})dxdyk(\eta\cup y^{(j)})d\lambda(\eta)\\ &\quad+\int_{\Gamma_0}\sum_{y^{(j)}\in\eta}\int_{\mathbb{R}^d} a(x-y)G(\eta\cup x^{(i)})dxk(\eta)d\lambda(\eta)\\ &= \int_{\Gamma_0} G(\eta)\left(\sum_{x^{(i)}\in\eta}\int_{\mathbb{R}^d} a(x-y)k((\eta\cup y^{(j)})\backslash x^{(i)})dy\right.\\ &\quad\left.+\sum_{y^{(j)}\in\eta}\sum_{x^{(i)}\in\eta\backslash y^{(j)}} a(x-y)k(\eta\backslash x^{(i)})\right)d\lambda(\eta);\end{aligned}$$

$$\begin{aligned}(L^\Delta k)(\eta) &= \sum_{x^{(i)}\in\eta}\int_{\mathbb{R}^d} a(x-y)k((\eta\cup y^{(j)})\backslash x^{(i)})dy\\ &\quad+\sum_{y^{(j)}\in\eta}\sum_{x^{(i)}\in\eta\backslash y^{(j)}} a(x-y)k(\eta\backslash x^{(i)}).\end{aligned}\tag{72}$$

In order to find Q^Δ, one substitutes $\widehat{L}$ into the following expression,

$$\begin{aligned}&\int_{\Gamma_0}\left(\widehat{L}e_\lambda(\theta)\right)(\eta)(\exp^* u)(\eta)d\lambda(\eta) =\\ &= \int_{\Gamma_0}\sum_{y^{(j)}\in\eta}\int_{\mathbb{R}^d} a(x-y)(D_x^{(i)}e_\lambda(\theta))(\eta\backslash y^{(j)})dx(\exp^* u)(\eta)d\lambda(\eta)\\ &\quad+\int_{\Gamma_0}\sum_{y^{(j)}\in\eta}\int_{\mathbb{R}^d} a(x-y)(D_y^{(j)}D_x^{(i)}e_\lambda(\theta))(\eta\backslash y^{(j)})dx(\exp^* u)(\eta)d\lambda(\eta).\end{aligned}$$

Then, transforming this expression so that it contains $e_\lambda(\theta,\eta)$ explicitly, and transforming all summations into integrals over all space $\mathbb{R}^d$, and expressing the resulting formula in terms of D_x, D_y, one obtains:

$$\begin{aligned}&\int_{\Gamma_0}\int_{\mathbb{R}^d}dy\int_{\mathbb{R}^d}dxa(x-y)e_\lambda(\theta,\eta)\theta(x^{(i)})D_y^{(j)}(\exp^* u)(\eta)d\lambda(\eta)+\\ &\quad+\int_{\Gamma_0}\int_{\mathbb{R}^d}dy\int_{\mathbb{R}^d}dxa(x-y)e_\lambda(\theta,\eta)\theta(y^{(j)})\theta(x^{(i)})D_y^{(j)}(\exp^* u)(\eta)d\lambda(\eta).\end{aligned}$$

Terms with more than one operation D are absent here, therefore here $Q^{\Delta} = L^{\Delta}$. In order to find $Q^{\Delta}_{\epsilon,\text{ren}}$ one obtains Q^{Δ}_{ϵ} from Q^{Δ} by replacing potential a by a_ϵ,

$$\begin{aligned}(Q^{\Delta}_{\epsilon}u)(\eta) &= \sum_{x^{(i)}\in\eta}\int_{\mathbb{R}^d} a_\epsilon(x-y)u((\eta\cup y^{(j)})\backslash x^{(i)})dy \\ &+ \sum_{y^{(j)}\in\eta}\sum_{x^{(i)}\in\eta\backslash y^{(j)}} a_\epsilon(x-y)u(\eta\backslash x^{(i)}).\end{aligned}$$

Using $a_\epsilon(\epsilon^{-1}x-\epsilon^{-1}y) = \epsilon^d a(x-y)$, and $S_{\epsilon^{-1}}\int a(x-y)dx = \int a(x-\epsilon^{-1}y)dx$, one obtains:

$$\begin{aligned}(S_{\epsilon^{-1}}Q^{\Delta}_{\epsilon}S_\epsilon u)(\eta) &= S_{\epsilon^{-1}}\sum_{x^{(i)}\in\eta}\int_{\mathbb{R}^d} a_\epsilon(x-y)u(\epsilon((\eta\cup y^{(j)})\backslash x^{(i)}))dy \\ &+S_{\epsilon^{-1}}\sum_{y^{(j)}\in\eta}\sum_{x^{(i)}\in\eta\backslash y^{(j)}} a_\epsilon(x-y)u(\epsilon(\eta\backslash x^{(i)})) \\ &= \sum_{x^{(i)}\in\eta}\int_{\mathbb{R}^d} a_\epsilon(\epsilon^{-1}x-y)u((\eta\cup\epsilon y^{(j)})\backslash x^{(i)})dy \\ &+ \sum_{y^{(j)}\in\eta}\sum_{x^{(i)}\in\eta\backslash y^{(j)}} a_\epsilon(\epsilon^{-1}x-\epsilon^{-1}y)u(\eta\backslash x^{(i)}) \\ &= \sum_{x^{(i)}\in\eta}\int_{\mathbb{R}^d} \epsilon^d a(x-\epsilon y)u((\eta\cup\epsilon y^{(j)})\backslash x^{(i)})dy \\ &+ \sum_{y^{(j)}\in\eta}\sum_{x^{(i)}\in\eta\backslash y^{(j)}} \epsilon^d a(x-y)u(\eta\backslash x^{(i)}) = \big((A+\epsilon^d B)u\big)(\eta),\end{aligned}$$

where

$$\begin{aligned}(Au)(\eta) &= \sum_{x^{(i)}\in\eta}\int_{\mathbb{R}^d} a(x-y)u(\eta\cup y^{(j)}\backslash x^{(i)})dy; \\ (Bu)(\eta) &= \sum_{y^{(j)}\in\eta}\sum_{x^{(i)}\in\eta\backslash y^{(j)}} a(x-y)u(\eta\backslash x^{(i)}).\end{aligned}$$

Thus, applying perturbation expansion in the limit $\epsilon^d \to 0$, one obtains:

$$(Av)(\eta) = \mathbb{1}_{\eta=\{x^{(m)}\}}\delta_{im}\int_{\mathbb{R}^d} a(x-y)q_j(y,t)dy. \tag{73}$$

In order to obtain equations for corrections w, one uses

$$\frac{\partial}{\partial t}w(t,\eta) = (Aw+Bv+Dv+C(v,w)+C(w,v))(\eta),$$

where $C = 0$, $D = 0$,

$$
\begin{aligned}
(Aw)(\eta) &= \sum_{x^{(i)}\in\eta} \int_{\mathbb{R}^d} a(x-y) w((\eta \cup y^{(j)})\backslash x^{(i)}) dy \\
&= \mathbb{1}_{w=\{x^{(m)}\}} \delta_{im} \int_{\mathbb{R}^d} a(x-y) p_j(y) dy \\
&+ \mathbb{1}_{w=\{x_1^{(m)}, x_2^{(n)}\}} \Big(\\
&\quad +\delta_{ni}\delta_{mi} \int_{\mathbb{R}^d} dy [g_{ij}(x_1, y) a(x_2 - y) + g_{ji}(y, x_2) a(x_1 - y)] \\
&\quad +\delta_{mi}\delta_{n\neq i} \int_{\mathbb{R}^d} dy g_{jn}(y, x_2) a(x_1 - y) \\
&\quad +\delta_{m\neq i}\delta_{ni} \int_{\mathbb{R}^d} dy g_{mj}(x_1, y) a(x_2 - y) \Big);
\end{aligned}
$$

$$
\begin{aligned}
(Bv)(\eta) &= \sum_{y^{(j)}\in\eta} \sum_{x^{(i)}\in\eta\backslash y^{(j)}} a(x-y) v(\eta\backslash x^{(i)}) \\
&= \mathbb{1}_{\eta=\{x_1^{(m)}, x_2^{(n)}\}} \Big(\\
&\quad +\delta_{mj}\delta_{ni} a(x_1 - x_2) q_j(x_1) \\
&\quad +\delta_{mi}\delta_{nj} a(x_1 - x_2) q_j(x_2).
\end{aligned}
$$

As a result, one obtains

$$
\begin{aligned}
H^{\mathrm{BT}}_{q_m}(x) &= \delta_{mi} \int_{\mathbb{R}^d} a(x-y) q_j(y) dy; \\
H^{\mathrm{BT}}_{p_m}(x) &= \delta_{mi} \int_{\mathbb{R}^d} a(x-y) p_j(y) dy; \\
H^{\mathrm{BT}}_{g_{mn}}(x_1, x_2) &= +\delta_{ni}\delta_{mi} \int_{\mathbb{R}^d} dy [g_{ij}(x_1, y) a(x_2 - y) + g_{ji}(y, x_2) a(x_1 - y)] \\
&\quad +\delta_{mi}\delta_{n\neq i} \int_{\mathbb{R}^d} dy g_{jn}(y, x_2) a(x_1 - y) \\
&\quad +\delta_{m\neq i}\delta_{ni} \int_{\mathbb{R}^d} dy g_{mj}(x_1, y) a(x_2 - y) \Big) \\
&\quad +\delta_{mj}\delta_{ni} a(x_1 - x_2) q_j(x_1) \\
&\quad +\delta_{mi}\delta_{nj} a(x_1 - x_2) q_j(x_2).
\end{aligned}
\tag{74}
$$

In the translationally invariant case, using the notation $A = \int_{\mathbb{R}^d} a(x) dx$, one obtains

$$
\begin{aligned}
H^{\mathrm{BT}}_{q_m} &= \delta_{mi} A q_j; \\
H^{\mathrm{BT}}_{p_m} &= \delta_{mi} A p_j; \\
H^{\mathrm{BT}}_{g_{mn}}(x) &= +\delta_{ni}\delta_{mi} 2 \int_{\mathbb{R}^d} dy g_{ij}(y) a(x-y) \\
&\quad +\delta_{mi}\delta_{n\neq i} \int_{\mathbb{R}^d} dy g_{jn}(y) a(x-y) \\
&\quad +\delta_{m\neq i}\delta_{ni} \int_{\mathbb{R}^d} dy g_{mj}(y) a(x-y) \\
&\quad +\delta_{mj}\delta_{ni} a(x) q_j \\
&\quad +\delta_{mi}\delta_{nj} a(x) q_j.
\end{aligned}
\tag{75}
$$

Birth, $L = L_i^{\mathrm{B}}(a)$

Process definition: existing individuals of type i produce new individuals of type i with kernel a,

$$(L_i^{\mathrm{B}}(a)F)(\gamma) = \sum_{y\in\gamma_i}\int_{\mathbb{R}^d} a(x-y)(L_i^{x^+}F)(\gamma)dx. \tag{76}$$

This process is a special case of the process (71). Results for this case are given by expressions (75) by using $j = i$. Thus, in the translationally invariant case:

$$\begin{aligned}
H^{\mathrm{B}}_{q_m} &= \delta_{mi}Aq_i;\\
H^{\mathrm{B}}_{p_m} &= \delta_{mi}Ap_i;\\
H^{\mathrm{B}}_{g_{mn}}(x) &= +\delta_{ni}\delta_{mi}\left(2\int_{\mathbb{R}^d} dy g_{ii}(y)a(x-y) + 2a(x)q_i\right)\\
&\quad +\delta_{mi}\delta_{n\neq i}\int_{\mathbb{R}^d} dy g_{in}(y)a(x-y)\\
&\quad +\delta_{m\neq i}\delta_{ni}\int_{\mathbb{R}^d} dy g_{mi}(y)a(x-y).
\end{aligned} \tag{77}$$

Birth to another type by facilitation, $L = L_{kji}^{\mathrm{BTF}}(a,b)$

Process definition: type j individuals produce offspring of type k with kernel b, mediated by (but not affecting) type i ($i \neq j$) individuals with kernel a. In order to consider a general case, index k can be equal to j or i. The process is defined by the following Markov operator $L_{kji}^{\mathrm{BTF}}(a,b)$,

$$(L_{kji}^{\mathrm{BTF}}(a,b)F)(\gamma) = \sum_{y\in\gamma_j}\sum_{z\in\gamma_i}\int_{\mathbb{R}^d} dx a(z-y)b(x-y)(L_k^{x^+}F)(\gamma). \tag{78}$$

Using $F = KG$, the expression for $\widehat{L}G$ is the following,

$$\begin{aligned}
(\widehat{L}G)(\eta) &= (K^{-1}L_{kji}^{\mathrm{BTF}}(a,b)KG)(\eta)\\
&= \sum_{\xi\subset\eta}(-1)^{|\eta\setminus\xi|}\sum_{y^{(j)},z^{(i)}\in\xi}\int_{\mathbb{R}^d} dx a(z-y)b(x-y)(KD_x^{(k)}G)(\xi).
\end{aligned}$$

Using the change of variables $\xi \to \xi\cup y^{(j)}$, and then $\xi \to \xi \cup z^{(i)}$, one obtains:

$$\begin{aligned}
(\widehat{L}G)(\eta) &= \sum_{y^{(j)}\in\eta}\sum_{\xi\subset\eta\setminus y^{(j)}}(-1)^{|(\eta\setminus y^{(j)})\setminus\xi|}\sum_{z^{(i)}\in\xi}\int_{\mathbb{R}^d} dx a(z-y)b(x-y)(D_y^{(j)}KD_x^{(k)}G)(\xi)\\
&= \sum_{y^{(j)},z^{(i)}\in\eta}\sum_{\xi\subset\eta\setminus y^{(j)}\setminus z^{(i)}}(-1)^{|(\eta\setminus y^{(j)}\setminus z^{(i)})\setminus\xi|}\int_{\mathbb{R}^d} dx a(z-y)b(x-y)(D_z^{(i)}D_y^{(j)}KD_x^{(k)}G)(\xi).
\end{aligned}$$

From (41) it follows:

$$\begin{aligned}
(D_z^{(i)}D_y^{(j)}KD_x^{(k)}G)(\xi) &= (D_z^{(i)}\left[KD_x^{(k)} + KD_y^{(j)}D_x^{(k)}\right]G)(\xi)\\
&= (\left[KD_x^{(k)} + KD_z^{(i)}D_x^{(k)} + KD_y^{(j)}D_x^{(k)} + KD_z^{(i)}D_y^{(j)}D_x^{(k)}\right]G)(\xi).
\end{aligned}$$

Applying the inverse K-transform, one obtains the expression for $\widehat{L}$,

$$\begin{aligned}(\widehat{L}G)(\eta) &= \sum_{y^{(j)},z^{(i)}\in\eta} \int_{\mathbb{R}^d} dx a(z-y)b(x-y)(\Big[D_x^{(k)} + D_z^{(i)}D_x^{(k)} + D_y^{(j)}D_x^{(k)} \\ &\quad + D_z^{(i)}D_y^{(j)}D_x^{(k)}\Big] G)(\eta\backslash y^{(j)}\backslash z^{(i)}).\end{aligned}$$

Using $\widehat{L}G$ and changing variables $\eta \to \eta\backslash x^{(k)}$ one can derive $L^{\triangle}$ as shown below:

$$\begin{aligned}\int_{\Gamma_0}(\widehat{L}G)(\eta)k(\eta)d\lambda(\eta) &= \int_{\Gamma_0}\sum_{y^{(j)},z^{(i)}\in\eta}\int_{\mathbb{R}^d} dxa(z-y)b(x-y)\left[G(\eta\backslash y^{(j)}\backslash z^{(i)}\cup x^{(k)})+\right. \\ &\quad \left.+G(\eta\backslash y^{(j)}\cup x^{(k)}) + G(\eta\backslash z^{(i)}\cup x^{(k)}) + G(\eta\cup x^{(k)})\right]k(\eta)d\lambda(\eta). \\ &= \int_{\Gamma_0} d\lambda(\eta)\sum_{y^{(j)},z^{(i)}\in\eta}\sum_{x^{(k)}\in\eta\backslash y^{(j)}\backslash z^{(i)}} a(z-y)b(x-y)\left[G(\eta\backslash y^{(j)}\backslash z^{(i)})+\right. \\ &\quad \left.+G(\eta\backslash y^{(j)}) + G(\eta\backslash z^{(i)}) + G(\eta)\right]k(\eta\backslash x^{(k)}).\end{aligned}$$

In the first three terms, using the change of variables $\eta \to \eta\cup w$, where w denotes $y^{(j)}$ or $z^{(i)}$, the expression becomes:

$$\begin{aligned}\int_{\Gamma_0}(\widehat{L}G)(\eta)k(\eta)d\lambda(\eta) &= \int_{\Gamma_0} d\lambda(\eta)\Bigg[\int_{\mathbb{R}^d} dy\int_{\mathbb{R}^d} dz \sum_{x^{(k)}\in\eta} G(\eta)k((\eta\backslash x^{(k)})\cup y^{(j)}\cup z^{(i)})+ \\ &\quad + \int_{\mathbb{R}^d} dy \sum_{x^{(k)}\in\eta}\sum_{z^{(i)}\in\eta\backslash x^{(k)}} G(\eta)k((\eta\backslash x^{(k)})\cup y^{(j)}) \\ &\quad + \int_{\mathbb{R}^d} dz \sum_{x^{(k)}\in\eta}\sum_{y^{(j)}\in\eta\backslash x^{(k)}} G(\eta)k((\eta\backslash x^{(k)})\cup z^{(i)}) \\ &\quad + \sum_{x^{(k)}\in\eta}\sum_{y^{(j)},z^{(i)}\in\eta\backslash x^{(k)}} G(\eta)k(\eta\backslash x^{(k)})\Bigg] a(z-y)b(x-y) \\ &= \int_{\Gamma_0} G(\eta)(L^{\triangle}k)(\eta)d\lambda(\eta).\end{aligned}$$

Thus, one obtains $L^{\triangle}$,

$$\begin{aligned}(L^{\triangle}k)(\eta) &= \sum_{x^{(k)}\in\eta}\Bigg[\int_{\mathbb{R}^d} dy\int_{\mathbb{R}^d} dz\; k((\eta\backslash x^{(k)})\cup y^{(j)}\cup z^{(i)})+ \\ &\quad + \int_{\mathbb{R}^d} dy \sum_{z^{(i)}\in\eta\backslash x^{(k)}} k((\eta\backslash x^{(k)})\cup y^{(j)}) \\ &\quad + \int_{\mathbb{R}^d} dz \sum_{y^{(j)}\in\eta\backslash x^{(k)}} k((\eta\backslash x^{(k)})\cup z^{(i)}) \\ &\quad + \sum_{y^{(j)},z^{(i)}\in\eta\backslash x^{(k)}} k(\eta\backslash x^{(k)})\Bigg] a(z-y)b(x-y).\end{aligned} \tag{79}$$

In order to find $Q^\Delta = L^\Delta + M^\Delta$, one substitutes $\widehat{L}$ into the following expression,

$$\int_{\Gamma_0}\left(\widehat{L}e_\lambda(\theta)\right)(\eta)(\exp^* u)(\eta)d\lambda(\eta) = \int_{\Gamma_0}\sum_{y^{(j)},z^{(i)}\in\eta}\int_{\mathbb{R}^d} dxa(z-y)b(x-y)\Big(\Big[D_x^{(k)} + D_z^{(i)}D_x^{(k)}$$
$$+D_y^{(j)}D_x^{(k)} + D_z^{(i)}D_y^{(j)}D_x^{(k)}\Big]e_\lambda(\theta)\Big)(\eta\backslash y^{(j)}\backslash z^{(i)})(\exp^* u)(\eta)d\lambda(\eta).$$

Then, one transforms this expression so that it contains $e_\lambda(\theta,\eta)$ explicitly, and then transforms all summations to integrals over all space $\mathbb{R}^d$, expressing the resulting formula in terms of D_x, D_y. As a result, one obtains the following expression:

$$\int_{\Gamma_0}\int_{(\mathbb{R}^d)^3} dxdydz\; a(z-y)b(x-y)\left[\theta(x^{(k)}) + \theta(z^{(i)})\theta(x^{(k)}) + \theta(y^{(j)})\theta(x^{(k)})\right.$$
$$\left.+\theta(z^{(i)})\theta(y^{(j)})\theta(x^{(k)})\right]e_\lambda(\theta,\eta)D_y^{(j)}D_z^{(i)}(\exp^* u)(\eta)d\lambda(\eta).$$

In order to find M^Δ, one substitutes $D_y^{(j)}D_z^{(i)}(\exp^* u)(\eta) \longrightarrow (D_y^{(j)}u * D_z^{(i)}u)(\eta)$. Returning to summations, one obtains the expression in the following form:

$$\int_{\Gamma_0} e_\lambda(\theta,\eta)\sum_{x^{(k)}\in\eta}\Bigg[\int_{(\mathbb{R}^d)^2} dydz\; a(z-y)b(x-y)(D_y^{(j)}u * D_z^{(i)}u)(\eta\backslash x^{(k)})$$
$$+\int_{\mathbb{R}^d} dy\sum_{z^{(i)}\in\eta\backslash x^{(k)}} a(z-y)b(x-y)(D_y^{(j)}u * D_z^{(i)}u)(\eta\backslash x^{(k)}\backslash z^{(i)})$$
$$+\int_{\mathbb{R}^d} dz\sum_{y^{(j)}\in\eta\backslash x^{(k)}} a(z-y)b(x-y)(D_y^{(j)}u * D_z^{(i)}u)(\eta\backslash x^{(k)}\backslash y^{(j)})$$
$$+\sum_{y^{(j)},z^{(i)}\in\eta\backslash x^{(k)}} a(z-y)b(x-y)(D_y^{(j)}u * D_z^{(i)}u)(\eta\backslash x^{(k)}\backslash y^{(j)}\backslash z^{(i)})\Bigg]d\lambda(\eta) =$$
$$= \int_{\Gamma_0} e_\lambda(\theta,\eta)\left(M^\Delta u\right)(\eta)d\lambda(\eta).$$

The expression for M^Δ can be rewritten as follows,

$$(M^\Delta u)(\eta) = \sum_{x^{(k)}\in\eta}\int_{(\mathbb{R}^d)^2} dydz\; a(z-y)b(x-y)(u(\cdot\cup y^{(j)}) * u(\cdot\cup z^{(i)}))(\eta\backslash x^{(k)})$$
$$+\sum_{x^{(k)}\in\eta}\int_{\mathbb{R}^d} dy\sum_{z^{(i)}\in\eta\backslash x^{(k)}} a(z-y)b(x-y)(u(\cdot\cup y^{(j)}) * u(\cdot\cup z^{(i)}))(\eta\backslash x^{(k)}\backslash z^{(i)})$$
$$+\sum_{x^{(k)}\in\eta}\int_{\mathbb{R}^d} dz\sum_{y^{(j)}\in\eta\backslash x^{(k)}} a(z-y)b(x-y)(u(\cdot\cup y^{(j)}) * u(\cdot\cup z^{(i)}))(\eta\backslash x^{(k)}\backslash y^{(j)})$$
$$+\sum_{x^{(k)}\in\eta}\sum_{y^{(j)},z^{(i)}\in\eta\backslash x^{(k)}} a(z-y)b(x-y)(u(\cdot\cup y^{(j)}) * u(\cdot\cup z^{(i)}))(\eta\backslash x^{(k)}\backslash y^{(j)}\backslash z^{(i)}).$$

In order to find $L^{\Delta}_{\epsilon,\mathrm{ren}}$ and $M^{\Delta}_{\epsilon,\mathrm{ren}}$ one should obtain L^{Δ}_{ϵ} and M^{Δ}_{ϵ} from L^{Δ} and M^{Δ} by replacing potentials a and b by a_ϵ and b_ϵ. Then, following the definition, one obtains:

$$
\begin{aligned}
(S_{\epsilon^{-1}} L^{\Delta}_{\epsilon} S_\epsilon k)(\eta) &= S_{\epsilon^{-1}} \sum_{x^{(k)}\in\eta} \int_{\mathbb{R}^d} dy \int_{\mathbb{R}^d} dz\ k(\epsilon((\eta\backslash x^{(k)}) \cup y^{(j)} \cup z^{(i)}))a_\epsilon(z-y)b_\epsilon(x-y)\\
&\quad + S_{\epsilon^{-1}} \sum_{x^{(k)}\in\eta} \int_{\mathbb{R}^d} dy \sum_{z^{(i)}\in\eta\backslash x^{(k)}} k(\epsilon((\eta\backslash x^{(k)}) \cup y^{(j)}))a_\epsilon(z-y)b_\epsilon(x-y)\\
&\quad + S_{\epsilon^{-1}} \sum_{x^{(k)}\in\eta} \int_{\mathbb{R}^d} dz \sum_{y^{(j)}\in\eta\backslash x^{(k)}} k(\epsilon((\eta\backslash x^{(k)}) \cup z^{(i)}))a_\epsilon(z-y)b_\epsilon(x-y)\\
&\quad + S_{\epsilon^{-1}} \sum_{x^{(k)}\in\eta} \sum_{y^{(j)},z^{(i)}\in\eta\backslash x^{(k)}} k(\epsilon(\eta\backslash x^{(k)}))a_\epsilon(z-y)b_\epsilon(x-y)\\
&= \int_{\mathbb{R}^d} dy \int_{\mathbb{R}^d} dz \sum_{x^{(k)}\in\eta} k((\eta\backslash x^{(k)}) \cup \epsilon y^{(j)} \cup \epsilon z^{(i)})\epsilon^d a(\epsilon z-\epsilon y)\epsilon^d b(x-\epsilon y)\\
&\quad + \int_{\mathbb{R}^d} dy \sum_{x^{(k)}\in\eta} \sum_{z^{(i)}\in\eta\backslash x^{(k)}} k((\eta\backslash x^{(k)}) \cup \epsilon y^{(j)})\epsilon^d a(z-\epsilon y)\epsilon^d b(x-\epsilon y)\\
&\quad + \int_{\mathbb{R}^d} dz \sum_{x^{(k)}\in\eta} \sum_{y^{(j)}\in\eta\backslash x^{(k)}} k((\eta\backslash x^{(k)}) \cup \epsilon z^{(i)})\epsilon^d a(\epsilon z-y)\epsilon^d b(x-y)\\
&\quad + \sum_{x^{(k)}\in\eta} \sum_{y^{(j)},z^{(i)}\in\eta\backslash x^{(k)}} k(\eta\backslash x^{(k)})\epsilon^d a(z-y)\epsilon^d b(x-y)\\
&= \int_{\mathbb{R}^d} dy \int_{\mathbb{R}^d} dz \sum_{x^{(k)}\in\eta} k((\eta\backslash x^{(k)}) \cup y^{(j)} \cup z^{(i)})a(z-y)b(x-y)\\
&\quad + \epsilon^d \int_{\mathbb{R}^d} dy \sum_{x^{(k)}\in\eta} \sum_{z^{(i)}\in\eta\backslash x^{(k)}} k((\eta\backslash x^{(k)}) \cup y^{(j)})a(z-y)b(x-y)\\
&\quad + \epsilon^d \int_{\mathbb{R}^d} dz \sum_{x^{(k)}\in\eta} \sum_{y^{(j)}\in\eta\backslash x^{(k)}} k((\eta\backslash x^{(k)}) \cup z^{(i)})a(z-y)b(x-y)\\
&\quad + \epsilon^{2d} \sum_{x^{(k)}\in\eta} \sum_{y^{(j)},z^{(i)}\in\eta\backslash x^{(k)}} k(\eta\backslash x^{(k)})a(z-y)b(x-y)\\
&= ((A+\epsilon^d B + o(\epsilon^d))k)(\eta),
\end{aligned}
$$

where

$$
\begin{aligned}
(Ak)(\eta) &= \int_{\mathbb{R}^d} dy \int_{\mathbb{R}^d} dz \sum_{x^{(k)}\in\eta} k((\eta\backslash x^{(k)}) \cup y^{(j)} \cup z^{(i)})a(z-y)b(x-y);\\
(Bk)(\eta) &= \int_{\mathbb{R}^d} dy \sum_{x^{(k)}\in\eta} \sum_{z^{(i)}\in\eta\backslash x^{(k)}} k((\eta\backslash x^{(k)}) \cup y^{(j)})a(z-y)b(x-y)\\
&\quad + \int_{\mathbb{R}^d} dz \sum_{x^{(k)}\in\eta} \sum_{y^{(j)}\in\eta\backslash x^{(k)}} k((\eta\backslash x^{(k)}) \cup z^{(i)})a(z-y)b(x-y).
\end{aligned}
$$

Then, $M^{\Delta}_{\epsilon,\mathrm{ren}}$ is found in the same way:

$$
\begin{aligned}
(S_{\epsilon^{-1}} M^{\Delta}_{\epsilon} S_{\epsilon} u)(\eta) = & \int_{(\mathbb{R}^d)^2} dydz \sum_{x^{(k)}\in\eta} a(z-y)b(x-y)(u(\cdot\cup y^{(j)}) * u(\cdot\cup z^{(i)}))(\eta\backslash x^{(k)}) \\
& + \epsilon^d \int_{\mathbb{R}^d} dy \sum_{x^{(k)}\in\eta} \sum_{z^{(i)}\in\eta\backslash x^{(k)}} a(z-y)b(x-y)(u(\cdot\cup y^{(j)}) * u(\cdot\cup z^{(i)}))(\eta\backslash x^{(k)}\backslash z^{(i)}) \\
& + \epsilon^d \int_{\mathbb{R}^d} dz \sum_{x^{(k)}\in\eta} \sum_{y^{(j)}\in\eta\backslash x^{(k)}} a(z-y)b(x-y)(u(\cdot\cup y^{(j)}) * u(\cdot\cup z^{(i)}))(\eta\backslash x^{(k)}\backslash y^{(j)}) \\
& + \epsilon^{2d} \sum_{x^{(k)}\in\eta} \sum_{y^{(j)},z^{(i)}\in\eta\backslash x^{(k)}} a(z-y)b(x-y)(u(\cdot\cup y^{(j)}) * u(\cdot\cup z^{(i)}))(\eta\backslash x^{(k)}\backslash y^{(j)}\backslash z^{(i)}) \\
& = ((C+\epsilon^d D + o(\epsilon^d))u)(\eta).
\end{aligned}
$$

Where

$$
\begin{aligned}
(Cu)(\eta) &= \int_{(\mathbb{R}^d)^2} dydz \sum_{x^{(k)}\in\eta} a(z-y)b(x-y)(u(\cdot\cup y^{(j)}) * u(\cdot\cup z^{(i)}))(\eta\backslash x^{(k)}); \\
(Du)(\eta) &= \int_{\mathbb{R}^d} dy \sum_{x^{(k)}\in\eta} \sum_{z^{(i)}\in\eta\backslash x^{(k)}} a(z-y)b(x-y)(u(\cdot\cup y^{(j)}) * u(\cdot\cup z^{(i)}))(\eta\backslash x^{(k)}\backslash z^{(i)}) \\
&\quad + \int_{\mathbb{R}^d} dz \sum_{x^{(k)}\in\eta} \sum_{y^{(j)}\in\eta\backslash x^{(k)}} a(z-y)b(x-y)(u(\cdot\cup y^{(j)}) * u(\cdot\cup z^{(i)}))(\eta\backslash x^{(k)}\backslash y^{(j)}).
\end{aligned}
$$

Thus, applying perturbation expansion in the limit $\epsilon^d \to 0$, one obtains:

$$
(Av+Cv)(\eta) = \mathbb{1}_{\eta=\{x^{(m)}\}} \delta_{km} \int_{(\mathbb{R}^d)^2} dydz a(z-y)b(x-y) q_j(t,y) q_i(t,z).
$$

In order to obtain equations for corrections w, one uses

$$
\frac{\partial}{\partial t} w(t,\eta) = (Aw + Bv + Dv + C(v,w) + C(w,v))(\eta),
$$

where

$$
\begin{aligned}
(Aw)(\eta) &= \int_{\mathbb{R}^d} dy \int_{\mathbb{R}^d} dz \sum_{x^{(k)}\in\eta} w((\eta\backslash x^{(k)}) \cup y^{(j)} \cup z^{(i)}) a(z-y)b(x-y) \\
&= \mathbb{1}_{\eta=\{x^{(m)}\}} \delta_{mk} \int_{(\mathbb{R}^d)^2} dydz\ g_{ji}(y,z) a(z-y)b(x-y);
\end{aligned}
$$

$$
\begin{aligned}
(Bv)(\eta) &= \int_{\mathbb{R}^d} dy \sum_{x^{(k)}\in\eta} \sum_{z^{(i)}\in\eta\backslash x^{(k)}} v((\eta\backslash x^{(k)}) \cup y^{(j)}) a(z-y)b(x-y) \\
&\quad + \int_{\mathbb{R}^d} dz \sum_{x^{(k)}\in\eta} \sum_{y^{(j)}\in\eta\backslash x^{(k)}} v((\eta\backslash x^{(k)}) \cup z^{(i)}) a(z-y)b(x-y) \\
&= 0,
\end{aligned}
$$

where the last equality is due to the requirement by two summations to have $|\eta| \geq 2$, but simultaneously $v(\eta) = 0$ if $|\eta| \neq 1$;

$$\begin{aligned}
(Dv)(\eta) &= \int_{\mathbb{R}^d} dy \sum_{x^{(k)}\in\eta} \sum_{z^{(i)}\in\eta\setminus x^{(k)}} a(z-y)b(x-y)(v(\cdot\cup y^{(j)}) * v(\cdot\cup z^{(i)}))(\eta\setminus x^{(k)}\setminus z^{(i)}) \\
&+ \int_{\mathbb{R}^d} dz \sum_{x^{(k)}\in\eta} \sum_{y^{(j)}\in\eta\setminus x^{(k)}} a(z-y)b(x-y)(v(\cdot\cup y^{(j)}) * v(\cdot\cup z^{(i)}))(\eta\setminus x^{(k)}\setminus y^{(j)}) \\
&= \int_{\mathbb{R}^d} dy \sum_{x^{(k)}\in\eta} \sum_{z^{(i)}\in\eta\setminus x^{(k)}} a(z-y)b(x-y)v(y^{(j)})v(\eta\setminus x^{(k)}) \\
&+ \int_{\mathbb{R}^d} dz \sum_{x^{(k)}\in\eta} \sum_{y^{(j)}\in\eta\setminus x^{(k)}} a(z-y)b(x-y)v(\eta\setminus x^{(k)})v(z^{(i)}) \\
&= \mathbb{1}_{\eta=\{x_1^{(m)},x_2^{(n)}\}} \Big(\\
&+\delta_{mi}\delta_{nk} \int_{\mathbb{R}^d} dy q_j(y)a(x_1-y)b(x_2-y)q_i(x_1) \\
&+\delta_{mk}\delta_{ni} \int_{\mathbb{R}^d} dy q_j(y)a(x_2-y)b(x_1-y)q_i(x_2)\Big) \\
&+\mathbb{1}_{\eta=\{x_1^{(m)},x_2^{(n)}\}} \Big(\\
&+\delta_{mj}\delta_{nk} b(x_1-x_2) \int_{\mathbb{R}^d} dz q_i(z)a(z-x_1)q_j(x_1) \\
&+\delta_{mk}\delta_{nj} b(x_1-x_2) \int_{\mathbb{R}^d} dz q_i(z)a(z-x_2)q_j(x_2)\Big);
\end{aligned}$$

$$\begin{aligned}
C(v,w)(\eta) &= \int_{(\mathbb{R}^d)^2} dydz \sum_{x^{(k)}\in\eta} a(z-y)b(x-y)(v(\cdot\cup y^{(j)}) * w(\cdot\cup z^{(i)}))(\eta\setminus x^{(k)}) \\
&= \int_{(\mathbb{R}^d)^2} dydz \sum_{x^{(k)}\in\eta} a(z-y)b(x-y)v(y^{(j)})w((\eta\setminus x^{(k)})\cup z^{(i)}) \\
&= \mathbb{1}_{\eta=\{x^{(m)}\}}\delta_{mk} \int_{(\mathbb{R}^d)^2} dydz\ a(z-y)b(x-y)q_j(y)p_i(z) \\
&+\mathbb{1}_{\eta=\{x_1^{(m)},x_2^{(n)}\}} \times \Big(\\
&+\delta_{mk}\delta_{nk} \int_{(\mathbb{R}^d)^2} dydz a(z-y)q_j(y)\left[b(x_1-y)g_{ik}(z,x_2) + b(x_2-y)g_{ki}(x_1,z)\right] \\
&+\delta_{m\neq k}\delta_{nk} \int_{(\mathbb{R}^d)^2} dydz\ a(z-y)b(x_2-y)q_j(y)g_{mi}(x_1,z) \\
&+\delta_{mk}\delta_{n\neq k} \int_{(\mathbb{R}^d)^2} dydz\ a(z-y)b(x_1-y)q_j(y)g_{in}(z,x_2)\Big);
\end{aligned}$$

$$
\begin{aligned}
C(w,v)(\eta) &= \int_{(\mathbb{R}^d)^2} dydz \sum_{x^{(k)}\in\eta} a(z-y)b(x-y)(w(\cdot\cup y^{(j)}) * v(\cdot\cup z^{(i)}))(\eta\backslash x^{(k)}) \\
&= \int_{(\mathbb{R}^d)^2} dydz \sum_{x^{(k)}\in\eta} a(z-y)b(x-y)w((\eta\backslash x^{(k)})\cup y^{(j)})v(z^{(i)}) \\
&= \mathbb{1}_{\eta=\{x^{(m)}\}}\delta_{mk}\int_{(\mathbb{R}^d)^2} dydz\ a(z-y)b(x-y)p_j(y)q_i(z) \\
&\quad +\mathbb{1}_{\eta=\{x_1^{(m)},x_2^{(n)}\}}\times\Big(\\
&\quad +\delta_{mk}\delta_{nk}\int_{(\mathbb{R}^d)^2} dydz\ a(z-y)q_i(z)[b(x_1-y)g_{jk}(y,x_2)+b(x_2-y)g_{kj}(x_1,y)] \\
&\quad +\delta_{m\neq k}\delta_{nk}\int_{(\mathbb{R}^d)^2} dydz\ a(z-y)b(x_2-y)g_{mj}(x_1,y)q_i(z) \\
&\quad +\delta_{mk}\delta_{n\neq k}\int_{(\mathbb{R}^d)^2} dydz\ a(z-y)b(x_1-y)g_{jn}(y,x_2)q_i(z)\Big).
\end{aligned}
$$

As a result, one obtains:

$$
\begin{aligned}
H^{\mathrm{BTF}}_{q_m}(x) &= \delta_{mk}\int_{(\mathbb{R}^d)^2} dydza(z-y)b(x-y)q_j(y)q_i(z); \\
H^{\mathrm{BTF}}_{p_m}(x) &= \delta_{mk}\int_{(\mathbb{R}^d)^2} dydz\ g_{ji}(y,z)a(z-y)b(x-y) \\
&\quad +\delta_{mk}\int_{(\mathbb{R}^d)^2} dydz\ a(z-y)b(x-y)[q_j(y)p_i(z)+p_j(y)q_i(z)]; \\
H^{\mathrm{BTF}}_{g_{mn}}(x_1,x_2) &= \delta_{mk}\delta_{nk}\Big(\int_{(\mathbb{R}^d)^2} dydz\ a(z-y)q_i(z)[b(x_1-y)g_{jk}(y,x_2)+b(x_2-y)g_{kj}(x_1,y)] \\
&\qquad +\int_{(\mathbb{R}^d)^2} dydza(z-y)q_j(y)\left[b(x_1-y)g_{ik}(z,x_2)+b(x_2-y)g_{ki}(x_1,z)\right]\Big) \\
&\quad +\delta_{mi}\delta_{nk}\int_{\mathbb{R}^d} dyq_j(y)a(x_1-y)b(x_2-y)q_i(x_1) \\
&\quad +\delta_{mk}\delta_{ni}\int_{\mathbb{R}^d} dyq_j(y)a(x_2-y)b(x_1-y)q_i(x_2) \\
&\quad +\delta_{mj}\delta_{nk}b(x_1-x_2)q_j(x_1)\int_{\mathbb{R}^d} dzq_i(z)a(z-x_1) \\
&\quad +\delta_{mk}\delta_{nj}b(x_1-x_2)q_j(x_2)\int_{\mathbb{R}^d} dzq_i(z)a(z-x_2) \\
&\quad +\delta_{m\neq k}\delta_{nk}\Big(\int_{(\mathbb{R}^d)^2} dydz\ a(z-y)b(x_2-y)q_j(y)g_{mi}(x_1,z) \\
&\qquad +\int_{(\mathbb{R}^d)^2} dydz\ a(z-y)b(x_2-y)g_{mj}(x_1,y)q_i(z)\Big) \\
&\quad +\delta_{mk}\delta_{n\neq k}\Big(\int_{(\mathbb{R}^d)^2} dydz\ a(z-y)b(x_1-y)q_j(y)g_{in}(z,x_2) \\
&\qquad +\int_{(\mathbb{R}^d)^2} dydz\ a(z-y)b(x_1-y)g_{jn}(y,x_2)q_i(z)\Big).
\end{aligned}
\tag{80}
$$

In translationally invariant case, using the notation $A = \int_{\mathbb{R}^d} a(x)dx$, $B = \int_{\mathbb{R}^d} b(x)dx$, the expressions become:

$$
\begin{aligned}
H^{\mathrm{BTF}}_{q_m} &= \delta_{mk} AB q_j q_i; \\
H^{\mathrm{BTF}}_{p_m} &= \delta_{mk} \left(AB(q_j p_i + p_j q_i) + B \int_{\mathbb{R}^d} dz\ g_{ji}(z) a(z) \right); \\
H^{\mathrm{BTF}}_{g_{nm}}(x) &= \delta_{mk}\delta_{nk} \left(2q_i A \int_{\mathbb{R}^d} dy b(x-y) g_{jk}(y) + 2q_j \int_{(\mathbb{R}^d)^2} dydz a(x-z) b(z-y) g_{ik}(y) \right) \\
&\quad + (\delta_{mi}\delta_{nk} + \delta_{mk}\delta_{ni})\ q_i q_j \int_{\mathbb{R}^d} dy a(x-y) b(y) \\
&\quad + (\delta_{mj}\delta_{nk} + \delta_{mk}\delta_{nj})\ q_i q_j A b(x) \\
&\quad + \delta_{m\neq k}\delta_{nk}\ \left(q_i A \int_{\mathbb{R}^d} dy b(x-y) g_{mj}(y) + q_j \int_{(\mathbb{R}^d)^2} dydz\ a(x-z) b(z-y) g_{mi}(y) \right) \\
&\quad + \delta_{mk}\delta_{n\neq k}\ \left(q_i A \int_{\mathbb{R}^d} dy b(x-y) g_{jn}(y) + q_j \int_{(\mathbb{R}^d)^2} dydz\ a(x-z) b(z-y) g_{in}(y) \right).
\end{aligned}
\tag{81}
$$

Birth by facilitation, $L = L^{\mathrm{BF}}_{ji}(a,b)$

Process definition: type j individuals produce offspring of type j with kernel b, mediated by (but not affecting) type i $(i \neq j)$ individuals with kernel a. The process is defined by the following Markov operator $L^{\mathrm{BF}}_{ji}(a,b)$,

$$
(L^{\mathrm{BF}}_{ji}(a,b)F)(\gamma) = \sum_{y\in\gamma_j} \sum_{z\in\gamma_i} \int_{\mathbb{R}^d} a(z-y) b(x-y) (L^{x^+}_j F)(\gamma) dx. \tag{82}
$$

This process is a special case of the process (78). In the translationally invariant case, results can be obtained from equations (81) by making k equal to j:

$$
\begin{aligned}
H^{\mathrm{BF}}_{q_m} &= \delta_{mj} AB q_j q_i; \qquad (83) \\
H^{\mathrm{BF}}_{p_m} &= \delta_{mj} \left(AB(q_j p_i + p_j q_i) + B \int_{\mathbb{R}^d} dz\ g_{ji}(z) a(z) \right); \\
H^{\mathrm{BF}}_{g_{nm}}(x) &= \delta_{mj}\delta_{nj} \left(2q_i q_j A b(x) + 2q_i A \int_{\mathbb{R}^d} dy b(x-y) g_{jj}(y) \right. \\
&\qquad \left. + 2q_j \int_{(\mathbb{R}^d)^2} dydz a(x-z) b(z-y) g_{ij}(y) \right) \\
&\quad + (\delta_{mi}\delta_{nj} + \delta_{mj}\delta_{ni})\ q_i q_j \int_{\mathbb{R}^d} dy a(x-y) b(y) \\
&\quad + \delta_{m\neq j}\delta_{nj}\ \left(q_i A \int_{\mathbb{R}^d} dy b(x-y) g_{mj}(y) + q_j \int_{(\mathbb{R}^d)^2} dydz\ a(x-z) b(z-y) g_{mi}(y) \right) \\
&\quad + \delta_{mj}\delta_{n\neq j}\ \left(q_i A \int_{\mathbb{R}^d} dy b(x-y) g_{jn}(y) + q_j \int_{(\mathbb{R}^d)^2} dydz\ a(x-z) b(z-y) g_{in}(y) \right).
\end{aligned}
$$

1.3.2 Processes defined by a single death event by an operator $L_i^{x^-}$

Density independent death, $L = L_i^{\mathrm{D}}(r)$

Process definition: individuals of type i die with a rate r,

$$(L_i^{\mathrm{D}}(r)F)(\gamma) = r \sum_{x\in\gamma_i} (L_i^{x^-} F)(\gamma). \tag{84}$$

Using $F = KG$, the expression for $\widehat{L}G$ follows from its definition:

$$\begin{aligned}(\widehat{L}G)(\eta) &= (K^{-1}L_i^{\mathrm{D}}(r)KG)(\eta)\\ &= -r\sum_{\xi\subset\eta}(-1)^{|\eta\backslash\xi|}\sum_{x^{(i)}\in\xi}(KD_x^{(i)}G)(\xi\backslash x^{(i)}).\end{aligned}$$

Using the change of variables $\xi \to \xi \cup x^{(i)}$, one obtains

$$\begin{aligned}(\widehat{L}G)(\eta) &= -r\sum_{x^{(i)}\in\eta}\sum_{\xi\subset\eta\backslash x^{(i)}}(-1)^{|(\eta\backslash x^{(i)})\backslash\xi|}(KD_x^{(i)}G)(\xi)\\ &= -r\sum_{x^{(i)}\in\eta}(D_x^{(i)}G)(\eta\backslash x^{(i)})\\ &= -r\sum_{x^{(i)}\in\eta}G(\eta)\\ &= -r|\eta_i|G(\eta).\end{aligned}$$

L^Δ can be easily found,

$$\int_{\Gamma_0}(\widehat{L}G)(\eta)k(\eta)d\lambda(\eta) = -r|\eta_i|\int_{\Gamma_0}G(\eta)k(\eta)d\lambda(\eta);$$

$$(L^\Delta k)(\eta) = -r|\eta_i|k(\eta). \tag{85}$$

In order to find Q^Δ, one substitutes $\widehat{L}$ into the following expression,

$$\begin{aligned}\int_{\Gamma_0}\left(\widehat{L}e_\lambda(\theta)\right)(\eta)(\exp^* u)(\eta)d\lambda(\eta) &= -\int_{\Gamma_0} r\sum_{x^{(i)}\in\eta}e_\lambda(\theta,\eta)(\exp^* u)(\eta)d\lambda(\eta)\\ &= -\int_{\Gamma_0} r\int_{\mathbb{R}^d}dx e_\lambda(\theta,\eta)\theta(x^{(i)})D_x^{(i)}(\exp^* u)(\eta)d\lambda(\eta).\end{aligned}$$

Here, the terms with more than one operation D are absent here, therefore here $Q^\Delta = L^\Delta$. There is no spatial interaction, therefore $Q^\Delta_{\epsilon,\mathrm{ren}} = Q^\Delta$.

$$(Q^\Delta_{\epsilon,\mathrm{ren}}u)(\eta) = -r|\eta_i|u(\eta). \tag{86}$$

Thus, applying perturbation expansion in the limit $\epsilon^d \to 0$, one obtains:

$$\begin{aligned}H^{\mathrm{D}}_{q_m}(x) &= -\delta_{mi}rq_i(x);\\ H^{\mathrm{D}}_{p_m}(x) &= -\delta_{mi}rp_i(x);\\ H^{\mathrm{D}}_{g_{mn}}(x_1,x_2) &= -\delta_{mi}\delta_{ni}2rg_{ii}(x_1,x_2) - \delta_{m\neq i}\delta_{ni}rg_{mi}(x_1,x_2) - \delta_{mi}\delta_{n\neq i}rg_{in}(x_1,x_2).\end{aligned}$$

In the translationally invariant case:

$$\begin{aligned}
H^{\mathrm{D}}_{q_m} &= -\delta_{mi} r q_i;\\
H^{\mathrm{D}}_{p_m} &= -\delta_{mi} r p_i;\\
H^{\mathrm{D}}_{g_{mn}}(x) &= -\delta_{mi}\delta_{ni} 2 r g_{ii}(x) - \delta_{m\neq i}\delta_{ni} r g_{mi}(x) - \delta_{mi}\delta_{n\neq i} r g_{in}(x).
\end{aligned} \tag{87}$$

Death by external factor, $L = L^{\mathrm{DE}}_{ji}(a)$

Process definition: type i individuals induce death in type j individuals with kernel a. In the case $i = j$ this example describes “Death by competition”. The process is defined by the following Markov operator:

$$(L^{\mathrm{DE}}_{ji}(a)F)(\gamma) = \sum_{x\in\gamma_j}\left(\sum_{y\in\gamma_i} a(x-y)\right)(L^{x^-}_j F)(\gamma). \tag{88}$$

Using $F = KG$, the expression for $\widehat{L}G$ follows from its definition,

$$\begin{aligned}
(\widehat{L}G)(\eta) &= (K^{-1}L^{\mathrm{DE}}_{ji}(a)KG)(\eta)\\
&= -\sum_{\xi\subset\eta}(-1)^{|\eta\backslash\xi|}\sum_{x^{(j)}\in\xi}\sum_{y^{(i)}\in\xi\backslash x^{(j)}} a(x-y)(KD^{(j)}_x G)(\xi\backslash x^{(j)})\\
&= -\sum_{x^{(j)}\in\eta}\sum_{y^{(i)}\in\eta\backslash x^{(j)}}\sum_{\xi\subset\eta\backslash x^{(j)}\backslash y^{(i)}}(-1)^{|(\eta\backslash x^{(j)}\backslash y^{(i)})\backslash\xi|} a(x-y)(D^{(i)}_y K D^{(j)}_x G)(\xi)\\
&= -\sum_{x^{(j)}\in\eta}\sum_{y^{(i)}\in\eta\backslash x^{(j)}} a(x-y)(K^{-1}D^{(i)}_y K D^{(j)}_x G)(\eta\backslash x^{(j)}\backslash y^{(i)})\\
&= -\sum_{x^{(j)}\in\eta}\sum_{y^{(i)}\in\eta\backslash x^{(j)}} a(x-y)(G(\eta\backslash y^{(i)}) + G(\eta)).
\end{aligned}$$

Using $\widehat{L}G$ one can derive L^{Δ} as shown below:

$$\begin{aligned}
\int_{\Gamma_0}(\widehat{L}G)(\eta)k(\eta)d\lambda(\eta) &= -\int_{\Gamma_0}\sum_{x^{(j)}\in\eta}\sum_{y^{(i)}\in\eta\backslash x^{(j)}} a(x-y)\\
&\quad\times\Big((D^{(i)}_x G)(\eta\backslash x^{(j)}\backslash y^{(i)}) + (D^{(i)}_y D^{(i)}_x G)(\eta\backslash x^{(j)}\backslash y^{(i)})\Big)k(\eta)d\lambda(\eta)\\
&= -\int_{\Gamma_0}\sum_{x^{(j)}\in\eta}\sum_{y^{(i)}\in\eta\backslash x^{(j)}} a(x-y)\left(G(\eta\backslash y^{(i)}) + G(\eta)\right)k(\eta)d\lambda(\eta)\\
&= -\int_{\Gamma_0} G(\eta)\sum_{x^{(j)}\in\eta}\left(\int_{\mathbb{R}^d} dy k(\eta\cup y^{(i)}) + \sum_{y^{(i)}\in\eta\backslash x^{(j)}} k(\eta)\right) a(x-y)d\lambda(\eta);
\end{aligned}$$

$$(L^{\Delta}k)(\eta) = -\sum_{x^{(j)}\in\eta}\left(\int_{\mathbb{R}^d} dy k(\eta\cup y^{(i)}) + \sum_{y^{(i)}\in\eta\backslash x^{(j)}} k(\eta)\right) a(x-y). \tag{89}$$

In order to find $Q^\Delta = L^\Delta + M^\Delta$, one substitutes $\widehat{L}$ into the following equation, and transforms it so that it contains D operators explicitly:

$$\begin{aligned}
&\int_{\Gamma_0} \left(\widehat{L} e_\lambda(\theta)\right)(\eta)(\exp^* u)(\eta) d\lambda(\eta) = \\
&= -\int_{\Gamma_0} \sum_{x^{(j)}\in\eta} \sum_{y^{(i)}\in\eta\setminus x^{(j)}} a(x-y)(e_\lambda(\theta,\eta\setminus y^{(i)}) + e_\lambda(\theta,\eta))(\exp^* u)(\eta) d\lambda(\eta) \\
&= -\int_{\Gamma_0} \int_{(\mathbb{R}^d)^2} dxdy a(x-y)(\theta(x^{(j)}) + \theta(x^{(j)})\theta(y^{(i)})) e_\lambda(\theta,\eta) D_x^{(j)} D_y^{(i)} (\exp^* u)(\eta) d\lambda(\eta).
\end{aligned}$$

In order to find M^Δ, one substitutes $D_x D_y \exp^* u \longrightarrow D_y u * D_x u$ and obtains:

$$\begin{aligned}
&-\int_{\Gamma_0} \int_{(\mathbb{R}^d)^2} dxdy a(x-y)(\theta(x^{(j)}) + \theta(x^{(j)})\theta(y^{(i)})) e_\lambda(\theta,\eta)(D_x^{(j)} u * D_y^{(i)} u)(\eta) d\lambda(\eta) = \\
&\quad = -\int_{\Gamma_0} \sum_{x^{(j)}\in\eta} \int_{\mathbb{R}^d} dy a(x-y) e_\lambda(\theta,\eta)(D_x^{(j)} u * D_y^{(i)} u)(\eta\setminus x^{(j)}) d\lambda(\eta) \\
&\quad\quad - \int_{\Gamma_0} \sum_{x^{(j)},y^{(i)}\in\eta} a(x-y) e_\lambda(\theta,\eta)(D_x^{(j)} u * D_y^{(i)} u)(\eta\setminus x^{(j)}\setminus y^{(i)}) d\lambda(\eta) \\
&\quad = \int_{\Gamma_0} e_\lambda(\theta,\eta)\left(M^\Delta u\right)(\eta) d\lambda(\eta).
\end{aligned}$$

Thus, M^Δ is given by the following expression:

$$\begin{aligned}
(M^\Delta u)(\eta) &= -\sum_{x^{(j)}\in\eta} \int_{\mathbb{R}^d} dy a(x-y)(u(\cdot\cup x^{(j)}) * u(\cdot\cup y^{(i)}))(\eta\setminus x^{(j)}) \\
&\quad - \sum_{x^{(j)},y^{(i)}\in\eta} a(x-y)(u(\cdot\cup x^{(j)}) * u(\cdot\cup y^{(i)}))(\eta\setminus x^{(j)}\setminus y^{(i)}).
\end{aligned}$$

In order to find $L^\Delta_{\epsilon,\mathrm{ren}}$ and $M^\Delta_{\epsilon,\mathrm{ren}}$ one should obtain L^Δ_ϵ and M^Δ_ϵ from L^Δ and M^Δ by replacing potential a by a_ϵ. Then, following the definition, one obtains:

$$\begin{aligned}
(S_{\epsilon^{-1}} L^\Delta_\epsilon S_\epsilon k)(\eta) &= -\sum_{x^{(j)}\in\eta} \left(\int_{\mathbb{R}^d} dy k(\eta\cup y^{(i)}) + \epsilon^d \sum_{y^{(i)}\in\eta\setminus x^{(j)}} k(\eta) \right) a(x-y) \\
&= ((A + \epsilon^d B)k)(\eta),
\end{aligned}$$

from where one finds:

$$\begin{aligned}
(Ak)(\eta) &= -\sum_{x^{(j)}\in\eta} \int_{\mathbb{R}^d} dy k(\eta\cup y^{(i)}) a(x-y); \\
(Bk)(\eta) &= -\sum_{x^{(j)}\in\eta} \sum_{y^{(i)}\in\eta\setminus x^{(j)}} k(\eta) a(x-y).
\end{aligned}$$

In the same way, one obtains:

$$
\begin{aligned}
(S_{\epsilon^{-1}} M_\epsilon^\Delta S_\epsilon k)(\eta) &= -\sum_{x^{(j)}\in\eta} \int_{\mathbb{R}^d} dy a(x-y)(u(\cdot\cup x^{(j)}) * u(\cdot\cup y^{(i)}))(\eta\backslash x^{(j)}) \\
&\quad -\epsilon^d \sum_{x^{(j)},y^{(i)}\in\eta} a(x-y)(u(\cdot\cup x^{(j)}) * u(\cdot\cup y^{(i)}))(\eta\backslash x^{(j)}\backslash y^{(i)}) \\
&= ((C+\epsilon^d D)u)(\eta),
\end{aligned}
$$

where

$$
\begin{aligned}
(Cu)(\eta) &= -\sum_{x^{(j)}\in\eta} \int_{\mathbb{R}^d} dy a(x-y)(u(\cdot\cup x^{(j)}) * u(\cdot\cup y^{(i)}))(\eta\backslash x^{(j)}); \\
(Du)(\eta) &= -\sum_{x^{(j)},y^{(i)}\in\eta} a(x-y)(u(\cdot\cup x^{(j)}) * u(\cdot\cup y^{(i)}))(\eta\backslash x^{(j)}\backslash y^{(i)}).
\end{aligned}
$$

Thus, applying perturbation expansion in the limit $\epsilon^d \to 0$, one obtains:

$$
(Av + Cv)(\eta) = -\mathbb{1}_{\eta=\{x^{(m)}\}}\delta_{mj} \int_{\mathbb{R}^d} dy a(x-y) q_j(x) q_i(y). \tag{90}
$$

In order to obtain equations for corrections w, one uses

$$
\frac{\partial}{\partial t} w(t,\eta) = \left(Aw + Bv + Dv + C(v,w) + C(w,v)\right)(\eta),
$$

where

$$
\begin{aligned}
(Aw)(\eta) &= -\sum_{x^{(j)}\in\eta} \int_{\mathbb{R}^d} dy w(\eta\cup y^{(i)}) a(x-y) \\
&= \mathbb{1}_{\eta=\{x^{(m)}\}} \left(-\delta_{mj} \int_{\mathbb{R}^d} dy g_{ji}(x,y) a(x-y)\right); \\
(Bv)(\eta) &= -\sum_{x^{(j)}\in\eta} \sum_{y^{(i)}\in\eta\backslash x^{(j)}} v(\eta) a(x-y) = 0; \\
(Dv)(\eta) &= -\sum_{x^{(j)},y^{(i)}\in\eta} a(x-y)(v(\cdot\cup x^{(j)}) * v(\cdot\cup y^{(i)}))(\eta\backslash x^{(j)}\backslash y^{(i)}) \\
&= -\sum_{x^{(j)},y^{(i)}\in\eta} a(x-y) v(x^{(j)}) v(\eta\backslash x^{(j)}) \\
&= \mathbb{1}_{\eta=\{x_1^{(m)},x_2^{(n)}\}} (\\
&\quad -\delta_{mi}\delta_{nj} a(x_2-x_1) q_j(x_2) q_i(x_1) \\
&\quad -\delta_{mj}\delta_{ni} a(x_1-x_2) q_j(x_1) q_i(x_2));
\end{aligned}
$$

$$
\begin{aligned}
C(v,w)(\eta) &= -\sum_{x^{(j)}\in\eta}\int_{\mathbb{R}^d} dya(x-y)(v(\cdot\cup x^{(j)})*w(\cdot\cup y^{(i)}))(\eta\backslash x^{(j)}) \\
&= -\sum_{x^{(j)}\in\eta}\int_{\mathbb{R}^d} dya(x-y)v(x^{(j)})w((\eta\backslash x^{(j)})\cup y^{(i)}) \\
&= \mathbb{1}_{\eta=\{x^{(m)}\}}\delta_{mj}\left(-\int_{\mathbb{R}^d} dya(x-y)q_j(x)p_i(y)\right) \\
&\quad +\mathbb{1}_{\eta=\{x_1^{(m)},x_2^{(n)}\}}\Big(\\
&\quad -\delta_{mj}\delta_{nj}\int_{\mathbb{R}^d} dy[a(x_1-y)q_j(x_1)g_{ij}(y,x_2)+a(x_2-y)q_j(x_2)g_{ji}(x_1,y)] \\
&\quad -\delta_{m\neq j}\delta_{nj}\int_{\mathbb{R}^d} dya(x_2-y)q_j(x_2)g_{mi}(x_1,y) \\
&\quad -\delta_{mj}\delta_{n\neq j}\int_{\mathbb{R}^d} dya(x_1-y)q_j(x_1)g_{in}(y,x_2)\Big);
\end{aligned}
$$

$$
\begin{aligned}
C(w,v)(\eta) &= -\sum_{x^{(j)}\in\eta}\int_{\mathbb{R}^d} dya(x-y)(w(\cdot\cup x^{(j)})*v(\cdot\cup y^{(i)}))(\eta\backslash x^{(j)}) \\
&= -\sum_{x^{(j)}\in\eta}\int_{\mathbb{R}^d} dya(x-y)w(\eta)v(y^{(i)}) \\
&= \mathbb{1}_{\eta=\{x^{(m)}\}}\left(-\delta_{mj}\int_{\mathbb{R}^d} dya(x-y)p_j(x)q_i(y)\right) \\
&+ \mathbb{1}_{\eta=\{x_1^{(m)},x_2^{(n)}\}}\Big(-\delta_{mj}\delta_{nj}g_{jj}(x_1,x_2)\int_{\mathbb{R}^d} dy[a(x_1-y)+a(x_2-y)]q_i(y) \\
&\quad -\delta_{m\neq j}\delta_{nj}g_{mj}(x_1,x_2)\int_{\mathbb{R}^d} dya(x_2-y)q_i(y) \\
&\quad -\delta_{mj}\delta_{n\neq j}g_{jn}(x_1,x_2)\int_{\mathbb{R}^d} dya(x_1-y)q_i(y)\Big).
\end{aligned}
$$

As a result, one obtains:

$$
\begin{aligned}
H_{q_m}^{\mathrm{DE}}(x) &= -\delta_{mj}\int_{\mathbb{R}^d} dy a(x-y)q_j(x)q_i(y);\\
H_{p_m}^{\mathrm{DE}}(x) &= -\delta_{mj}\int_{\mathbb{R}^d} dy g_{ji}(x,y)a(x-y)\\
&\quad -\delta_{mj}q_j(x)\int_{\mathbb{R}^d} dy a(x-y)p_i(y)\\
&\quad -\delta_{mj}p_j(x)\int_{\mathbb{R}^d} dy a(x-y)q_i(y);\\
H_{g_{mn}}^{\mathrm{DE}}(x_1,x_2) &= -\delta_{mi}\delta_{nj}a(x_2-x_1)q_j(x_2)q_i(x_1)\\
&\quad -\delta_{mj}\delta_{ni}a(x_1-x_2)q_j(x_1)q_i(x_2)\\
&\quad -\delta_{mj}\delta_{nj}\int_{\mathbb{R}^d} dy[a(x_1-y)q_j(x_1)g_{ij}(y,x_2)+a(x_2-y)q_j(x_2)g_{ji}(x_1,y)]\\
&\quad -\delta_{m\neq j}\delta_{nj}\int_{\mathbb{R}^d} dy a(x_2-y)q_j(x_2)g_{mi}(x_1,y)\\
&\quad -\delta_{mj}\delta_{n\neq j}\int_{\mathbb{R}^d} dy a(x_1-y)q_j(x_1)g_{in}(y,x_2)\\
&\quad -\delta_{mj}\delta_{nj}g_{jj}(x_1,x_2)\int_{\mathbb{R}^d} dy[a(x_1-y)+a(x_2-y)]q_i(y)\\
&\quad -\delta_{m\neq j}\delta_{nj}g_{mj}(x_1,x_2)\int_{\mathbb{R}^d} dy a(x_2-y)q_i(y)\\
&\quad -\delta_{mj}\delta_{n\neq j}g_{jn}(x_1,x_2)\int_{\mathbb{R}^d} dy a(x_1-y)q_i(y).
\end{aligned}
\tag{91}
$$

In the translationally invariant case using a notation $A = \int_{\mathbb{R}^d} a(x)dx$ one obtains:

$$
\begin{aligned}
H_{q_m}^{\mathrm{DE}} &= -\delta_{mj}Aq_jq_i;\\
H_{p_m}^{\mathrm{DE}} &= \delta_{mj}\left(-A(q_jp_i+p_jq_i)-\int_{\mathbb{R}^d} dy g_{ji}(y)a(y)\right);\\
H_{g_{mn}}^{\mathrm{DE}}(x) &= \delta_{mj}\delta_{nj}\left(-2Aq_ig_{jj}(x)-2q_j\int_{\mathbb{R}^d} dy a(x-y)g_{ij}(y)\right)\\
&\quad -(\delta_{mi}\delta_{nj}+\delta_{mj}\delta_{ni})a(x)q_iq_j\\
&\quad +\delta_{m\neq j}\delta_{nj}\left(-Aq_ig_{mj}(x)-q_j\int_{\mathbb{R}^d} dy a(x-y)g_{mi}(y)\right)\\
&\quad +\delta_{mj}\delta_{n\neq j}\left(-Aq_ig_{jn}(x)-q_j\int_{\mathbb{R}^d} dy a(x-y)g_{in}(y)\right).
\end{aligned}
\tag{92}
$$

Death by competition, $L = L_i^{\mathrm{C}}(a)$

Process definition: type i individuals induce death in type i individuals with the kernel a,

$$
(L_i^{\mathrm{C}}(a)F)(\gamma) = \sum_{x\in\gamma_i}\left(\sum_{y\in\gamma_i} a(x-y)\right)(L_i^{x^-}F)(\gamma). \tag{93}
$$

This process is a special case of the process (88). In the translationally invariant case, results are obtained from (92) by making $j = i$ substitution and using $A = \int_{\mathbb{R}^d} a(x)dx$:

$$\begin{aligned}
H^{\mathrm{C}}_{q_m} &= -\delta_{mi} A q_i q_i;\\
H^{\mathrm{C}}_{p_m} &= \delta_{mi}\left(-2Aq_i p_i - \int_{\mathbb{R}^d} dy g_{ii}(y) a(y)\right);\\
H^{\mathrm{C}}_{g_{mn}}(x) &= \delta_{mi}\delta_{ni}\left(-2Aq_i g_{ii}(x) - 2q_i \int_{\mathbb{R}^d} dy a(x-y) g_{ii}(y) - 2a(x) q_i q_i\right)\\
&+\delta_{m\neq i}\delta_{ni}\left(-Aq_i g_{mi}(x) - q_i \int_{\mathbb{R}^d} dy a(x-y) g_{mi}(y)\right)\\
&+\delta_{mi}\delta_{n\neq i}\left(-Aq_i g_{in}(x) - q_i \int_{\mathbb{R}^d} dy a(x-y) g_{in}(y)\right). \qquad (94)
\end{aligned}$$

1.3.3 Processes defined by one birth and one death events by an operator $L^{x^- y^+}_{ij}$

Change in type, $L = L^{\mathrm{CT}}_{ji}(r)$

Process definition: individuals of type i change spontaneously into type j ($j \neq i$) at rate r,

$$(L^{\mathrm{CT}}_{ji}(r)F)(\gamma) = r \sum_{x\in\gamma_i} L^{x^- x^+}_{ij} F(\gamma). \qquad (95)$$

Using $F = KG$, one obtains:

$$\begin{aligned}
(L^{x^- x^+}_{ij} KG)(\xi) &= KG((\xi\backslash x^{(i)}) \cup x^{(j)}) - KG(\xi) = KG((\xi\backslash x^{(i)}) \cup x^{(j)})\\
&\quad -KG(\xi\backslash x^{(i)}) + KG(\xi\backslash x^{(i)}) - KG(\xi)\\
&= (KD^{(j)}_x G)(\xi\backslash x^{(i)}) - (KD^{(i)}_x G)(\xi\backslash x^{(i)}).
\end{aligned}$$

Using the change of variables $\xi \to \xi \cup x^{(i)}$, one obtains the expression for $\widehat{L}G$,

$$\begin{aligned}
(\widehat{L}G)(\eta) &= (K^{-1} L^{\mathrm{CT}}_{ji}(r) KG)(\eta)\\
&= r\sum_{\xi\subset\eta}(-1)^{|\eta\backslash\xi|} \sum_{x^{(i)}\in\xi}\left[(KD^{(j)}_x G)(\xi\backslash x^{(i)}) - (KD^{(i)}_x G)(\xi\backslash x^{(i)})\right]\\
&= r\sum_{x^{(i)}\in\eta}\ \sum_{\xi\subset\eta\backslash x^{(i)}} (-1)^{|(\eta\backslash x^{(i)})\backslash\xi|}\left[(KD^{(j)}_x G)(\xi) - (KD^{(i)}_x G)(\xi)\right]\\
&= r\sum_{x^{(i)}\in\eta}\left[(D^{(j)}_x G)(\eta\backslash x^{(i)}) - (D^{(i)}_x G)(\eta\backslash x^{(i)})\right]\\
&= r\sum_{x^{(i)}\in\eta}\left[G((\eta\backslash x^{(i)}) \cup x^{(j)}) - G(\eta)\right]\\
&= -r|\eta_i| G(\eta) + r\sum_{x^{(i)}\in\eta} G((\eta\backslash x^{(i)}) \cup x^{(j)}).
\end{aligned}$$

Using $\widehat{L}G$ one can derive L^{Δ} as shown below:

$$\begin{aligned}\int_{\Gamma_0}(\widehat{L}G)(\eta)k(\eta)d\lambda(\eta) &= -r|\eta_i|\int_{\Gamma_0}G(\eta)k(\eta)d\lambda(\eta)\\ &+r\int_{\Gamma_0}\sum_{x^{(i)}\in\eta}G((\eta\backslash x^{(i)})\cup x^{(j)})k(\eta)d\lambda(\eta).\end{aligned}$$

In the last term one can change variables, $\eta\to\eta\cup x^{(i)}$, and obtain:

$$\text{last term} = r\int_{\Gamma_0}\int_{\mathbb{R}^d}G(\eta\cup x^{(j)})k(\eta\cup x^{(i)})dxd\lambda(\eta).$$

After changing variables $\eta\to\eta\backslash x^{(j)}$ the last term becomes:

$$\text{last term} = r\int_{\Gamma_0}\sum_{x^{(j)}\in\eta}G(\eta)k((\eta\backslash x^{(j)})\cup x^{(i)})d\lambda(\eta).$$

Therefore, one obtains the following expression for L^{Δ},

$$(L^{\Delta}k)(\eta) = -r|\eta_i|k(\eta)+r\sum_{x^{(j)}\in\eta}k((\eta\backslash x^{(j)})\cup x^{(i)}). \tag{96}$$

In order to find Q^{Δ}, one substitutes $\widehat{L}$ into the following expression, and rewrites the resulting expression using D operations:

$$\begin{aligned}\int_{\Gamma_0}\left(\widehat{L}e_{\lambda}(\theta)\right)(\eta)(\exp^* u)(\eta)d\lambda(\eta) &= -\int_{\Gamma_0}r\sum_{x^{(i)}\in\eta}e_{\lambda}(\theta,\eta)(\exp^* u)(\eta)d\lambda(\eta)\\ &+\int_{\Gamma_0}r\sum_{x^{(i)}\in\eta}e_{\lambda}(\theta,(\eta\backslash x^{(i)})\cup x^{(j)})(\exp^* u)(\eta)d\lambda(\eta)\\ &= -\int_{\Gamma_0}r\int_{\mathbb{R}^d}dxe_{\lambda}(\theta,\eta)\theta(x^{(i)})D_x^{(i)}(\exp^* u)(\eta)d\lambda(\eta)\\ &+\int_{\Gamma_0}r\int_{\mathbb{R}^d}dxe_{\lambda}(\theta,\eta)\theta(x^{(j)})D_x^{(i)}(\exp^* u)(\eta)d\lambda(\eta).\end{aligned}$$

Terms with more than one operation D are absent here, therefore here $Q^{\Delta}=L^{\Delta}$. There is no spatial interaction, therefore $L^{\Delta}_{\epsilon,\mathrm{ren}}=L^{\Delta}=A$, where

$$(Au)(\eta) = -r|\eta_i|u(\eta)+\sum_{x^{(j)}\in\eta}ru((\eta\backslash x^{(j)})\cup x^{(i)}).$$

Thus, applying perturbation expansion in the limit $\epsilon^d\to 0$, one obtains:

$$(Av)(\eta) = \mathbb{1}_{\eta=\{x^{(m)}\}}\left(-\delta_{mi}+\delta_{mj}\right)rq_i(x). \tag{97}$$

In order to obtain equations for corrections w, one uses

$$\frac{\partial}{\partial t}w(t,\eta) = \left(Aw+Bv+Dv+C(v,w)+C(w,v)\right)(\eta),$$

where $B = 0, C = 0, D = 0$,

$$\begin{aligned}(Aw)(\eta) &= \mathbb{1}_{\eta=\{x^{(m)}\}}\left(-\delta_{mi}+\delta_{mj}\right)rp_i(x)\\ &+\mathbb{1}_{\{x_1^{(m)},x_2^{(n)}\}}\left[-\delta_{mi}\delta_{ni}\ 2rg_{ii}(x_1,x_2)\right.\\ &-\delta_{m\neq i}\delta_{ni}\ rg_{mi}(x_1,x_2)\\ &-\delta_{mi}\delta_{n\neq i}\ rg_{in}(x_1,x_2)\\ &+\delta_{mj}\delta_{nj}\ \left(rg_{ij}(x_1,x_2)+rg_{ji}(x_1,x_2)\right)\\ &+\delta_{m\neq j}\delta_{nj}\ rg_{mi}(x_1,x_2)\\ &\left.+\delta_{mj}\delta_{n\neq j}\ rg_{in}(x_1,x_2)\right].\end{aligned}$$

As a result, one obtains:

$$\begin{aligned}H^{\mathrm{CT}}_{q_m}(x) &= -\delta_{mi}rq_i(x)\\ &+\delta_{mj}rq_i(x);\end{aligned}$$

$$\begin{aligned}H^{\mathrm{CT}}_{p_m}(x) &= -\delta_{mi}rp_i(x)\\ &+\delta_{mj}rp_i(x);\end{aligned}$$

$$\begin{aligned}H^{\mathrm{CT}}_{g_{mn}}(x_1,x_2) &= -\delta_{mi}\delta_{ni}\ 2rg_{ii}(x_1,x_2)\\ &-\delta_{m\neq i}\delta_{ni}\ rg_{mi}(x_1,x_2)\\ &-\delta_{mi}\delta_{n\neq i}\ rg_{in}(x_1,x_2)\\ &+\delta_{mj}\delta_{nj}\ \left(rg_{ij}(x_1,x_2)+rg_{ji}(x_1,x_2)\right)\\ &+\delta_{m\neq j}\delta_{nj}\ rg_{mi}(x_1,x_2)\\ &+\delta_{mj}\delta_{n\neq j}\ rg_{in}(x_1,x_2).\end{aligned}$$

In the translationally invariant case,

$$\begin{aligned}H^{\mathrm{CT}}_{q_m} &= (\delta_{mj}-\delta_{mi})rq_i;\\ H^{\mathrm{CT}}_{p_m} &= (\delta_{mj}-\delta_{mi})rp_i;\\ H^{\mathrm{CT}}_{g_{mn}}(x) &= -\delta_{mi}\delta_{ni}\ 2rg_{ii}(x)\\ &-\delta_{m\neq i}\delta_{ni}\ rg_{mi}(x)\\ &-\delta_{mi}\delta_{n\neq i}\ rg_{in}(x)\\ &+\delta_{mj}\delta_{nj}\ 2rg_{ij}(x)\\ &+\delta_{m\neq j}\delta_{nj}\ rg_{mi}(x)\\ &+\delta_{mj}\delta_{n\neq j}\ rg_{in}(x).\end{aligned} \tag{98}$$

Jump and change in type $L = L^{\mathrm{JCT}}_{ji}(a)$

Process definition: type i individual jumps by the kernel a and changes to type j,

$$(L^{\mathrm{JCT}}_{ji}(a)F)(\gamma) = \sum_{x\in\gamma_i}\int_{\mathbb{R}^d} a(x-y)(L^{x^-y^+}_{ij}F)(\gamma)dy. \tag{99}$$

Using $F = KG$, one obtains:

$$\begin{aligned}(L_{ij}^{x^- y^+} KG)(\xi) &= KG((\xi \backslash x^{(i)}) \cup y^{(j)}) - KG(\xi) = KG((\xi \backslash x^{(i)}) \cup y^{(j)}) \\ &\quad - KG(\xi \backslash x^{(i)}) + KG(\xi \backslash x^{(i)}) - KG(\xi) \\ &= (KD_y^{(j)} G)(\xi \backslash x^{(i)}) - (KD_x^{(i)} G)(\xi \backslash x^{(i)}).\end{aligned}$$

Using the previous equation, the expression for $\widehat{L}G$ is the following,

$$\begin{aligned}(\widehat{L}G)(\eta) &= (K^{-1} L_{ji}^{\mathrm{JCT}}(a) KG)(\eta) \\ &= \sum_{\xi \subset \eta} (-1)^{|\eta \backslash \xi|} \sum_{x^{(i)} \in \xi} \int_{\mathbb{R}^d} dy a(x-y) \Big((KD_y^{(j)} G) - (KD_x^{(i)} G) \Big) (\xi \backslash x^{(i)}) \\ &= \sum_{x^{(i)} \in \eta} \sum_{\xi \subset \eta \backslash x^{(i)}} (-1)^{|(\eta \backslash x^{(i)}) \backslash \xi|} \int_{\mathbb{R}^d} dy a(x-y) \Big((KD_y^{(j)} G) - (KD_x^{(i)} G) \Big) (\xi) \\ &= \sum_{x^{(i)} \in \eta} \int_{\mathbb{R}^d} dy a(x-y) \Big((D_y^{(j)} G) - (D_x^{(i)} G) \Big) (\eta \backslash x^{(i)}) \\ &= \sum_{x^{(i)} \in \eta} \int_{\mathbb{R}^d} dy a(x-y) \big(G((\eta \backslash x^{(i)}) \cup y^{(j)}) - G(\eta) \big).\end{aligned}$$

Using $\widehat{L}G$ one can derive $L^{\triangle}$ as shown below:

$$\begin{aligned}\int_{\Gamma_0} (\widehat{L}G)(\eta) k(\eta) d\lambda(\eta) &= \int_{\Gamma_0} k(\eta) d\lambda(\eta) \Bigg(\sum_{x^{(i)} \in \eta} \int_{\mathbb{R}^d} dy a(x-y) G((\eta \backslash x^{(i)}) \cup y^{(j)}) \\ &\quad - \sum_{x^{(i)} \in \eta} \int_{\mathbb{R}^d} dy a(x-y) G(\eta) \Bigg) \\ &= \int_{\Gamma_0} d\lambda(\eta) \Bigg(\sum_{y^{(j)} \in \eta} \int_{\mathbb{R}^d} dx a(x-y) k((\eta \backslash y^{(j)}) \cup x^{(i)}) G(\eta) \\ &\quad - \sum_{x^{(i)} \in \eta} \int_{\mathbb{R}^d} dy a(x-y) k(\eta) G(\eta) \Bigg).\end{aligned}$$

Thus, $L^{\triangle}$ is obtained:

$$\begin{aligned}(L^{\triangle} k)(\eta) &= \sum_{y^{(j)} \in \eta} \int_{\mathbb{R}^d} dx a(x-y) k((\eta \backslash y^{(j)}) \cup x^{(i)}) \\ &\quad - \sum_{x^{(i)} \in \eta} \int_{\mathbb{R}^d} dy a(x-y) k(\eta). \end{aligned} \tag{100}$$

In order to find $Q^{\triangle}$, one substitutes $\widehat{L}$ into the following equation,

$$\begin{aligned}&\int_{\Gamma_0} \big(\widehat{L} e_\lambda(\theta) \big) (\eta) (\exp^* u)(\eta) d\lambda(\eta) = \\ &= \int_{\Gamma_0} \int_{(\mathbb{R}^d)^2} dx dy a(x-y) \big(\theta(y^{(j)}) - \theta(x^{(i)}) \big) e_\lambda(\theta, \eta) D_x^{(i)} (\exp^* u)(\eta) d\lambda(\eta).\end{aligned}$$

As there are no terms with more than one operation D, one can conclude that here $Q^{\Delta} = L^{\Delta}$. In order to find $L^{\Delta}_{\epsilon,\mathrm{ren}}$ one should obtain L^{Δ}_{ϵ} from L^{Δ} by replacing potentials a by a_{ϵ}. Then, following the definition, one obtains:

$$\begin{aligned}
(S_{\epsilon^{-1}} L^{\Delta}_{\epsilon} S_{\epsilon} k)(\eta) &= \sum_{y^{(j)}\in\eta} \int_{\mathbb{R}^d} dx a(x-y) k((\eta\backslash y^{(j)}) \cup x^{(i)}) \\
&\quad - \sum_{x^{(i)}\in\eta} \int_{\mathbb{R}^d} dy a(x-y) k(\eta) \\
&= (Ak)(\eta).
\end{aligned}$$

Thus, applying perturbation expansion in the limit $\epsilon^d \to 0$, one obtains:

$$(Av)(\eta) = \mathbb{1}_{\eta=\{x^{(m)}\}} \left[\delta_{mj} \int_{\mathbb{R}^d} dy a(x-y) q_i(y) - \delta_{mi} q_i(x) \int_{\mathbb{R}^d} dy a(x-y) \right]. \tag{101}$$

In order to obtain equations for corrections w, one uses

$$\frac{\partial}{\partial t} w(t,\eta) = (Aw + Bv + Dv + C(v,w) + C(w,v))(\eta),$$

where

$$\begin{aligned}
(Aw)(\eta) &= \sum_{x^{(j)}\in\eta} \int_{\mathbb{R}^d} dy a(x-y) w((\eta\backslash x^{(j)}) \cup y^{(i)}) - \sum_{x^{(i)}\in\eta} \int_{\mathbb{R}^d} dy a(x-y) w(\eta) \\
&= \mathbb{1}_{\eta=\{x^{(m)}\}} \left(\delta_{mj} \int_{\mathbb{R}^d} dy a(x-y) p_i(y) - \delta_{mi} p_i(x) \int_{\mathbb{R}^d} dy a(x-y) \right) \\
&\quad + \mathbb{1}_{\eta=\{x_1^{(m)},x_2^{(n)}\}} \Big(\\
&\quad + \delta_{mj}\delta_{nj} \int_{\mathbb{R}^d} dy [a(x_1-y) g_{ij}(y,x_2) + a(x_2-y) g_{ji}(x_1,y)] \\
&\quad + \delta_{m\neq j}\delta_{nj} \int_{\mathbb{R}^d} dy a(x_2-y) g_{mi}(x_1,y) \\
&\quad + \delta_{mj}\delta_{n\neq j} \int_{\mathbb{R}^d} dy a(x_1-y) g_{in}(y,x_2) \\
&\quad - \delta_{mi}\delta_{ni} g_{ii}(x_1,x_2) \int_{\mathbb{R}^d} dy [a(x_1-y) + a(x_2-y)] \\
&\quad - \delta_{m\neq i}\delta_{ni} g_{mi}(x_1,x_2) \int_{\mathbb{R}^d} dy a(x_2-y) \\
&\quad - \delta_{mi}\delta_{n\neq i} g_{in}(x_1,x_2) \int_{\mathbb{R}^d} dy a(x_1-y) \Big).
\end{aligned}$$

As a result, one obtains:

$$\begin{aligned}
H_{q_m}^{\mathrm{JCT}}(x) &= \delta_{mj}\int_{\mathbb{R}^d} dya(x-y)q_i(y)\\
&\quad -\delta_{mi}q_i(x)\int_{\mathbb{R}^d} dya(x-y);\\
H_{p_m}^{\mathrm{JCT}}(x) &= \delta_{mj}\int_{\mathbb{R}^d} dya(x-y)p_i(y)\\
&\quad -\delta_{mi}p_i(x)\int_{\mathbb{R}^d} dya(x-y);\\
H_{g_{mn}}^{\mathrm{JCT}}(x_1,x_2) &= +\delta_{mj}\delta_{nj}\int_{\mathbb{R}^d} dy[a(x_1-y)g_{ij}(y,x_2)+a(x_2-y)g_{ji}(x_1,y)]\\
&\quad +\delta_{m\neq j}\delta_{nj}\int_{\mathbb{R}^d} dya(x_2-y)g_{mi}(x_1,y)\\
&\quad +\delta_{mj}\delta_{n\neq j}\int_{\mathbb{R}^d} dya(x_1-y)g_{in}(y,x_2)\\
&\quad -\delta_{mi}\delta_{ni}\ g_{ii}(x_1,x_2)\int_{\mathbb{R}^d} dy[a(x_1-y)+a(x_2-y)]\\
&\quad -\delta_{m\neq i}\delta_{ni}\ g_{mi}(x_1,x_2)\int_{\mathbb{R}^d} dya(x_2-y)\\
&\quad -\delta_{mi}\delta_{n\neq i}\ g_{in}(x_1,x_2)\int_{\mathbb{R}^d} dya(x_1-y).
\end{aligned} \tag{102}$$

In the translationally invariant case, using $A=\int_{\mathbb{R}^d} a(x)dx$, one obtains:

$$\begin{aligned}
H_{q_m}^{\mathrm{JCT}} &= q_iA(\delta_{mj}-\delta_{mi});\\
H_{p_m}^{\mathrm{JCT}} &= p_iA(\delta_{mj}-\delta_{mi});\\
H_{g_{mn}}^{\mathrm{JCT}}(x) &= +\delta_{mj}\delta_{nj}2\int_{\mathbb{R}^d} dya(x-y)g_{ij}(y)\\
&\quad +\delta_{m\neq j}\delta_{nj}\int_{\mathbb{R}^d} dya(x-y)g_{mi}(y)\\
&\quad +\delta_{mj}\delta_{n\neq j}\int_{\mathbb{R}^d} dya(x-y)g_{in}(y)\\
&\quad -\delta_{mi}\delta_{ni}\ g_{ii}(x)2A\\
&\quad -\delta_{m\neq i}\delta_{ni}\ g_{mi}(x)A\\
&\quad -\delta_{mi}\delta_{n\neq i}\ g_{in}(x)A.
\end{aligned} \tag{103}$$

Jump $L=L_i^{\mathrm{J}}(a)$

Process definition: type i individual jumps by the kernel a,

$$(L_i^{\mathrm{J}}(a)F)(\gamma)=\sum_{x\in\gamma_i}\int_{\mathbb{R}^d} a(x-y)(L_{ii}^{x^-y^+}F)(\gamma)dy. \tag{104}$$

This process is a special case of the process (99). In the translationally invariant case, results are obtained from (103) by making j equal to i :

$$\begin{aligned}
H^{\mathrm{J}}_{q_m} &= 0;\\
H^{\mathrm{J}}_{p_m} &= 0;\\
H^{\mathrm{J}}_{g_{mn}}(x) &= +\delta_{mi}\delta_{ni}\left(2\int_{\mathbb{R}^d} dya(x-y)g_{ii}(y) - 2Ag_{ii}(x)\right)\\
&\quad +\delta_{m\neq i}\delta_{ni}\left(\int_{\mathbb{R}^d} dya(x-y)g_{mi}(y) - Ag_{mi}(x)\right)\\
&\quad +\delta_{mi}\delta_{n\neq i}\left(\int_{\mathbb{R}^d} dya(x-y)g_{in}(y) - Ag_{in}(x)\right).
\end{aligned} \tag{105}$$

Infection $L = L^{\mathrm{I}}_{ji}(a)$

Process description: individuals of type j change individuals of type i $(i \neq j)$ into type j with kernel a,

$$(L^{\mathrm{I}}_{ji}(a)F)(\gamma) = \sum_{y\in\gamma_j}\sum_{x\in\gamma_i} a(x-y)(L^{x^-x^+}_{ij}F)(\gamma). \tag{106}$$

Using $F = KG$,

$$\begin{aligned}
(L^{x^-x^+}_{ij}KG)(\xi) &= KG((\xi\backslash x^{(i)})\cup x^{(j)}) - KG(\xi) = KG((\xi\backslash x^{(i)})\cup x^{(j)})\\
&\quad -KG(\xi\backslash x^{(i)}) + KG(\xi\backslash x^{(i)}) - KG(\xi)\\
&= (KD^{(j)}_x G)(\xi\backslash x^{(i)}) - (KD^{(i)}_x G)(\xi\backslash x^{(i)}),
\end{aligned}$$

the expression for $\widehat{L}G$ follows from its definition using the change of variables, $\xi \to \xi \cup x^{(i)}$, and then $\xi \to \xi \cup y^{(j)}$:

$$\begin{aligned}
(\widehat{L}G)(\eta) &= (K^{-1}L^{\mathrm{I}}_{ji}(a)KG)(\eta)\\
&= \sum_{\xi\subset\eta}(-1)^{|\eta\backslash\xi|}\sum_{y^{(j)}\in\xi}\sum_{x^{(i)}\in\xi} a(x-y)\Big[(KD^{(j)}_x G)(\xi\backslash x^{(i)}) - (KD^{(i)}_x G)(\xi\backslash x^{(i)})\Big]\\
&= \sum_{x^{(i)}\in\eta}\ \sum_{\xi\subset\eta\backslash x^{(i)}}(-1)^{|(\eta\backslash x^{(i)})\backslash\xi|}\sum_{y^{(j)}\in\xi} a(x-y)\Big[(KD^{(j)}_x G)(\xi) - (KD^{(i)}_x G)(\xi)\Big]\\
&= \sum_{x^{(i)},y^{(j)}\in\eta}\ \sum_{\xi\subset\eta\backslash(x^{(i)}\cup y^{(j)})}(-1)^{|(\eta\backslash(x^{(i)}\cup y^{(j)}))\backslash\xi|}a(x-y)\Big[(D^{(j)}_y KD^{(j)}_x G)(\xi)\\
&\quad -(D^{(j)}_y KD^{(i)}_x G)(\xi)\Big]\\
&= \sum_{x^{(i)},y^{(j)}\in\eta} a(x-y)\Big[(K^{-1}D^{(j)}_y KD^{(j)}_x G)(\eta\backslash(x^{(i)}\cup y^{(j)}))\\
&\quad -(K^{-1}D^{(j)}_y KD^{(i)}_x G)(\eta\backslash(x^{(i)}\cup y^{(j)}))\Big].
\end{aligned}$$

Then, according to (41),

$$(D_y(KD^{(i)}_x G))(\eta) = (KD^{(i)}_x G)(\eta) + (KD_y D^{(i)}_x G)(\eta),$$

one obtains:

$$
\begin{aligned}
(\widehat{L}G)(\eta) &= \sum_{x^{(i)},y^{(j)}\in\eta} a(x-y)\Big[(D_x^{(j)}G)(\eta\backslash(x^{(i)}\cup y^{(j)})) + (D_y^{(j)}D_x^{(j)}G)(\eta\backslash(x^{(i)}\cup y^{(j)})) \\
&\quad -(D_x^{(i)}G)(\eta\backslash(x^{(i)}\cup y^{(j)})) - (D_y^{(j)}D_x^{(i)}G)(\eta\backslash(x^{(i)}\cup y^{(j)}))\Big] \\
&= \sum_{x^{(i)},y^{(j)}\in\eta} a(x-y)\left[G((\eta\cup x^{(j)})\backslash x^{(i)}\backslash y^{(j)}) + G((\eta\cup x^{(j)})\backslash x^{(i)})\right. \\
&\quad \left. -G(\eta\backslash y^{(j)}) - G(\eta)\right].
\end{aligned}
$$

Finding L^{Δ} from the definition,

$$
\int_{\Gamma_0}(\widehat{L}G)(\eta)k(\eta)d\lambda(\eta) = \int_{\Gamma_0} G(\eta)(L^{\Delta}k)(\eta)d\lambda(\eta),
$$

one uses the following changes of variables in each term:

$$
\begin{aligned}
&\text{in term 1}: \quad \eta\to\eta\cup x^{(i)}\cup y^{(j)}\backslash x^{(j)}; \\
&\text{in term 2}: \quad \eta\to\eta\cup x^{(i)}\backslash x^{(j)}; \\
&\text{in term 3}: \quad \eta\to\eta\cup y^{(j)}.
\end{aligned}
$$

As a result, one obtains L^{Δ},

$$
\begin{aligned}
(L^{\Delta}k)(\eta) &= +\sum_{x\in\eta_j}\int_{\mathbb{R}^d} dy\ a(x-y)k(\eta\cup x^{(i)}\cup y^{(j)}\backslash x^{(j)}) \\
&\quad +\sum_{x\in\eta_j}\sum_{y\in\eta_j\backslash x} a(x-y)k(\eta\cup x^{(i)}\backslash x^{(j)}) \\
&\quad -\sum_{x\in\eta_i}\int_{\mathbb{R}^d} dy\ a(x-y)k(\eta\cup y^{(j)}) \\
&\quad -\sum_{x\in\eta_i}\sum_{y\in\eta_j} a(x-y)k(\eta).
\end{aligned} \tag{107}
$$

In order to find $Q^{\Delta} = L^{\Delta} + M^{\Delta}$, one substitutes $\widehat{L}$ into the following expression,

$$
\begin{aligned}
\int_{\Gamma_0}\left(\widehat{L}e_\lambda(\theta)\right)(\eta)(\exp^* u)(\eta)d\lambda(\eta) &= \int_{\Gamma_0}\sum_{x^{(i)},y^{(j)}\in\eta} a(x-y)\left[e_\lambda(\theta,(\eta\cup x^{(j)})\backslash x^{(i)}\backslash y^{(j)})\right. \\
&\quad \left. + e_\lambda(\theta,(\eta\cup x^{(j)})\backslash x^{(i)}) - e_\lambda(\theta,\eta\backslash y^{(j)}) - e_\lambda(\theta,\eta)\right](\exp^* u)(\eta)d\lambda(\eta) \\
&= \int_{\Gamma_0}\int_{(\mathbb{R}^d)^2} dxdy\ a(x-y)e_\lambda(\theta,\eta)\left[\theta(x^{(j)}) + \theta(x^{(j)})\theta(y^{(j)}) - \theta(x^{(i)})\right. \\
&\quad \left. -\theta(x^{(i)})\theta(y^{(j)})\right] D_x^{(i)}D_y^{(j)}(\exp^* u)(\eta)d\lambda(\eta).
\end{aligned}
$$

In order to find M^{Δ}, one should keep only terms with more than one operation D, and use the substitution $D_x^{(i)}D_y^{(j)}(\exp^* u)(\eta) \longrightarrow (D_x^{(i)}u * D_y^{(j)}u)(\eta)$:

$$\int_{\Gamma_0}\int_{(\mathbb{R}^d)^2} dxdy\ a(x-y)e_\lambda(\theta,\eta)\times$$
$$\times\left[\theta(x^{(j)})+\theta(x^{(j)})\theta(y^{(j)})-\theta(x^{(i)})-\theta(x^{(i)})\theta(y^{(j)})\right](D_x^{(i)}u * D_y^{(j)}u)(\eta)d\lambda(\eta) =$$
$$= \int_{\Gamma_0} e_\lambda(\theta,\eta)\Bigg[\int_{\mathbb{R}^d} dy \sum_{x^{(j)}\in\eta} a(x-y)(D_x^{(i)}u * D_y^{(j)}u)(\eta\backslash x^{(j)})$$
$$+\sum_{x^{(j)}\in\eta}\sum_{y^{(j)}\in\eta\backslash x^{(j)}} a(x-y)(D_x^{(i)}u * D_y^{(j)}u)(\eta\backslash x^{(j)}\backslash y^{(j)})$$
$$-\int_{\mathbb{R}^d} dy \sum_{x^{(i)}\in\eta} a(x-y)(D_x^{(i)}u * D_y^{(j)}u)(\eta\backslash x^{(i)})$$
$$-\sum_{x^{(i)},y^{(j)}\in\eta} a(x-y)(D_x^{(i)}u * D_y^{(j)}u)(\eta\backslash x^{(i)}\backslash y^{(j)})\Bigg]d\lambda(\eta)$$
$$= \int_{\Gamma_0} e_\lambda(\theta,\eta)\left(M^{\Delta}u\right)(\eta)d\lambda(\eta).$$

Thus, M^{Δ} is given by the expression in square brackets in the previous formula, which can be rewritten in the following way:

$$\begin{aligned}(M^{\Delta}u)(\eta) \ &= \ \int_{\mathbb{R}^d} dy \sum_{x^{(j)}\in\eta} a(x-y)(u(\cdot\cup x^{(i)}) * u(\cdot\cup y^{(j)}))(\eta\backslash x^{(j)})\\ &+\sum_{x^{(j)}\in\eta}\sum_{y^{(j)}\in\eta\backslash x^{(j)}} a(x-y)(u(\cdot\cup x^{(i)}) * u(\cdot\cup y^{(j)}))(\eta\backslash x^{(j)}\backslash y^{(j)})\\ &-\int_{\mathbb{R}^d} dy \sum_{x^{(i)}\in\eta} a(x-y)(u(\cdot\cup x^{(i)}) * u(\cdot\cup y^{(j)}))(\eta\backslash x^{(i)})\\ &-\sum_{x^{(i)},y^{(j)}\in\eta} a(x-y)(u(\cdot\cup x^{(i)}) * u(\cdot\cup y^{(j)}))(\eta\backslash x^{(i)}\backslash y^{(j)}).\end{aligned} \tag{108}$$

In order to find $L^{\Delta}_{\epsilon,\mathrm{ren}}$ and $M^{\Delta}_{\epsilon,\mathrm{ren}}$ one should obtain L^{Δ}_{ϵ} and M^{Δ}_{ϵ} from L^{Δ} and M^{Δ} by replacing potentials a by a_ϵ. Then, following the definition, one obtains:

$$\begin{aligned}(S_{\epsilon^{-1}}L^{\Delta}_{\epsilon}S_\epsilon k)(\eta) \ &= \ +\sum_{x\in\eta_j}\int_{\mathbb{R}^d} dy\ a(x-y)k(\eta\cup x^{(i)}\cup y^{(j)}\backslash x^{(j)})\\ &+\epsilon^d\sum_{x\in\eta_j}\sum_{y\in\eta_j\backslash x} a(x-y)k(\eta\cup x^{(i)}\backslash x^{(j)})\\ &-\sum_{x\in\eta_i}\int_{\mathbb{R}^d} dy\ a(x-y)k(\eta\cup y^{(j)})\\ &-\epsilon^d\sum_{x\in\eta_i}\sum_{y\in\eta_j} a(x-y)k(\eta) = \left((A+\epsilon^d B)k\right)(\eta),\end{aligned}$$

where

$$\begin{aligned}(Ak)(\eta) &= +\sum_{x\in\eta_j}\int_{\mathbb{R}^d} dy\ a(x-y)k((\eta\backslash x^{(j)})\cup x^{(i)}\cup y^{(j)})\\ &\quad -\sum_{x\in\eta_i}\int_{\mathbb{R}^d} dy\ a(x-y)k(\eta\cup y^{(j)});\end{aligned}$$

$$\begin{aligned}(Bk)(\eta) &= \sum_{x\in\eta_j}\sum_{y\in\eta_j\backslash x} a(x-y)k((\eta\backslash x^{(j)})\cup x^{(i)})\\ &\quad -\sum_{x\in\eta_i}\sum_{y\in\eta_j} a(x-y)k(\eta).\end{aligned}$$

In the same way, one obtains:

$$\begin{aligned}(S_{\epsilon^{-1}}M_\epsilon^\Delta S_\epsilon u)(\eta) &= \int_{\mathbb{R}^d} dy \sum_{x^{(j)}\in\eta} a(x-y)(u(\cdot\cup x^{(i)})*u(\cdot\cup y^{(j)}))(\eta\backslash x^{(j)})\\ &\quad +\epsilon^d \sum_{x^{(j)}\in\eta}\sum_{y^{(j)}\in\eta\backslash x^{(j)}} a(x-y)(u(\cdot\cup x^{(i)})*u(\cdot\cup y^{(j)}))(\eta\backslash x^{(j)}\backslash y^{(j)})\\ &\quad -\int_{\mathbb{R}^d} dy\sum_{x^{(i)}\in\eta} a(x-y)(u(\cdot\cup x^{(i)})*u(\cdot\cup y^{(j)}))(\eta\backslash x^{(i)})\\ &\quad -\epsilon^d\sum_{x^{(i)},y^{(j)}\in\eta} a(x-y)(u(\cdot\cup x^{(i)})*u(\cdot\cup y^{(j)}))(\eta\backslash x^{(i)}\backslash y^{(j)})\\ &= \left((C+\epsilon^d D)u\right)(\eta),\end{aligned}$$

where

$$\begin{aligned}(Cu)(\eta) &= \int_{\mathbb{R}^d} dy \sum_{x^{(j)}\in\eta} a(x-y)(u(\cdot\cup x^{(i)})*u(\cdot\cup y^{(j)}))(\eta\backslash x^{(j)})\\ &\quad -\int_{\mathbb{R}^d} dy\sum_{x^{(i)}\in\eta} a(x-y)(u(\cdot\cup x^{(i)})*u(\cdot\cup y^{(j)}))(\eta\backslash x^{(i)});\\ (Du)(\eta) &= \sum_{x^{(j)}\in\eta}\sum_{y^{(j)}\in\eta\backslash x^{(j)}} a(x-y)(u(\cdot\cup x^{(i)})*u(\cdot\cup y^{(j)}))(\eta\backslash x^{(j)}\backslash y^{(j)})\\ &\quad -\sum_{x^{(i)},y^{(j)}\in\eta} a(x-y)(u(\cdot\cup x^{(i)})*u(\cdot\cup y^{(j)}))(\eta\backslash x^{(i)}\backslash y^{(j)}).\end{aligned}$$

Thus, applying perturbation expansion in the limit $\epsilon^d\to 0$, one obtains

$$(Av)(t,\eta)=0,$$

$$(Cv)(\eta)=\mathbb{1}_{\eta=\{x^{(m)}\}}(\delta_{jm}-\delta_{im})q_i(t,x)\int_{\mathbb{R}^d} dy\ a(x-y)q_j(t,y).$$

In order to obtain equations for corrections w, one uses

$$\frac{\partial}{\partial t}w(t,\eta)=\left(Aw+Bv+Dv+C(v,w)+C(w,v)\right)(\eta),$$

where

$$
\begin{aligned}
(Aw)(\eta) &= +\sum_{x\in\eta_j}\int_{\mathbb{R}^d} dy\ a(x-y)w((\eta\backslash x^{(j)})\cup x^{(i)}\cup y^{(j)}) \\
&\quad -\sum_{x\in\eta_i}\int_{\mathbb{R}^d} dy\ a(x-y)w(\eta\cup y^{(j)}) \\
&= \mathbb{1}_{\eta=\{x^{(m)}\}}\bigg(+\delta_{mj}\int_{\mathbb{R}^d} dy\ a(x-y)g_{ij}(x^{(i)},y^{(j)}) \\
&\quad -\delta_{mi}\int_{\mathbb{R}^d} dy\ a(x-y)g_{mj}(x^{(m)},y^{(j)})\bigg);
\end{aligned}
$$

$$
\begin{aligned}
(Bv)(\eta) &= \sum_{x\in\eta_j}\sum_{y\in\eta_j\backslash x} a(x-y)v((\eta\backslash x^{(j)})\cup x^{(i)}) \\
&\quad -\sum_{x\in\eta_i}\sum_{y\in\eta_j} a(x-y)v(\eta) \\
&= 0;
\end{aligned}
$$

$$
\begin{aligned}
(Dv)(\eta) &= \sum_{x^{(j)}\in\eta}\sum_{y^{(j)}\in\eta\backslash x^{(j)}} a(x-y)v(x^{(i)})v(\eta\backslash x^{(j)}) \\
&\quad -\sum_{x^{(i)},y^{(j)}\in\eta} a(x-y)v(x^{(i)})v(\eta\backslash x^{(i)}) \\
&= \mathbb{1}_{\eta=\{x_1^{(m)},x_2^{(n)}\}}\times(\\
&\quad +\delta_{mj}\delta_{nj}a(x_1-x_2)[q_i(x_1)q_j(x_2)+q_i(x_2)q_j(x_1)] \\
&\quad -\delta_{mi}\delta_{nj}a(x_1-x_2)q_i(x_1)q_j(x_2) \\
&\quad -\delta_{mj}\delta_{ni}a(x_2-x_1)q_i(x_2)q_j(x_1));
\end{aligned}
$$

$$
\begin{aligned}
C(v,w)(\eta) &= \int_{\mathbb{R}^d} dy \sum_{x^{(j)}\in\eta} a(x-y)(v(\cdot\cup x^{(i)}) * w(\cdot\cup y^{(j)}))(\eta\backslash x^{(j)}) \\
&\quad - \int_{\mathbb{R}^d} dy \sum_{x^{(i)}\in\eta} a(x-y)(v(\cdot\cup x^{(i)}) * w(\cdot\cup y^{(j)}))(\eta\backslash x^{(i)}) \\
&= \int_{\mathbb{R}^d} dy \sum_{x^{(j)}\in\eta} a(x-y)(v(x^{(i)})w((\eta\backslash x^{(j)})\cup y^{(j)})) \\
&\quad - \int_{\mathbb{R}^d} dy \sum_{x^{(i)}\in\eta} a(x-y)(v(x^{(i)})w((\eta\backslash x^{(i)})\cup y^{(j)})) \\
&= \mathbb{1}_{\eta=\{x^{(m)}\}} \times \Big(\\
&\quad +\delta_{mj} \int_{\mathbb{R}^d} dy\ a(x-y)q_i(x)p_j(y) \\
&\quad -\delta_{mi} \int_{\mathbb{R}^d} dy\ a(x-y)q_i(x)p_j(y)\Big) \\
&+ \mathbb{1}_{\eta=\{x_1^{(m)},x_2^{(n)}\}} \times \Big(\\
&\quad +\delta_{mj}\delta_{nj} \int_{\mathbb{R}^d} dy[a(x_1-y)q_i(x_1)g_{jj}(y,x_2) + a(x_2-y)q_i(x_2)g_{jj}(x_1,y)] \\
&\quad +\delta_{m\neq j}\delta_{nj} \int_{\mathbb{R}^d} dy a(x_2-y)q_i(x_2)g_{mj}(x_1,y) \\
&\quad +\delta_{mj}\delta_{n\neq j} \int_{\mathbb{R}^d} dy a(x_1-y)q_i(x_1)g_{jn}(y,x_2) \\
&\quad -\delta_{mi}\delta_{ni} \int_{\mathbb{R}^d} dy[a(x_1-y)q_i(x_1)g_{ji}(y,x_2) + a(x_2-y)q_i(x_2)g_{ij}(x_1,y)] \\
&\quad -\delta_{m\neq i}\delta_{ni} \int_{\mathbb{R}^d} dy a(x_2-y)q_i(x_2)g_{mj}(x_1,y) \\
&\quad -\delta_{mi}\delta_{n\neq i} \int_{\mathbb{R}^d} dy a(x_1-y)q_i(x_1)g_{jn}(y,x_2)\Big);
\end{aligned}
$$

$$
\begin{aligned}
C(w,v)(\eta) &= \int_{\mathbb{R}^d} dy \sum_{x^{(j)}\in\eta} a(x-y)(w(\cdot\cup x^{(i)}) * v(\cdot\cup y^{(j)}))(\eta\backslash x^{(j)}) \\
&\quad - \int_{\mathbb{R}^d} dy \sum_{x^{(i)}\in\eta} a(x-y)(w(\cdot\cup x^{(i)}) * v(\cdot\cup y^{(j)}))(\eta\backslash x^{(i)}) \\
&= \int_{\mathbb{R}^d} dy \sum_{x^{(j)}\in\eta} a(x-y)w((\eta\backslash x^{(j)})\cup x^{(i)})v(y^{(j)}) \\
&\quad - \int_{\mathbb{R}^d} dy \sum_{x^{(i)}\in\eta} a(x-y)w(\eta)v(y^{(j)}) \\
&= \mathbb{1}_{\eta=\{x^{(m)}\}}\Big(\\
&\quad +\delta_{mj}\int_{\mathbb{R}^d} dy a(x-y)p_i(x)q_j(y) \\
&\quad -\delta_{mi}\int_{\mathbb{R}^d} dy a(x-y)p_i(x)q_j(y)\Big) \\
&+ \mathbb{1}_{\eta=\{x_1^{(m)},x_2^{(n)}\}}\Big(\\
&\quad +\delta_{mj}\delta_{nj}\int_{\mathbb{R}^d} dy q_j(y)[a(x_1-y)g_{ij}(x_1,x_2)+a(x_2-y)g_{ji}(x_1,x_2)] \\
&\quad +\delta_{m\neq j}\delta_{nj}\int_{\mathbb{R}^d} dy a(x_2-y)g_{mi}(x_1,x_2)q_j(y) \\
&\quad +\delta_{mj}\delta_{n\neq j}\int_{\mathbb{R}^d} dy a(x_1-y)g_{in}(x_1,x_2)q_j(y) \\
&\quad -\delta_{mi}\delta_{ni}g_{ii}(x_1,x_2)\int_{\mathbb{R}^d} dy q_j(y)[a(x_1-y)+a(x_2-y)] \\
&\quad -\delta_{m\neq i}\delta_{ni}g_{mi}(x_1,x_2)\int_{\mathbb{R}^d} dy a(x_2-y)q_j(y) \\
&\quad -\delta_{mi}\delta_{n\neq i}g_{in}(x_1,x_2)\int_{\mathbb{R}^d} dy a(x_1-y)q_j(y)\Big).
\end{aligned}
$$

As a result, one obtains:

$$
\begin{aligned}
H^{\mathrm{I}}_{q_m}(x) &= \delta_{mj}q_i(x)\int_{\mathbb{R}^d} dy\ a(x-y)q_j(y) \\
&\quad -\delta_{mi}q_i(x)\int_{\mathbb{R}^d} dy\ a(x-y)q_j(y); \\
H^{\mathrm{I}}_{p_m}(x) &= +\delta_{mj}\left(\int_{\mathbb{R}^d} dy\ a(x-y)g_{ij}(x,y)+\int_{\mathbb{R}^d} dy\ a(x-y)[q_i(x)p_j(y)+p_i(x)q_j(y)]\right) \\
&\quad +\delta_{mi}\left(-\int_{\mathbb{R}^d} dy\ a(x-y)g_{ij}(x,y)-\int_{\mathbb{R}^d} dy\ a(x-y)[q_i(x)p_j(y)+p_i(x)q_j(y)]\right);
\end{aligned}
$$

$$
\begin{aligned}
H^{\rm I}_{g_{mk}}(x_1,x_2) \;=\; & +\delta_{mj}\delta_{nj}\Big(a(x_1-x_2)[q_i(x_1)q_j(x_2)+q_i(x_2)q_j(x_1)] \\
& \quad + \int_{\mathbb{R}^d} dy[a(x_1-y)q_i(x_1)g_{jj}(y,x_2)+a(x_2-y)q_i(x_2)g_{jj}(x_1,y)] \\
& \quad + \int_{\mathbb{R}^d} dy q_j(y)[a(x_1-y)g_{ij}(x_1,x_2)+a(x_2-y)g_{ji}(x_1,x_2)]\Big) \\
& -\delta_{mi}\delta_{nj}a(x_1-x_2)q_i(x_1)q_j(x_2) \\
& -\delta_{mj}\delta_{ni}a(x_2-x_1)q_i(x_2)q_j(x_1) \\
& +\delta_{m\neq j}\delta_{nj}\left(\int_{\mathbb{R}^d} dya(x_2-y)q_i(x_2)g_{mj}(x_1,y)+\int_{\mathbb{R}^d} dya(x_2-y)g_{mi}(x_1,x_2)q_j(y)\right) \\
& +\delta_{mj}\delta_{n\neq j}\left(\int_{\mathbb{R}^d} dya(x_1-y)q_i(x_1)g_{jn}(y,x_2)+\int_{\mathbb{R}^d} dya(x_1-y)g_{in}(x_1,x_2)q_j(y)\right) \\
& +\delta_{mi}\delta_{ni}\Big(-\int_{\mathbb{R}^d} dy[a(x_1-y)q_i(x_1)g_{ji}(y,x_2)+a(x_2-y)q_i(x_2)g_{ij}(x_1,y)] \\
& \quad -g_{ii}(x_1,x_2)\int_{\mathbb{R}^d} dyq_j(y)[a(x_1-y)+a(x_2-y)]\Big) \\
& +\delta_{m\neq i}\delta_{ni}\left(-\int_{\mathbb{R}^d} dya(x_2-y)q_i(x_2)g_{mj}(x_1,y)-g_{mi}(x_1,x_2)\int_{\mathbb{R}^d} dya(x_2-y)q_j(y)\right) \\
& +\delta_{mi}\delta_{n\neq i}\left(-g_{in}(x_1,x_2)\int_{\mathbb{R}^d} dya(x_1-y)q_j(y)-\int_{\mathbb{R}^d} dya(x_1-y)q_i(x_1)g_{jn}(y,x_2)\right).
\end{aligned}
\tag{109}
$$

In the translationally invariant case, using $A=\int_{\mathbb{R}^d} a(x)dx$, one obtains:

$$
\begin{aligned}
H^{\rm I}_{q_m} \;=\; & (\delta_{mj}-\delta_{mi})Aq_iq_j; \\
H^{\rm I}_{p_m} \;=\; & (\delta_{mj}-\delta_{mi})\left(A[q_ip_j+p_iq_j]+\int_{\mathbb{R}^d} dy\ a(y)g_{ij}(y)\right); \\
H^{\rm I}_{g_{mn}}(x) \;=\; & +\delta_{mj}\delta_{nj}\left(2a(x)q_iq_j+2Aq_jg_{ij}(x)+2q_i\int_{\mathbb{R}^d} dya(x-y)g_{jj}(y)\right) \\
& +\delta_{m\neq j}\delta_{nj}\left(Aq_jg_{mi}(x)+q_i\int_{\mathbb{R}^d} dya(x-y)g_{mj}(y)\right) \\
& +\delta_{mj}\delta_{n\neq j}\left(Aq_jg_{in}(x)+q_i\int_{\mathbb{R}^d} dya(x-y)g_{jn}(y)\right) \\
& -(\delta_{mi}\delta_{nj}+\delta_{mj}\delta_{ni})\,a(x)q_iq_j \\
& +\delta_{mi}\delta_{ni}\left(-2Aq_jg_{ii}(x)-2q_i\int_{\mathbb{R}^d} dya(x-y)g_{ji}(y)\right) \\
& +\delta_{m\neq i}\delta_{ni}\left(-Aq_jg_{mi}(x)-q_i\int_{\mathbb{R}^d} dya(x-y)g_{mj}(y)\right) \\
& +\delta_{mi}\delta_{n\neq i}\left(-Aq_jg_{in}(x)-q_i\int_{\mathbb{R}^d} dya(x-y)g_{jn}(y)\right).
\end{aligned}
\tag{110}
$$

Birth to another type by consumption, $L=L^{\rm BTC}_{kij}(a,b)$

Process definition: type i individuals 'die' when type j ($i\neq j$) individuals are around with kernel a, and simultaneously type k individuals appear near type j individuals with kernel b. For

example, type j may consume type i ($i \neq j$) and give birth to type k.

$$(L^{\mathrm{BTC}}_{kij}(a,b)F)(\gamma) = \sum_{y\in\gamma_j}\sum_{z\in\gamma_i}\int_{\mathbb{R}^d} a(z-y)b(x-y)(L^{z^-x^+}_{ik}F)(\gamma)dx. \tag{111}$$

Using $F = KG$,

$$\begin{aligned}(L^{z^-x^+}_{ik}KG)(\xi) &= KG((\xi\backslash z^{(i)})\cup x^{(k)}) - KG(\xi) = KG((\xi\backslash z^{(i)})\cup x^{(k)}) \\ &\quad -KG(\xi\backslash z^{(i)}) + KG(\xi\backslash z^{(i)}) - KG(\xi) \\ &= (KD^{(k)}_x G)(\xi\backslash z^{(i)}) - (KD^{(i)}_z G)(\xi\backslash z^{(i)}).\end{aligned}$$

The expression for the $\widehat{L}G$ follows from its definition:

$$\begin{aligned}(\widehat{L}G)(\eta) &= (K^{-1}L^{\mathrm{BTC}}_{kij}(a,b)KG)(\eta) \\ &= \sum_{\xi\subset\eta}(-1)^{|\eta\backslash\xi|}\sum_{y^{(j)}\in\xi}\sum_{z^{(i)}\in\xi}\int_{\mathbb{R}^d} dx a(z-y)b(x-y)\Big((KD^{(k)}_x G) - (KD^{(i)}_z G)\Big)(\xi\backslash z^{(i)}) \\ &= \sum_{y^{(j)},z^{(i)}\in\eta}\sum_{\xi\subset\eta\backslash y^{(j)}\backslash z^{(i)}}(-1)^{|(\eta\backslash y^{(j)}\backslash z^{(i)})\backslash\xi|}\int_{\mathbb{R}^d} dx a(z-y)b(x-y)\Big((D^{(j)}_y KD^{(k)}_x G) - (D^{(j)}_y KD^{(i)}_z G)\Big)(\xi) \\ &= \sum_{y^{(j)},z^{(i)}\in\eta}\int_{\mathbb{R}^d} dx a(z-y)b(x-y)\Big((K^{-1}D^{(j)}_y KD^{(k)}_x G) - (K^{-1}D^{(j)}_y KD^{(i)}_z G)\Big)(\eta\backslash y^{(j)}\backslash z^{(i)}) \\ &= \sum_{y^{(j)},z^{(i)}\in\eta}\int_{\mathbb{R}^d} dx a(z-y)b(x-y)\Big((D^{(k)}_x G) + (D^{(j)}_y D^{(k)}_x G) - (D^{(i)}_z G) - (D^{(j)}_y D^{(i)}_z G)\Big)(\eta\backslash y^{(j)}\backslash z^{(i)}).\end{aligned}$$

Thus, using $\widehat{L}$ one can derive L^{Δ} as shown below:

$$\begin{aligned}\int_{\Gamma_0}(\widehat{L}G)(\eta)k(\eta)d\lambda(\eta) &= \int_{\Gamma_0} k(\eta)d\lambda(\eta)\sum_{y^{(j)},z^{(i)}\in\eta}\int_{\mathbb{R}^d} dx a(z-y)b(x-y) \\ &\quad \Big(G((\eta\cup x^{(k)})\backslash y^{(j)}\backslash z^{(i)}) + G(\eta\cup x^{(k)}\backslash z^{(i)}) - G(\eta\backslash y^{(j)}) - G(\eta)\Big) \\ &= \int_{\Gamma_0} d\lambda(\eta)\Bigg(\sum_{x^{(k)}\in\eta}\int_{(\mathbb{R}^d)^2} dydz k((\eta\backslash x^{(k)})\cup z^{(i)}\cup y^{(j)})G(\eta) \\ &\quad + \sum_{y^{(j)},x^{(k)}\in\eta}\int_{\mathbb{R}^d} dz k((\eta\backslash x^{(k)})\cup z^{(i)})G(\eta) \\ &\quad - \sum_{z^{(i)}\in\eta}\int_{(\mathbb{R}^d)^2} dxdy k(\eta\cup y^{(j)})G(\eta) \\ &\quad - \sum_{y^{(j)},z^{(i)}\in\eta}\int_{\mathbb{R}^d} dx k(\eta)G(\eta)\Bigg)a(z-y)b(x-y).\end{aligned}$$

From the previous expression it follows, that L^{Δ} is given by the following expression:

$$
\begin{aligned}
(L^{\Delta}k)(\eta) = & \Bigg(\sum_{x^{(k)}\in\eta} \int_{(\mathbb{R}^d)^2} dydzk((\eta\backslash x^{(k)}) \cup z^{(i)} \cup y^{(j)}) \\
& + \sum_{y^{(j)},x^{(k)}\in\eta} \int_{\mathbb{R}^d} dzk((\eta\backslash x^{(k)}) \cup z^{(i)}) \\
& - \sum_{z^{(i)}\in\eta} \int_{(\mathbb{R}^d)^2} dxdyk(\eta \cup y^{(j)}) \\
& - \sum_{y^{(j)},z^{(i)}\in\eta} \int_{\mathbb{R}^d} dxk(\eta) \Bigg) a(z-y)b(x-y).
\end{aligned}
$$

In order to find $Q^{\Delta} = L^{\Delta} + M^{\Delta}$, one substitutes $\widehat{L}$ into the following equation,

$$
\begin{aligned}
\int_{\Gamma_0} \left(\widehat{L}e_{\lambda}(\theta)\right)(\eta)(\exp^* u)(\eta)d\lambda(\eta) = & \int_{\Gamma_0}\int_{(\mathbb{R}^d)^3} dxdydza(z-y)b(x-y)D_z^{(i)}D_y^{(j)}(\exp^* u)(\eta)d\lambda(\eta) \\
& \left(\theta(x^{(k)}) + \theta(x^{(k)})\theta(y^{(j)}) - \theta(z^{(i)}) - \theta(y^{(j)})\theta(z^{(i)})\right) e_{\lambda}(\theta,\eta).
\end{aligned}
$$

In order to find M^{Δ}, one keeps only terms with more than one operation D, and uses the substitution $D_x D_y \exp^* u \longrightarrow D_y u \star D_x u$,

$$
\begin{aligned}
& \int_{\Gamma_0}\int_{(\mathbb{R}^d)^3} dxdydza(z-y)b(x-y)(D_z^{(i)}u \star D_y^{(j)}u)(\eta)d\lambda(\eta)\times \\
& \quad \times \left(\theta(x^{(k)}) + \theta(x^{(k)})\theta(y^{(j)}) - \theta(z^{(i)}) - \theta(y^{(j)})\theta(z^{(i)})\right) e_{\lambda}(\theta,\eta) = \\
& = \int_{\Gamma_0} d\lambda(\eta) e_{\lambda}(\theta,\eta) \Bigg(\sum_{x^{(k)}\in\eta} \int_{(\mathbb{R}^d)^2} dydz(D_z^{(i)}u \star D_y^{(j)}u)(\eta\backslash x^{(k)}) \\
& \quad + \sum_{x^{(k)},y^{(j)}\in\eta} \int_{\mathbb{R}^d} dz(D_z^{(i)}u \star D_y^{(j)}u)(\eta\backslash x^{(k)}\backslash y^{(j)}) \\
& \quad - \sum_{z^{(i)}\in\eta} \int_{(\mathbb{R}^d)^2} dxdy(D_z^{(i)}u \star D_y^{(j)}u)(\eta\backslash z^{(i)}) \\
& \quad - \sum_{y^{(j)},z^{(i)}\in\eta} \int_{\mathbb{R}^d} dx(D_z^{(i)}u \star D_y^{(j)}u)(\eta\backslash y^{(j)}\backslash z^{(i)}) \Bigg) a(z-y)b(x-y) = \\
& = \int_{\Gamma_0} e_{\lambda}(\theta,\eta)\left(M^{\Delta}u\right)(\eta)d\lambda(\eta).
\end{aligned}
$$

As a result, M^Δ is given by the next expression:

$$\begin{aligned}\left(M^\Delta u\right)(\eta) &= \Bigg(\sum_{x^{(k)}\in\eta}\int_{(\mathbb{R}^d)^2} dydz(u(\cdot\cup z^{(i)}) * u(\cdot\cup y^{(j)}))(\eta\backslash x^{(k)}) \\ &+ \sum_{x^{(k)},y^{(j)}\in\eta}\int_{\mathbb{R}^d} dz(u(\cdot\cup z^{(i)}) * u(\cdot\cup y^{(j)}))(\eta\backslash x^{(k)}\backslash y^{(j)}) \\ &- \sum_{z^{(i)}\in\eta}\int_{(\mathbb{R}^d)^2} dxdy(u(\cdot\cup z^{(i)}) * u(\cdot\cup y^{(j)}))(\eta\backslash z^{(i)}) \\ &- \sum_{y^{(j)},z^{(i)}\in\eta}\int_{\mathbb{R}^d} dx(u(\cdot\cup z^{(i)}) * u(\cdot\cup y^{(j)}))(\eta\backslash y^{(j)}\backslash z^{(i)})\Bigg) a(z-y)b(x-y).\end{aligned} \tag{112}$$

In order to find $L^\Delta_{\epsilon,\mathrm{ren}}$ and $M^\Delta_{\epsilon,\mathrm{ren}}$ one should obtain L^Δ_ϵ and M^Δ_ϵ from L^Δ and M^Δ by replacing potentials a and b by a_ϵ and b_ϵ. Then, following the definition, one obtains:

$$\begin{aligned}(S_{\epsilon^{-1}}L^\Delta_\epsilon S_\epsilon k)(\eta) &= \sum_{x^{(k)}\in\eta}\int_{(\mathbb{R}^d)^2} dydzk((\eta\backslash x^{(k)})\cup z^{(i)}\cup y^{(j)})a(z-y)b(x-y) \\ &+\epsilon^d \sum_{y^{(j)},x^{(k)}\in\eta}\int_{\mathbb{R}^d} dzk((\eta\backslash x^{(k)})\cup z^{(i)})a(z-y)b(x-y) \\ &- \sum_{z^{(i)}\in\eta}\int_{(\mathbb{R}^d)^2} dxdyk(\eta\cup y^{(j)})a(z-y)b(x-y) \\ &-\epsilon^d \sum_{y^{(j)},z^{(i)}\in\eta}\int_{\mathbb{R}^d} dxk(\eta)a(z-y)b(x-y) \\ &= \left((A+\epsilon^d B)k\right)(\eta),\end{aligned}$$

where

$$\begin{aligned}(Ak)(\eta) &= \sum_{x^{(k)}\in\eta}\int_{(\mathbb{R}^d)^2} dydzk((\eta\backslash x^{(k)})\cup z^{(i)}\cup y^{(j)})a(z-y)b(x-y) \\ &- \sum_{z^{(i)}\in\eta}\int_{(\mathbb{R}^d)^2} dxdyk(\eta\cup y^{(j)})a(z-y)b(x-y); \\ (Bk)(\eta) &= \sum_{y^{(j)},x^{(k)}\in\eta}\int_{\mathbb{R}^d} dzk((\eta\backslash x^{(k)})\cup z^{(i)})a(z-y)b(x-y) \\ &- \sum_{y^{(j)},z^{(i)}\in\eta}\int_{\mathbb{R}^d} dxk(\eta)a(z-y)b(x-y).\end{aligned}$$

In the same way, one obtains:

$$
\begin{aligned}
(S_{\epsilon^{-1}}M_{\epsilon}^{\Delta}S_{\epsilon}k)(\eta) = \sum_{x^{(k)}\in\eta}\int_{(\mathbb{R}^d)^2} dydz(u(\cdot\cup z^{(i)}) * u(\cdot\cup y^{(j)}))(\eta\backslash x^{(k)})a(z-y)b(x-y) \\
+\epsilon^d \sum_{x^{(k)},y^{(j)}\in\eta}\int_{\mathbb{R}^d} dz(u(\cdot\cup z^{(i)}) * u(\cdot\cup y^{(j)}))(\eta\backslash x^{(k)}\backslash y^{(j)})a(z-y)b(x-y) \\
-\sum_{z^{(i)}\in\eta}\int_{(\mathbb{R}^d)^2} dxdy(u(\cdot\cup z^{(i)}) * u(\cdot\cup y^{(j)}))(\eta\backslash z^{(i)})a(z-y)b(x-y) \\
-\epsilon^d \sum_{y^{(j)},z^{(i)}\in\eta}\int_{\mathbb{R}^d} dx(u(\cdot\cup z^{(i)}) * u(\cdot\cup y^{(j)}))(\eta\backslash y^{(j)}\backslash z^{(i)})a(z-y)b(x-y) \\
= ((C+\epsilon^d D)u)(\eta),
\end{aligned}
$$

where

$$
\begin{aligned}
(Cu)(\eta) &= \sum_{x^{(k)}\in\eta}\int_{(\mathbb{R}^d)^2} dydz(u(\cdot\cup z^{(i)}) * u(\cdot\cup y^{(j)}))(\eta\backslash x^{(k)})a(z-y)b(x-y) \\
&\quad -\sum_{z^{(i)}\in\eta}\int_{(\mathbb{R}^d)^2} dxdy(u(\cdot\cup z^{(i)}) * u(\cdot\cup y^{(j)}))(\eta\backslash z^{(i)})a(z-y)b(x-y); \\
(Du)(\eta) &= \sum_{x^{(k)},y^{(j)}\in\eta}\int_{\mathbb{R}^d} dz(u(\cdot\cup z^{(i)}) * u(\cdot\cup y^{(j)}))(\eta\backslash x^{(k)}\backslash y^{(j)})a(z-y)b(x-y) \\
&\quad -\sum_{y^{(j)},z^{(i)}\in\eta}\int_{\mathbb{R}^d} dx(u(\cdot\cup z^{(i)}) * u(\cdot\cup y^{(j)}))(\eta\backslash y^{(j)}\backslash z^{(i)})a(z-y)b(x-y).
\end{aligned}
$$

Thus, applying perturbation expansion in the limit $\epsilon^d \to 0$, one obtains:

$$
\begin{aligned}
(Av+Cv)(\eta) &= \mathbb{1}_{\eta=\{x^{(m)}\}}\Big(\delta_{mk}\int_{(\mathbb{R}^d)^2} dydza(z-y)b(x-y)q_j(y)q_i(z) \\
&\quad -\delta_{mi}q_i(x)\int_{(\mathbb{R}^d)^2} dydza(x-y)b(z-y)q_j(y)\Big).
\end{aligned}
$$

In order to obtain equations for corrections w, one uses

$$
\frac{\partial}{\partial t}w(t,\eta) = (Aw + Bv + Dv + C(v,w) + C(w,v))(\eta),
$$

where

$$
\begin{aligned}
(Aw)(\eta) &= \sum_{x^{(k)}\in\eta}\int_{(\mathbb{R}^d)^2} dydzw((\eta\backslash x^{(k)})\cup z^{(i)}\cup y^{(j)})a(z-y)b(x-y) \\
&\quad -\sum_{z^{(i)}\in\eta}\int_{(\mathbb{R}^d)^2} dxdyw(\eta\cup y^{(j)})a(z-y)b(x-y) \\
&= \mathbb{1}_{\eta=\{x^{(m)}\}}\Big(\delta_{mk}\int_{(\mathbb{R}^d)^2} dydzg_{ij}(z,y)a(z-y)b(x-y) \\
&\quad -\delta_{mi}\int_{(\mathbb{R}^d)^2} dzdyg_{ij}(x,y)a(x-y)b(z-y)\Big);
\end{aligned}
$$

$$
\begin{aligned}
(Bv)(\eta) &= \sum_{y^{(j)},x^{(k)}\in\eta}\int_{\mathbb{R}^d} dzv((\eta\backslash x^{(k)})\cup z^{(i)})a(z-y)b(x-y)\\
&\quad-\sum_{y^{(j)},z^{(i)}\in\eta}\int_{\mathbb{R}^d} dxv(\eta)a(z-y)b(x-y)\\
&= 0;
\end{aligned}
$$

$$
\begin{aligned}
(Dv)(\eta) &= \sum_{x^{(k)},y^{(j)}\in\eta}\int_{\mathbb{R}^d} dz(v(\cdot\cup z^{(i)})*v(\cdot\cup y^{(j)}))(\eta\backslash x^{(k)}\backslash y^{(j)})a(z-y)b(x-y)\\
&\quad-\sum_{y^{(j)},z^{(i)}\in\eta}\int_{\mathbb{R}^d} dx(v(\cdot\cup z^{(i)})*v(\cdot\cup y^{(j)}))(\eta\backslash y^{(j)}\backslash z^{(i)})a(z-y)b(x-y)\\
&= \sum_{x^{(k)},y^{(j)}\in\eta}\int_{\mathbb{R}^d} dzv(z^{(i)})v(\eta\backslash x^{(k)})a(z-y)b(x-y)\\
&\quad-\sum_{y^{(j)},z^{(i)}\in\eta}\int_{\mathbb{R}^d} dxv(\eta\backslash y^{(j)})v(y^{(j)})a(z-y)b(x-y)\\
&= \sum_{x^{(k)},y^{(j)}\in\eta}\int_{\mathbb{R}^d} dzv(z^{(i)})v(\eta\backslash x^{(k)})a(z-y)b(x-y)\\
&\quad-\sum_{x^{(j)},z^{(i)}\in\eta}\int_{\mathbb{R}^d} dyv(\eta\backslash x^{(j)})v(x^{(j)})a(z-x)b(x-y)\\
&= \mathbb{1}_{\eta=\{x_1^{(m)},x_2^{(n)}\}}\Big(\\
&\quad+\delta_{mk}\delta_{nj}\int_{\mathbb{R}^d} dzq_i(z)q_j(x_2)a(z-x_2)b(x_1-x_2)\\
&\quad+\delta_{mj}\delta_{nk}\int_{\mathbb{R}^d} dzq_i(z)q_j(x_1)a(z-x_1)b(x_2-x_1)\\
&\quad-\delta_{mj}\delta_{ni}\int_{\mathbb{R}^d} dyq_i(x_2)q_j(x_1)a(x_2-x_1)b(x_1-y)\\
&\quad-\delta_{mi}\delta_{nj}\int_{\mathbb{R}^d} dyq_i(x_1)q_j(x_2)a(x_1-x_2)b(x_2-y)\Big);
\end{aligned}
$$

$$
\begin{aligned}
C(v,w)(\eta) &= \sum_{x^{(k)}\in\eta}\int_{(\mathbb{R}^d)^2} dydz(v(\cdot\cup z^{(i)})*w(\cdot\cup y^{(j)}))(\eta\backslash x^{(k)})a(z-y)b(x-y)\\
&\quad-\sum_{z^{(i)}\in\eta}\int_{(\mathbb{R}^d)^2} dxdy(v(\cdot\cup z^{(i)})*w(\cdot\cup y^{(j)}))(\eta\backslash z^{(i)})a(z-y)b(x-y)\\
&= \sum_{x^{(k)}\in\eta}\int_{(\mathbb{R}^d)^2} dydzv(z^{(i)})w((\eta\backslash x^{(k)})\cup y^{(j)})a(z-y)b(x-y)\\
&\quad-\sum_{z^{(i)}\in\eta}\int_{(\mathbb{R}^d)^2} dxdyv(z^{(i)})w((\eta\backslash z^{(i)})\cup y^{(j)})a(z-y)b(x-y)\\
&= \sum_{x^{(k)}\in\eta}\int_{(\mathbb{R}^d)^2} dydzv(z^{(i)})w((\eta\backslash x^{(k)})\cup y^{(j)})a(z-y)b(x-y)\\
&\quad-\sum_{x^{(i)}\in\eta}\int_{(\mathbb{R}^d)^2} dzdyv(x^{(i)})w((\eta\backslash x^{(i)})\cup y^{(j)})a(x-y)b(z-y)\\
&= \mathbb{1}_{\eta=\{x^{(m)}\}}\Big(\\
&\quad+\delta_{mk}\int_{(\mathbb{R}^d)^2} dydzq_i(z)p_j(y)a(z-y)b(x-y)\\
&\quad-\delta_{mi}\int_{(\mathbb{R}^d)^2} dzdyq_i(x)p_j(y)a(x-y)b(z-y)\Big)\\
&+\ \mathbb{1}_{\eta=\{x_1^{(m)},x_2^{(n)}\}}\Big(\\
&\quad+\delta_{mk}\delta_{nk}\int_{(\mathbb{R}^d)^2} dydzq_i(z)[g_{jk}(y,x_2)b(x_1-y)+g_{kj}(x_1,y)b(x_2-y)]a(z-y)\\
&\quad+\delta_{m\neq k}\delta_{nk}\int_{(\mathbb{R}^d)^2} dydzq_i(z)g_{mj}(x_1,y)b(x_2-y)a(z-y)\\
&\quad+\delta_{mk}\delta_{n\neq k}\int_{(\mathbb{R}^d)^2} dydzq_i(z)g_{jn}(y,x_2)b(x_1-y)a(z-y)\\
&\quad-\delta_{mi}\delta_{ni}\int_{(\mathbb{R}^d)^2} dzdy[g_{ji}(y,x_2)q_i(x_1)a(x_1-y)+g_{ij}(x_1,y)q_i(x_2)a(x_2-y)]b(z-y)\\
&\quad-\delta_{m\neq i}\delta_{ni}\int_{(\mathbb{R}^d)^2} dzdyq_i(x_2)g_{mj}(x_1,y)a(x_2-y)b(z-y)\\
&\quad-\delta_{mi}\delta_{n\neq i}\int_{(\mathbb{R}^d)^2} dzdyq_i(x_1)g_{jn}(y,x_2)a(x_1-y)b(z-y)\Big);
\end{aligned}
$$

$$
\begin{aligned}
C(w,v)(\eta) &= \sum_{x^{(k)}\in\eta}\int_{(\mathbb{R}^d)^2} dydz(w(\cdot\cup z^{(i)}) * v(\cdot\cup y^{(j)}))(\eta\backslash x^{(k)})a(z-y)b(x-y)\\
&\quad -\sum_{z^{(i)}\in\eta}\int_{(\mathbb{R}^d)^2} dxdy(w(\cdot\cup z^{(i)}) * v(\cdot\cup y^{(j)}))(\eta\backslash z^{(i)})a(z-y)b(x-y)\\
&= \sum_{x^{(k)}\in\eta}\int_{(\mathbb{R}^d)^2} dydzw((\eta\backslash x^{(k)})\cup z^{(i)})v(y^{(j)})a(z-y)b(x-y)\\
&\quad -\sum_{z^{(i)}\in\eta}\int_{(\mathbb{R}^d)^2} dxdyw(\eta)v(y^{(j)})a(z-y)b(x-y)\\
&= \sum_{x^{(k)}\in\eta}\int_{(\mathbb{R}^d)^2} dydzw((\eta\backslash x^{(k)})\cup z^{(i)})v(y^{(j)})a(z-y)b(x-y)\\
&\quad -\sum_{x^{(i)}\in\eta}\int_{(\mathbb{R}^d)^2} dzdyw(\eta)v(y^{(j)})a(x-y)b(z-y)\\
&= \mathbb{1}_{\eta=\{x^{(m)}\}}\Big(+\delta_{mk}\int_{(\mathbb{R}^d)^2} dydzp_i(z)q_j(y)a(z-y)b(x-y)\\
&\quad -\delta_{mi}p_i(x)\int_{(\mathbb{R}^d)^2} dzdyq_j(y)a(x-y)b(z-y)\Big)\\
&+ \mathbb{1}_{\eta=\{x_1^{(m)},x_2^{(n)}\}}\Big(\\
&\quad +\delta_{mk}\delta_{nk}\int_{(\mathbb{R}^d)^2} dydzq_j(y)[g_{ik}(z,x_2)b(x_1-y)+g_{ki}(x_1,z)b(x_2-y)]a(z-y)\\
&\quad +\delta_{m\neq k}\delta_{nk}\int_{(\mathbb{R}^d)^2} dydzq_j(y)g_{mi}(x_1,z)a(z-y)b(x_2-y)\\
&\quad +\delta_{mk}\delta_{n\neq k}\int_{(\mathbb{R}^d)^2} dydzq_j(y)g_{in}(z,x_2)a(z-y)b(x_1-y)\\
&\quad -\delta_{mi}\delta_{ni}\int_{(\mathbb{R}^d)^2} dzdyq_j(y)g_{ii}(x_1,x_2)[a(x_1-y)+a(x_2-y)]b(z-y)\\
&\quad -\delta_{m\neq i}\delta_{ni}\int_{(\mathbb{R}^d)^2} dzdyq_j(y)g_{mi}(x_1,x_2)a(x_2-y)b(z-y)\\
&\quad -\delta_{mi}\delta_{n\neq i}\int_{(\mathbb{R}^d)^2} dzdyq_j(y)g_{in}(x_1,x_2)a(x_1-y)b(z-y))\Big).
\end{aligned}
$$

As a result, one obtains:

$$
\begin{aligned}
H_{q_m}^{\mathrm{BTC}}(x) &= \delta_{mk}\int_{(\mathbb{R}^d)^2} dydza(z-y)b(x-y)q_j(y)q_i(z)\\
&\quad -\delta_{mi}q_i(x)\int_{(\mathbb{R}^d)^2} dydza(x-y)b(z-y)q_j(y); \qquad (113)
\end{aligned}
$$

$$\begin{aligned}
H^{\mathrm{BTC}}_{p_m}(x) &= \delta_{mk}\int_{(\mathbb{R}^d)^2} dydz g_{ij}(z,y)a(z-y)b(x-y)\\
&-\delta_{mi}\int_{(\mathbb{R}^d)^2} dzdy g_{ij}(x,y)a(x-y)b(z-y)\\
&+\delta_{mk}\int_{(\mathbb{R}^d)^2} dydz q_i(z)p_j(y)a(z-y)b(x-y)\\
&-\delta_{mi}\int_{(\mathbb{R}^d)^2} dzdy q_i(x)p_j(y)a(x-y)b(z-y)\\
&+\delta_{mk}\int_{(\mathbb{R}^d)^2} dydz p_i(z)q_j(y)a(z-y)b(x-y)\\
&-\delta_{mi}p_i(x)\int_{(\mathbb{R}^d)^2} dzdy q_j(y)a(x-y)b(z-y); \qquad (114)
\end{aligned}$$

$$\begin{aligned}
H^{\mathrm{BTC}}_{g_{mn}}(x_1,x_2) &= +\delta_{mk}\delta_{nj}\int_{\mathbb{R}^d} dz q_i(z)q_j(x_2)a(z-x_2)b(x_1-x_2)\\
&+\delta_{mj}\delta_{nk}\int_{\mathbb{R}^d} dz q_i(z)q_j(x_1)a(z-x_1)b(x_2-x_1)\\
&-\delta_{mj}\delta_{ni}\int_{\mathbb{R}^d} dy q_i(x_2)q_j(x_1)a(x_2-x_1)b(x_1-y)\\
&-\delta_{mi}\delta_{nj}\int_{\mathbb{R}^d} dy q_i(x_1)q_j(x_2)a(x_1-x_2)b(x_2-y)\\
&+\delta_{mk}\delta_{nk}\int_{(\mathbb{R}^d)^2} dydz q_i(z)[g_{jk}(y,x_2)b(x_1-y)+g_{kj}(x_1,y)b(x_2-y)]a(z-y)\\
&+\delta_{m\neq k}\delta_{nk}\int_{(\mathbb{R}^d)^2} dydz q_i(z)g_{mj}(x_1,y)b(x_2-y)a(z-y)\\
&+\delta_{mk}\delta_{n\neq k}\int_{(\mathbb{R}^d)^2} dydz q_i(z)g_{jn}(y,x_2)b(x_1-y)a(z-y)\\
&-\delta_{mi}\delta_{ni}\int_{(\mathbb{R}^d)^2} dzdy[g_{ji}(y,x_2)q_i(x_1)a(x_1-y)+g_{ij}(x_1,y)q_i(x_2)a(x_2-y)]b(z-y)\\
&-\delta_{m\neq i}\delta_{ni}\int_{(\mathbb{R}^d)^2} dzdy q_i(x_2)g_{mj}(x_1,y)a(x_2-y)b(z-y)\\
&-\delta_{mi}\delta_{n\neq i}\int_{(\mathbb{R}^d)^2} dzdy q_i(x_1)g_{jn}(y,x_2)a(x_1-y)b(z-y)\\
&+\delta_{mk}\delta_{nk}\int_{(\mathbb{R}^d)^2} dydz q_j(y)[g_{ik}(z,x_2)b(x_1-y)+g_{ki}(x_1,z)b(x_2-y)]a(z-y)\\
&+\delta_{m\neq k}\delta_{nk}\int_{(\mathbb{R}^d)^2} dydz q_j(y)g_{mi}(x_1,z)a(z-y)b(x_2-y)\\
&+\delta_{mk}\delta_{n\neq k}\int_{(\mathbb{R}^d)^2} dydz q_j(y)g_{in}(z,x_2)a(z-y)b(x_1-y)\\
&-\delta_{mi}\delta_{ni}\int_{(\mathbb{R}^d)^2} dzdy q_j(y)g_{ii}(x_1,x_2)[a(x_1-y)+a(x_2-y)]b(z-y)\\
&-\delta_{m\neq i}\delta_{ni}\int_{(\mathbb{R}^d)^2} dzdy q_j(y)g_{mi}(x_1,x_2)a(x_2-y)b(z-y)\\
&-\delta_{mi}\delta_{n\neq i}\int_{(\mathbb{R}^d)^2} dzdy q_j(y)g_{in}(x_1,x_2)a(x_1-y)b(z-y)). \qquad (115)
\end{aligned}$$

In the translationally invariant case, using notations $A = \int_{\mathbb{R}^d} a(x)dx$, $B = \int_{\mathbb{R}^d} b(x)dx$, one obtains:

$$\begin{aligned}
H^{\mathrm{BTC}}_{q_m} &= (\delta_{mk} - \delta_{mi})q_j q_i AB;\\
H^{\mathrm{BTC}}_{p_m} &= \delta_{mk}\Big((q_i p_j + p_i q_j)AB + B\int_{\mathbb{R}^d} dy g_{ij}(y)a(y)\Big)\\
&\quad +\delta_{mi}\Big(-(q_i p_j + p_i q_j)AB - B\int_{\mathbb{R}^d} dy g_{ij}(y)a(y)\Big);\\
H^{\mathrm{BTC}}_{g_{mn}}(x) &= +\delta_{mk}\delta_{nk}\Big(2Aq_i\int_{\mathbb{R}^d} dy g_{jk}(y)b(x-y) + 2q_j\int_{(\mathbb{R}^d)^2} dydz g_{ik}(z)b(y)a(x-y-z)\Big)\\
&\quad +(\delta_{mk}\delta_{nj} + \delta_{mj}\delta_{nk})q_i q_j A b(x)\\
&\quad -(\delta_{mj}\delta_{ni} + \delta_{mi}\delta_{nj})q_i q_j a(x)B\\
&\quad +\delta_{m\neq k}\delta_{nk}\Big(Aq_i\int_{\mathbb{R}^d} dy g_{mj}(y)b(x-y) + q_j\int_{(\mathbb{R}^d)^2} dydz g_{mi}(z)b(y)a(x-y-z)\Big)\\
&\quad +\delta_{mk}\delta_{n\neq k}\Big(Aq_i\int_{\mathbb{R}^d} dy g_{jn}(y)b(x-y) + q_j\int_{(\mathbb{R}^d)^2} dydz g_{in}(z)b(y)a(x-y-z)\Big)\\
&\quad -\delta_{mi}\delta_{ni}\Big(2Bq_i\int_{\mathbb{R}^d} dy g_{ji}(y)a(x-y) + 2ABq_j g_{ii}(x)\Big)\\
&\quad -\delta_{m\neq i}\delta_{ni}\Big(Bq_i\int_{\mathbb{R}^d} dy g_{mj}(y)a(x-y) + ABq_j g_{mi}(x)\Big)\\
&\quad -\delta_{mi}\delta_{n\neq i}\Big(Bq_i\int_{\mathbb{R}^d} dy g_{jn}(y)a(x-y) + ABq_j g_{in}(x)\Big).
\end{aligned} \tag{116}$$

Birth by consumption, $L = L^{\mathrm{BC}}_{ji}(a, b)$

Process definition: type i individuals 'die' when type j $(i \neq j)$ individuals are around with kernel a, and simultaneously type j new individuals appear near type j individuals with kernel b,

$$(L^{BC}_{ji}F)(\gamma) = \sum_{y\in\gamma_j}\sum_{z\in\gamma_i}\int_{\mathbb{R}^d} a(z-y)b(x-y)(L^{z^- x^+}_{ij}F)(\gamma)dx. \tag{117}$$

This process is a special case of the process (111). In the translationally invariant case results are obtained from (116) when k is equal to j :

$$
\begin{aligned}
H^{\mathrm{BC}}_{q_m} &= (\delta_{mj} - \delta_{mi}) q_j q_i AB;\\
H^{\mathrm{BC}}_{p_m} &= \delta_{mj}\left((q_i p_j + p_i q_j) AB + B\int_{\mathbb{R}^d} dy g_{ij}(y) a(y)\right)\\
&\quad +\delta_{mi}\left(-(q_i p_j + p_i q_j) AB - B\int_{\mathbb{R}^d} dy g_{ij}(y) a(y)\right);\\
H^{\mathrm{BC}}_{g_{mn}}(x) &= -(\delta_{mj}\delta_{ni} + \delta_{mi}\delta_{nj}) q_i q_j a(x) B\\
&\quad +\delta_{mj}\delta_{nj}\left(2q_i q_j A b(x) + 2Aq_i\int_{\mathbb{R}^d} dy g_{jj}(y) b(x-y)\right.\\
&\qquad \left. +2q_j\int_{(\mathbb{R}^d)^2} dydz g_{ij}(z) b(y) a(x-y-z)\right)\\
&\quad +\delta_{m\neq j}\delta_{nj}\left(Aq_i\int_{\mathbb{R}^d} dy g_{mj}(y) b(x-y) + q_j\int_{(\mathbb{R}^d)^2} dydz g_{mi}(z) b(y) a(x-y-z)\right)\\
&\quad +\delta_{mj}\delta_{n\neq j}\left(Aq_i\int_{\mathbb{R}^d} dy g_{jn}(y) b(x-y) + q_j\int_{(\mathbb{R}^d)^2} dydz g_{in}(z) b(y) a(x-y-z)\right)\\
&\quad +\delta_{mi}\delta_{ni}\left(-2Bq_i\int_{\mathbb{R}^d} dy g_{ji}(y) a(x-y) - 2ABq_j g_{ii}(x)\right)\\
&\quad +\delta_{m\neq i}\delta_{ni}\left(-Bq_i\int_{\mathbb{R}^d} dy g_{mj}(y) a(x-y) - ABq_j g_{mi}(x)\right)\\
&\quad +\delta_{mi}\delta_{n\neq i}\left(-Bq_i\int_{\mathbb{R}^d} dy g_{jn}(y) a(x-y) - ABq_j g_{in}(x)\right). \qquad (118)
\end{aligned}
$$

1.3.4 Processes defined by three birth-death events by an operator $L^{x^- y^- y^+}_{ijk}$

Change in type by consumption, $L = L^{\mathrm{CTC}}_{kji}(a)$

Process definition: type j individual changes to type k $(k \neq j)$ individual by consuming a type i $(i \notin \{k, j\})$ individual with kernel a,

$$
(L^{\mathrm{CTC}}_{kji}(a)F)(\gamma) = \sum_{y\in\gamma_j}\sum_{x\in\gamma_i} a(x-y)(L^{x^- y^- y^+}_{ijk} F)(\gamma). \qquad (119)
$$

Using $F = KG$,

$$
\begin{aligned}
(L^{x^- y^- y^+}_{ijk} KG)(\xi) &= KG((\xi\backslash x^{(i)}\backslash y^{(j)}) \cup y^{(k)}) - KG(\xi)\\
&= KG((\xi\backslash x^{(i)}\backslash y^{(j)}) \cup y^{(k)}) - KG(\xi\backslash x^{(i)}\backslash y^{(j)})\\
&\quad +KG(\xi\backslash x^{(i)}\backslash y^{(j)}) - KG(\xi\backslash x^{(i)})\\
&\quad +KG(\xi\backslash x^{(i)}) - KG(\xi)\\
&= (KD^{(k)}_y G)(\xi\backslash x^{(i)}\backslash y^{(j)}) - (KD^{(j)}_y G)(\xi\backslash x^{(i)}\backslash y^{(j)}) - (KD^{(i)}_x G)(\xi\backslash x^{(i)}).
\end{aligned}
$$

The expression for $\widehat{L}G$ follows from its definition:

$$
\begin{aligned}
(\widehat{L}G)(\eta) &= (K^{-1}L^{\mathrm{CTC}}_{kji}(a)KG)(\eta) \\
&= \sum_{\xi\subset\eta}(-1)^{|\eta\setminus\xi|}\sum_{x^{(i)},y^{(j)}\in\xi} a(x-y)\Big[\Big(KD_y^{(k)}G - KD_y^{(j)}G\Big)(\xi\setminus x^{(i)}\setminus y^{(j)}) - (KD_x^{(i)}G)(\xi\setminus x^{(i)})\Big] \\
&= \sum_{x^{(i)},y^{(j)}\in\eta}\ \sum_{\xi\subset\eta\setminus x^{(i)}\setminus y^{(j)}} (-1)^{|(\eta\setminus x^{(i)}\setminus y^{(j)})\setminus\xi|} a(x-y) \\
&\quad \times\Big[\Big(KD_y^{(k)}G - KD_y^{(j)}G\Big)(\xi) - (D_y^{(j)}KD_x^{(i)}G)(\xi)\Big] \\
&= \sum_{x^{(i)},y^{(j)}\in\eta} a(x-y)\Big((D_y^{(k)}G) - (D_y^{(j)}G) - (K^{-1}D_y^{(j)}KD_x^{(i)}G)\Big)(\eta\setminus x^{(i)}\setminus y^{(j)}) \\
&= \sum_{x^{(i)},y^{(j)}\in\eta} a(x-y)\Big((D_y^{(k)}G) - (D_y^{(j)}G) - (D_x^{(i)}G) - (D_y^{(j)}D_x^{(i)}G)\Big)(\eta\setminus x^{(i)}\setminus y^{(j)}).
\end{aligned}
$$

Now, using $\widehat{L}$ one can derive L^{Δ} as shown below:

$$
\begin{aligned}
\int_{\Gamma_0}(\widehat{L}G)(\eta)k(\eta)d\lambda(\eta) &= \int_{\Gamma_0}k(\eta)d\lambda(\eta)\sum_{x^{(i)},y^{(j)}\in\eta} a(x-y)\times \\
&\quad \Big(G(\eta\cup y^{(k)}\setminus x^{(i)}\setminus y^{(j)}) - G(\eta\setminus x^{(i)}) - G(\eta\setminus y^{(j)}) - G(\eta)\Big) \\
&= \int_{\Gamma_0}d\lambda(\eta)\Bigg(\int_{\mathbb{R}^d}dx\sum_{y^{(k)}\in\eta} a(x-y)k((\eta\setminus y^{(k)})\cup y^{(j)}\cup x^{(i)})G(\eta) \\
&\quad -\sum_{y^{(j)}\in\eta}\int_{\mathbb{R}^d}dx a(x-y)k(\eta\cup x^{(i)})G(\eta) \\
&\quad -\sum_{x^{(i)}\in\eta}\int_{\mathbb{R}^d}dy a(x-y)k(\eta\cup y^{(j)})G(\eta) \\
&\quad -\sum_{x^{(i)},y^{(j)}\in\eta} a(x-y)k(\eta)G(\eta)\Bigg);
\end{aligned}
$$

$$
\begin{aligned}
(L^{\Delta}k)(\eta) &= \int_{\mathbb{R}^d}dx\sum_{y^{(k)}\in\eta} a(x-y)k((\eta\setminus y^{(k)})\cup y^{(j)}\cup x^{(i)}) \\
&\quad -\sum_{y^{(j)}\in\eta}\int_{\mathbb{R}^d}dx a(x-y)k(\eta\cup x^{(i)}) \\
&\quad -\sum_{x^{(i)}\in\eta}\int_{\mathbb{R}^d}dy a(x-y)k(\eta\cup y^{(j)}) \\
&\quad -\sum_{x^{(i)},y^{(j)}\in\eta} a(x-y)k(\eta). \qquad (120)
\end{aligned}
$$

In order to find M^{Δ}, one substitutes $\widehat{L}$ into the following equation,

$$
\int_{\Gamma_0}\Big(\widehat{L}e_\lambda(\theta)\Big)(\eta)(\exp^\star u)(\eta)d\lambda(\eta) = \int_{\Gamma_0}\int_{(\mathbb{R}^d)^2}dxdyD_x^{(i)}D_y^{(j)}(\exp^\star u)(\eta)d\lambda(\eta)a(x-y)\times
$$
$$
\times\Big(\theta(y^{(k)}) - \theta(y^{(j)}) - \theta(x^{(i)}) - \theta(y^{(j)})\theta(x^{(i)})\Big)e_\lambda(\theta,\eta),
$$

and keeping only terms with more than one operation D, and substituting, $D_xD_y\exp^* u \longrightarrow D_yu * D_xu$, one obtains:

$$\int_{\Gamma_0} d\lambda(\eta)e_\lambda(\theta,\eta)\times$$
$$\times\int_{(\mathbb{R}^d)^2} dxdy(D_x^{(i)}u * D_y^{(j)}u)(\eta)a(x-y)\left(\theta(y^{(k)})-\theta(y^{(j)})-\theta(x^{(i)})-\theta(y^{(j)})\theta(x^{(i)})\right)=$$
$$=\int_{\Gamma_0} d\lambda(\eta)e_\lambda(\theta,\eta)\Bigg(\int_{\mathbb{R}^d} dx\sum_{y^{(k)}\in\eta}(D_x^{(i)}u * D_y^{(j)}u)(\eta\backslash y^{(k)})$$
$$-\int_{\mathbb{R}^d} dx\sum_{y^{(j)}\in\eta}(D_x^{(i)}u * D_y^{(j)}u)(\eta\backslash y^{(j)})$$
$$-\int_{\mathbb{R}^d} dy\sum_{x^{(i)}\in\eta}(D_x^{(i)}u * D_y^{(j)}u)(\eta\backslash x^{(i)})$$
$$-\sum_{x^{(i)},y^{(j)}\in\eta}(D_x^{(i)}u * D_y^{(j)}u)(\eta\backslash x^{(i)}\backslash y^{(j)})\Bigg)a(x-y)=$$
$$=\int_{\Gamma_0} e_\lambda(\theta)\left(M^\Delta u\right)d\lambda.$$

Thus, M^Δ is obtained,

$$\begin{aligned}\left(M^\Delta u\right)(\eta) &= \int_{\mathbb{R}^d} dx\sum_{y^{(k)}\in\eta}(u(\cdot\cup x^{(i)}) * u(\cdot\cup y^{(j)}))(\eta\backslash y^{(k)})a(x-y)\\ &-\int_{\mathbb{R}^d} dx\sum_{y^{(j)}\in\eta}(u(\cdot\cup x^{(i)}) * u(\cdot\cup y^{(j)}))(\eta\backslash y^{(j)})a(x-y)\\ &-\int_{\mathbb{R}^d} dy\sum_{x^{(i)}\in\eta}(u(\cdot\cup x^{(i)}) * u(\cdot\cup y^{(j)}))(\eta\backslash x^{(i)})a(x-y)\\ &-\sum_{x^{(i)},y^{(j)}\in\eta}(u(\cdot\cup x^{(i)}) * u(\cdot\cup y^{(j)}))(\eta\backslash x^{(i)}\backslash y^{(j)})a(x-y).\end{aligned}\tag{121}$$

In order to find $L^\Delta_{\epsilon,\mathrm{ren}}$ and $M^\Delta_{\epsilon,\mathrm{ren}}$ one should obtain L^Δ_ϵ and M^Δ_ϵ from L^Δ and M^Δ by replacing potentials a by a_ϵ. Then, following the definition, one obtains:

$$\begin{aligned}(S_{\epsilon^{-1}}L^\Delta_\epsilon S_\epsilon k)(\eta) &= \int_{\mathbb{R}^d} dx\sum_{y^{(k)}\in\eta}a(x-y)k((\eta\backslash y^{(k)})\cup y^{(j)}\cup x^{(i)})\\ &-\sum_{y^{(j)}\in\eta}\int_{\mathbb{R}^d} dxa(x-y)k(\eta\cup x^{(i)})\\ &-\sum_{x^{(i)}\in\eta}\int_{\mathbb{R}^d} dya(x-y)k(\eta\cup y^{(j)})\\ &-\epsilon^d\sum_{x^{(i)},y^{(j)}\in\eta}a(x-y)k(\eta)\\ &= \left((A+\epsilon^dB)k\right)(\eta),\end{aligned}$$

where

$$
\begin{aligned}
(Ak)(\eta) &= \int_{\mathbb{R}^d} dx \left(\sum_{y^{(k)}\in\eta} k((\eta\backslash y^{(k)}) \cup y^{(j)} \cup x^{(i)}) - \sum_{y^{(j)}\in\eta} k(\eta \cup x^{(i)}) \right) a(x-y) \\
&\quad - \sum_{x^{(i)}\in\eta} \int_{\mathbb{R}^d} dy a(x-y) k(\eta \cup y^{(j)}); \\
(Bk)(\eta) &= - \sum_{x^{(i)}, y^{(j)}\in\eta} a(x-y) k(\eta).
\end{aligned}
$$

In the same way, one obtains:

$$
\begin{aligned}
(S_{\epsilon^{-1}} M_\epsilon^\Delta S_\epsilon k)(\eta) &= \int_{\mathbb{R}^d} dx \sum_{y^{(k)}\in\eta} (u(\cdot \cup x^{(i)}) * u(\cdot \cup y^{(j)}))(\eta\backslash y^{(k)}) a(x-y) \\
&\quad - \int_{\mathbb{R}^d} dx \sum_{y^{(j)}\in\eta} (u(\cdot \cup x^{(i)}) * u(\cdot \cup y^{(j)}))(\eta\backslash y^{(j)}) a(x-y) \\
&\quad - \int_{\mathbb{R}^d} dy \sum_{x^{(i)}\in\eta} (u(\cdot \cup x^{(i)}) * u(\cdot \cup y^{(j)}))(\eta\backslash x^{(i)}) a(x-y) \\
&\quad - \epsilon^d \sum_{x^{(i)}, y^{(j)}\in\eta} (u(\cdot \cup x^{(i)}) * u(\cdot \cup y^{(j)}))(\eta\backslash x^{(i)}\backslash y^{(j)}) a(x-y) \\
&= ((C + \epsilon^d D)u)(\eta), \qquad (122)
\end{aligned}
$$

where

$$
\begin{aligned}
(Cu)(\eta) &= \int_{\mathbb{R}^d} dx \sum_{y^{(k)}\in\eta} (u(\cdot \cup x^{(i)}) * u(\cdot \cup y^{(j)}))(\eta\backslash y^{(k)}) a(x-y) \\
&\quad - \int_{\mathbb{R}^d} dx \sum_{y^{(j)}\in\eta} (u(\cdot \cup x^{(i)}) * u(\cdot \cup y^{(j)}))(\eta\backslash y^{(j)}) a(x-y) \\
&\quad - \int_{\mathbb{R}^d} dy \sum_{x^{(i)}\in\eta} (u(\cdot \cup x^{(i)}) * u(\cdot \cup y^{(j)}))(\eta\backslash x^{(i)}) a(x-y); \\
(Du)(\eta) &= - \sum_{x^{(i)}, y^{(j)}\in\eta} (u(\cdot \cup x^{(i)}) * u(\cdot \cup y^{(j)}))(\eta\backslash x^{(i)}\backslash y^{(j)}) a(x-y).
\end{aligned}
$$

Thus, applying perturbation expansion in the limit $\epsilon^d \to 0$, one obtains:

$$
\begin{aligned}
(Av + Cv)(\eta) &= \mathbb{1}_{\eta=\{x^{(m)}\}} \Big((\delta_{mk} - \delta_{mj}) q_j(x) \int_{\mathbb{R}^d} dy q_i(y) a(x-y) \\
&\quad - \delta_{mi} q_i(x) \int_{\mathbb{R}^d} dy q_j(y) a(x-y) \Big). \qquad (123)
\end{aligned}
$$

In order to obtain equations for corrections w, one uses

$$
\frac{\partial}{\partial t} w(t,\eta) = (Aw + Bv + Dv + C(v,w) + C(w,v))(\eta),
$$

where

$$
\begin{aligned}
(Aw)(\eta) &= \int_{\mathbb{R}^d} dx \left(\sum_{y^{(k)} \in \eta} w((\eta \backslash y^{(k)}) \cup y^{(j)} \cup x^{(i)}) - \sum_{y^{(j)} \in \eta} w(\eta \cup x^{(i)}) \right) a(x-y) \\
&\quad - \sum_{x^{(i)} \in \eta} \int_{\mathbb{R}^d} dy a(x-y) w(\eta \cup y^{(j)}) \\
&= \int_{\mathbb{R}^d} dy \sum_{x^{(k)} \in \eta} a(x-y) w((\eta \backslash x^{(k)}) \cup x^{(j)} \cup y^{(i)}) \\
&\quad - \int_{\mathbb{R}^d} dy \sum_{x^{(j)} \in \eta} a(x-y) w(\eta \cup y^{(i)}) \\
&\quad - \int_{\mathbb{R}^d} dy \sum_{x^{(i)} \in \eta} a(x-y) w(\eta \cup y^{(j)}) \\
&= \mathbb{1}_{\eta = \{x^{(m)}\}} \int_{\mathbb{R}^d} dy a(y-x) \left(\delta_{mk} g_{ji}(x^{(j)}, y^{(i)}) - \delta_{mj} g_{ji}(x^{(j)}, y^{(i)}) - \delta_{mi} g_{ij}(x^{(i)}, y^{(j)}) \right);
\end{aligned}
$$

$$
\begin{aligned}
(Bv)(\eta) &= - \sum_{x^{(i)}, y^{(j)} \in \eta} a(x-y) v(\eta) \\
&= \mathbb{1}_{\eta = \{x^{(m)}\}} \left(- \sum_{x^{(i)}, y^{(j)} \in \eta} a(x-y) v(\eta) \right) = 0;
\end{aligned}
$$

$$
\begin{aligned}
(Dv)(\eta) &= - \sum_{x^{(i)}, y^{(j)} \in \eta} (v(\cdot \cup x^{(i)}) * v(\cdot \cup y^{(j)}))(\eta \backslash x^{(i)} \backslash y^{(j)}) a(x-y) \\
&= - \sum_{x^{(i)}, y^{(j)} \in \eta} v(x^{(i)}) v(\eta \backslash x^{(i)}) a(x-y) \\
&= \mathbb{1}_{\eta = \{x_1^{(m)}, x_2^{(n)}\}} \left([-\delta_{mi} \delta_{nj} q_i(x_1) q_j(x_2) - \delta_{mj} \delta_{ni} q_i(x_2) q_j(x_1)] a(x_1 - x_2) \right);
\end{aligned}
$$

$$
\begin{aligned}
C(v,w)(\eta) &= \int_{\mathbb{R}^d} dx \sum_{y^{(k)}\in\eta} (v(\cdot\cup x^{(i)}) * w(\cdot\cup y^{(j)}))(\eta\backslash y^{(k)})a(x-y) \\
&\quad - \int_{\mathbb{R}^d} dx \sum_{y^{(j)}\in\eta} (v(\cdot\cup x^{(i)}) * w(\cdot\cup y^{(j)}))(\eta\backslash y^{(j)})a(x-y) \\
&\quad - \int_{\mathbb{R}^d} dy \sum_{x^{(i)}\in\eta} (v(\cdot\cup x^{(i)}) * w(\cdot\cup y^{(j)}))(\eta\backslash x^{(i)})a(x-y) \\
&= \int_{\mathbb{R}^d} dx \sum_{y^{(k)}\in\eta} v(x^{(i)})w((\eta\backslash y^{(k)})\cup y^{(j)})a(x-y) \\
&\quad - \int_{\mathbb{R}^d} dx \sum_{y^{(j)}\in\eta} v(x^{(i)})w(\eta)a(x-y) \\
&\quad - \int_{\mathbb{R}^d} dy \sum_{x^{(i)}\in\eta} v(x^{(i)})w((\eta\backslash x^{(i)})\cup y^{(j)})a(x-y) \\
&= \int_{\mathbb{R}^d} dy \sum_{x^{(k)}\in\eta} v(y^{(i)})w((\eta\backslash x^{(k)})\cup x^{(j)})a(x-y) \\
&\quad - \int_{\mathbb{R}^d} dy \sum_{x^{(j)}\in\eta} v(y^{(i)})w(\eta)a(x-y) \\
&\quad - \int_{\mathbb{R}^d} dy \sum_{x^{(i)}\in\eta} v(x^{(i)})w((\eta\backslash x^{(i)})\cup y^{(j)})a(x-y) \\
&= \mathbb{1}_{\eta=\{x^{(m)}\}} \times \Bigg(\delta_{mk} \int_{\mathbb{R}^d} dy q_i(y^{(i)})p_j(x^{(j)})a(x-y) \\
&\quad -\delta_{mj} \int_{\mathbb{R}^d} dy q_i(y^{(i)})p_j(x^{(j)})a(x-y) \\
&\quad -\delta_{mi} \int_{\mathbb{R}^d} dy q_i(x^{(i)})p_j(y^{(j)})a(x-y)\Bigg) \\
&+ \mathbb{1}_{\eta=\{x_1^{(m)},x_2^{(n)}\}} \times \Bigg(+\delta_{mk}\delta_{nk} \int_{\mathbb{R}^d} dy q_i(y)[g_{jk}(x_1,x_2)a(x_1-y) + g_{kj}(x_1,x_2)a(x_2-y)] \\
&\quad +\delta_{m\neq k}\delta_{nk} \int_{\mathbb{R}^d} dy q_i(y)g_{mj}(x_1,x_2)a(x_2-y) \\
&\quad +\delta_{mk}\delta_{n\neq k} \int_{\mathbb{R}^d} dy q_i(y)g_{jn}(x_1,x_2)a(x_1-y) \\
&\quad - \int_{\mathbb{R}^d} dy q_i(y)\delta_{mj}\delta_{nj}g_{jj}(x_1,x_2)[a(x_1-y)+a(x_2-y)] \\
&\qquad - \int_{\mathbb{R}^d} dy q_i(y)[\delta_{m\neq j}\delta_{nj}g_{mj}(x_1,x_2)a(x_2-y) + \delta_{mj}\delta_{n\neq j}g_{jn}(x_1,x_2)a(x_1-y)] \\
&\quad -\delta_{mi}\delta_{ni} \int_{\mathbb{R}^d} dy[q_i(x_1)g_{ji}(y^{(j)},x_2)a(x_1-y) + q_i(x_2)g_{ij}(x_1,y^{(j)})a(x_2-y)] \\
&\quad -\delta_{m\neq i}\delta_{ni} \int_{\mathbb{R}^d} dy q_i(x_2)g_{mj}(x_1,y^{(j)})a(x_2-y) \\
&\quad -\delta_{mi}\delta_{n\neq i} \int_{\mathbb{R}^d} dy q_i(x_1)g_{jn}(y^{(j)},x_2)a(x_1-y)\Bigg);
\end{aligned}
$$

$$
\begin{aligned}
C(w,v)(\eta) &= \int_{\mathbb{R}^d} dx \sum_{y^{(k)}\in\eta} (w(\cdot\cup x^{(i)}) * v(\cdot\cup y^{(j)}))(\eta\backslash y^{(k)})a(x-y) \\
&\quad - \int_{\mathbb{R}^d} dx \sum_{y^{(j)}\in\eta} (w(\cdot\cup x^{(i)}) * v(\cdot\cup y^{(j)}))(\eta\backslash y^{(j)})a(x-y) \\
&\quad - \int_{\mathbb{R}^d} dy \sum_{x^{(i)}\in\eta} (w(\cdot\cup x^{(i)}) * v(\cdot\cup y^{(j)}))(\eta\backslash x^{(i)})a(x-y) \\
&= \int_{\mathbb{R}^d} dx \sum_{y^{(k)}\in\eta} w((\eta\backslash y^{(k)})\cup x^{(i)})v(y^{(j)})a(x-y) \\
&\quad - \int_{\mathbb{R}^d} dx \sum_{y^{(j)}\in\eta} w((\eta\backslash y^{(j)})\cup x^{(i)})v(y^{(j)})a(x-y) \\
&\quad - \int_{\mathbb{R}^d} dy \sum_{x^{(i)}\in\eta} w(\eta)v(y^{(j)})a(x-y) \\
&= \int_{\mathbb{R}^d} dy \sum_{x^{(k)}\in\eta} w((\eta\backslash x^{(k)})\cup y^{(i)})v(x^{(j)})a(x-y) \\
&\quad - \int_{\mathbb{R}^d} dy \sum_{x^{(j)}\in\eta} w((\eta\backslash x^{(j)})\cup y^{(i)})v(x^{(j)})a(x-y) \\
&\quad - \int_{\mathbb{R}^d} dy \sum_{x^{(i)}\in\eta} w(\eta)v(y^{(j)})a(x-y),
\end{aligned}
$$

$$
\begin{aligned}
C(w,v)(\eta) &= \mathbb{1}_{\eta=\{x^{(m)}\}} \times \Big(\\
&\quad +\delta_{mk} \int_{\mathbb{R}^d} dy p_i(y^{(i)})q_j(x^{(j)})a(x-y) \\
&\quad -\delta_{mj} \int_{\mathbb{R}^d} dy p_i(y^{(i)})q_j(x^{(j)})a(x-y) \\
&\quad -\delta_{mi} \int_{\mathbb{R}^d} dy p_i(x)q_j(y^{(j)})a(x-y)\Big) + \\
&+ \mathbb{1}_{\eta=\{x_1^{(m)},x_2^{(n)}\}} \times \Big(\\
&\quad +\delta_{mk}\delta_{nk} \int_{\mathbb{R}^d} dy[g_{ik}(y^{(i)},x_2)q_j(x_1)a(x_1-y) + g_{ki}(x_1,y^{(i)})q_j(x_2)a(x_2-y)] \\
&\quad +\delta_{m\neq k}\delta_{nk} \int_{\mathbb{R}^d} dy g_{mi}(x_1,y^{(i)})q_j(x_2)a(x_2-y) \\
&\quad +\delta_{mk}\delta_{n\neq k} \int_{\mathbb{R}^d} dy g_{in}(y^{(i)},x_2)q_j(x_1)a(x_1-y) \\
&\quad -\delta_{mj}\delta_{nj} \int_{\mathbb{R}^d} dy[g_{ij}(y^{(i)},x_2)q_j(x_1)a(x_1-y) + g_{ji}(x_1,y^{(i)})q_j(x_2)a(x_2-y)] \\
&\quad -\delta_{m\neq j}\delta_{nj} \int_{\mathbb{R}^d} dy g_{mi}(x_1,y^{(i)})q_j(x_2)a(x_2-y) \\
&\quad -\delta_{mj}\delta_{n\neq j} \int_{\mathbb{R}^d} dy g_{in}(y^{(i)},x_2)q_j(x_1)a(x_1-y) \\
&\quad\quad -\delta_{mi}\delta_{ni}g_{ii}(x_1,x_2) \int_{\mathbb{R}^d} dy q_j(y^{(j)})[a(x_1-y)+a(x_2-y)] \\
&\quad\quad -\delta_{m\neq i}\delta_{ni}g_{mi}(x_1,x_2) \int_{\mathbb{R}^d} dy q_j(y^{(j)})a(x_2-y) \\
&\quad\quad -\delta_{mi}\delta_{n\neq i}g_{in}(x_1,x_2) \int_{\mathbb{R}^d} dy q_j(y^{(j)})a(x_1-y)\Big).
\end{aligned}
$$

As a result, one finds:

$$\begin{aligned}
H^{\mathrm{CTC}}_{q_m}(x) &= \mathbb{1}_{\eta=\{x^{(m)}\}}\Big((\delta_{mk}-\delta_{mj})q_j(x)\int_{\mathbb{R}^d}dy q_i(y)a(x-y) \\
&\quad -\delta_{mi}q_i(x)\int_{\mathbb{R}^d}dy q_j(y)a(x-y)\Big); \\
H^{\mathrm{CTC}}_{p_m}(x) &= \int_{\mathbb{R}^d}dy a(y-x)\left(\delta_{mk}g_{ji}(x^{(j)},y^{(i)})-\delta_{mj}g_{ji}(x^{(j)},y^{(i)})-\delta_{mi}g_{ij}(x^{(i)},y^{(j)})\right) \\
&\quad +\Big(\delta_{mk}\int_{\mathbb{R}^d}dy q_i(y^{(i)})p_j(x^{(j)})a(x-y) \\
&\quad -\delta_{mj}\int_{\mathbb{R}^d}dy q_i(y^{(i)})p_j(x^{(j)})a(x-y) \\
&\quad -\delta_{mi}\int_{\mathbb{R}^d}dy q_i(x^{(i)})p_j(y^{(j)})a(x-y)\Big) \\
&\quad +\Big(\delta_{mk}\int_{\mathbb{R}^d}dy p_i(y^{(i)})q_j(x^{(j)})a(x-y) \\
&\quad -\delta_{mj}\int_{\mathbb{R}^d}dy p_i(y^{(i)})q_j(x^{(j)})a(x-y) \\
&\quad -\delta_{mi}\int_{\mathbb{R}^d}dy p_i(x)q_j(y^{(j)})a(x-y)\Big);
\end{aligned} \tag{124}$$

$$
\begin{aligned}
H^{\mathrm{CTC}}_{g_{mn}}(x_1,x_2) \;\; = \;\; & [-\delta_{mi}\delta_{nj}q_i(x_1)q_j(x_2) - \delta_{mj}\delta_{ni}q_i(x_2)q_j(x_1)]a(x_1-x_2) \\
+ \;\; & \Big(+\delta_{mk}\delta_{nk}\int_{\mathbb{R}^d} dyq_i(y)[g_{jk}(x_1,x_2)a(x_1-y) + g_{kj}(x_1,x_2)a(x_2-y)] \\
& +\delta_{m\neq k}\delta_{nk}\int_{\mathbb{R}^d} dyq_i(y)g_{mj}(x_1,x_2)a(x_2-y) \\
& +\delta_{mk}\delta_{n\neq k}\int_{\mathbb{R}^d} dyq_i(y)g_{jn}(x_1,x_2)a(x_1-y) \\
& -\int_{\mathbb{R}^d} dyq_i(y)\delta_{mj}\delta_{nj}g_{jj}(x_1,x_2)[a(x_1-y)+a(x_2-y)] \\
& \qquad -\int_{\mathbb{R}^d} dyq_i(y)[\delta_{m\neq j}\delta_{nj}g_{mj}(x_1,x_2)a(x_2-y) + \delta_{mj}\delta_{n\neq j}g_{jn}(x_1,x_2)a(x_1-y)] \\
& -\delta_{mi}\delta_{ni}\int_{\mathbb{R}^d} dy[q_i(x_1)g_{ji}(y,x_2)a(x_1-y) + q_i(x_2)g_{ij}(x_1,y)a(x_2-y)] \\
& -\delta_{m\neq i}\delta_{ni}\int_{\mathbb{R}^d} dyq_i(x_2)g_{mj}(x_1,y)a(x_2-y) \\
& -\delta_{mi}\delta_{n\neq i}\int_{\mathbb{R}^d} dyq_i(x_1)g_{jn}(y,x_2)a(x_1-y)\Big) \\
+ \;\; & \Big(+\delta_{mk}\delta_{nk}\int_{\mathbb{R}^d} dy[g_{ik}(y,x_2)q_j(x_1)a(x_1-y) + g_{ki}(x_1,y)q_j(x_2)a(x_2-y)] \\
& +\delta_{m\neq k}\delta_{nk}\int_{\mathbb{R}^d} dyg_{mi}(x_1,y)q_j(x_2)a(x_2-y) \\
& +\delta_{mk}\delta_{n\neq k}\int_{\mathbb{R}^d} dyg_{in}(y,x_2)q_j(x_1)a(x_1-y) \\
& -\delta_{mj}\delta_{nj}\int_{\mathbb{R}^d} dy[g_{ij}(y,x_2)q_j(x_1)a(x_1-y) + g_{ji}(x_1,y)q_j(x_2)a(x_2-y)] \\
& -\delta_{m\neq j}\delta_{nj}\int_{\mathbb{R}^d} dyg_{mi}(x_1,y)q_j(x_2)a(x_2-y) \\
& -\delta_{mj}\delta_{n\neq j}\int_{\mathbb{R}^d} dyg_{in}(y,x_2)q_j(x_1)a(x_1-y) \\
& \quad -\delta_{mi}\delta_{ni}g_{ii}(x_1,x_2)\int_{\mathbb{R}^d} dyq_j(y)[a(x_1-y)+a(x_2-y)] \\
& \quad -\delta_{m\neq i}\delta_{ni}g_{mi}(x_1,x_2)\int_{\mathbb{R}^d} dyq_j(y)a(x_2-y) \\
& \quad -\delta_{mi}\delta_{n\neq i}g_{in}(x_1,x_2)\int_{\mathbb{R}^d} dyq_j(y)a(x_1-y)\Big).
\end{aligned}
$$

In the translationally invariant case, using $A = \int_{\mathbb{R}^d} a(x)dx$, one obtains:

$$
\begin{aligned}
H_{q_m}^{\text{CTC}} &= (\delta_{mk} - \delta_{mj} - \delta_{mi})\, q_i q_j A; \\
H_{p_m}^{\text{CTC}} &= (\delta_{mk} - \delta_{mj} - \delta_{mi}) \left(\int_{\mathbb{R}^d} dy a(y) g_{ji}(y) + (q_i p_j + q_j p_i) A \right); \\
H_{g_{mn}}^{\text{CTC}}(x) &= +\delta_{mk}\delta_{nk} \left(2 q_i g_{jk}(x) A + 2 q_j \int_{\mathbb{R}^d} dy g_{ik}(y) a(x-y) \right) \\
&\quad +\delta_{m\neq k}\delta_{nk} \left(q_i g_{mj}(x) A + q_j \int_{\mathbb{R}^d} dy g_{mi}(y) a(x-y) \right) \\
&\quad +\delta_{mk}\delta_{n\neq k} \left(q_i g_{jn}(x) A + q_j \int_{\mathbb{R}^d} dy g_{in}(y) a(x-y) \right) \\
&\quad -(\delta_{mi}\delta_{nj} + \delta_{mj}\delta_{ni}) q_i q_j a(x) \\
&\quad +\delta_{mj}\delta_{nj} \left(-2 q_i g_{jj}(x) A - 2 q_j \int_{\mathbb{R}^d} dy g_{ij}(y) a(x-y) \right) \\
&\quad +\delta_{m\neq j}\delta_{nj} \left(-q_i g_{mj}(x) A - q_j \int_{\mathbb{R}^d} dy g_{mi}(y) a(x-y) \right) \\
&\quad +\delta_{mj}\delta_{n\neq j} \left(-q_i g_{jn}(x) A - q_j \int_{\mathbb{R}^d} dy g_{in}(y) a(x-y) \right) \\
&\quad +\delta_{mi}\delta_{ni} \left(-2 q_j g_{ii}(x) A - 2 q_i \int_{\mathbb{R}^d} dy g_{ji}(y) a(x-y) \right) \\
&\quad +\delta_{m\neq i}\delta_{ni} \left(-q_j g_{mi}(x) A - q_i \int_{\mathbb{R}^d} dy g_{mj}(y) a(x-y) \right) \\
&\quad +\delta_{mi}\delta_{n\neq i} \left(-q_j g_{in}(x) A - q_i \int_{\mathbb{R}^d} dy g_{jn}(y) a(x-y) \right).
\end{aligned} \tag{125}
$$

1.4 Derivations for a general process

In this section, we derive a hierarchy of equations describing the evolution of correlation functions and cumulants for a general process from a wide class of stochastic spatial point processes. The perturbation expansion in the approximation of long-ranged interactions is applied, and functions H_q, H_p and H_g for a general process are derived. The derivation uses the same formalism and the same scheme as described in sections 1.1 and 1.2.

1.4.1 Formulation of a general process

Consider that all points (or particles, or individuals) in the system consists of different species, where species are enumerated by numbers $i_1, ..., i_P$, $j_1, ..., j_R$, and $k_1, ..., k_C$. As will be explained below, we distinguish 3 groups of species denoted by letters i, j, k, with P, R, C species in each group respectively. The locally finite configuration γ consists of configurations corresponding to different species:

$$\gamma = (\gamma_{i_1}, ..., \gamma_{i_P}, \gamma_{j_1}, ..., \gamma_{j_R}, \gamma_{k_1}, ..., \gamma_{k_C}). \tag{126}$$

Consequently, the point characterised by species m and a coordinate x_n is denoted as $x_n^{(m)}$. For example, the point of a species i_m at a coordinate x_{j_n} is denoted as $x_{j_n}^{(i_m)}$, which is defined as:

$$x_{j_n}^{(i_m)} := (\xi_{i_1}, ..., \xi_{i_n}, ..., \xi_{k_C}) \subset \gamma, \qquad \xi_l = \begin{cases} \varnothing, & l \neq i_m; \\ \{x_{j_n}\}, & l = i_m. \end{cases} \tag{127}$$

Hereafter, for the sake of brevity, the condition $x_{j_n} \in \gamma_{i_m}$ and the condition $x_{j_n}^{(i_m)} \in \gamma$ are equivalent.

Consider a general process, during which the observable $F(\gamma)$ changes into the following observable:

$$F(\gamma \backslash x_{j_1}^{(j_1)} \backslash ... \backslash x_{j_R}^{(j_R)} \cup x_{i_1}^{(i_1)} \cup ... \cup x_{i_P}^{(i_P)}). \tag{128}$$

Thus, during a single event in this process points $x_{i_1}^{(i_1)}, ... x_{i_P}^{(i_P)}$ are added, and points $x_{j_1}^{(j_1)}, ..., x_{j_R}^{(j_R)}$ are deleted. In general, some indices and some coordinates of these points may be the same. Nevertheless, we denote all these indices and coordinates uniquely, and specify later which of them equal to each other.

All points which are added to the system, i.e. points $x_{i_1}^{(i_1)}, ... x_{i_P}^{(i_P)}$ in (128), are said to belong to the group named "Products". For simplicity, different species in this group are denoted as i_1, i_2, etc, and coordinates are denoted as x_{i_1}, x_{i_2}, etc. The group is characterised by the following list of pairs (index,coordinate),

$$\mathbf{P} = ((i_1, x_{i_1}), (i_2, x_{i_2}), ..., (i_P, x_{i_P})). \tag{129}$$

All points which are removed from the system, i.e. points $x_{j_1}^{(j_1)}, ..., x_{j_R}^{(j_R)}$ in (128), are said to belong to the group named "Reactants". Different species in this group are denoted as j_1, j_2 etc, and coordinates are denoted as x_{j_1}, x_{j_2}, etc. The group is characterised by the following list:

$$\mathbf{R} = ((j_1, x_{j_1}), (j_2, x_{j_2}), ..., (j_R, x_{j_R})). \tag{130}$$

Also, there are points in the system which influence the rate of the process but which are not removed or added. These points are said to belong to the group named "Catalysts". Different species in this group are denoted as k_1, k_2 etc, and coordinates are denoted as x_{k_1}, x_{k_2}, etc. The group is characterised by the following list:

$$\mathbf{C} = ((k_1, x_{k_1}), (k_2, x_{k_2}), ..., (k_C, x_{k_C})). \tag{131}$$

We set, that all coordinates belong to the same space $\mathbb{R}^d$, i.e. $x_{i_m} \in \mathbb{R}^d$, $x_{j_m} \in \mathbb{R}^d$, $x_{k_m} \in \mathbb{R}^d$ for any integer m.

Let $r(\mathbf{P}, \mathbf{R}, \mathbf{C})$ be a function which depends on all coordinates and all indices used in (129)-(131). The function r specifies coordinates and indices which are the same in $\mathbf{P}$, $\mathbf{R}$, $\mathbf{C}$. Moreover, we consider that the dependence of $r(\mathbf{P}, \mathbf{R}, \mathbf{C})$ on coordinates is such that only distances between points can be arguments of $r(\mathbf{P}, \mathbf{R}, \mathbf{C})$. Therefore, the function $r(\mathbf{P}, \mathbf{C}, \mathbf{R})$ is invariant with respect to any distance-preserving transformations of coordinates, e.g. translations, rotations, inversions.

Using notations introduced above, let L^{PRC} be the Markov operator for a general process defined as:

$$\begin{aligned} \left(L^{\text{PRC}} F\right)(\gamma) \quad = \quad & \int_{(\mathbb{R}^d)^P} dx_{i_1} ... dx_{i_P} \sum_{x_{j_1}^{(j_1)}, ..., x_{j_R}^{(j_R)} \in \gamma} \; \sum_{x_{k_1}^{(k_1)}, ..., x_{k_C}^{(k_C)} \in \gamma} r(\mathbf{P}, \mathbf{R}, \mathbf{C}) \times \\ & \times \Big(F(\gamma \backslash x_{j_1}^{(j_1)} \backslash ... \backslash x_{j_R}^{(j_R)} \cup x_{i_1}^{(i_1)} \cup ... \cup x_{i_P}^{(i_P)}) - F(\gamma) \Big). \end{aligned} \tag{132}$$

In these notations, all examples of processes studied in the previous section are shown in Supplementary Table 2 and Supplementary Table 1.

$\mathbf{P}, \mathbf{R}, \mathbf{C}$	$(LF)(\gamma)$	Process	Function $r(\mathbf{P}, \mathbf{R}, \mathbf{C})$	Verbal model specification
$\mathbf{P} = \{i, x_1\}$; $\mathbf{R} = \varnothing$; $\mathbf{C} = \varnothing$;	$\int_{\mathbb{R}^d} dx_1 \Big(F(\gamma \cup x_1^{(i)}) - F(\gamma) \Big) r(\mathbf{P}, \mathbf{R}, \mathbf{C})$	IM	r	"Immigration". Individuals of type i appear spontaneously at rate r.
$\mathbf{P} = \{i, x_1\}$; $\mathbf{R} = \varnothing$; $\mathbf{C} = \{k, x_2\}$;	$\int_{\mathbb{R}^d} dx_1 \sum_{x_2^{(k)} \in \gamma} \Big(F(\gamma \cup x_1^{(i)}) - F(\gamma) \Big) r(\mathbf{P}, \mathbf{R}, \mathbf{C})$	B	$\mathbf{1}_{k=i} a(x_1 - x_2)$	"Birth". Existing individuals of type i produce new individuals of type i with kernel a.
		BT	$a(x_1 - x_2)$	"Birth to another type". Existing individuals of type k produce new individuals of type i with kernel a.
$\mathbf{P} = \{i, x_1\}$; $\mathbf{R} = \varnothing$; $\mathbf{C} = \{\{k, x_2\}, \{k', x_3\}\}$;	$\int_{\mathbb{R}^d} dx_1 \sum_{x_2^{(k)} \in \gamma} \sum_{x_3^{(k')} \in \gamma} \Big(F(\gamma \cup x_1^{(i)}) - F(\gamma) \Big) \times$ $\times\, r(\mathbf{P}, \mathbf{R}, \mathbf{C})$	BF	$\mathbb{1}_{k=i} a(x_3 - x_2) b(x_2 - x_1)$	"Birth by facilitation". Type i individuals, influenced by type k' individuals ($k' \neq i$) with kernel a, produce offspring of type i with kernel b.
		BTF	$a(x_3 - x_2) b(x_2 - x_1)$	"Birth to another type by facilitation". Type k individuals, influenced by type k' individuals ($k' \neq k$) with kernel a, produce offspring of type i with kernel b.
$\mathbf{P} = \varnothing$; $\mathbf{R} = \{j, x_1\}$; $\mathbf{C} = \varnothing$;	$\sum_{x_1^{(j)} \in \gamma} \Big(F(\gamma \backslash x_1^{(j)}) - F(\gamma) \Big) r(\mathbf{P}, \mathbf{R}, \mathbf{C})$	D	r	"Density independent death". Individuals of type j die spontaneously with rate r.
$\mathbf{P} = \varnothing$; $\mathbf{R} = \{j, x_1\}$; $\mathbf{C} = \{k, x_2\}$;	$\sum_{x_1^{(j)} \in \gamma} \sum_{x_2^{(k)} \in \gamma} \Big(F(\gamma \backslash x_1^{(j)}) - F(\gamma) \Big) r(\mathbf{P}, \mathbf{R}, \mathbf{C})$	C	$\mathbb{1}_{k=j} a(x_1 - x_2)$	"Death by competition". Type j individuals induce death in type j individuals with kernel a.
		DE	$a(x_1 - x_2)$	"Death by external factor". Type k individuals induce death in type j individuals with kernel a.

Supplementary Table 1: Selected processes discussed in section 1.3 shown as a particular case of a general process (132).

$\mathbf{P}, \mathbf{R}, \mathbf{C}$	$(LF)(\gamma)$	Process	Function $r(\mathbf{P}, \mathbf{R}, \mathbf{C})$	Verbal model specification
$\mathbf{P} = \{i, x_1\}$; $\mathbf{R} = \{j, x_2\}$; $\mathbf{C} = \varnothing$;	$\int_{\mathbb{R}^d} dx_1 \sum_{x_2^{(j)} \in \gamma} \Big(F(\gamma \backslash x_2^{(j)} \cup x_1^{(i)}) - F(\gamma) \Big) \times$ $\times\, r(\mathbf{P}, \mathbf{R}, \mathbf{C})$	CT	$r\delta(x_1 - x_2)$	"Change in type". Individuals of type j change spontaneously into type i ($i \neq j$) at rate r.
		J	$\mathbb{1}_{j=i} a(x_1 - x_2)$	"Jump". Type i individuals change their positions by a jump with kernel a.
		JCT	$a(x_1 - x_2)$	"Jump and change of type". Type j individuals change their position by a jump with kernel a, and simultaneously change into type i individuals.
$\mathbf{P} = \{i, x_1\}$; $\mathbf{R} = \{j, x_2\}$; $\mathbf{C} = \{k, x_3\}$;	$\int_{\mathbb{R}^d} dx_1 \sum_{x_2^{(j)} \in \gamma} \sum_{x_3^{(k)} \in \gamma} \Big(F(\gamma \backslash x_2^{(j)} \cup x_1^{(i)}) - F(\gamma) \Big) \times$ $\times\, r(\mathbf{P}, \mathbf{R}, \mathbf{C})$	I	$\mathbb{1}_{k=i} \delta(x_1 - x_2) a(x_2 - x_3)$	"Infection". Individuals of type i change individuals of type j ($j \neq k$) into type i individuals with kernel a.
		BC	$\mathbb{1}_{k=i} a(x_2 - x_3) b(x_1 - x_3)$	"Birth by consumption". Type i individuals induce death of type j ($j \neq i$) individuals with kernel a, and simultaneously give birth to type i individuals with kernel b.
		BTC	$a(x_2 - x_3) b(x_1 - x_3)$	"Birth to another type by consumption". Type k individuals induce death of type j ($j \neq k$) individuals with kernel a, and simultaneously give birth to type i individuals with kernel b.
$\mathbf{P} = \{i, x_1\}$; $\mathbf{R} = \{\{j, x_2\}, \{j', x_3\}\}$; $\mathbf{C} = \varnothing$;	$\int_{\mathbb{R}^d} dx_1 \sum_{x_2^{(j)} \in \gamma} \sum_{x_3^{(j')} \in \gamma} r(\mathbf{P}, \mathbf{R}, \mathbf{C}) \times$ $\times \Big(F(\gamma \backslash x_2^{(j)} \backslash x_3^{(j')} \cup x_1^{(i)}) - F(\gamma) \Big) \times$	CTC	$\delta(x_1 - x_2) a(x_2 - x_3)$	"Change in type by consumption". Type j individuals change to type i ($i \neq j$) individuals, and simultaneously induce death of type j' individuals with kernel a.

Supplementary Table 2: Selected processes discussed in section 1.3 shown as a particular case of a general process (132).

1.4.2 Derivation of the operator $\widehat{L}$

The definition of the operator $\widehat{L}$ is:

$$(\widehat{L}G)(\eta) := (K^{-1}L^{\mathrm{PRC}}KG)(\eta). \tag{133}$$

Using $F = KG$, where K is defined in (38) as

$$(KG)(\gamma) := \sum_{\eta \Subset \gamma} G(\eta),$$

the difference of two F functions in Eq.(132) becomes:

$$\begin{aligned} KG(\gamma \backslash x_{j_1}^{(j_1)} \backslash ... \backslash x_{j_R}^{(j_R)} \cup x_{i_1}^{(i_1)} \cup ... \cup x_{i_P}^{(i_P)}) - KG(\gamma) = \\ = \sum_{\eta \Subset \gamma \backslash x_{j_1}^{(j_1)} \backslash ... \backslash x_{j_R}^{(j_R)} \cup x_{i_1}^{(i_1)} \cup ... \cup x_{i_P}^{(i_P)}} G(\eta) - \sum_{\eta \Subset \gamma} G(\eta). \end{aligned} \tag{134}$$

It is convenient to rewrite this expression in terms of D operators, here we recall their definitions (39):

$$(D_x^{(i)} u)(\eta) := u(\eta \cup x^{(i)}).$$

One can show that:

$$\begin{aligned} \sum_{\eta \Subset \gamma} G(\eta) &= \sum_{\eta \Subset \gamma \backslash x} G(\eta) + \sum_{\eta \Subset \gamma \backslash x} G(\eta \cup x) \\ &= \sum_{\eta \Subset \gamma \backslash x} (1 + D_x)\, G(\eta) \\ &= \sum_{\eta \Subset \gamma \backslash x \backslash y} (1 + D_x)\,(1 + D_y) G(\eta) = \sum_{\eta \Subset \gamma \backslash x \backslash y} \prod_{i = x,y} (1 + D_i)\, G(\eta), \end{aligned} \tag{135}$$

where $\prod_{i \in \varnothing} = 1$. Therefore, using the following representation,

$$\begin{aligned} \sum_{\eta \Subset \gamma \backslash x_{j_1}^{(j_1)} \backslash ... \backslash x_{j_R}^{(j_R)} \cup x_{i_1}^{(i_1)} \cup ... \cup x_{i_P}^{(i_P)}} G(\eta) &= \sum_{\eta \Subset \gamma \backslash x_{j_1}^{(j_1)} \backslash ... \backslash x_{j_R}^{(j_R)}} \prod_{i=i_1}^{i_P} \left(1 + D_{x_i}^{(i)}\right) G(\eta); \\ \sum_{\eta \Subset \gamma} G(\eta) &= \sum_{\eta \Subset \gamma \backslash x_{j_1}^{(j_1)} \backslash ... \backslash x_{j_R}^{(j_R)}} \prod_{j=j_1}^{j_R} (1 + D_{x_j}^{(j)}) G(\eta), \end{aligned} \tag{136}$$

the equation (134) becomes:

$$\begin{aligned} KG(\gamma \backslash x_{j_1}^{(j_1)} \backslash ... \backslash x_{j_R}^{(j_R)} \cup x_{i_1}^{(i_1)} \cup ... \cup x_{i_P}^{(i_P)}) - KG(\gamma) = \\ = \sum_{\eta \Subset \gamma \backslash x_{j_1}^{(j_1)} \backslash ... \backslash x_{j_R}^{(j_R)} \cup x_{i_1}^{(i_1)} \cup ... \cup x_{i_P}^{(i_P)}} G(\eta) - \sum_{\eta \Subset \gamma} G(\eta) = \\ = \sum_{\eta \Subset \gamma \backslash x_{j_1}^{(j_1)} \backslash ... \backslash x_{j_R}^{(j_R)}} \left(\prod_{i=i_1}^{i_P} \left(1 + D_{x_i}^{(i)}\right) - \prod_{j=j_1}^{j_R} (1 + D_{x_j}^{(j)}) \right) G(\eta) = \\ = \left(K \left[\prod_{i=i_1}^{i_P} (1 + D_{x_i}^{(i)}) - \prod_{j=j_1}^{j_R} (1 + D_{x_j}^{(j)}) \right] G \right) (\gamma \backslash x_{j_1}^{(j_1)} \backslash ... \backslash x_{j_R}^{(j_R)}). \end{aligned} \tag{137}$$

Thus, the expression for a Markov operator (132), using $F = KG$, becomes:

$$\begin{aligned}\left(L^{\mathrm{PRC}}KG\right)(\gamma) &= \int_{(\mathbb{R}^d)^P} dx_{i_1}...dx_{i_P} \sum_{x^{(j_1)}_{j_1},...,x^{(j_R)}_{j_R}\in\gamma} \sum_{x^{(k_1)}_{k_1},...,x^{(k_C)}_{k_C}\in\gamma} r(\mathbf{P},\mathbf{R},\mathbf{C}) \times \\ &\times \left(K\left[\prod_{i=i_1}^{i_P}(1+D^{(i)}_{x_i}) - \prod_{j=j_1}^{j_R}(1+D^{(j)}_{x_j})\right]G\right)(\gamma\backslash x^{(j_1)}_{j_1}\backslash...\backslash x^{(j_R)}_{j_R}). \end{aligned} \tag{138}$$

For the sake of brevity, one can introduce the following function $\widetilde{F}(\eta)$,

$$\widetilde{F}(\eta) := \int_{(\mathbb{R}^d)^P} dx_{i_1}...dx_{i_P} r(\mathbf{P},\mathbf{R},\mathbf{C})\left(K\left[\prod_{i=i_1}^{i_P}(1+D^{(i)}_{x_i}) - \prod_{j=j_1}^{j_R}(1+D^{(j)}_{x_j})\right]G\right)(\eta). \tag{139}$$

Then, the equation (138) can be rewritten as:

$$\left(L^{\mathrm{PRC}}KG\right)(\gamma) = \sum_{x^{(j_1)}_{j_1},...,x^{(j_R)}_{j_R}\in\gamma} \sum_{x^{(k_1)}_{k_1},...,x^{(k_C)}_{k_C}\in\gamma} \widetilde{F}(\gamma\backslash x^{(j_1)}_{j_1}\backslash...\backslash x^{(j_R)}_{j_R}). \tag{140}$$

Using the definition of the inverse K-transform and (140), the expression for $\widehat{L}$ becomes:

$$\begin{aligned}(\widehat{L}G)(\eta) &= \sum_{\xi\subset\eta}(-1)^{|\eta\backslash\xi|}(L^{\mathrm{PRC}}KG)(\xi) \\ &= \sum_{\xi\subset\eta}(-1)^{|\eta\backslash\xi|} \sum_{x^{(j_1)}_{j_1},...,x^{(j_R)}_{j_R}\in\xi} \sum_{x^{(k_1)}_{k_1},...,x^{(k_C)}_{k_C}\in\xi} \widetilde{F}(\xi\backslash x^{(j_1)}_{j_1}\backslash...\backslash x^{(j_R)}_{j_R}). \end{aligned} \tag{141}$$

One can change the sequence of the summation over reactants $\sum_{x_j}$ and the summation over the finite subset $\sum_{\xi}$,

$$\sum_{\xi\subset\eta}\sum_{x\in\xi}F(\xi,x) = \sum_{x\in\eta}\sum_{\xi\subset\eta\backslash x}F(\xi\cup x, x). \tag{142}$$

Thus, applying this to (141) the expression for $\widehat{L}$ becomes:

$$(\widehat{L}G)(\eta) = \sum_{x^{(j_1)}_{j_1},...,x^{(j_R)}_{j_R}\in\eta} \sum_{\xi\subset\eta\backslash x^{(j_1)}_{j_1}\backslash...\backslash x^{(j_R)}_{j_R}} (-1)^{|(\eta\backslash x^{(j_1)}_{j_1}\backslash...\backslash x^{(j_R)}_{j_R})\backslash\xi|} \sum_{x^{(k_1)}_{k_1},...,x^{(k_C)}_{k_C}\in\xi} \widetilde{F}(\xi).$$

After changing the sequence of the summation over catalysts $\sum_{x_k}$ and the summation over the finite subset $\sum_{\xi}$, the expression for $\widehat{L}$ becomes:

$$\begin{aligned}(\widehat{L}G)(\eta) &= \sum_{x^{(j_1)}_{j_1},...,x^{(j_R)}_{j_R}\in\eta} \sum_{x^{(k_1)}_{k_1},...,x^{(k_C)}_{k_C}\in\eta} \sum_{\xi\subset\eta\backslash x^{(j_1)}_{j_1}\backslash...\backslash x^{(j_R)}_{j_R}\backslash x^{(k_1)}_{k_1}\backslash...\backslash x^{(k_C)}_{k_C}} \\ & (-1)^{|(\eta\backslash x^{(j_1)}_{j_1}\backslash...\backslash x^{(j_R)}_{j_R}\backslash x^{(k_1)}_{k_1}\backslash...\backslash x^{(k_C)}_{k_C})\backslash\xi|} \prod_{k=k_1}^{k_C} D^{(k)}_{x_k}\widetilde{F}(\xi). \end{aligned} \tag{143}$$

It can be rewritten using the notation for the inverse K-transform:

$$(\widehat{L}G)(\eta) \;=\; \sum_{x_{j_1}^{(j_1)},\dots,x_{j_R}^{(j_R)}\in\eta} \;\; \sum_{x_{k_1}^{(k_1)},\dots,x_{k_C}^{(k_C)}\in\eta} \left(K^{-1}\prod_{k=k_1}^{k_C} D_{x_k}^{(k)}\widetilde{F}\right)(\eta\backslash x_{j_1}^{(j_1)}\backslash\dots\backslash x_{j_R}^{(j_R)}\backslash x_{k_1}^{(k_1)}\backslash\dots\backslash x_{k_C}^{(k_C)}).$$

Substituting $\widetilde{F}$ from its definition (139), one obtains:

$$(\widehat{L}G)(\eta) = \int_{(\mathbb{R}^d)^P} dx_{i_1}\dots dx_{i_P} \sum_{x_{j_1}^{(j_1)},\dots,x_{j_R}^{(j_R)}\in\eta} \;\; \sum_{x_{k_1}^{(k_1)},\dots,x_{k_C}^{(k_C)}\in\eta} r(\mathbf{P},\mathbf{R},\mathbf{C})\times$$
$$\times\left(K^{-1}\prod_{k=k_1}^{k_C} D_{x_k}^{(k)} K\left[\prod_{i=i_1}^{i_P}(1+D_{x_i}^{(i)}) - \prod_{j=j_1}^{j_R}(1+D_{x_j}^{(j)})\right]G\right)(\eta\backslash x_{j_1}^{(j_1)}\backslash\dots\backslash x_{j_R}^{(j_R)}\backslash x_{k_1}^{(k_1)}\backslash\dots\backslash x_{k_C}^{(k_C)}). \quad (144)$$

Using the property $D_x KG(\eta) = KG(\eta) + KD_xG(\eta)$, (135), one can show the following:

$$\begin{aligned}\prod_{k=k_1}^{k_C} D_{x_k}^{(k)} KG &= \prod_{k=k_1}^{k_{C-1}} D_{x_k}^{(k)} K\left(1 + D_{x_{k_C}}^{(k_C)}\right)G \\ &= K\prod_{k=k_1}^{k_C}\left(1 + D_{x_k}^{(k)}\right)G. \end{aligned} \quad (145)$$

Finally, substituting the above expression into (144), one derives the following expression for $(\widehat{L}G)(\eta)$,

$$(\widehat{L}G)(\eta) = \int_{(\mathbb{R}^d)^P} dx_{i_1}\dots dx_{i_P} \sum_{x_{j_1}^{(j_1)},\dots,x_{j_R}^{(j_R)}\in\eta} \;\; \sum_{x_{k_1}^{(k_1)},\dots,x_{k_C}^{(k_C)}\in\eta} r(\mathbf{P},\mathbf{R},\mathbf{C})\times$$
$$\times\left(\prod_{k=k_1}^{k_C}\left(1+D_{x_k}^{(k)}\right)\left[\prod_{i=i_1}^{i_P}(1+D_{x_i}^{(i)}) - \prod_{j=j_1}^{j_R}(1+D_{x_j}^{(j)})\right]G\right)(\eta\backslash x_{j_1}^{(j_1)}\backslash\dots\backslash x_{j_R}^{(j_R)}\backslash x_{k_1}^{(k_1)}\backslash\dots\backslash x_{k_C}^{(k_C)}). \quad (146)$$

1.4.3 Derivation of the operator L^{Δ}

The operator L^{Δ} can be determined from the following equation:

$$\int_{\Gamma_0} G(\eta)(L^{\Delta}k)(\eta)d\lambda(\eta) = \int_{\Gamma_0} (\widehat{L}G)(\eta)k(\eta)d\lambda(\eta). \quad (147)$$

Using the expression (146) for $\widehat{L}G$, all sums should be transformed into integrals. This is done by the following change of variables: $\eta \to \eta \cup x_{j_1}^{(j_1)} \cup \dots \cup x_{j_R}^{(j_R)} \cup x_{k_1}^{(k_1)} \cup \dots \cup x_{k_C}^{(k_C)}$, where the change of

variables is performed according to rules (45) and (46). Therefore, equation (147) becomes:

$$\int_{\Gamma_0} G(\eta)(L^{\Delta}k)(\eta)d\lambda(\eta) =$$
$$= \int_{\Gamma_0} d\lambda(\eta) \int_{(\mathbb{R}^d)^P} dx_{i_1}...dx_{i_P} \int_{(\mathbb{R}^d)^R} dx_{j_1}...dx_{j_R} \int_{(\mathbb{R}^d)^C} dx_{k_1}...dx_{k_C} r(\mathbf{P},\mathbf{R},\mathbf{C})\times$$
$$\times k(\eta \cup x_{j_1}^{(j_1)} \cup ... \cup x_{j_R}^{(j_R)} \cup x_{k_1}^{(k_1)} \cup ... \cup x_{k_C}^{(k_C)})\times$$
$$\times \left(\prod_{k=k_1}^{k_C} \left(1 + D_{x_k}^{(k)}\right) \left[\prod_{i=i_1}^{i_P} (1 + D_{x_i}^{(i)}) - \prod_{j=j_1}^{j_R} (1 + D_{x_j}^{(j)}) \right] G \right)(\eta). \quad (148)$$

In order to transform this expression further, consider the following transformations:

$$\begin{aligned} \int_{\Gamma_0} d\lambda(\eta) \int_{\mathbb{R}^d} dx_k k(\eta \cup \xi) \left(D_{x_k}^{(k)} G\right)(\eta) &= \int_{\Gamma_0} d\lambda(\eta) \int_{\mathbb{R}^d} dx_k k(\eta \cup \xi) G(\eta \cup x_k^{(k)}) \\ &= \int_{\Gamma_0} d\lambda(\eta) \sum_{x_k^{(k)} \in \eta} k(\eta \cup \xi \backslash x_k^{(k)}) G(\eta), \end{aligned} \quad (149)$$

by changing the name of the variable $x_k^{(k)}$ into $y_k^{(k)}$, the expression (149) continues as:

$$\begin{aligned} \int_{\Gamma_0} d\lambda(\eta) \sum_{x_k^{(k)} \in \eta} k(\eta \cup \xi \backslash x_k^{(k)}) G(\eta) &= \int_{\Gamma_0} d\lambda(\eta) \sum_{y_k^{(k)} \in \eta} k(\eta \cup \xi \backslash y_k^{(k)}) G(\eta) \\ &= \int_{\Gamma_0} d\lambda(\eta) \int_{\mathbb{R}^d} dx_k G(\eta) \left(\sum_{y_k^{(k)} \in \eta} \delta(x_k^{(k)} - y_k^{(k)}) \right) k(\eta \cup \xi \backslash x_k^{(k)}), \end{aligned} \quad (150)$$

where in the last expression the Dirac δ-function is used. One can introduce the following operator:

$$D_{x_m}^{\dagger(m)} := \left(\sum_{y_m^{(m)} \in \eta} \delta(x_m^{(m)} - y_m^{(m)}) \widetilde{D}_{x_m}^{\dagger(m)} \right), \quad (151)$$

where

$$\widetilde{D}_{x_m}^{\dagger(m)} k(\eta) := k(\eta \backslash x_m^{(m)}). \quad (152)$$

Then, from (150) one obtains:

$$\int_{\Gamma_0} d\lambda(\eta) \int_{\mathbb{R}^d} dx_k k(\eta \cup \xi) \left(D_{x_k}^{(k)} G\right)(\eta) = \int_{\Gamma_0} d\lambda(\eta) \int_{\mathbb{R}^d} dx_k \left(D_{x_k}^{\dagger(k)} k\right)(\eta \cup \xi) G(\eta), \quad (153)$$

which can be used to re-write the expression (148) in the following form:

$$\int_{\Gamma_0} G(\eta)(L^{\Delta}k)(\eta)d\lambda(\eta) =$$

$$= \int_{\Gamma_0} d\lambda(\eta)\int_{(\mathbb{R}^d)^P} dx_{j_1}...dx_{j_R}\int_{(\mathbb{R}^d)^R} dx_{k_1}...dx_{k_C}\int_{(\mathbb{R}^d)^C} dx_{i_1}...dx_{i_P} r(\mathbf{P},\mathbf{R},\mathbf{C})$$
$$\times G(\eta)\left(\prod_{k=k_1}^{k_C}\left(1+D^{\dagger(k)}_{x_k}\right)\left[\prod_{i=i_1}^{i_P}(1+D^{\dagger(i)}_{x_i})-\right.\right.$$
$$\left.\left.-\prod_{j=j_1}^{j_R}(1+D^{\dagger(j)}_{x_j})\right]k\right)(\eta\cup x^{(j_1)}_{j_1}\cup...\cup x^{(j_R)}_{j_R}\cup x^{(k_1)}_{k_1}\cup...\cup x^{(k_C)}_{k_C}). \tag{154}$$

From (154) the expression for L^{Δ} follows:

$$(L^{\Delta}k)(\eta) = \int_{(\mathbb{R}^d)^P} dx_{i_1}...dx_{i_P}\int_{(\mathbb{R}^d)^R} dx_{j_1}...dx_{j_R}\int_{(\mathbb{R}^d)^C} dx_{k_1}...dx_{k_C} r(\mathbf{P},\mathbf{R},\mathbf{C})\times$$
$$\times\left(\prod_{k=k_1}^{k_C}\left(1+D^{\dagger(k)}_{x_k}\right)\left[\prod_{i=i_1}^{i_P}(1+D^{\dagger(i)}_{x_i})-\right.\right.$$
$$\left.\left.-\prod_{j=j_1}^{j_R}(1+D^{\dagger(j)}_{x_j})\right]D^{(j_1)}_{x_{j_1}}...D^{(j_R)}_{x_{j_R}}D^{(k_1)}_{x_{k_1}}...D^{(k_C)}_{x_{k_C}}k\right)(\eta). \tag{155}$$

In order to obtain the expression for L^{Δ} for each particular case, operators $D^{\dagger}$ should be applied to k function. After relatively trivial and straightforward transformations in each particular case, the equation of evolution of correlation functions k can be obtained from the expression (155). Thus, the evolution of correlation functions for a general process discussed in this section is provided by

$$\frac{\partial k(\eta)}{\partial t} = (L^{\Delta}k)(\eta), \tag{156}$$

where the operator L^{Δ} is given by (155).

1.4.4 Derivation of the operator Q^{Δ}

The evolution of cumulants $u(\eta)$ is described by Q^{Δ}, which is formally defined by (51). Here we recall the definition (51),

$$(Q^{\Delta}u)(\eta) := \left[(\exp^{*-1}u)*\left(L^{\Delta}(\exp^{*}u)\right)\right](\eta), \tag{157}$$

i.e. the following equality holds:

$$\int_{\Gamma_0} e_{\lambda}(\theta,\eta)\left(L^{\Delta}(\exp^{*}u)\right)(\eta)d\lambda(\eta) = \int_{\Gamma_0} e_{\lambda}(\theta,\eta)\left((\exp^{*}u)*(Q^{\Delta}u)\right)(\eta)d\lambda(\eta). \tag{158}$$

Thus, using L^{Δ} defined by (155), one obtains:

$$(Q^{\Delta}u)(\eta) = \left[(\exp^{*-1}u)*\left(\int_{(\mathbb{R}^d)^{P+R+C}} dx_{i_1}...dx_{i_P}dx_{j_1}...dx_{j_R}dx_{k_1}...dx_{k_C} r(\mathbf{P},\mathbf{R},\mathbf{C})\times\right.\right.$$
$$\left.\left.\times\prod_{k=k_1}^{k_C}\left(1+D^{\dagger(k)}_{x_k}\right)\left[\prod_{i=i_1}^{i_P}(1+D^{\dagger(i)}_{x_i})-\prod_{j=j_1}^{j_R}(1+D^{\dagger(j)}_{x_j})\right]D^{(j_1)}_{x_{j_1}}...D^{(j_R)}_{x_{j_R}}D^{(k_1)}_{x_{k_1}}...D^{(k_C)}_{x_{k_C}}\exp^{*}u\right)\right](\eta). \tag{159}$$

According to the properties of the operator $D^{(m)}_{x_n}$, shown in (56), i.e. that $D_x(\exp^* u) = D_x u * (\exp^* u)$, one can represent the action of D operators on $\exp^* u$ in the following form:

$$D^{(j_1)}_{x_{j_1}}...D^{(j_R)}_{x_{j_R}}D^{(k_1)}_{x_{k_1}}...D^{(k_C)}_{x_{k_C}}\exp^* u = V * \exp^* u. \tag{160}$$

Also, one can use the following equality:

$$\int_{\Gamma_0} d\lambda(\eta)\int_{\mathbb{R}^d} dx_k D^{\dagger(k)}_{x_k}(V * \exp^* u)(\eta) = \int_{\Gamma_0} d\lambda(\eta)\sum_{x^{(k)}_k\subset\eta}((\widetilde{D}^{\dagger(k)}_{x_k}V) * \exp^* u)(\eta), \tag{161}$$

which can be proven by the following calculations using definitions (151) and (152) of $D^\dagger$ operators,

$$\begin{aligned}
&\int_{\Gamma_0} d\lambda(\eta)\int_{\mathbb{R}^d} dx_k e_\lambda(\theta,\eta) D^{\dagger(k)}_{x_k}(V * \exp^* u)(\eta) = \\
&\quad= \int_{\Gamma_0} d\lambda(\eta)\int_{\mathbb{R}^d} dx_k e_\lambda(\theta,\eta)\left(\sum_{y^{(k)}_k\in\eta}\delta(x^{(k)}_k - y^{(k)}_k)\widetilde{D}^{\dagger(k)}_{x_k}\right)(V * \exp^* u)(\eta) = \\
&\quad= \int_{\Gamma_0} d\lambda(\eta)\sum_{y^{(k)}_k\subset\eta} e_\lambda(\theta,\eta)(V * \exp^* u)(\eta\backslash y^{(k)}_k) \\
&\quad= \int_{\Gamma_0} d\lambda(\eta)\int_{\mathbb{R}^d} dy_k e_\lambda(\theta,\eta\cup y^{(k)}_k)(V * \exp^* u)(\eta) \\
&\quad= \int_{\Gamma_0} d\lambda(\eta)\int_{\mathbb{R}^d} dy_k e_\lambda(\theta,\eta)\theta(y^{(k)}_k)(V * \exp^* u)(\eta) \\
&\quad= \int_{\Gamma_0} d\lambda(\xi)e_\lambda(\theta,\xi)(\exp^* u)(\xi)\int_{\Gamma_0} d\lambda(\eta)\int_{\mathbb{R}^d} dy_k e_\lambda(\theta,\eta\cup y^{(k)}_k)V(\eta) \\
&\quad= \int_{\Gamma_0} d\lambda(\xi)e_\lambda(\theta,\xi)(\exp^* u)(\xi)\int_{\Gamma_0} d\lambda(\eta)\sum_{y^{(k)}_k\subset\eta} e_\lambda(\theta,\eta)V(\eta\backslash y^{(k)}_k).
\end{aligned} \tag{162}$$

Thus, substituting equation (160) into (159) and using (161), one can see that $\exp^* u$ and $\exp^{*-1} u$ cancel each other due to equalities $1^* * v = v$, and $(\exp^* u) * (\exp^{*-1} u) = 1^*$, shown in (52) and (53). This leads to the expression for Q^Δ,

$$\begin{aligned}
(Q^\Delta u)(\eta) = \Bigg(&\int_{(\mathbb{R}^d)^P} dx_{i_1}...dx_{i_P}\int_{(\mathbb{R}^d)^R} dx_{j_1}...dx_{j_R}\int_{(\mathbb{R}^d)^C} dx_{k_1}...dx_{k_C} r(\mathbf{P},\mathbf{R},\mathbf{C}) \times \\
&\times \prod_{k=k_1}^{k_C}\left(1 + D^{\dagger(k)}_{x_k}\right)\left[\prod_{i=i_1}^{i_P}(1 + D^{\dagger(i)}_{x_i}) - \prod_{j=j_1}^{j_R}(1 + D^{\dagger(j)}_{x_j})\right]V\Bigg)(\eta),
\end{aligned} \tag{163}$$

where, according to (160), V can be straightforwardly calculated as follows:

$$V := (\exp^{*-1} u) * \left(D^{(j_1)}_{x_{j_1}}...D^{(j_R)}_{x_{j_R}}D^{(k_1)}_{x_{k_1}}...D^{(k_C)}_{x_{k_C}}\exp^* u\right). \tag{164}$$

Thus, the evolution of cumulants for a general process discussed in this section is provided by

$$\frac{\partial u(\eta)}{\partial t} = (Q^\Delta u)(\eta), \tag{165}$$

where the operator Q^Δ is given by (163).

1.4.5 Derivation of functions H_q, H_p and H_g

Scaling of the interaction function $r(\mathbf{P},\mathbf{R},\mathbf{C})$

The mean-field solution obtained in the limit of long-ranged interactions exists only under a certain scaling of the function $r(\mathbf{P},\mathbf{R},\mathbf{C})$. In this section such a scaling of the function $r(\mathbf{P},\mathbf{R},\mathbf{C})$ is determined. The scaling is assumed to be of the following form, where r_ϵ denotes a scaled version of r,

$$r_\epsilon(\mathbf{P},\mathbf{R},\mathbf{C}) := \left(\epsilon^d\right)^\alpha r(\mathbf{P}_\epsilon,\mathbf{R}_\epsilon,\mathbf{C}_\epsilon), \tag{166}$$

where the exponent α is to be determined below in this section, and

$$\mathbf{P}_\epsilon := S_\epsilon \mathbf{P} = ((i_1,\epsilon x_{i_1}),(i_2,\epsilon x_{i_2}),...,(i_P,\epsilon x_{i_P})), \tag{167}$$

$\mathbf{R}_\epsilon$ and $\mathbf{C}_\epsilon$ are defined similarly to (167).

The derivation of α is demonstrated hereafter. In case of pairwise symmetric kernels we consider the following scaling of interaction kernels, where a scaled version of a kernel is denoted by a_ϵ,

$$a_\epsilon(x) := \epsilon^d a(\epsilon x). \tag{168}$$

The integral of the scaled kernel remains constant:

$$\int_{\mathbb{R}^d} a_\epsilon(x)dx = \int_{\mathbb{R}^d} a(x)dx. \tag{169}$$

Thus, formally, after the scaling procedure each operator of integration obtains a pre-factor ϵ^{-d},

$$\left(\int_{\mathbb{R}^d} dx\right)_\epsilon = \epsilon^{-d}\left(\int_{\mathbb{R}^d} d(\epsilon x)\right), \tag{170}$$

where $\left(\int_{\mathbb{R}^d} dx\right)_\epsilon$ denotes a scaled version of the integration. The operation $D^\dagger$ removes one integration, transforming it into a summation due to δ-function in the definition of $D^\dagger$. Therefore during the scaling procedure the operation $D^\dagger$ acquires a pre-factor ϵ^d in order to cancel the pre-factor ϵ^{-d} at the integration it is going to remove, i.e.

$$\left(D^\dagger\right)_\epsilon = \epsilon^d D^\dagger, \tag{171}$$

where $\left(D^\dagger\right)_\epsilon$ denotes a scaled version of $D^\dagger$.

In order for the mean-field solution to exist, the leading contribution in the solution should not depend on ϵ. This condition is satisfied only when the number of integrations in the expression (163) equals to the number $N_{D^\dagger}$ of $D^\dagger$ operators together with the power α of ϵ^d originating from the function $r(\mathbf{P},\mathbf{R},\mathbf{C})$, i.e.

$$N_{\text{int}} = N_{D^\dagger} + \alpha. \tag{172}$$

The number of integrations in (163) equals to $N_{\text{int}} = P+R+C$, where P, R and C are numbers of species in the group of producs, reactants and catalysts respectively. There cannot be less than one operator $D^\dagger$ in the expression (163), as it follows from the expression inside square brackets in (163). Thus, after the scaling procedure one formally has the following expansion for the following

part of the integrand function in Eq.(163),

$$\prod_{k=k_1}^{k_C}\left(1+D_{x_k}^{\dagger(k)}\right)\left[\prod_{i=i_1}^{i_P}(1+D_{x_i}^{\dagger(i)})-\prod_{j=j_1}^{j_R}(1+D_{x_j}^{\dagger(j)})\right]=\epsilon^d\left(\sum_{i=i_1}^{i_P}D_{x_i}^{\dagger(i)}-\sum_{j=j_1}^{j_R}D_{x_j}^{\dagger(j)}\right)+O(\epsilon^{2d}). \tag{173}$$

When considering the equation for the mean-field solution, one should consider only the leading contribution to (173) in the approximation of small ϵ^d. Thus, $N_{D^\dagger}=1$. Therefore, in order for the mean-field solution to exist, the maximal power of ϵ^d in a pre-factor in the function $r(\mathbf{P},\mathbf{R},\mathbf{C})$ can be the following:

$$\alpha = P+R+C-1. \tag{174}$$

Moreover, α can be equal only to $P+R+C-1$, because if the power is larger, then after the scaling procedure the mean-field solution will be approaching zero when $\epsilon^d\to 0$; if the power is smaller, then mean-field solution diverges when $\epsilon^d\to 0$. Thus, the scaling of the function $r(\mathbf{P},\mathbf{R},\mathbf{C})$ is the following:

$$r_\epsilon(\mathbf{P},\mathbf{R},\mathbf{C}) := \left(\epsilon^d\right)^{P+R+C-1} r(\mathbf{P}_\epsilon,\mathbf{R}_\epsilon,\mathbf{C}_\epsilon). \tag{175}$$

Equations of the evolution of the mean-field solution and its corrections are obtained by substituting the perturbation expansion (29) of the solution u_ϵ into the scaled and renormalized evolution equation for cumulants. Thus, the solution u_ϵ considered in the following form,

$$u_\epsilon(t,\eta) = \mathbb{1}_{\eta=\{x^{(m)}\}}\left(q_m(t,x)+\epsilon^d p_m(t,x)\right)+\mathbb{1}_{\eta=\{x_1^{(m)},x_2^{(n)}\}}\epsilon^d g_{mn}(t,x_1,x_2)+O(\epsilon^d), \tag{176}$$

leads to equations of evolutions for q,p,g functions, as in (33):

$$\begin{aligned}\frac{\partial}{\partial t}q_m(t,x) &= H_{q_m}(t,x);\\ \frac{\partial}{\partial t}p_m(t,x) &= H_{p_m}(t,x);\\ \frac{\partial}{\partial t}g_{mn}(t,x,y) &= H_{g_{mn}}(t,x,y).\end{aligned} \tag{177}$$

Derivation of H_q

Substituting (176), (163), (164) and (173) into the scaled and renormalized equations of the evolution of cumulants, one obtains equations for mean field q_m, where $m\in\{i_1,...,i_P,\ j_1,...,j_R,\ k_1,...,k_C\}$,

$$\begin{aligned}\frac{\partial q_m(x)}{\partial t} &= \int_{(\mathbb{R}^d)^{P+R+C}} dx_{i_1}...dx_{i_P}dx_{j_1}...dx_{j_R}dx_{k_1}...dx_{k_C} r(\mathbf{P},\mathbf{R},\mathbf{C})\left(\sum_{i=i_1}^{i_P}D_{x_i}^{\dagger(i)}-\sum_{j=j_1}^{j_R}D_{x_j}^{\dagger(j)}\right)\times\\ &\times\left(\left(\exp^{*-1}u_\epsilon\right)*\left(D_{x_{j_1}}^{(j_1)}...D_{x_{j_R}}^{(j_R)}D_{x_{k_1}}^{(k_1)}...D_{x_{k_C}}^{(k_C)}\exp^* u_\epsilon\right)\right)(\eta)\Big|_{\eta=\{x^{(m)}\}}.\end{aligned} \tag{178}$$

Here, u_ϵ can have non-zero value only on a single point configuration. Only one point is removed by operation $D^\dagger$, but each operation D adds one point. Taking into account that the configuration $\eta=\{x^{(m)}\}$ has only a single point, operations $D^\dagger$ and D should cancel each other. Therefore, only a single term remains after acting by D on $\exp^* u$, where the contributions are given only by q

and not by p, as p would bring additional power of ϵ^d. Thus,

$$\frac{\partial q_m(x)}{\partial t} = \int_{(\mathbb{R}^d)^{P+R+C}} dx_{i_1}...dx_{i_P}dx_{j_1}...dx_{j_R}dx_{k_1}...dx_{k_C} r(\mathbf{P},\mathbf{R},\mathbf{C}) \left(\sum_{i=i_1}^{i_P} D^{\dagger(i)}_{x_i} - \sum_{j=j_1}^{j_R} D^{\dagger(j)}_{x_j}\right) \times \times \left(D^{(j_1)}_{x_{j_1}} q * ... * D^{(j_R)}_{x_{j_R}} q * D^{(k_1)}_{x_{k_1}} q * ... * D^{(k_C)}_{x_{k_C}} q\right)(\eta)\Big|_{\eta=\{x^{(m)}\}}. \tag{179}$$

Consider the action of $D^\dagger$ operators first. They acts on η only if m coincides with the index of $D^\dagger$, i.e. $D^{\dagger(i)}_{x_i}$ can remove a point $x_i^{(i)}$ from the configuration $\eta = \{x^{(m)}\}$ only if $x_i^{(i)} = x^{(m)}$, or, the same, if $i = m$ and $x_i = x$. After the action of $D^\dagger$ operators on η the configuration becomes empty, $|\eta| = 0$, and therefore the convolution becomes just a product of q functions on corresponding points added by D operators:

$$\frac{\partial q_m(x)}{\partial t} = \int_{(\mathbb{R}^d)^{P+R+C}} dx_{i_1}...dx_{i_P}dx_{j_1}...dx_{j_R}dx_{k_1}...dx_{k_C} r(\mathbf{P},\mathbf{R},\mathbf{C}) \times \times \left(\sum_{i=i_1}^{i_P} \delta_{mi}\delta(x_i - x) - \sum_{j=j_1}^{j_R} \delta_{mj}\delta(x_j - x)\right) \prod_{\beta_\varsigma \in \{j_1,...,k_C\}} q_{\beta_\varsigma}(x_{\beta_\varsigma}). \tag{180}$$

This is the equation for q in the general process. In translationally invariant case there should be no dependence on x, so one can choose $x = 0$.

$$H_{q_m} = \frac{\partial q_m}{\partial t} = \left(\prod_{\beta_\varsigma \in \{j_1,...,k_C\}} q_{\beta_\varsigma}\right) \int_{(\mathbb{R}^d)^{P+R+C}} dx_{i_1}...dx_{i_P}dx_{j_1}...dx_{j_R}dx_{k_1}...dx_{k_C} r(\mathbf{P},\mathbf{R},\mathbf{C}) \times \times \left(\sum_{i=i_1}^{i_P} \delta_{mi}\delta(x_i) - \sum_{j=j_1}^{j_R} \delta_{mj}\delta(x_j)\right). \tag{181}$$

Derivation of H_p

The right hand side of the equation for p is the expression which has a pre-factor with a single additional power of ϵ^d when compared to the equation for q, Eq.(180). This additional single power can have one origin from the three possible ones: (1) from an additional operation $D^\dagger$, (2) from a single usage of p instead of q; (3) from a single usage of g instead of q. Thus, the final expression consists of three terms (1), (2), (3) named correspondingly to the origin of the additional power of ϵ^d.

In the term (1), taking into accout that q exists on a single point configuration only, the action of a single $D^\dagger$ on such a single-point configuration creates an empty set, therefore the action of a second $D^\dagger$ is zero. Therefore, the contribution (1) is zero.

In the term (2) the $*-$product is effectively defined on an empty set (obtained after removal of the initial point by $D^\dagger$). Operators D act on q and p, thus creating a single point configuration. Thus, $*-$product becomes just a product. One D acts on p, and there is a summation over index of D acting on p.

In the term (3), similarly to the case (1), there are two operators D acting on g. Corresponding summation over indices of these two D is present. The $*-$product is also defined on an empty set (initial point is removed by $D^\dagger$). Thus, the $*-$product becomes just a product.

The resulting expression becomes:

$$\frac{\partial p_m(x)}{\partial t} = \int_{(\mathbb{R}^d)^{P+R+C}} dx_{i_1}...dx_{i_P}dx_{j_1}...dx_{j_R}dx_{k_1}...dx_{k_C} r(\mathbf{P},\mathbf{R},\mathbf{C}) \times$$

$$\times\left\{\left(\sum_{i=i_1}^{i_P} D_{x_i}^{\dagger(i)} - \sum_{j=j_1}^{j_R} D_{x_j}^{\dagger(j)}\right) \sum_{\substack{\beta_1\in\{j_1,...k_C\} \\ \beta_\zeta\neq\beta_1;\zeta=\overline{2,R+C} \\ \beta_2<\beta_3<...<\beta_{R+C} \\ \beta_1\cup\{\beta_\zeta\}=\{j_1,...k_C\}}} \left(D_{x_{\beta_1}}^{(\beta_1)} p * D_{x_{\beta_2}}^{(\beta_2)} q * ... * D_{x_{\beta_{R+C}}}^{(\beta_{R+C})} q\right)(\eta)\Big|_{\eta=\{x^{(m)}\}} + \right.$$

$$+\left(\sum_{i=i_1}^{i_P} D_{x_i}^{\dagger(i)} - \sum_{j=j_1}^{j_R} D_{x_j}^{\dagger(j)}\right) \times$$

$$\left.\times \sum_{\substack{\beta_1<\beta_2\in\{j_1,...k_C\} \\ \beta_\zeta\neq\beta_1,\beta_2;\zeta=\overline{3,R+C} \\ \beta_3<\beta_4<...<\beta_{R+C} \\ \beta_1\cup\beta_2\cup\{\beta_\zeta\}=\{j_1,...k_C\}}} \left(D_{x_{\beta_1}}^{(\beta_1)} D_{x_{\beta_2}}^{(\beta_2)} g * D_{x_{\beta_3}}^{(\beta_3)} q * ... * D_{x_{\beta_{R+C}}}^{(\beta_{R+C})} q\right)(\eta)\Big|_{\eta=\{x^{(m)}\}}\right\}. \tag{182}$$

Finally, transforming $*-$product as discussed before, one obtains the equation of the evolution of p_m,

$$\begin{aligned}\frac{\partial p_m(x)}{\partial t} &= \int_{(\mathbb{R}^d)^{P+R+C}} dx_{i_1}...dx_{i_P}dx_{j_1}...dx_{j_R}dx_{k_1}...dx_{k_C} r(\mathbf{P},\mathbf{R},\mathbf{C}) \times \\ &\times\left(\sum_{i=i_1}^{i_P} \delta_{mi}\delta(x_i - x) - \sum_{j=j_1}^{j_R} \delta_{mj}\delta(x_j - x)\right) \times \\ &\times\left(\sum_{\beta_1\in\{j_1,...k_C\}} p_{\beta_1}(x_{\beta_1}) \prod_{\beta_\zeta\in\{j_1,...k_C\}\backslash\beta_1} q_{\beta_\zeta}(x_{\beta_\zeta}) + \right. \\ &\left. + \sum_{\beta_1=j_1}^{k_C} \sum_{\beta_2=\beta_1+1}^{k_C} g_{\beta_1\beta_2}(x_{\beta_1}, x_{\beta_2}) \prod_{\beta_\zeta\in\{j_1,...k_C\}\backslash\beta_1\backslash\beta_2} q_{\beta_\zeta}(x_\zeta)\right).\end{aligned} \tag{183}$$

In translationally invariant case one obtains:

$$
\begin{aligned}
H_{p_m} = \frac{\partial p_m}{\partial t} &= \int_{(\mathbb{R}^d)^{P+R+C}} dx_{i_1}...dx_{i_P} dx_{j_1}...dx_{j_R} dx_{k_1}...dx_{k_C} r(\mathbf{P},\mathbf{R},\mathbf{C}) \times \\
&\times \left(\sum_{i=i_1}^{i_P} \delta_{mi}\delta(x_i) - \sum_{j=j_1}^{j_R} \delta_{mj}\delta(x_j) \right) \times \\
&\times \Bigg(\sum_{\beta_1 \in \{j_1,...k_C\}} p_{\beta_1} \prod_{\beta_\zeta \in \{j_1,...k_C\}\setminus\beta_1} q_{\beta_\zeta} + \\
&\quad + \sum_{\beta_1=j_1}^{k_C} \sum_{\beta_2=\beta_1+1}^{k_C} g_{\beta_1\beta_2}(x_{\beta_1} - x_{\beta_2}) \prod_{\beta_\zeta \in \{j_1,...k_C\}\setminus\beta_1\setminus\beta_2} q_{\beta_\zeta} \Bigg).
\end{aligned} \tag{184}
$$

Derivation of H_g

Equation for g originates form the same order in powers of ϵ^d as equation for p, but it is calculated on two points configuration $\eta = \{x_m^{(m)}, x_n^{(n)}\}$. Similar contributions will be present, with the same single occurrence of p or g, or term with just q. But more variations of how Ds act. Thus, there will be similar terms (1), (2), (3) as in equation for p.

In the term (1): effectively $\star$−product is defined on empty set, as two initial points are taken by two $D^\dagger$. Therefore, there is no need to have a second D acting on a single q, then every q will get an empty set and one D resulting in a single point configuration.

In the term (2): $|\eta| = 1$ in the $\star$−product, however this term contains only q and p, and an additional point is already created in each of them, therefore the total configuration for at one of them will be a two point configuration. Therefore, the whole term is zero. Thus, equation on g does not depend on p.

In term (3): again, $|\eta| = 1$ in the $\star$−product, thus it is similar to the term (2). However, this term contains g, which is non-zero on two point configuration. Thus, formally changing p in term (2) into g one obtains term (3).

$$
\begin{aligned}
\frac{\partial g_{mn}}{\partial t} &= \mathbb{1}_{\eta=\{x_1^{(m)},x_2^{(n)}\}} \int_{(\mathbb{R}^d)^{P+R+C}} dx_{i_1}...dx_{i_P} dx_{j_1}...dx_{j_R} dx_{k_1}...dx_{k_C} r(\mathbf{P},\mathbf{R},\mathbf{C}) \times \\
\times & \Bigg\{ \Bigg[\sum_{i=i_1}^{i_P} \sum_{i'>i}^{i_P} D_{x_i}^{\dagger(i)} D_{x_{i'}}^{\dagger(i)} - \sum_{j=j_1}^{j_R} \sum_{j'>j}^{j_R} D_{x_j}^{\dagger(j)} D_{x_{j'}}^{\dagger(j)} + \sum_{k=k_1}^{k_C} D_{x_k}^{\dagger(k)} \left(\sum_{i=i_1}^{i_P} D_{x_i}^{\dagger(i)} - \sum_{j=j_1}^{j_R} D_{x_j}^{\dagger(j)} \right) \Bigg] \times \\
& \times \left(D_{x_{j_1}}^{(j_1)} q \star ... \star D_{x_{j_R}}^{(j_R)} q \star ... \star D_{x_{k_C}}^{(k_C)} q \right)(\eta) + \\
& + \left(\sum_{i=i_1}^{i_P} D_{x_i}^{\dagger(i)} - \sum_{j=j_1}^{j_R} D_{x_j}^{\dagger(j)} \right) \sum_{\substack{\beta_1 \in \{j_1,...k_C\} \\ \beta_\zeta \neq \beta_1; \zeta=\overline{2,R+C} \\ \beta_2<\beta_3<...<\beta_{R+C} \\ \beta_1 \cup \{\beta_\zeta\} = \{j_1,...k_C\}}} \left(D_{x_{\beta_1}}^{(\beta_1)} g \star D_{x_{\beta_2}}^{(\beta_2)} q \star ... \star D_{x_{\beta_{R+C}}}^{(\beta_{R+C})} q \right)(\eta) \Bigg\}.
\end{aligned} \tag{185}
$$

Now the convolution can be transformed as discussed before. Using the definition of $D^\dagger$ operators

explicitly, the expression becomes:

$$
\begin{aligned}
\frac{\partial g_{mn}(x_1,x_2)}{\partial t} &= \int_{(\mathbb{R}^d)^{P+R+C}} dx_{i_1}...dx_{i_P} dx_{j_1}...dx_{j_R} dx_{k_1}...dx_{k_C} r(\mathbf{P},\mathbf{R},\mathbf{C}) \times \\
\times \Bigg\{ \Bigg[& \sum_{i=i_1}^{i_P} \sum_{i'>i}^{i_P} (\delta_{mi}\delta_{ni'} + \delta_{mi'}\delta_{ni}) \sum_{y_i^{(i)}\in\eta} \delta(x_i - y_i) \sum_{y_{i'}^{(i')}\in\eta} \delta(x_{i'} - y_{i'}) \\
& - \sum_{j=j_1}^{j_R} \sum_{j'>j}^{j_R} (\delta_{mj}\delta_{nj'} + \delta_{mj'}\delta_{nj}) \sum_{y_j^{(j)}\in\eta} \delta(x_j - y_j) \sum_{y_{j'}^{(j')}\in\eta} \delta(x_{j'} - y_{j'}) \\
& + \left(\sum_{k=k_1}^{k_C} (\delta_{mk} + \delta_{nk}) \sum_{y_k^{(k)}\in\eta} \delta(x_k - y_k) \right) \left(\sum_{i=i_1}^{i_P} (\delta_{mi} + \delta_{ni}) \sum_{y_i^{(i)}\in\eta} \delta(x_i - y_i) \right) \\
& - \left(\sum_{k=k_1}^{k_C} (\delta_{mk} + \delta_{nk}) \sum_{y_k^{(k)}\in\eta} \delta(x_k - y_k) \right) \left(\sum_{j=j_1}^{j_R} (\delta_{mj} + \delta_{nj}) \sum_{y_j^{(j)}\in\eta} \delta(x_j - y_j) \right) \Bigg] \times \\
& \times q_{j_1}(x_{j_1})...q_{j_R}(x_{j_R}) q_{k_1}(x_{k_1})...q_{k_C}(x_{k_C}) \\
& + \sum_{i=i_1}^{i_P} \sum_{y_i^{(i)}\in\eta} \delta(x_i - y_i) \sum_{\beta_1\in\{j_1,...k_C\}} [\delta_{mi} g_{\beta_1 n}(x_{\beta_1}, x_2) + \delta_{ni} g_{m\beta_1}(x_1, x_{\beta_1})] \times \\
& \quad \times \prod_{\beta_\zeta\in\{j_1,...k_C\}\setminus\beta_1} q_{\beta_\zeta}(x_{\beta_\zeta}) \\
& - \sum_{j=j_1}^{j_R} \sum_{y_j^{(j)}\in\eta} \delta(x_j - y_j) \sum_{\beta_1\in\{j_1,...k_C\}} [\delta_{mj} g_{\beta_1 n}(x_{\beta_1}, x_2) + \delta_{nj} g_{m\beta_1}(x_1, x_{\beta_1})] \times \\
& \quad \times \prod_{\beta_\zeta\in\{j_1,...k_C\}\setminus\beta_1} q_{\beta_\zeta}(x_{\beta_\zeta}) \Bigg\}.
\end{aligned}
\tag{186}
$$

It is convenient to use the explicit form $\eta = \{x_1^{(m)}, x_2^{(n)}\}$ in sums $\sum_{y\in\eta}$, and to perform summations over points from such configurations η. Noticing, that terms which contain $\delta_{mk}\delta_{mi}$ are zero, one obtains the final equation of the evolution of the g function:

$$
\begin{aligned}
\frac{\partial g_{mn}(x_1,x_2)}{\partial t} &= \int_{(\mathbb{R}^d)^{P+R+C}} dx_{i_1}...dx_{i_P}dx_{j_1}...dx_{j_R}dx_{k_1}...dx_{k_C} r(\mathbf{P},\mathbf{R},\mathbf{C}) \times \\
&\times \Bigg\{ \Bigg[\sum_{i=i_1}^{i_P} \sum_{i'>i}^{i_P} (\delta_{mi}\delta_{ni'}\delta(x_i - x_1)\delta(x_{i'} - x_2) + \delta_{mi'}\delta_{ni}\delta(x_i - x_2)\delta(x_{i'} - x_1)) \\
&- \sum_{j=j_1}^{j_R} \sum_{j'>j}^{j_R} (\delta_{mj}\delta_{nj'}\delta(x_j - x_1)\delta(x_{j'} - x_2) + \delta_{mj'}\delta_{nj}\delta(x_j - x_2)\delta(x_{j'} - x_1)) \\
&+ \sum_{k=k_1}^{k_C} \delta_{mk}\delta(x_k - x_1) \sum_{i=i_1}^{i_P} \delta_{ni}\delta(x_i - x_2) + \sum_{i=i_1}^{i_P} \delta_{mi}\delta(x_i - x_1) \sum_{k=k_1}^{k_C} \delta_{nk}\delta(x_k - x_2) \\
&- \sum_{k=k_1}^{k_C} \delta_{mk}\delta(x_k - x_1) \sum_{j=j_1}^{j_R} \delta_{nj}\delta(x_j - x_2) - \sum_{j=j_1}^{j_R} \delta_{mj}\delta(x_j - x_1) \sum_{k=k_1}^{k_C} \delta_{nk}\delta(x_k - x_2) \Bigg] \times \\
&\qquad \times q_{j_1}(x_{j_1})...q_{j_R}(x_{j_R}) q_{k_1}(x_{k_1})...q_{k_C}(x_{k_C}) \\
+ & \sum_{i=i_1}^{i_P} \sum_{\beta_1 \in \{j_1,...k_C\}} [\delta_{mi}\delta(x_i - x_1) g_{\beta_1 n}(x_{\beta_1}, x_2) + \delta_{ni}\delta(x_i - x_2) g_{m\beta_1}(x_1, x_{\beta_1})] \times \\
&\qquad \times \prod_{\beta_\zeta \in \{j_1,...k_C\} \backslash \beta_1} q_{\beta_\zeta}(x_{\beta_\zeta}) \\
- & \sum_{j=j_1}^{j_R} \sum_{\beta_1 \in \{j_1,...k_C\}} [\delta_{mj}\delta(x_j - x_1) g_{\beta_1 n}(x_{\beta_1}, x_2) + \delta_{nj}\delta(x_j - x_2) g_{m\beta_1}(x_1, x_{\beta_1})] \times \\
&\qquad \times \prod_{\beta_\zeta \in \{j_1,...k_C\} \backslash \beta_1} q_{\beta_\zeta}(x_{\beta_\zeta}) \Bigg\} .
\end{aligned} \tag{187}
$$

In translationally invariant case, one obtains:

$$
\begin{aligned}
&H_{g_{mn}}(x_1) = \frac{\partial g_{mn}(x_1)}{\partial t} = \\
&= \int_{(\mathbb{R}^d)^{P+R+C}} dx_{i_1}...dx_{i_P} dx_{j_1}...dx_{j_R} dx_{k_1}...dx_{k_C} r(\mathbf{P},\mathbf{R},\mathbf{C}) \times \\
&\times \Bigg\{ \Bigg[\sum_{i=i_1}^{i_P} \sum_{i'>i}^{i_P} (\delta_{mi}\delta_{ni'}\delta(x_i - x_1)\delta(x_{i'}) + \delta_{mi'}\delta_{ni}\delta(x_i)\delta(x_{i'} - x_1)) \\
&- \sum_{j=j_1}^{j_R} \sum_{j'>j}^{j_R} (\delta_{mj}\delta_{nj'}\delta(x_j - x_1)\delta(x_{j'}) + \delta_{mj'}\delta_{nj}\delta(x_j)\delta(x_{j'} - x_1)) \\
&+ \sum_{k=k_1}^{k_C} \delta_{mk}\delta(x_k - x_1) \sum_{i=i_1}^{i_P} \delta_{ni}\delta(x_i) + \sum_{i=i_1}^{i_P} \delta_{mi}\delta(x_i - x_1) \sum_{k=k_1}^{k_C} \delta_{nk}\delta(x_k) \\
&- \sum_{k=k_1}^{k_C} \delta_{mk}\delta(x_k - x_1) \sum_{j=j_1}^{j_R} \delta_{nj}\delta(x_j) - \sum_{j=j_1}^{j_R} \delta_{mj}\delta(x_j - x_1) \sum_{k=k_1}^{k_C} \delta_{nk}\delta(x_k) \Bigg] \prod_{\beta\in\{j_1,...,k_C\}} q_\beta \\
&+ \sum_{i=i_1}^{i_P} \sum_{\beta_1\in\{j_1,...k_C\}} [\delta_{mi}\delta(x_i - x_1) g_{\beta_1 n}(x_{\beta_1}) + \delta_{ni}\delta(x_i) g_{m\beta_1}(x_1 - x_{\beta_1})] \prod_{\beta_\zeta\in\{j_1,...k_C\}\setminus\beta_1} q_{\beta_\zeta} \\
&- \sum_{j=j_1}^{j_R} \sum_{\beta_1\in\{j_1,...k_C\}} [\delta_{mj}\delta(x_j - x_1) g_{\beta_1 n}(x_{\beta_1}) + \delta_{nj}\delta(x_j) g_{m\beta_1}(x_1 - x_{\beta_1})] \prod_{\beta_\zeta\in\{j_1,...k_C\}\setminus\beta_1} q_{\beta_\zeta} \Bigg\}.
\end{aligned}
\tag{188}
$$

1.4.6 The general case of spatially heterogeneous systems

The equations (180), (183), and (187) describe evolution of q, p and g functions in a general case of spatially heterogeneous systems, including systems with spatially heterogeneous initial conditions. These equations can be used to study many interesting problems, for example such as invasion from small spatially restricted initial population, or externally defined environmental heterogeneity. These equations can be studied analytically or numerically. However, as such studies can be done in many different ways, development of the software incorporating a general module for external heterogeneity is beyond the scope of the present paper.

1.4.7 List of expressions for H_q, H_p and H_g for selected basic processes

Using expressions (181), (184) and (188) for functions H_{q_m}, H_{p_m} and $H_{g_{mn}}(x)$ for a general process in spatially homogeneous system, such functions are calculated for each selected basic process considered in section 1.3. To recall, the definition of H_{q_m}, H_{p_m} and $H_{g_{mn}}(x)$ are given by the following equations:

$$\frac{\partial q_m}{\partial t} = H_{q_m}; \tag{189}$$

$$\frac{\partial p_m}{\partial t} = H_{p_m}; \tag{190}$$

$$\frac{\partial g_{mn}(x)}{\partial t} = H_{g_{mn}}(x). \tag{191}$$

We also use the following notations: $A = \int_{\mathbb{R}^d} a(x)dx$, $B = \int_{\mathbb{R}^d} b(x)dx$.

Immigration: $\{P,R,C\} = \{1,0,0\}$. Process definition: an immigrant of type i appears spontaneously at location x with rate per unit area r.

$$\begin{aligned}
(L_i^{\mathrm{IM}}(r)F)(\gamma) &= r\int_{\mathbb{R}^d}(L_i^{x^+}F)(\gamma)dx \\
&= r\int_{\mathbb{R}^d}dx\left(F(\gamma\cup x^{(i)})-F(\gamma)\right); \\
H_{q_m,i}^{\mathrm{IM}(r)} &= r\delta_{mi}; \\
H_{p_m,i}^{\mathrm{IM}(r)} &= 0; \\
H_{g_{mn},i}^{\mathrm{IM}(r)}(x) &= 0.
\end{aligned} \tag{192}$$

Birth: $\{P,R,C\} = \{1,0,1\}$. Process definition: existing individuals of type i produce new individuals of type i with kernel a.

$$\begin{aligned}
(L_i^{\mathrm{B}}(a)F)(\gamma) &= \sum_{y\in\gamma_i}\int_{\mathbb{R}^d}a(x-y)(L_i^{x^+}F)(\gamma)dx \\
&= \sum_{y\in\gamma_i}\int_{\mathbb{R}^d}a(x-y)(F(\gamma\cup x^{(i)})-F(\gamma))dx; \\
H_{q_m,i}^{\mathrm{B}(a)} &= \delta_{mi}Aq_i; \\
H_{p_m,i}^{\mathrm{B}(a)} &= \delta_{mi}Ap_i; \\
H_{g_{mn},i}^{\mathrm{B(a)}}(x_1) &= \delta_{mi}\delta_{ni}2a(x_1)q_i+\int_{\mathbb{R}^d}dxa(x-x_1)(\delta_{mi}g_{in}(x)+\delta_{ni}g_{mi}(x)).
\end{aligned} \tag{193}$$

Birth to another type: $\{P,R,C\} = \{1,0,1\}$**.** Process definition: existing individuals of type k produce new individuals of type i with the kernel a, which incorporates both fecundity and dispersal.

$$\begin{aligned}
(L_{ik}^{\mathrm{BT}}(a)F)(\gamma) &= \sum_{y\in\gamma_k}\int_{\mathbb{R}^d}a(x-y)(L_i^{x^+}F)(\gamma)dx \\
&= \int_{\mathbb{R}^d}dx_1\sum_{x_2^{(k)}\in\gamma}\left(F(\gamma\cup x_1^{(i)})-F(\gamma)\right)a(x_1-x_2); \\
H_{q_m,ik}^{\mathrm{BT}(a)} &= \delta_{mi}Aq_k; \\
H_{p_m,ik}^{\mathrm{BT}(a)} &= \delta_{mi}Ap_k; \\
H_{g_{mn},ik}^{\mathrm{BT}(a)}(x_1) &= a(x_1)q_k\left[\delta_{mk}\delta_{ni}+\delta_{mi}\delta_{nk}\right]+\int_{\mathbb{R}^d}dxa(x_1-x)\left[\delta_{mi}g_{kn}(x)+\delta_{ni}g_{mk}(x)\right].
\end{aligned} \tag{194}$$

Birth to another type by facilitation: $\{P,R,C\} = \{1,0,2\}$. Process definition: type k_1 individuals produce offspring of type i_1 with kernel b, mediated by (but not affecting) type k_2

$(k_1 \neq k_2)$ individuals with kernel a.

$$
\begin{aligned}
(L^{\mathrm{BTF}}_{i_1k_1k_2}(a,b)F)(\gamma) &= \sum_{y\in\gamma_{k_1}}\sum_{z\in\gamma_{k_2}}\int_{\mathbb{R}^d} a(z-y)b(x-y)(L^{x^+}_{i_1}F)(\gamma)dx \\
&= \int_{\mathbb{R}^d} dx_1 \sum_{x_2^{(k_1)}\in\gamma}\sum_{x_3^{(k_2)}\in\gamma}\Big(F(\gamma\cup x_1^{(i_1)})-F(\gamma)\Big)a(x_3-x_2)b(x_1-x_2); \\
H^{\mathrm{BTF}(a,b)}_{q_m,i_1k_1k_2} &= \delta_{mi_1}q_{k_1}q_{k_2}AB; \\
H^{\mathrm{BTF}(a,b)}_{p_m,i_1k_1k_2} &= \delta_{mi_1}B\left(A(p_{k_1}q_{k_2}+p_{k_2}q_{k_1})+\int_{\mathbb{R}^d}dx_3 a(x_3)g_{k_1k_2}(x_3)\right); \\
H^{\mathrm{BTF}(a,b)}_{g_{mn},i_1k_1k_2}(x) &= (\delta_{mk_1}\delta_{ni_1}+\delta_{mi_1}\delta_{nk_1})q_{k_1}q_{k_2}b(x)A \\
&\quad +(\delta_{mk_2}\delta_{ni_1}+\delta_{mi_1}\delta_{nk_2})q_{k_1}q_{k_2}\int_{\mathbb{R}^d}dx_2 a(x-x_2)b(x_2) \\
&\quad +\delta_{mi_1}A\int_{\mathbb{R}^d}dx_2 b(x-x_2)g_{k_1n}(x_2)q_{k_2} \\
&\quad +\delta_{ni_1}A\int_{\mathbb{R}^d}dx_2 b(x_2)g_{mk_1}(x-x_2)q_{k_2} \\
&\quad +\delta_{mi_1}\int_{(\mathbb{R}^d)^2}dx_2dx_3 a(x_3-x_2)b(x-x_2)g_{k_2n}(x_3)q_{k_1} \\
&\quad +\delta_{ni_1}\int_{(\mathbb{R}^d)^2}dx_2dx_3 a(x_3-x_2)b(x_2)g_{mk_2}(x-x_3)q_{k_1}.
\end{aligned}
\tag{195}
$$

Birth by facilitation: $\{P,R,C\} = \{1,0,2\}$**.** Process definition: type i individuals produce offspring of type i with kernel b, mediated by (but not affecting) type k $(i \neq k)$ individuals with kernel a.

$$
\begin{aligned}
(L^{\mathrm{BF}}_{ik}(a,b)F)(\gamma) &= \sum_{y\in\gamma_i}\sum_{z\in\gamma_k}\int_{\mathbb{R}^d} a(z-y)b(x-y)(L^{x^+}_{i}F)(\gamma)dx \\
&= \int_{\mathbb{R}^d} dx_1 \sum_{x_2^{(i)}\in\gamma}\sum_{x_3^{(k)}\in\gamma}\Big(F(\gamma\cup x_1^{(i)})-F(\gamma)\Big)a(x_3-x_2)b(x_2-x_1); \\
H^{\mathrm{BF}(a,b)}_{q_m,ik} &= \delta_{mi}ABq_iq_k; \\
H^{\mathrm{BF}(a,b)}_{p_m,ik} &= \delta_{mi}\left(AB(q_ip_k+p_iq_k)+B\int_{\mathbb{R}^d}dz\ g_{ik}(z)a(z)\right); \\
H^{\mathrm{BF}(a,b)}_{g_{mn},ik}(x) &= \delta_{mi}\delta_{ni}2q_iq_kAb(x) \\
&\quad +(\delta_{mk}\delta_{ni}+\delta_{mi}\delta_{nk})q_iq_k\int_{\mathbb{R}^d}dx_2 a(x-x_2)b(x_2) \\
&\quad +Aq_k\int_{\mathbb{R}^d}dx_2 b(x-x_2)\left[\delta_{mi}g_{in}(x_2)+\delta_{ni}g_{mi}(x_2)\right] \\
&\quad +q_i\int_{(\mathbb{R}^d)^2}dx_2dx_3 a(x_3-x_2)b(x-x_2)\left[\delta_{mi}g_{kn}(x_3)+\delta_{ni}g_{mk}(x_3)\right].
\end{aligned}
\tag{196}
$$

Density independent death: $\{P,R,C\} = \{0,1,0\}$. Process definition: individuals of type j die with a rate r.

$$\begin{aligned}
(L_j^{\mathrm{D}}(r)F)(\gamma) &= r\sum_{x\in\gamma_j}(L_j^{x^-}F)(\gamma) \\
&= r\sum_{x_1^{(j)}\in\gamma}\Big(F(\gamma\backslash x_1^{(j)}) - F(\gamma)\Big) \\
H^{\mathrm{D}(r)}_{q_m,j} &= -\delta_{mj} r q_j; \\
H^{\mathrm{D}(r)}_{p_m,j} &= -\delta_{mj} r p_j; \\
H^{\mathrm{D}(r)}_{g_{mn},j}(x_1) &= -r\delta_{mj}g_{jn}(x_1) - r\delta_{nj}g_{mj}(x_1).
\end{aligned} \tag{197}$$

Death by external factor: $\{P,R,C\} = \{0,1,1\}$. Process definition: type k individuals induce death in type j individuals with kernel a.

$$\begin{aligned}
(L_{jk}^{\mathrm{DE}}(a)F)(\gamma) &= \sum_{x\in\gamma_j}\left(\sum_{y\in\gamma_k}a(x-y)\right)(L_j^{x^-}F)(\gamma) \\
&= \sum_{x_1^{(j)}\in\gamma}\sum_{x_2^{(k)}\in\gamma}\Big(F(\gamma\backslash x_1^{(j)}) - F(\gamma)\Big)a(x_1-x_2) \\
H^{\mathrm{DE}(a)}_{q_m,jk} &= -\delta_{mj}Aq_jq_k; \\
H^{\mathrm{DE}(a)}_{p_m,jk} &= \delta_{mj}\left(-A(q_jp_k+p_jq_k) - \int_{\mathbb{R}^d}dy g_{jk}(y)a(y)\right); \\
H^{\mathrm{DE}(a)}_{g_{mn},jk}(x_1) &= -a(x_1)q_jq_k\left[\delta_{mk}\delta_{nj}+\delta_{mj}\delta_{nk}\right] \\
&\quad -Aq_k\left[\delta_{mj}g_{jn}(x_1)+\delta_{nj}g_{mj}(x_1)\right] \\
&\quad -q_j\int_{\mathbb{R}^d}dx a(x_1-x)\left[\delta_{mj}g_{kn}(x)+\delta_{nj}g_{mk}(x)\right].
\end{aligned} \tag{198}$$

Death by competition: $\{P,R,C\} = \{0,1,1\}$. Process definition: type j individuals induce death in type j individuals with the kernel a.

$$\begin{aligned}
(L_j^{\mathrm{C}}(a)F)(\gamma) &= \sum_{x\in\gamma_j}\left(\sum_{y\in\gamma_j}a(x-y)\right)(L_j^{x^-}F)(\gamma) \\
&= \sum_{x_1^{(j)}\in\gamma}\sum_{x_2^{(j)}\in\gamma}\Big(F(\gamma\backslash x_1^{(j)}) - F(\gamma)\Big)a(x_1-x_2); \\
H^{\mathrm{C}(a)}_{q_m,j} &= -\delta_{mj}Aq_jq_j; \\
H^{\mathrm{C}(a)}_{p_m,j} &= \delta_{mj}\left(-A(q_jp_j+p_jq_j) - \int_{\mathbb{R}^d}dy g_{jj}(y)a(y)\right); \\
H^{\mathrm{C}(a)}_{g_{mn},j}(x_1) &= -\delta_{mj}\delta_{nj}2a(x_1)q_jq_j \\
&\quad -Aq_j\left[\delta_{mj}g_{jn}(x_1)+\delta_{nj}g_{mj}(x_1)\right] \\
&\quad -q_j\int_{\mathbb{R}^d}dx a(x_1-x)\left[\delta_{mj}g_{jn}(x)+\delta_{nj}g_{mj}(x)\right].
\end{aligned} \tag{199}$$

Change in type: $\{P,R,C\} = \{1,1,0\}$. Process definition: individuals of type j change spontaneously into type i ($j \neq i$) at rate r.

$$
\begin{aligned}
(L_{ij}^{\mathrm{CT}}(r)F)(\gamma) &= r\sum_{x\in\gamma_j} L_{ji}^{x^-x^+}F(\gamma)\\
&= r\sum_{x^{(j)}\in\gamma}\left(F(\gamma\backslash x^{(j)}\cup x^{(i)}) - F(\gamma)\right);\\
H_{q_m,ij}^{\mathrm{CT}(r)} &= (\delta_{mi}-\delta_{mj})rq_j;\\
H_{p_m,ij}^{\mathrm{CT}(r)} &= (\delta_{mi}-\delta_{mj})rp_j;\\
H_{g_{mn},ij}^{\mathrm{CT}(r)}(x_1) &= r\left[\delta_{mi}g_{jn}(x_1)+\delta_{ni}g_{mj}(x_1)\right]\\
&\quad -r\left[\delta_{mj}g_{jn}(x_1)+\delta_{nj}g_{mj}(x_1)\right]. \qquad (200)
\end{aligned}
$$

Jump and change in type: $\{P,R,C\} = \{1,1,0\}$. Process definition: type j individual jumps by the kernel a and changes to type i.

$$
\begin{aligned}
(L_{ij}^{\mathrm{JCT}}(a)F)(\gamma) &= \sum_{x\in\gamma_j}\int_{\mathbb{R}^d} a(x-y)(L_{ji}^{x^-y^+}F)(\gamma)dy\\
&= \sum_{x\in\gamma_j}\int_{\mathbb{R}^d} a(x-y)(F(\gamma\backslash x^{(j)}\cup y^{(i)}) - F(\gamma))dy;\\
H_{q_m,ij}^{\mathrm{JCT}(a)} &= q_jA\left(\delta_{mi}-\delta_{mj}\right);\\
H_{p_m,ij}^{\mathrm{JCT}(a)} &= p_jA\left(\delta_{mi}-\delta_{mj}\right);\\
H_{g_{mn},ij}^{\mathrm{JCT}(a)}(x_1) &= -A\left[\delta_{mj}g_{jn}(x_1)+\delta_{nj}g_{mj}(x_1)\right]\\
&\quad +\int_{\mathbb{R}^d} dx a(x_1-x)\left[\delta_{mi}g_{jn}(x)+\delta_{ni}g_{mj}(x)\right]. \qquad (201)
\end{aligned}
$$

Jump: $\{P,R,C\} = \{1,1,0\}$. Process definition: type i individual jumps by the kernel a.

$$
\begin{aligned}
(L_{i}^{\mathrm{J}}(a)F)(\gamma) &= \sum_{x\in\gamma_i}\int_{\mathbb{R}^d} a(x-y)(L_{ii}^{x^-y^+}F)(\gamma)dy\\
&= \int_{\mathbb{R}^d} dx_1 \sum_{x_2^{(i)}\in\gamma}\left(F(\gamma\backslash x_2^{(i)}\cup x_1^{(i)}) - F(\gamma)\right)a(x_1-x_2);\\
H_{q_m,i}^{\mathrm{J}(a)} &= 0;\\
H_{p_m,i}^{\mathrm{J}(a)} &= 0;\\
H_{g_{mn},i}^{\mathrm{J}(a)}(x_1) &= -A\left[\delta_{mi}g_{in}(x_1)+\delta_{ni}g_{mi}(x_1)\right]\\
&\quad +\int_{\mathbb{R}^d} dx a(x_1-x)\left[\delta_{mi}g_{in}(x)+\delta_{ni}g_{mi}(x)\right]. \qquad (202)
\end{aligned}
$$

Infection: $\{P,R,C\}=\{1,1,1\}$. Process description: individuals of type i change individuals of type j $(j\neq i)$ into type i with kernel a.

$$
\begin{aligned}
(L^{\mathrm{I}}_{ij}(a)F)(\gamma) &= \sum_{y\in\gamma_i}\sum_{x\in\gamma_j} a(x-y)(L^{x^-x^+}_{ji}F)(\gamma)\\
&= \sum_{x_2^{(j)}\in\gamma}\sum_{x_3^{(i)}\in\gamma}\Big(F(\gamma\backslash x_2^{(j)}\cup x_2^{(i)})-F(\gamma)\Big)a(x_2-x_3);\\
H^{\mathrm{I}(a)}_{q_m,ij} &= (\delta_{mi}-\delta_{mj})Aq_iq_j;\\
H^{\mathrm{I}(a)}_{p_m,ij} &= (\delta_{mi}-\delta_{mj})\left(A[q_ip_j+p_iq_j]+\int_{\mathbb{R}^d}dy\ a(y)g_{ij}(y)\right);\\
H^{\mathrm{I}(a)}_{g_{mn},ij}(x_1) &= a(x_1)q_jq_i\left[2\delta_{mi}\delta_{ni}-\delta_{mi}\delta_{nj}-\delta_{mj}\delta_{ni}\right]\\
&\quad +Aq_i\left[(\delta_{mi}-\delta_{mj})g_{jn}(x_1)+(\delta_{ni}-\delta_{nj})g_{mj}(x_1)\right]\\
&\quad +q_j\int_{\mathbb{R}^d}dxa(x_1-x)\left[(\delta_{mi}-\delta_{mj})g_{in}(x)+(\delta_{ni}-\delta_{nj})g_{mi}(x)\right]. \qquad (203)
\end{aligned}
$$

Birth to another type by consumption: $\{P,R,C\}=\{1,1,1\}$. Process definition: type j individuals 'die' when type k $(j\neq k)$ individuals are around with kernel a, and simultaneously type i individuals appear near type k individuals with kernel b. For example, type k may consume type j $(j\neq k)$ and give birth to type i.

$$
\begin{aligned}
(L^{\mathrm{BTC}}_{ijk}(a,b)F)(\gamma) &= \sum_{x_k\in\gamma_k}\sum_{x_j\in\gamma_j}\int_{\mathbb{R}^d}a(x_j-x_k)b(x_i-x_k)(L^{x_j^-x_i^+}_{ji}F)(\gamma)dx_i;\\
&= \sum_{x_k\in\gamma_k}\sum_{x_j\in\gamma_j}\int_{\mathbb{R}^d}a(x_j-x_k)b(x_i-x_k)(F(\gamma\backslash x_j^{(j)}\cup x_i^{(i)})-F(\gamma))dx_i;\\
H^{\mathrm{BTC}(a,b)}_{q_m,ijk} &= q_jq_kAB\left(\delta_{mi}-\delta_{mj}\right);\\
H^{\mathrm{BTC}(a,b)}_{p_m,ijk} &= (\delta_{mi}-\delta_{mj})\left[(p_jq_k+p_kq_j)AB+B\int_{\mathbb{R}^d}dxa(x)g_{jk}(x)\right];\\
H^{\mathrm{BTC}(a,b)}_{g_{mn},ijk}(x_1) &= Ab(x_1)q_jq_k\left[\delta_{mk}\delta_{ni}+\delta_{mi}\delta_{nk}\right]\\
&\quad -a(x_1)Bq_jq_k\left[\delta_{mk}\delta_{nj}+\delta_{mj}\delta_{nk}\right]\\
&\quad +q_k\int_{(\mathbb{R}^d)^2}dx_2dx_3a(x_2-x_3)b(x_1-x_3)\left[\delta_{mi}g_{jn}(x_2)+\delta_{ni}g_{mj}(x_2)\right]\\
&\quad +q_jA\int_{\mathbb{R}^d}dx_3b(x_1-x_3)\left[\delta_{mi}g_{kn}(x_3)+\delta_{ni}g_{mk}(x_3)\right]\\
&\quad -ABq_k\left[\delta_{mj}g_{jn}(x_1)+\delta_{nj}g_{mj}(x_1)\right]\\
&\quad -Bq_j\int_{\mathbb{R}^d}dxa(x_1-x)\left[\delta_{mj}g_{kn}(x)+\delta_{nj}g_{mk}(x)\right].
\end{aligned}
$$

(204)

Birth by consumption: $\{P,R,C\}=\{1,1,1\}$**.** Process definition: type j individuals 'die' when type i $(j\neq i)$ individuals are around with kernel a, and simultaneously type i new individuals

appear near type i individuals with kernel b.

$$
\begin{aligned}
(L_{ij}^{\mathrm{BC}}(a,b)F)(\gamma) &= \sum_{y\in\gamma_i}\sum_{z\in\gamma_j}\int_{\mathbb{R}^d} a(z-y)b(x-y)(L_{ji}^{z^-x^+}F)(\gamma)dx \\
&= \int_{\mathbb{R}^d} dx_1 \sum_{x_2^{(i)}\in\gamma}\sum_{x_3^{(j)}\in\gamma}\Big(F(\gamma\backslash x_3^{(j)}\cup x_1^{(i)})-F(\gamma)\Big)a(x_3-x_2)b(x_1-x_2); \\
H_{q_m,ij}^{\mathrm{BC}(a,b)} &= (\delta_{mi}-\delta_{mj})q_iq_jAB; \\
H_{p_m,ij}^{\mathrm{BC}(a,b)} &= \delta_{mi}\left((q_ip_j+p_iq_j)AB+B\int_{\mathbb{R}^d} dyg_{ij}(y)a(y)\right) \\
&\quad +\delta_{mj}\left(-(q_ip_j+p_iq_j)AB-B\int_{\mathbb{R}^d} dyg_{ij}(y)a(y)\right); \\
H_{g_{mn},ij}^{\mathrm{BC}(a,b)}(x_1) &= \delta_{mi}\delta_{ni}2Ab(x_1)q_jq_i \\
&\quad -a(x_1)Bq_jq_i\left[\delta_{mi}\delta_{nj}+\delta_{mj}\delta_{ni}\right] \\
&\quad +q_i\int_{(\mathbb{R}^d)^2} dx_2dx_3a(x_2-x_3)b(x_1-x_3)\left[\delta_{mi}g_{jn}(x_2)+\delta_{ni}g_{mj}(x_2)\right] \\
&\quad +q_jA\int_{\mathbb{R}^d} dx_3b(x_1-x_3)\left[\delta_{mi}g_{in}(x_3)+\delta_{ni}g_{mi}(x_3)\right] \\
&\quad -ABq_i\left[\delta_{mj}g_{jn}(x_1)+\delta_{nj}g_{mj}(x_1)\right] \\
&\quad -Bq_j\int_{\mathbb{R}^d} dxa(x_1-x)\left[\delta_{mj}g_{in}(x)+\delta_{nj}g_{mi}(x)\right].
\end{aligned}
\tag{205}
$$

Change in type by consumption: $\{P,R,C\}=\{1,2,0\}$. Process definition: type j_1 individual changes to type i ($i\neq j_1$) individual by consuming a type j_2 ($j_2\neq i, j_2\neq j_1$) individual with kernel a.

$$
\begin{aligned}
(L_{ij_1j_2}^{\mathrm{CTC}}(a)F)(\gamma) &= \sum_{y\in\gamma_{j_1}}\sum_{x\in\gamma_{j_2}} a(x-y)(L_{j_2j_1i}^{x^-y^-y^+}F)(\gamma) \\
&= \sum_{x_1^{(j_1)}\in\gamma}\sum_{x_2^{(j_2)}\in\gamma}\Big(F(\gamma\backslash x_2^{(j_2)}\backslash x_1^{(j_1)}\cup x_1^{(i)})-F(\gamma)\Big)a(x_1-x_2); \\
H_{q_m,ij_1j_2}^{\mathrm{CTC}(a)} &= (\delta_{mi}-\delta_{mj_1}-\delta_{mj_2})\,q_{j_2}q_{j_1}A; \\
H_{p_m,ij_1j_2}^{\mathrm{CTC}(a)} &= (\delta_{mi}-\delta_{mj_1}-\delta_{mj_2})\left(\int_{\mathbb{R}^d} dya(y)g_{j_1j_2}(y)+(q_{j_2}p_{j_1}+q_{j_1}p_{j_2})A\right); \\
H_{g_{mn},ij_1j_2}^{\mathrm{CTC}(a)}(x_1) &= -a(x_1)q_{j_1}q_{j_2}\left[\delta_{mj_1}\delta_{nj_2}+\delta_{mj_2}\delta_{nj_1}\right] \\
&\quad +Aq_{j_2}\left[(\delta_{mi}-\delta_{mj_1})g_{j_1n}(x_1)+(\delta_{ni}-\delta_{nj_1})g_{mj_1}(x_1)\right] \\
&\quad +q_{j_1}\int_{\mathbb{R}^d} dxa(x_1-x)\left[(\delta_{mi}-\delta_{mj_1})g_{j_2n}(x)+(\delta_{ni}-\delta_{nj_1})g_{mj_2}(x)\right] \\
&\quad -q_{j_2}\int_{\mathbb{R}^d} dxa(x_1-x)\left[\delta_{mj_2}g_{j_1n}(x)+\delta_{nj_2}g_{mj_1}(x)\right] \\
&\quad -Aq_{j_1}\left[\delta_{mj_2}g_{j_2n}(x_1)+\delta_{nj_2}g_{mj_2}(x_1)\right].
\end{aligned}
\tag{206}
$$

1.5 Transformation of equations for H_q, H_p and H_g for the use in toolboxes

In this section the expressions (181), (184) and (188) are transformed into the form which is used to program the algorithm to obtain functions H_q, H_p and H_g for analytical and numerical calculations in a complex process consisting of an arbitrary number of basic processes.

1.5.1 Simplifications based on symmetry properties of the interaction function.

Using the symmetry properties of the interaction function $r(\mathbf{P},\mathbf{R},\mathbf{C})$, the expressions derived can be simplified. The expressions contain the integral of the interaction function $r(\mathbf{P},\mathbf{R},\mathbf{C})$ over all locations of points except the two: except the location x_{N_n}, i.e. the n-th point in the group N (here $N \in \{i,j,k\}$) which is substituted by the location x_1, and except the location x_{M_m}, i.e. the m-th point in the list M (here $M \in \{i,j,k\}$) which is substituted by the location x_2,

$$\int_{(\mathbb{R}^d)^{P+R+C}} \prod_{\alpha\in\{i_1,\dots,k_C\}} dx_\alpha r(\mathbf{P},\mathbf{R},\mathbf{C})\delta(x_{N_n}-x_1)\delta(x_{M_m}-x_2). \tag{207}$$

Denoting the subscript N_n by β_1, and M_m by β_2, such an integral, denoted as $C_2(x_{\beta_1},x_1;x_{\beta_2},x_2)$, can be written in a general form as follows:

$$C_2(x_{\beta_1},x_1;x_{\beta_2},x_2) \equiv \int_{(\mathbb{R}^d)^{P+R+C}} \prod_{\alpha\in\{i_1,\dots,k_C\}} dx_\alpha r(\mathbf{P},\mathbf{R},\mathbf{C})\delta(x_{\beta_1}-x_1)\delta(x_{\beta_2}-x_2). \tag{208}$$

Here, in the notation $C_2(x_{\beta_1},x_1;x_{\beta_2},x_2)$ the pair of arguments x_{β_1} and x_{β_1} and the pair of arguments x_1 and x_2 have a different meaning. Variables x_{β_1} and x_{β_2} denote full set of coordinates of points from three groups $\mathbf{P},\mathbf{R},\mathbf{C}$, over which the integration is not performed, whereas variables x_1 and x_2 define locations in d-dimensional space $\mathbb{R}^d$.

Taking into account, that the interaction function $r(\mathbf{P},\mathbf{R},\mathbf{C})$ is symmetric with respect to transformations which preserve distances between points, the same holds for $C_2(x_{\beta_1},x_1;x_{\beta_2},x_2)$. Indeed,

$$C_2(x_{\beta_1},x_1;x_{\beta_2},x_2) = C_2(x_{\beta_1},x_1+X;x_{\beta_2},x_2+X), \tag{209}$$

which follows from the observation that the integral C_2 does not change if all variables of integration are shifted by X or by $-X$, where $X \in \mathbb{R}^d$. Due to the symmetry of $r(\mathbf{P},\mathbf{R},\mathbf{C})$ the function C_2 is also symmetric with respect to inversion of coordinates:

$$C_2(x_{\beta_1},x_1;x_{\beta_2},x_2) = C_2(x_{\beta_1},-x_1;x_{\beta_2},-x_2), \tag{210}$$

and C_2 is also rotationally symmetric. As a result, $C_2(x_{\beta_1},x_1;x_{\beta_2},x_2)$ depends only on the distance between x_1 and x_2,

$$C_2(x_{\beta_1},x_1;x_{\beta_2},x_2) = C_2(x_{\beta_1},0;x_{\beta_2},|x_1-x_2|) = C_2(x_{\beta_1},|x_1-x_2|;x_{\beta_2},0). \tag{211}$$

Taking these properties into account, it is reasonable to introduce the following notation:

$$C(x_{\beta_1},x_{\beta_2};x) \equiv C_2(x_{\beta_1},x;x_{\beta_2},0) = C_2(x_{\beta_1},0;x_{\beta_2},x). \tag{212}$$

When the integration over the location of only a single point is removed, e.g. as in the following expression,

$$\int_{(\mathbb{R}^d)^{P+R+C}} \prod_{\alpha\in\{i_1,\dots,k_C\}} dx_\alpha r(\mathbf{P},\mathbf{R},\mathbf{C})\delta(x_\beta), \tag{213}$$

the value of such an integral is constant. Therefore, we introduce the following notations:

$$C(x_\beta, 0; 0) \equiv \int_{(\mathbb{R}^d)^{P+R+C}} \prod_{\alpha\in\{i_1,\dots,k_C\}} dx_\alpha r(\mathbf{P},\mathbf{R},\mathbf{C})\delta(x_\beta); \tag{214}$$

$$C(x_{\beta_1}, x_{\beta_2}; X) \equiv \int_{(\mathbb{R}^d)^{P+R+C}} \prod_{\alpha\in\{i_1,\dots,k_C\}} dx_\alpha r(\mathbf{P},\mathbf{R},\mathbf{C})\delta(x_{\beta_1})\delta(x_{\beta_2}-X). \tag{215}$$

Formally, if in the last equation $\beta_2 = \beta_1$, then one has:

$$C(x_{\beta_1}, x_{\beta_1}; X) = \delta(X)C(x_{\beta_1}, 0; 0). \tag{216}$$

Detailed transformations of different terms in equations (181), (184) and (188) for functions H_q, H_p and H_g are shown below. All such terms are considered as groups 1, 2a, 2b, 3a and 3b.

Group 1, integrals in H_g.

$$\begin{aligned} C(x_i, x_{i'}; x_1) &= \int_{(\mathbb{R}^d)^{P+R+C}} \prod_{\alpha\in\{i_1,\dots,k_C\}} dx_\alpha r(\mathbf{P},\mathbf{R},\mathbf{C})\delta(x_i - x_1)\delta(x_{i'}) \\ &= \int_{(\mathbb{R}^d)^{P+R+C}} \prod_{\alpha\in\{i_1,\dots,k_C\}} dx_\alpha r(\mathbf{P},\mathbf{R},\mathbf{C})\delta(x_i)\delta(x_{i'} - x_1). \end{aligned} \tag{217}$$

$$\begin{aligned} C(x_j, x_{j'}; x_1) &= \int_{(\mathbb{R}^d)^{P+R+C}} \prod_{\alpha\in\{i_1,..,k_C\}} dx_\alpha r(\mathbf{P},\mathbf{R},\mathbf{C})\delta(x_j - x_1)\delta(x_{j'}) \\ &= \int_{(\mathbb{R}^d)^{P+R+C}} \prod_{\alpha\in\{i_1,..,k_C\}} dx_\alpha r(\mathbf{P},\mathbf{R},\mathbf{C})\delta(x_j)\delta(x_{j'} - x_1). \end{aligned} \tag{218}$$

$$\begin{aligned} C(x_k, x_i; x_1) &= \int_{(\mathbb{R}^d)^{P+R+C}} \prod_{\alpha\in\{i_1,..,k_C\}} dx_\alpha r(\mathbf{P},\mathbf{R},\mathbf{C})\delta(x_k - x_1)\delta(x_i) \\ &= \int_{(\mathbb{R}^d)^{P+R+C}} \prod_{\alpha\in\{i_1,..,k_C\}} dx_\alpha r(\mathbf{P},\mathbf{R},\mathbf{C})\delta(x_k)\delta(x_i - x_1). \end{aligned} \tag{219}$$

$$\begin{aligned} C(x_k, x_j; x_1) &= \int_{(\mathbb{R}^d)^{P+R+C}} \prod_{\alpha\in\{i_1,..,k_C\}} dx_\alpha r(\mathbf{P},\mathbf{R},\mathbf{C})\delta(x_k - x_1)\delta(x_j) \\ &= \int_{(\mathbb{R}^d)^{P+R+C}} \prod_{\alpha\in\{i_1,..,k_C\}} dx_\alpha r(\mathbf{P},\mathbf{R},\mathbf{C})\delta(x_k)\delta(x_j - x_1). \end{aligned} \tag{220}$$

Group 2a, integrals in H_g. Here we consider the following integral,

$$\int_{(\mathbb{R}^d)^{P+R+C}} \prod_{\alpha\in\{i_1,\dots,k_C\}} dx_\alpha r(\mathbf{P},\mathbf{R},\mathbf{C})\delta(x_{\beta_0})g_{m\beta_1}(x_1 - x_{\beta_1}),$$

where $\beta_0 \in \{i_1, ..., j_R\}$, and $\beta_1 \in \{j_1, ..., k_C\}$. By definition all variables of integration x_α are considered to be independent variables. However, the interaction function $r(\mathbf{P}, \mathbf{R}, \mathbf{C})$ may contain a constraint implying that x_{β_0} and x_{β_1} denote the same variable, i.e. formally $\delta_{x_{\beta_0} x_{\beta_1}} = 1$, where δ_{ij} is the Kronecker delta function. Therefore, one should consider both cases when x_{β_0} and x_{β_1} denote the same or different variables of integration. First, we consider the case when x_{β_0} and x_{β_1} denote different variables. The integral becomes:

$$\begin{aligned}
&\int_{(\mathbb{R}^d)^{P+R+C-1}} dx_{\beta_1} \prod_{\alpha \in \{i_1,...,k_C\} \backslash \beta_0 \backslash \beta_1} dx_\alpha \, r(\mathbf{P}, \mathbf{R}, \mathbf{C})|_{x_{\beta_0} \to 0} \, g_{m\beta_1}(x_1 - x_{\beta_1}) \\
&= \int_{\mathbb{R}^d} dX C_2(x_{\beta_1}, X; x_{\beta_0}, 0) g_{m\beta_1}(x_1 - X) \\
&= \int_{\mathbb{R}^d} dX C_2(x_{\beta_1}, X + x_1; x_{\beta_0}, 0) g_{m\beta_1}(X) \\
&= \int_{\mathbb{R}^d} dX C(x_{\beta_0}, x_{\beta_1}; X + x_1) g_{m\beta_1}(X).
\end{aligned} \tag{221}$$

In the second case, when x_{β_0} and x_{β_1} denote the same variables, i.e.when $\delta_{x_{\beta_0} x_{\beta_1}} = 1$, the integral becomes:

$$\int_{(\mathbb{R}^d)^{P+R+C-1}} \prod_{\alpha \in \{i_1,...,k_C\} \backslash \beta_0} dx_\alpha \, r(\mathbf{P}, \mathbf{R}, \mathbf{C})|_{x_{\beta_0} \to 0} \, g_{m\beta_1}(x_1) = g_{m\beta_1}(x_1) C(x_{\beta_0}, 0; 0). \tag{222}$$

Group 2b, integrals in H_g. Here we consider the following integral,

$$\int_{(\mathbb{R}^d)^{P+R+C}} \prod_{\alpha \in \{i_1,...,k_C\}} dx_\alpha r(\mathbf{P}, \mathbf{R}, \mathbf{C}) \delta(x_{\beta_0} - x_1) g_{\beta_1 n}(x_{\beta_1}),$$

where $\beta_0 \in \{i_1, ..., k_C\}$, and $\beta_1 \in \{j_1, ..., k_C\}$. If x_{β_0} and x_{β_1} denote different variables, we have

$$\begin{aligned}
&\int_{(\mathbb{R}^d)^{P+R+C-1}} dx_{\beta_1} \prod_{\alpha \in \{i_1,...,k_C\} \backslash \beta_0 \backslash \beta_1} dx_\alpha \, r(\mathbf{P}, \mathbf{R}, \mathbf{C})|_{x_{\beta_0} \to x_1} \, g_{\beta_1 n}(x_{\beta_1}) \\
&= \int_{\mathbb{R}^d} dX C_2(x_{\beta_1}, X; x_{\beta_0}, x_1) g_{\beta_1 n}(X) = \int_{\mathbb{R}^d} dX C_2(x_{\beta_1}, X + x_1; x_{\beta_0}, 0) g_{\beta_1 n}(X) \\
&= \int_{\mathbb{R}^d} dX C(x_{\beta_0}, x_{\beta_1}; X + x_1) g_{\beta_1 n}(X).
\end{aligned} \tag{223}$$

If x_{β_0} and x_{β_1} denote the same variable, i.e. if $\delta_{x_{\beta_0} x_{\beta_1}} = 1$, then the expression becomes:

$$\begin{aligned}
&\int_{(\mathbb{R}^d)^{P+R+C-1}} \prod_{\alpha \in \{i_1,...,k_C\} \backslash \beta_0} dx_\alpha \, r(\mathbf{P}, \mathbf{R}, \mathbf{C})|_{x_{\beta_0} \to x_1} \, g_{\beta_1 n}(x_1) = g_{\beta_1 n}(x_1) C_2(x_{\beta_0}, x_1; 0, 0) \\
&= g_{\beta_1 n}(x_1) C(x_{\beta_0}, 0; 0).
\end{aligned} \tag{224}$$

Notice, the contribution of integrals from the groups 2a and 2b is possible if there are at least two different locations in the groups $\mathbf{P}, \mathbf{R}$ and $\mathbf{C}$ of the process. As a result, the corresponding expression in H_g becomes:

$$
\begin{aligned}
&\int_{(\mathbb{R}^d)^{P+R+C}} \prod_{\alpha\in\{i_1,\dots,k_C\}} dx_\alpha r(\mathbf{P},\mathbf{R},\mathbf{C}) \times \qquad (225)\\
&\times\left\{\sum_{i=i_1}^{i_P}\sum_{\beta_1\in\{j_1,\dots k_C\}}\left[\delta_{mi}\delta(x_i-x_1)g_{\beta_1 n}(x_{\beta_1})+\delta_{ni}\delta(x_i)g_{m\beta_1}(x_1-x_{\beta_1})\right]\prod_{\beta_\zeta\in\{j_1,\dots k_C\}\setminus\beta_1} q_{\beta_\zeta}\right.\\
&\left.-\sum_{j=j_1}^{j_R}\sum_{\beta_1\in\{j_1,\dots k_C\}}\left[\delta_{mj}\delta(x_j-x_1)g_{\beta_1 n}(x_{\beta_1})+\delta_{nj}\delta(x_j)g_{m\beta_1}(x_1-x_{\beta_1})\right]\prod_{\beta_\zeta\in\{j_1,\dots k_C\}\setminus\beta_1} q_{\beta_\zeta}\right\}=\\
&=\sum_{\beta_1\in\{j_1,\dots k_C\}}\left(\prod_{\beta_\zeta\in\{j_1,\dots k_C\}\setminus\beta_1} q_{\beta_\zeta}\right)\times\\
&\times\left\{\sum_{i=i_1}^{i_P}\delta_{mi}\left[\delta_{x_i x_{\beta_1}}g_{\beta_1 n}(x_1)C(x_i,0;0)+(1-\delta_{x_i x_{\beta_1}})\int_{\mathbb{R}^d}dXC(x_i,x_{\beta_1};X+x_1)g_{\beta_1 n}(X)\right]\right.\\
&+\sum_{i=i_1}^{i_P}\delta_{ni}\left[\delta_{x_i x_{\beta_1}}g_{m\beta_1}(x_1)C(x_i,0;0)+(1-\delta_{x_i x_{\beta_1}})\int_{\mathbb{R}^d}dXC(x_i,x_{\beta_1};X+x_1)g_{m\beta_1}(X)\right]\\
&-\sum_{j=j_1}^{j_R}\delta_{mj}\left[\delta_{x_j x_{\beta_1}}g_{\beta_1 n}(x_1)C(x_j,0;0)+(1-\delta_{x_j x_{\beta_1}})\int_{\mathbb{R}^d}dXC(x_j,x_{\beta_1};X+x_1)g_{\beta_1 n}(X)\right]\\
&\left.-\sum_{j=j_1}^{j_R}\delta_{nj}\left[\delta_{x_j x_{\beta_1}}g_{m\beta_1}(x_1)C(x_j,0;0)+(1-\delta_{x_j x_{\beta_1}})\int_{\mathbb{R}^d}dXC(x_j,x_{\beta_1};X+x_1)g_{m\beta_1}(X)\right]\right\}.
\end{aligned}
$$

Group 3a, integrals in H_p. Here, consider the following integral,

$$
I_{3\text{a}} = \int_{(\mathbb{R}^d)^{P+R+C}} \prod_{\alpha\in\{i_1,\dots,k_C\}} dx_\alpha r(\mathbf{P},\mathbf{R},\mathbf{C})\delta(x_{\beta_0})g_{\beta_1\beta_2}(x_{\beta_1}-x_{\beta_2}), \qquad (226)
$$

were $\beta_0\in\{i_1,\dots,k_C\}$, and $\beta_1,\beta_2\in\{j_1,\dots,k_C\}$. One can notice that x_{β_1} and x_{β_2} always denote different variables of integration, as among all locations in groups $\mathbf{R}$ and $\mathbf{C}$ there cannot be two or more identical locations. Here we consider the case when x_{β_0} denotes a variable which is different from both x_{β_1} and x_{β_2}. We change variables, $x_{\beta_1}\to x_{\beta_2}+X$, and obtain

$$
I_{3\text{a}} = \int_{\mathbb{R}^d} dX \prod_{\alpha\in\{i_1,\dots,k_C\}\setminus\beta_0\setminus\beta_1} dx_\alpha g_{\beta_1\beta_2}(X)\left(r(\mathbf{P},\mathbf{R},\mathbf{C})\big|_{\substack{x_{\beta_0}=0\\ x_{\beta_1}=x_{\beta_2}+X}}\right). \qquad (227)
$$

We use properties of $r(\mathbf{P},\mathbf{R},\mathbf{C})$ and shift all points by $-x_{\beta_2}$, i.e. a location x_α becomes $x_\alpha-x_{\beta_2}$. As a result, the argument x_{β_2} in $r(\mathbf{P},\mathbf{R},\mathbf{C})$ becomes equal zero (instead of x_{β_0} being equal zero), and x_{β_1} becomes equal X. The initial integration over x_{β_2} becomes effectively the integration over x_{β_0} due to the fact that after the shift the variable x_{β_0} equals to $-x_{\beta_2}$. Re-naming the initial integration over x_{β_2} as integration over x_{β_0}, the resulting integration is performed over all locations except

x_{β_1} and x_{β_2}.

$$
\begin{aligned}
I_{3\mathrm{a}} &= \int_{\mathbb{R}^d} dX \prod_{\alpha\in\{i_1,\dots,k_C\}\setminus\beta_1\setminus\beta_2} dx_\alpha g_{\beta_1\beta_2}(X)\left(r(\mathbf{P},\mathbf{R},\mathbf{C})\big|_{\substack{x_{\beta_1}=X\\ x_{\beta_2}=0}}\right)\\
&= \int_{\mathbb{R}^d} dX g_{\beta_1\beta_2}(X)\int_{(\mathbb{R}^d)^{P+R+C}} \prod_{\alpha\in\{i_1,\dots,k_C\}} dx_\alpha r(\mathbf{P},\mathbf{R},\mathbf{C})\delta(x_{\beta_1}-X)\delta(x_{\beta_2}-0)\\
&= \int_{\mathbb{R}^d} dX g_{\beta_1\beta_2}(X) C_2(x_{\beta_1},X;x_{\beta_2},0)\\
&= \int_{\mathbb{R}^d} dX g_{\beta_1\beta_2}(X) C(x_{\beta_1},x_{\beta_2};X). \qquad (228)
\end{aligned}
$$

Group 3b, integrals in H_p. We consider the integral (226) where the variable of integration x_{β_0} equals either to x_{β_1} or to x_{β_2}. If the variable x_{β_0} is the same as the variable x_{β_1}, then the integral becomes:

$$
\begin{aligned}
I_{3\mathrm{b}} &= \int_{(\mathbb{R}^d)^{P+R+C-1}} \prod_{\alpha\in\{i_1,\dots,k_C\}\setminus\beta_1} dx_\alpha\, r(\mathbf{P},\mathbf{R},\mathbf{C})\big|_{x_{\beta_1}=0} g_{\beta_1\beta_2}(-x_{\beta_2}) =\\
&= \int_{(\mathbb{R}^d)^{P+R+C-1}} dX \prod_{\alpha\in\{i_1,\dots,k_C\}\setminus\beta_1\setminus\beta_2} dx_\alpha\, r(\mathbf{P},\mathbf{R},\mathbf{C})\big|_{\substack{x_{\beta_1}=0\\ x_{\beta_2}=X}} g_{\beta_1\beta_2}(X)\\
&= \int_{\mathbb{R}^d} dX C_2(x_{\beta_2},X;x_{\beta_1},0) g_{\beta_1\beta_2}(X)\\
&= \int_{\mathbb{R}^d} dX C(x_{\beta_1},x_{\beta_2};X) g_{\beta_1\beta_2}(X). \qquad (229)
\end{aligned}
$$

If the variable x_{β_0} is the same as the variable x_{β_2}, then the integral becomes:

$$
\begin{aligned}
I'_{3\mathrm{b}} &= \int_{(\mathbb{R}^d)^{P+R+C-1}} \prod_{\alpha\in\{i_1,\dots,k_C\}\setminus\beta_2} dx_\alpha\, r(\mathbf{P},\mathbf{R},\mathbf{C})\big|_{x_{\beta_2}=0} g_{\beta_1\beta_2}(x_{\beta_1}) =\\
&= \int_{(\mathbb{R}^d)^{P+R+C-1}} dX \prod_{\alpha\in\{i_1,\dots,k_C\}\setminus\beta_1\setminus\beta_2} dx_\alpha\, r(\mathbf{P},\mathbf{R},\mathbf{C})\big|_{\substack{x_{\beta_1}=X\\ x_{\beta_2}=0}} g_{\beta_1\beta_2}(X)\\
&= \int_{\mathbb{R}^d} dX C_2(x_{\beta_1},X;x_{\beta_2},0) g_{\beta_1\beta_2}(X)\\
&= \int_{\mathbb{R}^d} dX C(x_{\beta_1},x_{\beta_2};X) g_{\beta_1\beta_2}(X). \qquad (230)
\end{aligned}
$$

Notice, that the non-zero contribution from integrals 3a or 3b is possible if at least both x_{β_1} and x_{β_2} exist. Therefore in the process there should be at least two different locations in groups $\mathbf{R}$ and $\mathbf{C}$ jointly. As a result, the integral in the expression for H_p becomes:

$$
\begin{aligned}
&\int_{(\mathbb{R}^d)^{P+R+C}} \prod_{\alpha\in\{i_1,\dots,k_C\}} dx_\alpha r(\mathbf{P},\mathbf{R},\mathbf{C})\left(\sum_{i=i_1}^{i_P}\delta_{mi}\delta(x_i)-\sum_{j=j_1}^{j_R}\delta_{mj}\delta(x_j)\right)\times\\
&\times\left(\sum_{\beta_1=j_1}^{k_C}\sum_{\beta_2=\beta_1+1}^{k_C} g_{\beta_1\beta_2}(x_{\beta_1}-x_{\beta_2})\prod_{\beta_\zeta\in\{j_1,\dots k_C\}\setminus\beta_1\setminus\beta_2} q_{\beta_\zeta}\right)=\\
&= \sum_{\beta_1=j_1}^{k_C}\sum_{\beta_2=\beta_1+1}^{k_C}\prod_{\alpha\in\{j_1,\dots k_C\}\setminus\beta_1\setminus\beta_2} q_\alpha\left(\sum_{i=i_1}^{i_P}\delta_{mi}-\sum_{j=j_1}^{j_R}\delta_{mj}\right)\int_{\mathbb{R}^d} dX C(x_{\beta_1},x_{\beta_2};X) g_{\beta_1\beta_2}(X).
\end{aligned}
$$

(231)

1.5.2 Equations in the real space

Applying transformations discussed in the previous section to equations (181), (184) and (188) for functions H_q, H_p and H_g we obtain the following expressions. Here, we would like to recall, that species are denoted by indices m, n and i, j, k with subscripts, whereas the locations of individuals are denoted by x with subscripts corresponding to species, e.g. x_β, x_{i_1}, etc.

$$\frac{\partial q_m}{\partial t} = \left(\prod_{\alpha\in\{j_1,\dots k_C\}} q_{s_\alpha}\right)\left(\sum_{i=i_1}^{i_P}\delta_{mi}C(x_i,0;0) - \sum_{j=j_1}^{j_R}\delta_{mj}C(x_j,0;0)\right); \tag{232}$$

$$\begin{aligned}\frac{\partial p_m}{\partial t} &= \left(\sum_{i=i_1}^{i_P}\delta_{mi}C(x_i,0;0) - \sum_{j=j_1}^{j_R}\delta_{mj}\delta(x_j)C(x_j,0;0)\right)\sum_{\beta_1\in\{j_1,\dots k_C\}} p_{\beta_1}\left(\prod_{\alpha\in\{j_1,\dots k_C\}\backslash\beta_1} q_\alpha\right)\\ &+ \sum_{\beta_1=j_1}^{k_C}\sum_{\beta_2=\beta_1+1}^{k_C}\prod_{\alpha\in\{j_1,\dots k_C\}\backslash\beta_1\backslash\beta_2} q_\alpha\left(\sum_{i=i_1}^{i_P}\delta_{mi} - \sum_{j=j_1}^{j_R}\delta_{mj}\right)\int_{\mathbb{R}^d} dX C(x_{\beta_1},x_{\beta_2};X)g_{\beta_1\beta_2}(X);\end{aligned} \tag{233}$$

$$\begin{aligned}\frac{\partial g_{mn}(x_1)}{\partial t} &= \Bigg[\sum_{i=i_1}^{i_P}\sum_{i'=i+1}^{i_P}(\delta_{mi}\delta_{ni'} + \delta_{mi'}\delta_{ni})\,C(x_i,x_{i'};x_1)\\ &\quad+\sum_{j=j_1}^{j_R}\sum_{j'=j+1}^{j_R}(-\delta_{mj}\delta_{nj'} - \delta_{mj'}\delta_{nj})\,C(x_j,x_{j'};x_1)\\ &\quad+\sum_{k=k_1}^{k_C}\sum_{i=i_1}^{i_P}(\delta_{mk}\delta_{ni} + \delta_{mi}\delta_{nk})\,C(x_k,x_i;x_1)\\ &\quad+\sum_{k=k_1}^{k_C}\sum_{j=j_1}^{j_R}(-\delta_{mk}\delta_{nj} - \delta_{mj}\delta_{nk})\,C(x_k,x_j;x_1)\Bigg]\left(\prod_{z\in\{j_1,\dots,k_C\}} q_z\right)\\ &+ \sum_{\beta_1\in\{j_1,\dots k_C\}}\left(\prod_{z\in\{j_1,\dots k_C\}\backslash\beta_1} q_z\right)\times\\ &\quad\times\Bigg\{\sum_{i=i_1}^{i_P}\delta_{mi}\delta_{x_ix_{\beta_1}}g_{\beta_1 n}(x_1)C(x_i,0;0)\\ &\quad+\sum_{i=i_1}^{i_P}\delta_{mi}(1-\delta_{x_ix_{\beta_1}})\int_{\mathbb{R}^d} dX C(x_i,x_{\beta_1};X+x_1)g_{\beta_1 n}(X)\\ &\quad+\sum_{i=i_1}^{i_P}\delta_{ni}\delta_{x_ix_{\beta_1}}g_{m\beta_1}(x_1)C(x_i,0;0)\\ &\quad+\sum_{i=i_1}^{i_P}\delta_{ni}(1-\delta_{x_ix_{\beta_1}})\int_{\mathbb{R}^d} dX C(x_i,x_{\beta_1};X+x_1)g_{m\beta_1}(X)\\ &\quad-\sum_{j=j_1}^{j_R}\delta_{mj}\delta_{x_jx_{\beta_1}}g_{\beta_1 n}(x_1)C(x_j,0;0)-\end{aligned}$$

$$
\begin{aligned}
&- \sum_{j=j_1}^{j_R} \delta_{mj}(1-\delta_{x_j x_{\beta_1}}) \int_{\mathbb{R}^d} dX C(x_j, x_{\beta_1}; X + x_1) g_{\beta_1 n}(X) \\
&- \sum_{j=j_1}^{j_R} \delta_{nj}\delta_{x_j x_{\beta_1}} g_{m\beta_1}(x_1) C(x_j, 0; 0) \\
&- \sum_{j=j_1}^{j_R} \delta_{nj}(1-\delta_{x_j x_{\beta_1}}) \int_{\mathbb{R}^d} dX C(x_j, x_{\beta_1}; X + x_1) g_{m\beta_1}(X) \Bigg\}. \qquad (234)
\end{aligned}
$$

1.5.3 Equations in the Fourier space

The function $f(x)$ and its Fourier transform $\tilde{f}(k)$ are connected by the following transformations, here $x, k \in \mathbb{R}^d$,

$$
\tilde{f}(k) = \int_{\mathbb{R}^d} dx f(x) \mathrm{e}^{-\mathrm{i}2\pi kx}; \qquad (235)
$$

$$
f(x) = \int_{\mathbb{R}^d} dk \tilde{f}(k) \mathrm{e}^{\mathrm{i}2\pi kx}. \qquad (236)
$$

Using the same notations, $\tilde{C}(x_{\beta_1}, x_{\beta_2}; k)$ and $\tilde{g}(k)$ denote Fourier transforms of $C(x_{\beta_1}, x_{\beta_2}; x)$ and $g(x)$ respectively. The constant $\tilde{C}(x_\beta, 0; 0)$ equals to the constant $C(x_\beta, 0; 0)$ expressed in terms of Fourier transforms of the interaction function, e.g. in the case of a single kernel a using the following equality $\int_{\mathbb{R}^d} a(x)dx = \tilde{a}(0)$.

Equations for q_m and p_m expressed in terms of functions in Fourier space have the following form:

$$
\frac{\partial q_m}{\partial t} = \left(\prod_{\alpha \in \{j_1, \dots k_C\}} q_\alpha \right) \left(\sum_{i=i_1}^{i_P} \delta_{mi} \tilde{C}(x_i, 0; 0) - \sum_{j=j_1}^{j_R} \delta_{mj} \tilde{C}(x_j, 0; 0) \right); \qquad (237)
$$

$$
\begin{aligned}
\frac{\partial p_m}{\partial t} &= \left(\sum_{i=i_1}^{i_P} \delta_{mi} \tilde{C}(x_i, 0; 0) - \sum_{j=j_1}^{j_R} \delta_{mj} \delta(x_j) \tilde{C}(x_j, 0; 0) \right) \sum_{\beta_1 \in \{j_1, \dots k_C\}} p_{s_{\beta_1}} \left(\prod_{\alpha \in \{j_1, \dots k_C\} \backslash \beta_1} q_\alpha \right) \\
&+ \sum_{\beta_1 = j_1}^{k_C} \sum_{\beta_2 = \beta_1 + 1}^{k_C} \left(\prod_{\alpha \in \{j_1, \dots k_C\} \backslash \beta_1 \backslash \beta_2} q_\alpha \right) \times \\
&\times \left\{ \left(\sum_{i=i_1}^{i_P} \delta_{mi} - \sum_{j=j_1}^{j_R} \delta_{mj} \right) \int_{\mathbb{R}^d} dk \tilde{C}(x_{\beta_1}, x_{\beta_2}; k) \tilde{g}_{\beta_1 \beta_2}(k) \right\}. \qquad (238)
\end{aligned}
$$

The integral present in the equation (238) can be simplified taking into account, that Fourier transforms of g and C are symmetric functions, $\tilde{g}_{mn}(k) = \tilde{g}_{mn}(|k|)$, $\tilde{C}(k) = \tilde{C}(|k|)$. Thus, in spaces with dimensionality $d = 2$ or $d = 3$ one can use polar or spherical system of coordinates and perform integration over angles:

$$
\int_{\mathbb{R}^d} dk \tilde{C}(x_{\beta_1}, x_{\beta_2}; k) \tilde{g}_{\beta_1 \beta_2}(k) = \int_R d|k| I_{|k|} \tilde{C}(x_{\beta_1}, x_{\beta_2}; |k|) \tilde{g}_{\beta_1 \beta_2}(|k|), \qquad (239)
$$

where

$$
I_{|k|} = \begin{cases} 1, & d = 1; \\ 2\pi|k|, & d = 2; \\ 4\pi|k|^2, & d = 3. \end{cases} \qquad (240)
$$

Equation for $\tilde{g}_{mn}(k)$ has the following form:

$$
\begin{aligned}
\frac{\partial \tilde{g}_{mn}(k)}{\partial t} = & \Bigg\{\Bigg[\sum_{i=i_1}^{i_P}\sum_{i'=i+1}^{i_P}\left(\delta_{mi}\delta_{ni'}+\delta_{mi'}\delta_{ni}\right)\tilde{C}(x_i,x_{i'};k) \\
& +\sum_{j=j_1}^{j_R}\sum_{j'=j+1}^{j_R}\left(-\delta_{mj}\delta_{nj'}-\delta_{mj'}\delta_{nj}\right)\tilde{C}(x_j,x_{j'};k) \\
& +\sum_{k=k_1}^{k_C}\sum_{i=i_1}^{i_P}\left(\delta_{mk}\delta_{ni}+\delta_{mi}\delta_{nk}\right)\tilde{C}(x_k,x_i;k) \\
& +\sum_{k=k_1}^{k_C}\sum_{j=j_1}^{j_R}\left(-\delta_{mk}\delta_{nj}-\delta_{mj}\delta_{nk}\right)\tilde{C}(x_k,x_j;k)\Bigg]\left(\prod_{z\in\{j_1,\dots,k_C\}} q_z\right) \\
+ & \sum_{\beta_1\in\{j_1,\dots k_C\}}\left(\prod_{z\in\{j_1,\dots k_C\}\setminus\beta_1} q_z\right)\times \\
& \times\Bigg\{\sum_{i=i_1}^{i_P}\delta_{mi}\delta_{x_i x_{\beta_1}}\tilde{g}_{\beta_1 n}(k)\tilde{C}(x_i,0;0) \\
& +\sum_{i=i_1}^{i_P}\delta_{mi}(1-\delta_{x_i x_{\beta_1}})\tilde{C}(x_i,x_{\beta_1};k)\tilde{g}_{\beta_1 n}(k) \\
& +\sum_{i=i_1}^{i_P}\delta_{ni}\delta_{x_i x_{\beta_1}}\tilde{g}_{m\beta_1}(k)\tilde{C}(x_i,0;0) \\
& +\sum_{i=i_1}^{i_P}\delta_{ni}(1-\delta_{x_i x_{\beta_1}})\tilde{C}(x_i,x_{\beta_1};k)\tilde{g}_{m\beta_1}(k) \\
& -\sum_{j=j_1}^{j_R}\delta_{mj}\delta_{x_j x_{\beta_1}}\tilde{g}_{\beta_1 n}(k)\tilde{C}(x_j,0;0) \\
& -\sum_{j=j_1}^{j_R}\delta_{mj}(1-\delta_{x_j x_{\beta_1}})\tilde{C}(x_j,x_{\beta_1};k)\tilde{g}_{\beta_1 n}(k) \\
& -\sum_{j=j_1}^{j_R}\delta_{nj}\delta_{x_j x_{\beta_1}}\tilde{g}_{m\beta_1}(k)\tilde{C}(x_j,0;0) \\
& -\sum_{j=j_1}^{j_R}\delta_{nj}(1-\delta_{x_j x_{\beta_1}})\tilde{C}(x_j,x_{\beta_1};k)\tilde{g}_{m\beta_1}(k)\Bigg\}. \qquad (241)
\end{aligned}
$$

1.5.4 The structure of the interaction function $r(\mathbf{P},\mathbf{R},\mathbf{C})$

The equations for functions H_q, H_p and H_g in a general form (181), (184) and (188), as well as in the simplified form in real space (232)-(234), and in Fourier space (237), (238), (241) can be used to describe a process with the interaction function which satisfies the condition (175) recalled here:

$$r_\epsilon(\mathbf{P},\mathbf{R},\mathbf{C}) := \left(\epsilon^d\right)^{P+R+C-1} r(\mathbf{P}_\epsilon,\mathbf{R}_\epsilon,\mathbf{C}_\epsilon). \qquad (242)$$

The equations mentioned above allowed us to develop programming codes which can generate equations for H_q, H_p and H_g for a general process from a very wide class. However, the processes which can be studied by “The Model Constructor” and “The Model Simulator” toolboxes should satisfy an additional requirement, which still allowed us to apply these toolboxes to a very wide

class of processes including all selected basic processes studied in this Supplementary Note 1. Particularly, the toolboxes developed by us can be applied to point processes with N points in groups $\mathbf{P}$, $\mathbf{R}$ and $\mathbf{C}$, $N = P+R+C$, where the interaction function $r(\mathbf{P},\mathbf{R},\mathbf{C})$ is given by a product of $N-1$ kernels, and each kernel is a symmetric function of a single variable. Each of these kernels depends on a distance between two points and satisfies scaling condition (23), e.g. for a kernel a the condition is

$$a_\epsilon(x) := \epsilon^d a(\epsilon x). \tag{243}$$

One can see, that the interaction function $r(\mathbf{P},\mathbf{R},\mathbf{C})$ described here satisfies the condition (242).

It is convenient to use the graph theory to represent the structure of the interaction function $r(\mathbf{P},\mathbf{R},\mathbf{C})$ of processes which can be studied by the toolboxes presented in this work. Indeed, a simple (or undirected) graph can be constructed based on the structure of the function $r(\mathbf{P},\mathbf{R},\mathbf{C})$ if for every point in groups $\mathbf{P}$ and $\mathbf{R}$ and $\mathbf{C}$ one point is constructed in the graph, and for each kernel which is present in $r(\mathbf{P},\mathbf{R},\mathbf{C})$ and which depends on a distance between two points one line is constructed in the graph such that this line connects the corresponding two points in the graph. By definition, the number of lines in a graph equals to a number of kernels in $r(\mathbf{P},\mathbf{R},\mathbf{C})$. For all processes where single events involve more than one point, every point is connected to any other point by a single kernel or by kernels connected to intermediate points. Therefore, in the graph corresponding to such a process any point should be connected to any other point by a certain path(s), i.e. the graph is a connected graph. Moreover, taking into account that the graph is a connected graph with N points and $N-1$ lines, it follows that the graph does not have loops, i.e. there should be only a unique path connecting any two points in the graph. This can be proven by a construction of such a graph: one can see that it is impossible to construct neither a closed loop nor more than one single connected graph using N different points and $N-1$ lines connecting points.

Having the interaction function $r(\mathbf{P},\mathbf{R},\mathbf{C})$ of the type described above the expressions for functions $C(x_{\beta_1}, x_{\beta_2}; x)$ and $\tilde{C}(x_{\beta_1}, x_{\beta_2}; k)$ for a general process can be written in a simple form. We would like to recall, that the function $C(x_{\beta_1}, x_{\beta_2}; x)$ is obtained from the full interaction function by fixing locations of points x_{β_1} and x_{β_2} so that $|x_{\beta_1} - x_{\beta_2}| = x$, and integrating over the whole space the locations of the rest of points. Let kernels $a_{\text{in}}(x), b_{\text{in}}(x), ..., c_{\text{in}}(x)$ be the kernels which correspond to lines in the path connecting x_{β_1} and x_{β_2} in the graph, and let kernels $a_{\text{out}}(x), b_{\text{out}}(x), ..., c_{\text{out}}(x)$ be the kernels which corresponds to lines outside that path. Therefore, formally the interaction function contains the following product of kernels:

$$r(\mathbf{P},\mathbf{R},\mathbf{C}) \sim a_{\text{in}}(x')b_{\text{in}}(y')...c_{\text{in}}(z')a_{\text{out}}(x'')b_{\text{out}}(y'')...c_{\text{out}}(z''). \tag{244}$$

The integration over locations of points which do not belong to the path connecting x_{β_1} and x_{β_2} will effectively integrate each kernel with the subscript "out" over the whole space, as using the change of variables the integration over argument of each such kernel can be made independently from other integrations. Denoting the integral over the whole space by capital letters, e.g. $A_{\text{out}} = \int_{\mathbb{R}^d} a_{\text{out}}(x)dx$, the contribution from such an integration is given by $A_{\text{out}}B_{\text{out}}...C_{\text{out}}$.

The integration over locations of points which stay inside the path connecting x_{β_1} and x_{β_2} creates a convolution of kernels which corresponds to lines on that path. Denoting the convolution by the symbol $\star$, the contribution from such an integration is given by $(a_{\text{in}} \star b_{\text{in}} \star ... \star c_{\text{in}})(x) = \int_{(\mathbb{R}^d)^n} dx_1 dx_2 ... dx_n a_{\text{in}}(x_1) b_{\text{in}}(x_1 - x_2) ... c_{\text{in}}(x_n - x)$.

Thus, combining two contributions together, we obtain

$$C(x_{\beta_1}, x_{\beta_2}; x) = (a_{\text{in}} \star b_{\text{in}} \star ... \star c_{\text{in}})(x) A_{\text{out}} B_{\text{out}} ... C_{\text{out}}. \tag{245}$$

Using Fourier transforms of kernels, e.g. a Fourier transform $\tilde{a}_{\text{in}}(k)$ of the kernel $a_{\text{in}}(x)$, the expression for $\tilde{C}(x_{\beta_1}, x_{\beta_2}; k)$ becomes:

$$\tilde{C}(x_{\beta_1}, x_{\beta_2}; k) = \tilde{a}_{\text{in}}(k)\tilde{b}_{\text{in}}(k)...\tilde{c}_{\text{in}}(k)\tilde{a}_{\text{out}}(0)\tilde{b}_{\text{out}}(0)...\tilde{c}_{\text{out}}(0). \tag{246}$$

Thus, the algorithm mentioned here is used to write a programming code in "The Model Constructor" toolbox to calculate functions $C(\beta_1, \beta_2, x)$ and $\tilde{C}(\beta_1, \beta_2, k)$ for a general process with the interaction function satisfying the required conditions.

Supplementary Note 2

The tutorial to the toolbox “The model simulator” and to the toolbox “The model constructor”

We present a toolbox “The model simulator” and a toolbox “The model constructor” which allow the reader to obtain individual-based simulations and to perform analytical analysis of spatial stochastic ecological dynamics in the system that interests them.

Spatial stochastic models are known to be important in ecology and generally in population biology. The variety of biological systems is huge, but the ecological dynamics of an arbitrary biological system consists of a number of a typical distinct processes (e.g. birth, death, dispersal, etc.). Therefore, a particular ecological scenario can be constructed from the known constituent terms. To apply our method in a particular ecological scenario, the reader simply has to sum the constituent terms appropriate for their problem, and to use one of toolboxes presented in this work.

Therefore, in this tutorial we show: 1) how to define the model (section 2.1); 2) how to use “The model simulator” toolbox to obtain individual-based simulations of the defined model (section 2.2); 3) how to use “The model constructor” toolbox to obtain analytical expressions for spacial moments and cumulants for the defined model in the approximation of long-ranged interactions (section 2.3).

Contents

2.1 Define the model 99

- 2.1.1 Commonly applied model components. 100
- 2.1.2 Combine model components into the full model. 101
- 2.1.3 User-defined model component. 102

2.2 Tutorial for “The model simulator” toolbox 103

2.3 Tutorial for “The model constructor” toolbox 114

2.1 Define the model

The biological system is represented by individuals (or particles, or points) which can be distinguished by their belonging to certain species. The dynamics of individuals may be approximated as such that take place in one, two or three spatial dimensions. Often, the ecological dynamics is considered on certain surface, i.e. in two dimensions.

2.1.1 Commonly applied model components.

Typically, the dynamics of individuals in the system has n (where n is integer) different types of individual-based events (corresponding to births, deaths, movements, etc.) characterised by certain rates (e.g. birth rate, mortality rate, etc). Each type of events together with its rate forms a separate model component. The individual-based definition of the full model is complete when all model components are identified. In Supplementary Table 3 we present a graphical definition of a variety of commonly applied model components. The description of each component is also provided below.

Immigration[s_1, r] – individuals of type s_1 appear spontaneously at rate r. This model component is denoted by the operator $L^{\mathrm{IM}}_{s_1}(r)$.

Birth[s_1, a] – existing individuals of type s_1 produce new individuals of type s_1 with kernel a. This means, that if there is only a single individual of type s_1 at the location x_1 then the new individual of type $s1$ at location x_2 will appear with rate $a(x_1-x_2)$. Also, the kernel a incorporates both fecundity (as an integral of a over the whole space) and dispersal (as a shape of a). This model component is denoted by the operator $L^{\mathrm{B}}_{s_1}(a)$.

BirthToAnotherType[s_1, s_2, a] – existing individuals of type s_2 produce new individuals of type s_1 with kernel a. This model component is denoted by the operator $L^{\mathrm{BT}}_{s_1,s_2}(a)$.

BirthByFacilitation[s_1, s_3, a, b] – type s_1 individuals, influenced by type s_3 individuals ($s_1 \neq s_3$) with kernel a, produce offspring of type s_1 with kernel b. This model component is denoted by the operator $L^{\mathrm{BF}}_{s_1,s_3}(a,b)$.

BirthToAnotherTypeByFacilitation[s_1, s_2, s_3, a, b] – type s_2 individuals, influenced by type s_3 individuals ($s_2 \neq s_3$) with kernel a, produce offspring of type s_1 with kernel b. This model component is denoted by the operator $L^{\mathrm{BTF}}_{s_1,s_2,s_3}(a,b)$.

DensityIndependentDeath[s_1, r] – individuals of type s_1 die spontaneously with rate r. This model component is denoted by the operator $L^{\mathrm{D}}_{s_1}(r)$.

DeathByCompetition[s_1, a] – type s_1 individuals induce death in type s_1 individuals with kernel a. This model component is denoted by the operator $L^{\mathrm{C}}_{s_1}(a)$.

DeathByExternalFactor[s_1, s_2, a] – type s_2 individuals induce death in type s_1 individuals with kernel a. This model component is denoted by the operator $L^{\mathrm{DE}}_{s_1,s_2}(a)$.

ChangeInType[s_1, s_2, r] – individuals of type s_2 change spontaneously into type s_1 ($s_1 \neq s_2$) at rate r. This model component is denoted by the operator $L^{\mathrm{CT}}_{s_1,s_2}(r)$.

Jump[s_1, a] – type s_1 individuals change their positions by a jump with kernel a. This model component is denoted by the operator $L^{\mathrm{J}}_{s_1}(a)$.

JumpAndChangeInType[s_1, s_2, a] – type s_2 individuals change their positions by a jump with kernel a, and simultaneously type s_2 individuals change their type into s_1. This model component is denoted by the operator $L^{\mathrm{JCT}}_{s_1,s_2}(a)$.

Infection[s_1, s_2, a] – individuals of type s_1 change individuals of type s_2 ($s_2 \neq s_1$) into type s_1 individuals with kernel a. This model component is denoted by the operator $L^{\mathrm{I}}_{s_1,s_2}(a)$.

BirthByConsumption[s_1, s_2, a, b] – type s_1 individuals induce death of type s_2 individuals

Model component	Before			After			Rate
	x_1	x_2	x_3	x_1	x_2	x_3	
Immigration[s_1, r]				s_1			r
Birth[s_1, a]		s_1		s_1	s_1		$a(x_1 - x_2)$
BirthToAnotherType[s_1, s_2, a]		s_2		s_1	s_2		$a(x_1 - x_2)$
BirthByFacilitation[s_1, s_3, a, b]		s_1	s_3	s_1	s_1	s_3	$a(x_3 - x_2)b(x_2 - x_1)$
BirthToAnotherTypeByFacilitation[s_1, s_2, s_3, a, b]		s_2	s_3	s_1	s_2	s_3	$a(x_3 - x_2)b(x_2 - x_1)$
DensityIndependentDeath[s_1, r]	s_1						r
DeathByCompetition[s_1, a]	s_1	s_1			s_1		$a(x_1 - x_2)$
DeathByExternalFactor[s_1, s_2, a]	s_1	s_2			s_2		$a(x_1 - x_2)$
ChangeInType[s_1, s_2, r]		s_2			s_1		r
JumpAndChangeInType[s_1, s_2, a]		s_2		s_1			$a(x_1 - x_2)$
Jump[s_1, a]		s_1		s_1			$a(x_1 - x_2)$
Infection[s_1, s_2, a]		s_2	s_1		s_1	s_1	$a(x_2 - x_3)$
BirthByConsumption[s_1, s_2, a, b]		s_2	s_1	s_1		s_1	$a(x_2 - x_3)b(x_1 - x_3)$
BirthToAnotherTypeByConsumption[s_1, s_2, s_3, a, b]		s_2	s_3	s_1		s_3	$a(x_2 - x_3)b(x_1 - x_3)$
ChangeInTypeByConsumption[s_1, s_2, s_3, a]		s_2	s_3		s_1		$a(x_2 - x_3)$

Supplementary Table 3: Graphical definition of some commonly applied model components (processes). Each component is denoted by a name followed by arguments in square brackets. Symbols s_1, s_2 and s_3 denote types of species, the symbol r denotes a rate which does not depend on coordinates, a and b denote kernels which are symmetric functions of coordinates, i.e. $a(-x) = a(x)$, $b(-x) = b(x)$. Here, each component is defined by a single typical event during which an example configuration shown in the column "Before" changes into the configuration shown in the column "After" with the rate shown in the last column. Three different locations of species in continuous space are denoted symbolically by x_1, x_2 and x_3. Dynamics of species is considered either in one-, two- or three-dimensional space.

with kernel a; and simultaneously give birth to type s_1 individuals with kernel b. This model component is denoted by the operator $L^{\mathrm{BC}}_{s_1,s_2}(a,b)$.

BirthToAnotherTypeByConsumption[s_1, s_2, s_3, a, b] – type s_3 individuals induce death of type s_2 individuals with kernel a; and simultaneously give birth to type s_1 individuals with kernel b. This model component is denoted by the operator $L^{\mathrm{BTC}}_{s_1,s_2,s_3}(a,b)$.

ChangeInTypeByConsumption[s_1, s_2, s_3, a] – type s_2 individuals change to type s_1 ($s_1 \neq s_2$) individuals, and simultaneously induce death of type s_3 individuals with kernel a. This model component is denoted by the operator $L^{\mathrm{CTC}}_{s_1,s_2,s_3}(a)$.

2.1.2 Combine model components into the full model

The individual-based definition of the model is complete when all model components are identified. Such a complete definition is given by an operator L which is found as a sum of all corresponding operators for each model component:

$$L = L_1 + L_2 + \ldots + L_n, \tag{247}$$

where the L_i are operators corresponding to the model components in the system.

For example, consider the system where all individuals belong to the same species. The individuals produce new individuals with kernel a^+, which incorporates both fecundity (as an integral of a^+ over whole space) and dispersal (as a shape of a^+). The death rate includes a density independent component (background mortality rate μ) and a density dependent component (due to competition with kernel a^-). Denoting type of species in the system by index 1, such a model is defined by the following operator L,

$$L = L_1^{\mathrm{B}}(a^+) + L_1^{\mathrm{D}}(\mu) + L_1^{\mathrm{C}}(a^-). \tag{248}$$

Once all model components are identified, it is possible to use "The model simulator" toolbox (the tutorial is presented in the section 2.2), or "The model constructor" toolbox (the tutorial is presented in the section 2.3).

2.1.3 User-defined model component

The user may need to use a model component which is not presented in the Supplementary Table 3. In such a case a new model component can be constructed in the following way.

First, the process should be defined graphically in the same way as in the Supplementary Table 3. Second, using the graphical definition the groups "Products", "Reactants" and "Catalysts" should be identified and expressed in terms of species and their locations (see Supplementary Note 1 for the definition of these groups). Notice, that the following conditions should be fulfilled:

1) Any location cannot appear in any single group more than once, as no two species can occupy the same location. However, the same location can appear in different groups.

2) All locations in the group "Catalysts" should be unique and cannot appear in groups "Products" and "Reactants".

3) Groups "Products" and "Reactants" should not have identical pairs {species, location}.

"The model constructor" toolbox allows the user to check if the user-defined processes satisfy these three conditions, this is discussed in the tutorial for "The model constructor" toolbox in the section 2.3.

Second, the user should define rates and kernels according to requirements discussed in the Supplementary Note 1, section 1.5.4.

Third, the characteristics of the new model component should be assembled into the definition in the same txt format as shown in the examples in Supplementary Figure 1. In order to use the

```
Immigration[s1_, r_] := Module[{Products = {{s1, x1}}, Reactants = {}, Catalysts = {}, listAll,
function}, listAll = {Products, Reactants, Catalysts}; function[x1_] := r; {listAll, function}];

BirthByFacilitation[s1_, s3_, a_, b_] := Module[{Products = {{s1, x1}}, Reactants = {}, Catalysts
= {{s1, x2}, {s3, x3}}, listAll, function}, listAll = {Products, Reactants, Catalysts}; function[x1_,
x2_, x3_] := a[x3 - x2] b[x2 - x1]; {listAll, function}];
```

Supplementary Figure 1: Examples of definitions of model components for "The model simulator" toolbox.

new model component in "The model simulator" toolbox the definition of the model component should be added to the file "processes.txt" needed to run "The model simulator" toolbox. The use

of the new model component in “The model constructor” toolbox requires few additional changes to the definition, this is explained in details in the tutorial for “The model constructor” toolbox in the section 2.3.

2.2 Tutorial for “The model simulator” toolbox

Introduction

The main part of the “The model simulator” toolbox is ppsimulator, which is a C-program for simulating point processes in continuous time and space. Each point is associated with a coordinate and a discrete species attribute. Points are located either on 1D or 2D torus space. User defines the set of processes and the initial configuration after which the simulator runs Gillespie algorithm (*Gillespie D. J. Phys. Chem,* ***81****(25), 2340-236 (1977),* Ref. [33] in the main text) in such a way that the information of point locations are taken into account, i.e., the system is not assumed to be well-mixed. The state of the configuration can be outputted at user-defined constant time intervals. Auxiliary R-functions are provided for calculating summary statistics and creating figures and animations based on the simulation. Input for the simulator is given by means of text files and few command line arguments. Output of the simulator is written in text files.

Note: version 1.0 supports only simple processes where the connectivities between the points (if present) must be defined by direct links from a single central point of the process.

Contents

Introduction 103
Installing the software 104
Command line arguments 105
  Process definition –p 105
  Model definition –m 106
  Initial configuration –i 106
  Simulation space –U, -w 107
  Simulation time –T, -dT, -E, -s 107
  Random number seed –r 107
  Output –o, -osep, -outc, -outn, -oute 107
  Multiresolution grid –H 108
  Information about the simulation –info 108
Examples 108
  Generating initial configuration 109
  Running the simulator 109
  Visualizing the results 109
  Calculating spatial moments 111
  Crosscorrelation 111
  Creating animation 112
  Running simulator over the set of parameters 112

Installing the software

Software has been packed into a single file ppsimulator.zip. After uncompressing the file, user should have C source code and a data directory containing exemplary processes and R functions. R is not required for running the simulator but it is useful for visualizing the results. R can be downloaded from http://www.r-project.org for all three platforms Linux, Mac, and Windows.

The first task is to compile the C program. Linux users typically have a pre-installed C compiler in their operating system. Mac users can get it by means of Xcode and Windows users can get a GNU C compiler e.g. by installing Cygwin https://www.cygwin.com. Using the terminal (here character '$' denotes the prompt), the compiling is done by

```
$ gcc ppsimulator.c -lm -O2 -o ppsimulator
```

When the program is run without any arguments, it prints its usage

```
$ ./ppsimulator
PointProcessSimulator version 1.0
required arguments:
 -p filename process definitions
 -m filename model components
 -i filename initial configuration
 -o filename basename of output
 -U integer/float space length
 -T integer max time
optional arguments:
 -dT integer/float interval for saving the state of configuration,
     negative value means saving only final output (default -1)
 -osep filename basename for separate point coordinate files
       (default none, if defined, this will set -outc 0)
 -outc 1/0 output merged point coordinate file '.points', 1: yes,
       0: no (default 1)
 -outn 1/0 output point count file '.counts', 1: yes, 0: no
       (default 1)
 -oute 1/0 output event count file '.events', 1: yes, 0: no
       (default 1)
-trL float truncation limit for Gaussian kernel in units of sd
     (default 3)
 -w integer/float cell width (default min kernel radius), note: w
    will be rounded up so that U is its multiple
 -r integer seed value for random number generator (default 1)
 -E integer max number of events (default 2147483647)
 -s 1/0 stop if extinction of points, 1: yes, 0: no (default 1)
 -H integer number of hierarchical levels to speedup computation
    for large U/w (default 0)
 -info integer (default 0)
```

Command line arguments

Process definition –p

Process definition file contains the symbolic text descriptions of the processes. The syntax is from Mathematica. This is because the present software has originally been written to be used for comparing its results against analytical calculations and the software package for calculating the analytical results has been written using Mathematica. It is easiest for the user that the same syntax can be used in both programs. A collection of exemplary process definitions using the syntax of the Mathematica cookbook (see Supplementary Note 2, section 2.3) is placed in a file processes.txt in data directory. In brief, each process may have input points which are either reactants or catalysts, and output points which are called products. Difference between reactant and catalyst is that reactants disappear during the process whereas catalysts remain unchanged. In addition, each process has a rate which gives the probability for the process to occur. There are three options for the locations of the products: 1) anywhere in space (rate per area process), 2) specific coordinate based on reactant point (change-of-type process), and 3) random position according to dispersal kernel.

In general, the processes can be divided into three types based on how the process rates are calculated: 1) processes without input (rate per area), 2) processes with input points without connectivities (rate per individual), and 3) processes with input points where rate is based on connectivities. With the Mathematica syntax it is possible to define fairly general processes with complex dependencies between the points. However, the current version 1.0 of ppsimulator supports only simple processes where all connectivities (if present) must be defined based on a single central point of the process and the number of points per species is limited, see Supplementary Figure 2. The reason for this is mainly computational. Although this may sound very restrictive, it nonetheless allows to define many interesting processes. For example, all processes listed in the file processes.txt are such. Although each individual process is simple, their combinations allow to generate non-trivial models.

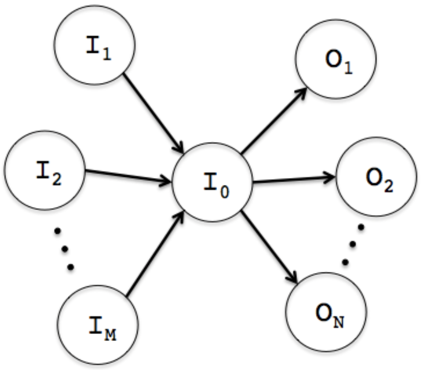

Supplementary Figure 2. Processes involving connectivities must be defined based on a central input point in version 1.0. Furthermore, all points I_1...I_M, must be from different species (one of them is allowed to be the same as the species of I_0). Inputs can be reactants or catalysts and outputs are products.

For the simulation, only those processes are generated which are defined in a model file using command line option -m. There is no overhead in the simulation if process definition file contains additional processes, therefore the process definition file can be considered as a

library of all user defined processes and for each individual simulation, only the model file needs to be changed.

Model definition –m

Model input file provides numerical values for process parameters. Parameters include species identifiers and rate parameters. Species identifiers are integers starting from 1. In principle, any integer values can be used, but in practice it is computationally most efficient if for N species, the values are consecutive integers 1…N. If process involves connectivities, two basic types of connectivity kernels are supported: tophat kernel and truncated Gaussian, see Supplementary Figure 3. Both kernel types are spherically symmetric with finite support. Tophat kernel is parameterized with two values: integral and radius, and truncated Gaussian is parameterized with two values: integral and standard deviation. The truncation radius can be given by the command line argument –trL, it is given as the units of standard deviation and the default value is three times the standard deviation. If the kernel should cover the entire space, a character string `global` can be given for the value of radius. In this case the density is a constant for both tophat and truncatedGaussian kernel, the value being integral/domain area.

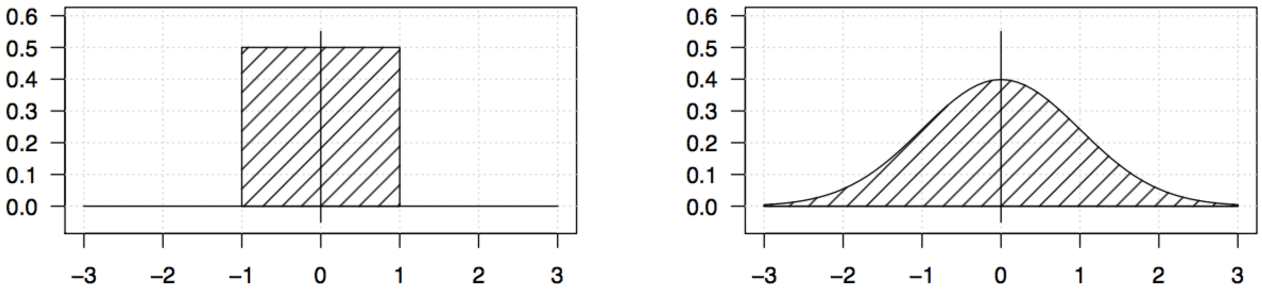

Supplementary Figure 3. Two kernels `tophat[1,1]` (left) and `truncatedGaussian[1,1]` (right) in 1D.

For modeling attractors or repellants, two types of connectivity kernels shown in Supplementary Figure 4 can be used. They require three parameters: integral, distance from the central attractor/repellant point to the center of the edge area, and the radius/standard deviation of the edge area. The value of the second parameter must be at least the value of the truncation radius.

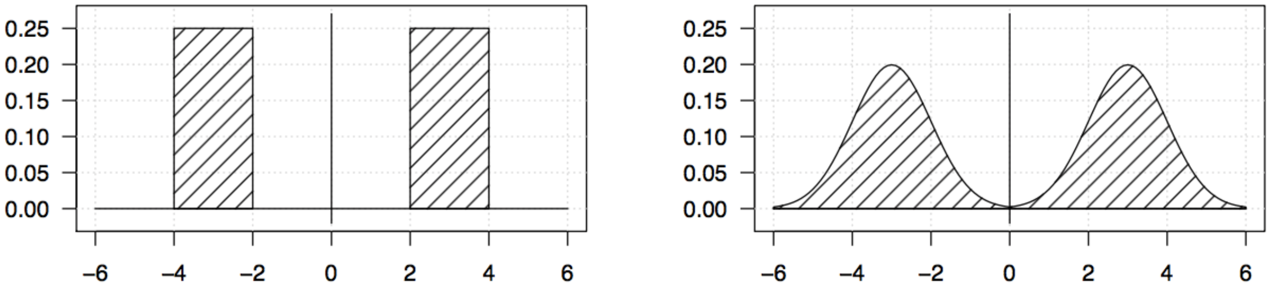

Supplementary Figure 4. Kernels `edgeTophat[1,3,1]` (left) and `edgeTruncatedGaussian[1,3,1]` (right) in 1D. The origin is the location of the attractor/repellant point.

Initial configuration –i

Initial point configuration is given by a text file where each point is in a separate line. First column is a species identifier and the remaining columns are for point coordinates. For 1D data, the file should contain only two columns (species id and x-coordinate) and for 2D data, the file should have exactly three columns (species id, x-coordinate, and y-coordinate). The dimensionality is obtained based on the content of the input file, columns are separated by white space.

Simulation space –U, -w

User defines the length of the space by –U. Both x and y coordinates of the points are between 0…U and 2D space is defined to be U-by-U area. The space has torus topology so all coordinate values are treated with modulo U. For the computational efficiency, the space is divided into a discrete grid. However, all point coordinates are continuous valued (with the resolution of double precision floating point number) and the system is not assumed to be well-mixed within a grid. Since all connectivity kernels have finite support, the grid allows to reduce the distance calculations between points by screening out all points located at distant grid tiles. By default, the length of the grid tile equals the radius of the smallest connectivity kernel but it can be set manually with command line option –w. The final value of w is rounded up so that U is its multiple.

Simulation time –T, -dT, -E, -s

Maximum simulation time is set by command line option –T. The time refers to the Gillespie algorithm and it has nothing to do with real or cpu time. By default, the program outputs only final configuration, but if user sets a constant time interval with argument –dT, the program outputs the state of the configuration at that time interval. This can be used for obtaining information about the dynamics of the process and/or for creating animations. By default, simulator stops if 1) maximum allowed time has been passed, 2) maximum number of events have occurred, 3) there are no points left in the configuration, or 4) process activity (rate parameter of exponential distribution) drops below threshold 1e-10. If the user wants that the simulation continues even when there are no points in the configuration, the command line argument –s 0 can be set. This makes sense e.g. if the model includes a migration process in which case an empty space can be just a temporary state during simulation.

Random number seed –r

The simulator utilizes random number generator drand48 from C-library stdlib. Seed value can be set by command line argument –r, the default value is 1.

Output –o, -osep, -outc, -outn, -oute

By default, program outputs three files whose base name is provided by –o. The file with suffix .points contains the species ids and coordinates of the points, the file with suffix .counts contains the number of the points within each species, and the file with suffix .events contains the number of process events. There is one column for each model component. The fourth file with the suffix .eventnames.txt contains the full names of the process corresponding to the mnemonics e1, e2, etc. used in the .events file. If the user is not intererested in point coordinates (which may result in large file size), the corresponding file is not generated if setting the command line argument –outc 0. Similarily, the file containing the number of

events is not generated if setting –oute 0, and the number of points per species group is not generated if setting –outn 0.

The default output format for point coordinates is a file where for each snapshot of the process, the points are listed in one line. With large point configurations, this is not practical, and user can produce separate file for each snapshot. This is done by option -osep filename, where filename is the base name for snapshot files. The format of each snapshot file is the same as the format of the initial configuration file where each point is in a separate line. When generating several snapshots of the simulation e.g. for an animation, it may be a good practice to create a separate directory for each simulation run where snapshot files are written. As an example:

```
$ mkdir snapshots
$ ../ppsimulator -p processes.txt -m model.txt -i pointsinfile -o
out -U 50 -osep snapshots/tmp -T 1000 -dT 1
```

The benefit of using the argument –osep is that the output file can be used directly as an input to continue the simulation. This can be utilized e.g. if there are issues with numerical accuracy when calculating the process rates. Every time the simulator is started from command line, the process rates of all points are re-calculated.

Multiresolution grid –H

In some cases when the width of connectivity kernel is small compared to U and U/w is large, the most time consuming function in the simulator is the search for the grid cell where the next process will take place. This can be done more efficiently when using multiresolution hierarchy for the grid cells. The number of hierarchy levels is controlled by –H. The speedup depends on the model and the parameters. Profiling the code with the specific model reveals which part of the simulator is the bottleneck. In Linux, profiling can be done e.g. with gprof.

Information about the simulation –info

By default, the simulator runs silently outputting on screen only the number of events in the end of the simulation. With –info 1, it outputs the name of the event each time it occurs, and with –info 2, also the current configuration is outputted. In both cases, the simulation run slows down considerably. Besides for debugging purposes, the main reason for using –info 2 is to check that the input files have been prepared correctly. This can be done by running the simulator with –T 0 which doesn't generate any events but prints the process descriptions and model definitions on screen. For checking the behavior of each process, one possibility is to define –info 2 –E 1 which processes only a single process event. For this case the model file should be prepared so that it contains only a single process under investigation and the initial configuration may contain only few points so that it is easy to check that the process has performed as intended.

Examples

Note: When copy-pasting commands from pdf to terminal, some characters may change, especially dash (-) and double quote ("), so these characters may need to be re-typed.

Generating initial configuration

Initial point configuration file can be generated by any text editor. For random initializations, the data directory contains a file of R functions to automatize the task. After starting R, auxiliary R-functions can be taken into use by giving source command (here '>' denotes the R-prompt). It is assumed that the user is in the data directory where the file rfunctions.R is.

```
> source("rfunctions.R")
```

Let us define two species which are evenly distributed with different densities in a 2D space. Domain of space is 30 spatial units in both dimensions. R-command rbind merges the two matrices (the same command can be used for merging more than two matrices) and the initial configuration is written in a file "xin". Function initialize has an optional argument poisson=TRUE/FALSE to control whether the number of initial points are drawn from Poisson distribution or not. By default, the value is FALSE, which means that the number of points is exactly floor(density.per.area * U^dim).

```
> x1=initialize(species=1, density.per.area=2, U=30, dim=2)
> x2=initialize(species=2, density.per.area=0.02, U=30, dim=2)
> xall=rbind(x1,x2)
> write.file(xall, "xin")
```

Running the simulator

Simulation is launched from command line. The following assumes that the user is in the data directory and the executable program is in its parent directory (../). Example model file contains processes for two species 1 and 2. The model consists of two components: 1) species 2 eats species 1, and 2) species 2 can move. These are defined in the file model.txt:

```
DeathByExternalFactor[1, 2, truncatedGaussian[1,1]]
Jump[2, truncatedGaussian[0.1, 0.5]]
```

In the example below, output is generated at every dT=1 time intervals up to T=100.

```
$ ../ppsimulator -p processes.txt -m model.txt -i xin -o out -U 30
-T 100 -dT 1
```

Alternatively, simulation can be launched from R:

```
> cmd="../ppsimulator -p processes.txt -m model.txt -i xin -o out
-U 30 -T 100 -dT 1"
> system(cmd)
```

Visualizing the results

Results can be visualized in R. If starting a new R session, functions in the file rfunctions.R must again be taken into use by command source("rfunctions.R"). Event history:

```
> a=read.table("out.events",header=T)
> plot.event.count(a)
```

Species density in time:

```
> n=read.table("out.counts",header=T)
> plot.species.density(n,U=30,dim=2)
```

In order to visualize the point coordinates, the time must be defined by the row index of the output file .points. Here the point configuration is shown in the beginning and end of the simulation. The following assumes that the entire time series is saved in a single file.

```
> y=read.timeseries.file("out.points")
> y.first=get.point.coordinates(y,ind=1,dim=2)
> plot.points(y.first$coord,y.first$species,U=30,main="Initial")

> y.last=get.point.coordinates(y,ind=length(y),dim=2)
> plot.points(y.last$coord, y.last$species, U=30, main="Final")
```

If the snapshots of the time series have been saved in separate files (using option –osep), individual files can be read one by one. Plotting function is the same.

```
> y1 = read.singletime.file("snapshots/tmp000007")
> plot.points(y1$coord, y1$species, U=30, main="Snapshot 7")
```

In R it is possible to modify the appearance of plots in many ways by changing the type of the character to be drawn and its size and color. By default, each species is drawn with different color but using the equally sized symbol (filled dot). In R, plot symbol, color, and size are controlled by arguments pch, col, and cex, respectively. In functions plot.points, movie, and movie2file, these are replaced by spch, scol, and scex, respectively (s coming from species). In case of 4 species, we can specify a 4-dimensional vector, e.g. spch=c("B","b","A","a") to plot species 1 points by letter B, species 2 points by letter b and so on. Instead of letters, we can use R plot symbols which can be selected using numbers, e.g. spch=c(19,17,1,2) would use filled circle and triangle for species 1 and 2, and open circle and triangle for species 3 and 4, respectively. More information about plot symbols can be found in R help. Plot colors can be specified with numbers or textual names, e.g. scol=c("black","blue","red","green"). The default size of the symbol is 1 which can be made larger or smaller e.g. by scex=c(2, 1, 0.5, 1.3). In the example data, we can plot species 2 with larger blue dot and species 1 with smaller green dot

```
> plot.points(y.last$coord, y.last$species, U=30, scex=c(1,2),
scol=c("green","blue") )
```

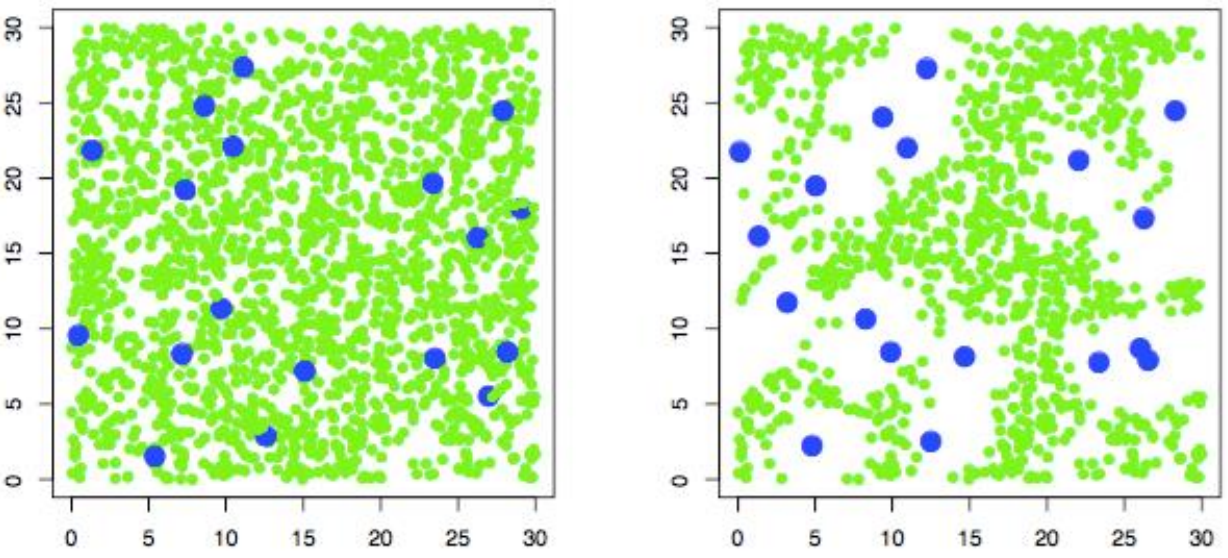

Supplementary Figure 5. Example simulation in initial state (left) and final state (right). Individuals from species 2 (blue dots) have consumed individuals from species 1 (green dots) within the radius of 3 spatial units according to truncated Gaussian kernel. Species 2 individuals have also moved slightly.

Calculating spatial moments

Second spatial moment can be investigated using R function spm2 in rfunctions.R. Spatial resolution can be controlled by argument grid.resolution whose default value is 1. Input domain U (U-by-U in case of 2D) is first discretized after which circular autocorrelation is calculated using FFT. In case of many species, it is easiest to use a wrapper function plot.spm2 which calls the function spm2 for every species. It works also in case of only one species.

```
> plot.spm2(y.first$coord,y.first$species,U=30, main="Initial")
> plot.spm2(y.last$coord, y.last$species, U=30, main="Final")
```

In case the interest is in values corresponding to small distances, the range of x axis can be limited by xlim.

```
> plot.spm2(y.last$coord, y.last$species, U=30, xlim=c(0,5),
grid.resolution=0.2)
```

In order to plot 2nd spatial cumulant, plot.spm2 can be provided with argument cumulant=T.

Crosscorrelation

Two point configurations can be compared by function spx which calculates spatial crosscorrelation. Function plot.spx is based on spx. Argument cumulant=T can be used also in this function. The following shows the spatial crosscorrelation between the beginning and end locations of species 1.

```
> plot.spx(y.first$coord, y.first$species, 1, y.last$coord,
```

```
y.last$species, 1, U=30, main="Species 1, start vs end")
```

This shows the spatial crosscorrelation between species 1 and 2 in the end of the simulation.

```
> plot.spx(y.last$coord, y.last$species, 1, y.last$coord,
y.last$species, 2, U=30, main="Species 1 vs 2")
```

Creating animation

With command line argument –dT, user can set the value of the time interval which is used for saving the state of the configuration. By default, all snapshots are stored in a single file with suffix .points, but with –osep, separate output file is generated for each time point.

In case all states of the time series are stored in a single file, the following R commands can be used (remembering to use source("rfunctions.R") in the beginning of new R session). Function movie shows the movie on screen and movie2file creates separate png images to be combined by external software. Parameters spch, scol, and scex can be defined in both functions to control the symbol type, color, and size of the point of each species.

```
> a=read.timeseries.file("out.points")
> movie(a,U=30,pause.interval=.1, scex=c(1,2), scol=c("green",
"blue"))
```

The following command assumes that the user has first created a directory named pics under the current working directory (Linux command mkdir pics).

```
> movie2file(a,U=30,"pics/tmp",scex=c(1,2),scol=c("green","blue"))
```

Names of the snapshot files are numbered consecutively so the numerical (also alphabetical) sort results in the correct order of images in the animation. Separate png images can be combined with Java program ImageJ http://imagej.nih.gov/ij/ which runs in all three platforms Linux, Mac, and Windows.

After starting ImageJ: 1) browse the directory containing the snapshot png images using File->Import->ImageSequence, click one of the files, Open and OK, and 2) save the movie by selecting File->SaveAs->AVI, choose the framerate (how many figures are shown per second), click OK, and give the name of the output filename. In Windows it may be necessary to tick the box "Convert to RGB" in step 1 in order to prevent flickering background.

Running simulator over the set of parameters

Here is an example how to run simulations over the set of parameter values. The same example model is used as before with the same initial configuration stored in file 'xin'. We use R to define a vector for the values of the standard deviation of truncatedGaussian kernel used in model component DeathByExternalFactor.

```
> stdevs=c(0.5,1,2)
```

The following for-loop in R runs over the length of stdevs-vector and creates a separate directory for each model. Simulations are also launched from R using system command and outputs of the simulations are stored in the same directory with the model.

```
for (i in 1:length(stdevs)) {
 # create output directory

 odir=sprintf("out%d",i)
 system(sprintf("mkdir %s",odir))

 # create model

 m1=sprintf("DeathByExternalFactor[1, 2, truncatedGaussian[1,%f]]",stdevs[i])
 m2="Jump[2, truncatedGaussian[0.1, 0.5]]"
 m=rbind(m1,m2)

 # write model file

 modelfile=sprintf("%s/model.txt",odir)
 write.table(m,modelfile,row.names=F,col.names=F,quote=F)

 # run simulator

 outname = sprintf("%s/out",odir)
 cmd = sprintf("../ppsimulator -p processes.txt -m %s -i xin -o %s -U 30 -T 100
-dT 1",modelfile,outname)
 print(cmd)
 system(cmd)
}
```

Outputs from each simulation are stored in separate directories out1, out2, and out3.

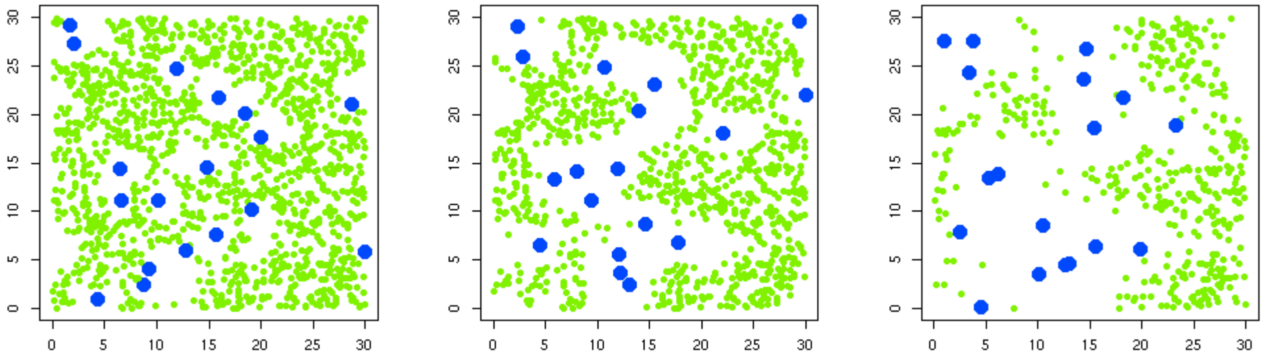

Supplementary Figure 6. Final configurations after T=100, for three models with different values for standard deviation of truncatedGaussian in model component DeathByExternalFactor: 0.5, 1.0, and 2.0 from left to right, respectively.

2.3 Tutorial for "The model constructor" toolbox

Contents

Introduction....114
1. Define the model....115
2. Obtain $\TeX$ files with expressions....116
3. Obtain analytical expressions and/or numerical solutions....118
  3.1. Compile the vector "processes" for analytical analysis....118
  3.2. Obtain analytical expressions....119
  3.3. Obtain numerical solutions in the chosen equilibrium....122
4. Define a new model component....127

Introduction

``The model constructor'' toolbox for Spatial Stochastic Point Processes (SSPP) is written in Wolfram Mathematica.
The toolbox consists of the following Mathematica packages:
1. SSPPlibraryOfProcessesForTeX.m
2. SSPPwriteInTeX.m
3. SSPPlibraryOfProcesses.m
4. SSPPanalyticalExpressions.m
5. SSPPnumericalResultsForEquilibrium.m

This toolbox is designed for analytical and numerical analysis of equations for evolution of the correlation functions and cumulants in the approximation of long-ranged interactions for a broad class of spatial stochastic point processes in 1-, 2-, and 3- dimensional space.

To use this toolbox packages should be installed and loaded.
To install the package, open the corresponding *.m file in Mathematica, using menu commands **File > Install** choose **"Type of Item to Install:" > Package, "Source:" > the name of the file, "Install Name:" > the name of the file.**
To load the package "packageName", use the command : **Get["packageName`"]**. Notice, that "packageName`" is a string consisting of the name and a back - tick mark.

This tutorial is constructed as follows. Section 1 explains how to define a system from a broad class of spatial stochastic point processes using pre-defined model components. In Section 2 it is shown how to construct and save in TEX file the full analytical expressions for right hand side of equations in Figure 1E in the main text. In Section 3, and particularly in 3.1 and 3.2, it is explained how to get analytical expressions (within Mathematica for further analysis) for these equations and for their analytical solutions at equilibrium. In Section 3.3 it is described how to obtain numerical solutions of equations in Figure 1E in the main text in a chosen equilibrium of the system.

1. Define the model

In the main text we describe the system by the list **processes,** which is constructed as model components with corresponding arguments written inside curly brackets and separated by a come, as shown below:

processes = {component1[arguments1], component2[arguments2], ...};

To find all pre-defined model components load the package "SSPPlibraryOfProcessesForTeX`".

```
Get["SSPPlibraryOfProcessesForTeX`"]
```

See all basic processes (model components) defined in the package by compiling "?SSPPlibraryOfProcessesForTeX`*". Clicking on any process in the list will provide its description:

```
? SSPPlibraryOfProcessesForTeX`*
```

▼ SSPPlibraryOfProcessesForTeX`

Birth	DeathByExternalFactor
BirthByConsumption	DensityIndependentDeath
BirthByFacilitation	Immigration
BirthToAnotherType	Infection
BirthToAnotherTypeByConsumption	Jump
BirthToAnotherTypeByFacilitation	JumpAndChangeInType
ChangeInType	listOfElementaryProcesses
ChangeInTypeByConsumption	TestTheCorrectnessOfProcesses
DeathByCompetition	

Construct the list **processes** from basic processes shown above.

Immigration[s1, r, Coefficient]
DensityIndependentBirth[s1, a, Af, Coefficient]
BirthToAnotherType[s1, s2, a, Af, Coefficient]
BirthByFacilitation[s1, s3, a, Af, b, Bf, Coefficient]
BirthToAnotherTypeByFacilitation[s1, s2, s3, a, Af, b, Bf, Coefficient]
DensityIndependentDeath[s1, r, Coefficient]
DeathByCompetition[s1, a, Af, Coefficient]
DeathByExternalFactor[s1, s2, a, Af, Coefficient]
ChangeInType[s1, s2, r, Coefficient]

Jump[s1, a, Af, Coefficient]
Infection[s1, s2, a, Af, Coefficient]
ChangeInTypeByConsumption[s1, s2, s3, a, Af, Coefficient]
BirthByConsumption[s1, s2, a, Af, b, Bf, Coefficient]

Often the prefactor “Coefficient” can be included in the definition of kernels and therefore “Coefficient”=1.

Denote species by numbers, e.g. a system with three different species could be described by species 1, 2 and 3, or by species 5, 8, 10; species should be denoted by numbers and not by string objects.

In this tutorial, an example system is described by the following list processes :

```
processes = {DensityIndependentBirth[1, ap, Apf, 1],
    DensityIndependentDeath[1, mu, 1], DeathByCompetition[1, am, Amf, 1]};
```

There are three basic processes occurring in the system: DensityIndependentBirth, DensityIndependentDeath, and DeathByCompetition. There is only one type of species, denoted by 1 (the first argument of each of these three processes). Interaction kernels are given by some functions **ap**, and **am** in real space, and their Fourier transforms are **Apf** and **Amf**, mortality rate is mu.

2. Obtain TEX files with expressions

2.1. Compile the vector "processes" for TEX

First, load the package “SSPPlibraryOfProcessesForTeX`”. The package “SSPPlibraryOfProcessesForTeX`” defines basic spatial stochastic point processes. To produce TEX files, this package is needed to compile the vector “processes” which describes the system.

```
Get["SSPPlibraryOfProcessesForTeX`"]
```

See all processes defined in the package by compiling “?SSPPlibraryOfProcessesForTeX`*”. Clicking on any process in the list will provide its description:

```
? SSPPlibraryOfProcessesForTeX`*
```

▼ SSPPlibraryOfProcessesForTeX`

Birth	DeathByExternalFactor
BirthByConsumption	DensityIndependentDeath
BirthByFacilitation	Immigration
BirthToAnotherType	Infection
BirthToAnotherTypeByConsumption	Jump
BirthToAnotherTypeByFacilitation	JumpAndChangeInType
ChangeInType	listOfElementaryProcesses
ChangeInTypeByConsumption	TestTheCorrectnessOfProcesses
DeathByCompetition	

Construct the vector “processes” from processes shown above. To produce TEX files all kernels and rates must be defined only symbolically, not numerically. For example:

```
processes = {DensityIndependentBirth[1, ap, Apf, 1],
   DensityIndependentDeath[1, mu, 1], DeathByCompetition[1, am, Amf, 1]};
```

2.2. Obtain $\TeX$ files

Now, load the package “SSPPwriteInTeX`”. The package “SSPPwriteInTeX`” allows to obtain $\TeX$ files with the expressions for time derivatives of the mean field, correction to the mean field, and leading contribution to cumulants.

```
Get["SSPPwriteInTeX`"]
```

See all processes defined in the package by compiling “?SSPPwriteInTeX`*”. Clicking on any process in the list will provide its description:

```
? SSPPwriteInTeX`*
```

▼ SSPPwriteInTeX`

inFourierSeparately	inFourierTogether	inRealSeparately	inRealTogether

inFourierSeparately[processes_,MyFileName_,qpgVariables_,kVariableInG_,variableOfIntegrationFT_] is a function that creates a TeX file with expressions for Hq, Hp and Hg functions in Fourier space for each elementary process separately, e.g. Hq for species 1 for a first elementary process, then for another species or another process etc.

Arguments:
processes -- is a list of elementary processes which describe the system.
MyFileName -- should be a string, as it provides
a name for a resulting tex file: MyFileName_InRealSpaceSeparately.tex.
qpgVariables -- should be given as a list of symbols used to denote q, p and g quantities from the main text. As a default, use qpgVariables={q,p,g}. Notice, these symbols should be clear and not used by other codes in Mathematica.
kVariableInG -- is a symbol to denote a variable in Fourier space used in g function. For example, kVariableInG=k.
variableOfIntegrationFT -- is a symbol used to denote a variable of
integration in Fourier space which appears in the integrals within expressions for Hp and Hg.

Choose the name of outcome $\TeX$:

```
MyFileName = "TestSystem";
```

Choose notations for the mean field, the correction to the mean field, and to the leading contribution to cumulants. These can be chosen, for example, as {q,p,g}:

```
qpgVariables = {q, p, g};
```

Also, choose notations for radial components in real and Fourier spaces:

```
kVariableInG = k;
variableOfIntegrationFT = k;
xVariableInG = x;
variableOfIntegration = x;
```

Compile the function you want to use, and find $\TeX$ files in the same folder where the file with these cells is located.

```
inRealSeparately[processes, MyFileName,
 qpgVariables, xVariableInG, variableOfIntegration]
```

```
inRealTogether[processes, MyFileName,
 qpgVariables, xVariableInG, variableOfIntegration]

inFourierSeparately[processes, MyFileName,
 qpgVariables, kVariableInG, variableOfIntegrationFT]

inFourierTogether[processes, MyFileName,
 qpgVariables, kVariableInG, variableOfIntegrationFT]
```

3. Obtain analytical expressions and/or numerical solutions

3.1. Compile the vector "processes" for analytical analysis

Here, load the package “SSPPlibraryOfProcesses`” twice, so that functions from the package “SSPPlibraryOfProcessesForTeX`” are re-defined. The package “SSPPlibraryOfProcesses`” defines basic spatial and stochastic point processes. This package is needed to generate the vector “processes” which describes the system, and which can be used to obtain analytical and numerical results.

```
Get["SSPPlibraryOfProcesses`"]
```

See all processes defined in the package by compiling “?SSPPlibraryOfProcesses`*”. Clicking on any process in the list will provide its description. This package contains the same processes as the package “SSPPlibraryOfProcessesForTeX`”, modified to be used for analytical and numerical calculations.

```
? SSPPlibraryOfProcesses`*
```

▼ SSPPlibraryOfProcesses`

Birth	DeathByExternalFactor
BirthByConsumption	DensityIndependentDeath
BirthByFacilitation	Immigration
BirthToAnotherType	Infection
BirthToAnotherTypeByConsumption	Jump
BirthToAnotherTypeByFacilitation	JumpAndChangeInType
ChangeInType	listOfElementaryProcesses
ChangeInTypeByConsumption	TestTheCorrectnessOfProcesses
DeathByCompetition	

Compile the vector “processes” which describes the system:

```
processes = {DensityIndependentBirth[1, ap, Apf, 1],
   DensityIndependentDeath[1, mu, 1], DeathByCompetition[1, am, Amf, 1]};
```

3.2. Obtain analytical expressions

Load the package "SSPPanalyticalExpressions`".

```
Get["SSPPanalyticalExpressions`"]
```

See all processes defined in the package by compiling "?SSPPanalyticalExpressions`*". Clicking on any process in the list will provide its description.

```
? SSPPanalyticalExpressions`*
```

▼ SSPPanalyticalExpressions`

gAllSolutionsAtGeneralQ	HGfALL	pAllSolutionsAtGeneralQ
gAllSolutionsAtSolvedQ	HPf	pAllSolutionsAtSolvedQG
gSolutionAtGeneralQ	HPfALL	qSolution
gSolutionAtSolvedQ	HQf	qSolutionsALL
HGf	HQfALL	speciesSSPP

gAllSolutionsAtGeneralQ[qpgVariables,processes,kVariable] shows a list of analytical expressions for all correlation functions g calculated at some equilibrium for a given system.

Arguments:
qpgVariables -- should be given as a list of symbols used to denote q, p and g quantities from the main text. As a default, use qpgVariables={q,p,g}. Notice, these symbols should be clear and not used by other codes in Mathematica.
processes -- is a list of elementary processes which describe the system.
kVariable -- the symbol denoting the modular component of wave vector in Fourier space.

Choose notations for the mean field, the correction to the mean field, and to the leading contribution to cumulants. These can be chosen, for example, as {q,p,g}:

```
qpgVariables = {q, p, g};
```

Choose notations for radial components in Fourier spaces:

```
variableOfIntegrationFT = k;
kVariable = k;
```

Choose a dimensionality of the space where dynamics is considered

```
dim = 2;
```

3.2.1. Analytical expressions for expressions in equilibrium

Species in the system are given by the function ``speciesSSPP''. The sequence of expressions in resulting expressions for the mean field and its corrections corresponds to the sequence of species in this function.

```
speciesSSPP[processes]
{1}
```

Solutions for the mean field in all types of equilibrium, stable and unstable, are given by the function "qSolutionsALL"

```
qSolutionsALL[qpgVariables, processes]
```

$$\left\{\{0\}, \left\{\frac{-mu + Apf[0]}{Amf[0]}\right\}\right\}$$

Solution for the leading contribution to the cumulant at some equilibrium without specifying the equilibrium is given by the function “gAllSolutionsAtGeneralQ”

```
gAllSolutionsAtGeneralQ[qpgVariables, processes, kVariable]
```

$$\left\{\frac{q[1]\,(Apf[k] - Amf[k]\,q[1])}{mu - Apf[k] + Amf[0]\,q[1] + Amf[k]\,q[1]}\right\}$$

In case of multiple species, cumulants calculated on two particular species m and n can be found separately using the function “gSolutionAtGeneralQ”, for example as g[m,n]. In this example there is only one species denoted by 1, so g[1,1] can be found by this function:

```
gSolutionAtGeneralQ[1, 1, qpgVariables, processes, kVariable]
```

$$\frac{q[1]\,(Apf[k] - Amf[k]\,q[1])}{mu - Apf[k] + Amf[0]\,q[1] + Amf[k]\,q[1]}$$

Solution for corrections to the mean field in some equilibrium without specifying the equilibrium is given by the function “pAllSolutionsAtGeneralQ”

```
pAllSolutionsAtGeneralQ[qpgVariables, processes, variableOfIntegrationFT, dim]
```

$$\left\{-\frac{2\pi\int_0^{\infty} k\,Amf[k]\,g[1, 1, k]\,dk}{mu - Apf[0] + 2\,Amf[0]\,q[1]}\right\}$$

In case when only the certain equilibrium should be considered, one should choose the equilibrium by assigning its order number in the list given by “qSolutionsALL” to the following parameter, for example:

```
solutionNumber = 2;
```

Check, that the solution chosen is correct

```
qSolution[qpgVariables, processes, solutionNumber]
```

$$\left\{\frac{-mu + Apf[0]}{Amf[0]}\right\}$$

Find expressions to cumulants and to corrections to the mean field using functions “gAllSolutionsAtSolvedQ” and “pAllSolutionsAtSolvedQG”:

```
gAllSolutionsAtSolvedQ[qpgVariables, processes, kVariable, solutionNumber]
```

```
{-(((mu - Apf[0]) (Amf[k] (mu - Apf[0]) + Amf[0] Apf[k])) /
     (Amf[0] (Amf[k] (-mu + Apf[0]) + Amf[0] (Apf[0] - Apf[k]))))}
```

```
gSolutionAtSolvedQ[1, 1, qpgVariables, processes, kVariable, solutionNumber]
```

```
-(((mu - Apf[0]) (Amf[k] (mu - Apf[0]) + Amf[0] Apf[k])) /
    (Amf[0] (Amf[k] (-mu + Apf[0]) + Amf[0] (Apf[0] - Apf[k]))))
```

```
pAllSolutionsAtSolvedQG[qpgVariables,
 processes, variableOfIntegrationFT, dim, solutionNumber]
```

$$\left\{\left(2\,\pi\int_0^{\infty}-\left(\left(k\,\text{Amf}[k]\,(\text{mu}-\text{Apf}[0])\,(\text{Amf}[k]\,(\text{mu}-\text{Apf}[0])+\text{Amf}[0]\,\text{Apf}[k])\right)\Big/\left(\text{Amf}[0]\,(\text{Amf}[k]\,(-\text{mu}+\text{Apf}[0])+\text{Amf}[0]\,(\text{Apf}[0]-\text{Apf}[k]))\right)\right)dk\right)\Big/\,(\text{mu}-\text{Apf}[0])\right\}$$

3.2.2. Analytical expressions for Hq,Hp,Hg functions

To obtain analytical expressions for functions Hq and Hp for specific species n, or for function Hg for species m and n, determine the species of interest:

```
speciesN = 1;
speciesM = 1;
```

and use the functions HQfALL, HPfALL and HGfALL:

```
HQfALL[qpgVariables, processes, speciesN]
```

$$-\text{mu}\,q[1]+\text{Apf}[0]\,q[1]-\text{Amf}[0]\,q[1]^2$$

```
HPfALL[qpgVariables, processes, speciesN, variableOfIntegrationFT, dim]
```

$$-2\,\pi\int_0^{\infty}k\,\text{Amf}[k]\,g[1,1,k]\,dk-\text{mu}\,p[1]+\text{Apf}[0]\,p[1]-2\,\text{Amf}[0]\,p[1]\,q[1]$$

```
HGfALL[qpgVariables, processes, speciesM, speciesN, kVariable]
```

$$-2\,\text{mu}\,g[1,1,k]+2\,\text{Apf}[k]\,g[1,1,k]+2\,\text{Apf}[k]\,q[1]-2\,\text{Amf}[0]\,g[1,1,k]\,q[1]-2\,\text{Amf}[k]\,g[1,1,k]\,q[1]-2\,\text{Amf}[k]\,q[1]^2$$

To obtain the part of functions Hq, Hp or Hg, which originate from particular single process within all processes, determine the order number Npr of this process in the list of all processes, for example:

```
Npr = 3;
```

This process corresponds to “DeathByCompetition” process in this example. Now, use the functions HQf, HPf or HGf:

```
HQf[qpgVariables, processes, Npr, speciesN]
```

$$-\text{Amf}[0]\,q[1]^2$$

```
HPf[qpgVariables, processes, Npr, speciesN, variableOfIntegrationFT, dim]
```

$$-2\,\pi\int_0^{\infty}k\,\text{Amf}[k]\,g[1,1,k]\,dk-2\,\text{Amf}[0]\,p[1]\,q[1]$$

```
HGf[qpgVariables, processes, Npr, speciesM, speciesN, kVariable]
```

$$-2\,\text{Amf}[0]\,g[1,1,k]\,q[1]-2\,\text{Amf}[k]\,g[1,1,k]\,q[1]-2\,\text{Amf}[k]\,q[1]^2$$

3.3. Obtain numerical solutions in the chosen equilibrium

3.3.1. Obtain and save results

Load the package “SSPPnumericalResultsForEquilibrium`”.

```
Get["SSPPnumericalResultsForEquilibrium`"]
```

See all processes defined in the package by compiling “?SSPPnumericalResultsForEquilibrium`*”. Clicking on any process in the list will provide its description.

```
? SSPPnumericalResultsForEquilibrium`*
```

▼ SSPPnumericalResultsForEquilibrium`

chosenMeanFieldAndCorrectionsInFourier	qNum
correctionsInReal	qNumericalSolutionsALL
GaussianF	qNumericalSolutionStability
gkNum	speciesNumSSPP
grNum	TophatF
pNum	

In this version of the package only two types of kernels are supported : tophat and gaussian :

```
? TophatF
```

TophatF[IntegralOverAllSpace,BorderR0,dim][k] is

a Fourier transform of a tophat kernel, which in real space corresponds to:

TophatR[IntegralOverAllSpace_,BorderR0_,dim_][r_]:=If[dim==2,If[−BorderR0≤r≤BorderR0, $\frac{\text{IntegralOverAllSpace}}{\pi\ \text{BorderR0}^2}$,0],If[dim==1, If[−BorderR0≤r≤BorderR0, $\frac{\text{IntegralOverAllSpace}}{2\ \text{BorderR0}}$,0],If[dim==3,If[−BorderR0≤r≤BorderR0, $\frac{\text{IntegralOverAllSpace}}{\frac{4}{3}\pi\ \text{BorderR0}^3}$,0]]]];

In Fourier space, TophatF is determined by:

TophatF[IntegralOverAllSpace_,BorderR0_,dim_][k_]:=If[dim==2,If[k==0,IntegralOverAllSpace,IntegralOverAllSpace $\frac{\text{BesselJ}[1,\ 2\ \pi\ \text{k BorderR0}]}{\pi\ \text{k BorderR0}}$],If[dim==1,If[k==0,IntegralOverAllSpace,IntegralOverAllSpace $\frac{\text{Sin}[2\ \text{k}\ \pi\ \text{BorderR0}]}{2\ \text{k}\ \pi\ \text{BorderR0}}$],If[dim==3,If[k==0,IntegralOverAllSpace,IntegralOverAllSpace 3 ($\frac{\text{Sin}[2\ \pi\ \text{k BorderR0}]}{(2\ \pi\ \text{k BorderR0})^3}-\frac{\text{Cos}[2\ \pi\ \text{k BorderR0}]}{(2\ \pi\ \text{k BorderR0})^2}$)]]]];

Parameters:

IntegralOverAllSpace −− it determines the integral of this kernel over the whole space;

BorderR0 −− is the radius which determines the non−zero part of the kernel;

dim − dimensionality of the space where the dynamics is considered. dim can be equal to 1,2, or 3;

k −− radial component of the dim−dimensional wave vector in Fourier space.

```
? GaussianF
```

GaussianF[IntegralOverAllSpace,s][k] is a Fourier transform of a gaussian kernel, which in real space corresponds to:

GaussianR[IntegralOverAllSpace_,s_,dim_][r_]:= $\frac{\text{IntegralOverAllSpace}}{\left(s\sqrt{2\pi}\right)^{\text{dim}}} e^{-r^2/(2 s^2)}$;

and in Fourier space is determined as:

GaussianF[IntegralOverAllSpace_,s_][k_]:=IntegralOverAllSpace $e^{-2\pi^2 k^2 s^2}$;

Parameters:

IntegralOverAllSpace -- it determines the integral of this kernel over whole space;

s -- is a standard deviation;

k -- radial component of the dim-dimensional wave vector in Fourier space.

The user can use a user-defined kernel. For this, the kernel should be defined in real space and in Fourier space in the same format as tophat and gaussian kernels are defined above.

Define all kernels, rates and parameters numerically.

```
dim = 2;
mu = 1;
apIntegralOverAllSpace = 2;
apBorderR0 = 1;
ap = "ap";
Apf[k_] := TophatF[apIntegralOverAllSpace, apBorderR0, dim][k];
amIntegralOverAllSpace = 1;
amBorderR0 = 1;
am = "am";
Amf[k_] := TophatF[amIntegralOverAllSpace, amBorderR0, dim][k];
```

Choose notations for the mean field, the correction to the mean field, and to the leading contribution to cumulants. These can be chosen, for example, as {q,p,g}:

```
qpgVariables = {q, p, g};
```

```
qNumericalSolutionsALL[qpgVariables, processes]
```

```
{{0.}, {1.}}
```

Linear stability of each solution can be tested using the function “qNumericalSolutionStability”

```
solutionNumber = 1;
qNumericalSolutionStability[qpgVariables, processes, solutionNumber]
```

```
Unstable.
```

```
solutionNumber = 2;
qNumericalSolutionStability[qpgVariables, processes, solutionNumber]
```

```
Stable! All eigenvalues are negative.
```

Choose notations for radial components in Fourier spaces, and a dimensionality “dim” of the space where dynamics is considered.

```
variableOfIntegrationFT = k;
kVariable = k;
dim = 2;
```

Also choose the name of the file where results will be saved, it can be the same as chosen in previous section:

```
MyFileName = "TestSystem";
```

Choose the list “kList” of values of the radial component of a wave vector in Fourier space, e.g.

```
kList = Table[k, {k, 0.01, 20.01, 0.1}];
```

Finally, choose the mean field solution, and obtain corrections and cumulants in Fourier space:

```
solutionNumber = 2;

chosenMeanFieldAndCorrectionsInFourier[qpgVariables,
 processes, kList, solutionNumber, dim, MyFileName]
```

Now, the results are written in csv files in the same folder as this notebook.

Cumulants can be obtained in real space using the following function “correctionsInReal”. For this, define a list of values “xList” of radial component of radius vector:

```
xList = Table[x, {x, 0.01, 5.01, 0.1}];

correctionsInReal[qpgVariables, processes, xList, dim, MyFileName]
```

Result is written in csv file in the same folder as this notebook.

3.3.2. Load and plot results

Results can be loaded, for example, by the following way. For species defined by parameter “speciesM”

```
speciesM = 1;
```

the mean field and its correction can be found by functions “qNum” and “pNum”

```
qNum[speciesM, processes, MyFileName]
pNum[speciesM, processes, MyFileName]

1.

-0.23647
```

Cumulant for species “speciesM” and “speciesN” can by found by function “gkNum” in Fourier space, and by function “grNum” in real space. Resulting functions can be plotted in Fourier space:

```
speciesM = 1; speciesN = 1;
gk[speciesM, speciesN] = gkNum[speciesM, speciesN, processes, MyFileName];
ListPlot[gk[speciesM, speciesN], Joined → True,
 PlotRange → All, AxesLabel → {"k", "g[1,1,k]"}]
```

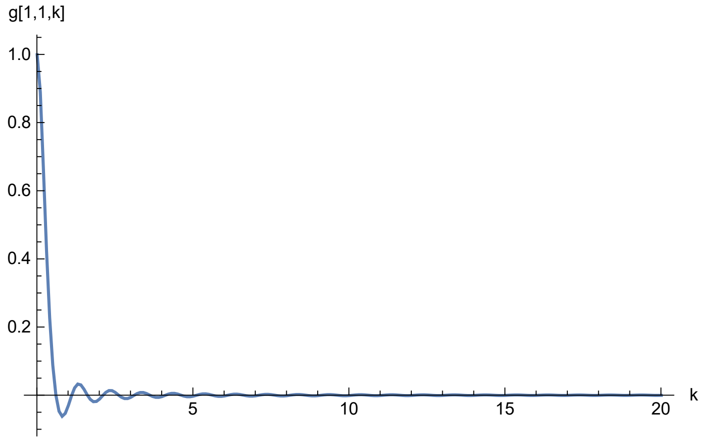

and in real space:

```
speciesM = 1; speciesN = 1;
gr[speciesM, speciesN] = grNum[speciesM, speciesN, processes, MyFileName];
ListPlot[gr[speciesM, speciesN], Joined → True,
 PlotRange → All, AxesLabel → {"r", "g[1,1,r]"}]
```

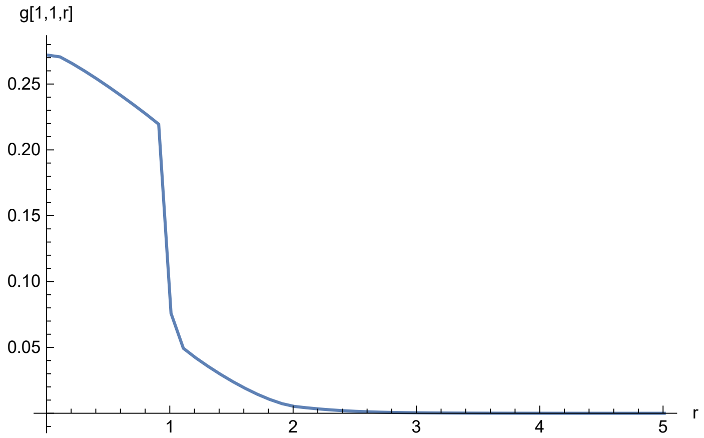

In case of system with many different species, all functions g[m,n] can be plotted as follows in Fourier space:

```
species = speciesNumSSPP[processes];
$m = 0;
Do[$m++; $n = $m; Do[
    gk[species[[$m]], species[[$n]]] =
     gkNum[species[[$m]], species[[$n]], processes, MyFileName];
    Print[ListPlot[gk[species[[$m]], species[[$n]]], Joined → True,
      PlotRange → All, AxesLabel → {"k", None}, PlotLabel → ToString["g[" <>
         ToString[species[[$m]]] <> "," <> ToString[species[[$n]]] <> ",k]"]]];
    $n++, {Length[species] + 1 - $m}], {Length[species]}];
```

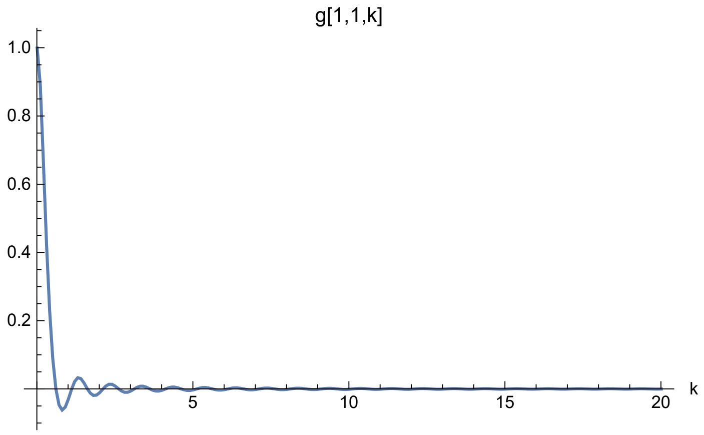

and in real space:

```
(*Load all functions gr, and plot them*)
species = speciesNumSSPP[processes];
$m = 0;
Do[$m++; $n = $m; Do[
    gr[species[[$m]], species[[$n]]] =
     grNum[species[[$m]], species[[$n]], processes, MyFileName];
    Print[ListPlot[gr[species[[$m]], species[[$n]]], Joined → True,
      PlotRange → All, AxesLabel → {"r", None}, PlotLabel → ToString["g[" <>
         ToString[species[[$m]]] <> "," <> ToString[species[[$n]]] <> ",r]"]]];
    $n++, {Length[species] + 1 - $m}], {Length[species]}];
```

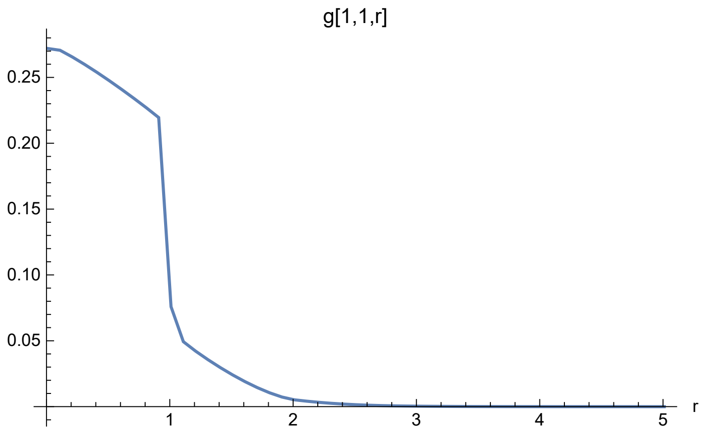

4. Define a new model component

4.1. New model component to produce TEX file

Follow instructions shown in the Appendix B.1. After the groups ``Products'', ``Reactants'' and ``Catalysts'' are constructed, and after rates and kernels of the processes are identified, the new model component can be constructed following the examples of definitions of model components from the package "SSPPlibraryOfProcessesForTeX`" shown below. Compile the cell with the definition of the new model component before defining the full model by the variable ``processes''. It is recommended to avoid using capital letters at the beginning of the names denoting rates.

```
(* species s1 immigrates with rate r*Coeffic,
where Coeffic is the overall prefactor to this process. *)
Immigration[s1_, r_, Coeffic_] := Module[{Products = {{s1, "x1"}}, Reactants = {},
   Catalysts = {}, listAll, function, Interactions, name},
  listAll = {Products, Reactants, Catalysts};
  function[x1_] := r;
  Interactions = r;
  name = "Immigration";
  {listAll, function, Interactions, name, Coeffic}]
```

```
(* species s1 produces species s1(itself) with the kernel b,
this is facilitated by species s3 with the kernel a,
both kernels are in real space. Af and Bf are Fourier transforms of kernels a
 and b respectively, Coeffic is the overall prefactor to this process. *)
BirthByFacilitation[s1_, s3_, a_, Af_, b_, Bf_, Coeffic_] :=
  Module[{Products = {{s1, "x1"}}, Reactants = {},
    Catalysts = {{s1, "x2"}, {s3, "x3"}}, listAll, function, Interactions, name},
   listAll = {Products, Reactants, Catalysts};
   function[x1_, x2_, x3_] := Style[a, FontSlant → "Italic"][x3 - x2]
     Style[b, FontSlant → "Italic"][x2 - x1];
   Interactions = {{Style[a, FontSlant → "Italic"],
      Style[Af, FontSlant → "Italic"], "x3", "x2"}, {Style[b,
       FontSlant → "Italic"], Style[Bf, FontSlant → "Italic"], "x2", "x1"}};
   name = "BirthByFacilitation";
   {listAll, function, Interactions, name, Coeffic}];
```

```
(* s1 infects s2 with the kernel a, s2 becomes infected
 and turns into s1. Af is a Fourier transform of the kernel a,
Coeffic is the overall prefactor to this process. *)
Infection[s1_, s2_, a_, Af_, Coeffic_] :=
  Module[{Products = {{s1, "x2"}}, Reactants = {{s2, "x2"}},
    Catalysts = {{s1, "x3"}}, listAll, function, Interactions, name},
   listAll = {Products, Reactants, Catalysts};
   function[x2_, x3_] := Style[a, FontSlant → "Italic"][x2 - x3];
   Interactions = {{Style[a, FontSlant → "Italic"],
      Style[Af, FontSlant → "Italic"], "x2", "x3"}};
   name = "Infection";
   {listAll, function, Interactions, name, Coeffic}];
```

To check the correctness of definitions of groups Products, Reactants and Catalysts, the user can apply the function “TestTheCorrectnessOfProcesses” to the processes, for example:

```
Get["SSPPlibraryOfProcessesForTeX`"];

processes = {Birth[1, ap, Apf, 1],
   DensityIndependentDeath[1, mu, 1], DeathByCompetition[1, am, Amf, 1]};

TestTheCorrectnessOfProcesses[processes]

No errors in species or coordinates were found. The correctness of
  interactions was not tested. Make sure that interactions are correct.
```

An example of a mistaken process where groups Products, Reactants and Catalysts contain errors is shown below:

```
MistakenProcessForTex[s1_, s2_, s3_, a_, Af_, Coeffic_] := Module[
   {Products = {{s1, "x2"}, {s3, "x2"}}, Reactants = {{s2, "x2"}, {s3, "x2"}},
    Catalysts = {{s1, "x3"}, {s3, "x2"}}, listAll, function, Interactions, name},
   listAll = {Products, Reactants, Catalysts};
   function[x2_, x3_] := Style[a, FontSlant → "Italic"][x2 - x3];
   Interactions = {{Style[a, FontSlant → "Italic"],
      Style[Af, FontSlant → "Italic"], "x2", "x3"}};
   name = "Infection";
   {listAll, function, Interactions, name, Coeffic}];

processes = {Birth[1, ap, Apf, 1], MistakenProcessForTex[1, 2, 3, a, Af, 1]};

TestTheCorrectnessOfProcesses[processes]

Warning! 5 errors were found!
 List of errors:
 Error 1 -- In the component 2: same coordinates in P.
 Error 2 -- In the component 2: same coordinates in R.
 Error 3 -- In the component 2: same coordinates in C and P.
 Error 4 -- In the component 2: same coordinates in C and R.
 Error 5 -- In the component 2: completely similar pairs in P and R.
```

4.2. New model component to use in analytical and numerical calculations in Mathematica

Follow instructions shown in the Appendix B.1. After the groups ``Products'', ``Reactants'' and ``Catalysts'' are constructed, and after rates and kernels of the processes are identified, the new model component can be constructed following the examples of definitions of model components from the package "SSPPlibraryOfProcesses`" shown below. Compile the cell with the definition of the new model component before defining the full model by the variable ``processes''. It is recommended to avoid using capital letters at the beginning of the names denoting rates.

```
(* species s1 immigrates with rate r*Coeffic,
where Coeffic is the overall prefactor to this process. *)
Immigration[s1_, r_, Coeffic_] := Module[{Products = {{s1, x1}}, Reactants = {},
   Catalysts = {}, listAll, function, Interactions, name},
  listAll = {Products, Reactants, Catalysts};
  function[x1_] := r;
  Interactions = r;
  name = "Immigration";
  {listAll, function, Interactions, name, Coeffic}]
```

```
(* species s1 produces species s1(itself) with the kernel b,
this is facilitated by species s3 with the kernel a,
both kernels are in real space. Af and Bf are Fourier transforms of kernels a
 and b respectively, Coeffic is the overall prefactor to this process. *)
BirthByFacilitation[s1_, s3_, a_, Af_, b_, Bf_, Coeffic_] :=
  Module[{Products = {{s1, x1}}, Reactants = {},
    Catalysts = {{s1, x2}, {s3, x3}}, listAll, function, Interactions, name},
   listAll = {Products, Reactants, Catalysts};
   function[x1_, x2_, x3_] := a[x3 - x2] b[x2 - x1];
   Interactions = {{a, Af, x3, x2}, {b, Bf, x2, x1}};
   name = "BirthByFacilitation";
   {listAll, function, Interactions, name, Coeffic}];
```

```
(* s1 infects s2 with the kernel a, s2 becomes infected
 and turns into s1. Af is a Fourier transform of the kernel a,
Coeffic is the overall prefactor to this process. *)
Infection[s1_, s2_, a_, Af_, Coeffic_] :=
  Module[{Products = {{s1, x2}}, Reactants = {{s2, x2}},
    Catalysts = {{s1, x3}}, listAll, function, Interactions, name},
   listAll = {Products, Reactants, Catalysts};
   function[x2_, x3_] := a[x2 - x3];
   Interactions = {{a, Af, x2, x3}};
   name = "Infection";
   {listAll, function, Interactions, name, Coeffic}];
```

To check the correctness of definitions of groups Products, Reactants and Catalysts, the user can apply the function "TestTheCorrectnessOfProcesses" to the processes, for example:

```
Get["SSPPlibraryOfProcesses`"];

processes = {Birth[1, ap, Apf, 1],
   DensityIndependentDeath[1, mu, 1], DeathByCompetition[1, am, Amf, 1]};

TestTheCorrectnessOfProcesses[processes]

No errors in species or coordinates were found. The correctness of
  interactions was not tested. Make sure that interactions are correct.
```

An example of a mistaken process where groups Products, Reactants and Catalysts contain errors is shown below:

```
MistakenProcess[s1_, s2_, s3_, a_, Af_, Coeffic_] :=
  Module[{Products = {{s1, x2}, {s3, x2}}, Reactants = {{s2, x2}, {s3, x2}},
    Catalysts = {{s1, x3}, {s3, x2}}, listAll, function, Interactions, name},
   listAll = {Products, Reactants, Catalysts};
   function[x2_, x3_] := a[x2 - x3];
   Interactions = {{a, Af, x2, x3}};
   name = "Infection";
   {listAll, function, Interactions, name, Coeffic}];

processes = {Birth[1, ap, Apf, 1], MistakenProcess[1, 2, 3, a, Af, 1]};

TestTheCorrectnessOfProcesses[processes]

Warning! 5 errors were found!
 List of errors:
 Error 1 -- In the component 2: same coordinates in P.
 Error 2 -- In the component 2: same coordinates in R.
 Error 3 -- In the component 2: same coordinates in C and P.
 Error 4 -- In the component 2: same coordinates in C and R.
 Error 5 -- In the component 2: completely similar pairs in P and R.
```

Supplementary Note 3

The case study 1 “Optimal landscape connectivity”

Contents

3.1 The definition of the model **131**
3.2 Generating simulations from “The model simulator” toolbox **132**
3.3 Generating model equations from “The model constructor” toolbox. **134**
3.4 The research question and results **138**
3.4.1 Occupancy-connectivity correlation 139
3.4.2 Optimal connectivity kernel 142
3.4.3 Bessel function colonisation kernel 143
3.4.4 Other colonisation kernels 144
3.4.5 Optimal length scale for connectivity kernel of a given shape 146

3.1 The definition of the model

The model involves two types of patches: unoccupied (denoted by 1) and occupied (denoted by 2). Unoccupied patches are created spontaneously by immigration (with rate r), which is described by a model component “Immigration” and by the operator $L_1^{\mathrm{IM}}(r)$. Unoccupied patches are converted to occupied patches due to the colonisation with colonisation kernel c from other occupied patches. The kernel c is assumed to be a symmetric function, i.e. $c(-x) = c(x)$. This process is described by the model component “Infection” and the operator $L_{21}^{\mathrm{I}}(c)$. Occupied patches die naturally at rate e, reverting to unoccupied patches. This is described by the model component “Change In Type” and the corresponding operator $L_{12}^{\mathrm{CT}}(e)$. Both types of patch can vanish at rate μ, which is described by a model component “Density Independent Death” and by the operator $L_i^{\mathrm{D}}(\mu)$, for $i = 1, 2$. Thus, the model is defined by the following operator L,

$$L = L_1^{\mathrm{IM}}(r) + L_{21}^{\mathrm{I}}(c) + L_{12}^{\mathrm{CT}}(e) + L_1^{D}(\mu) + L_2^{D}(\mu). \quad (249)$$

3.2 Generating simulations from "The model simulator" toolbox

The detailed tutorial for "The model simulator" toolbox is provided in Supplementary Note 2, section 2.2. In this section we demonstrate a brief description of how simulations can be obtained and analyzed using "The model simulator" toolbox.

First, for "The model simulator" toolbox to use the model definition (249), such a definition should be written into the .txt file as shown below. The model definition (249) is equivalent to the following sequence: Immigration[1, r], Infection[2, 1, c], ChangeInType[1, 2, e], DensityIndependentDeath[1, mu], DensityIndependentDeath[2, mu]. All symbols should be substituted by numerical values. Using, for example, the following numerical values of model parameters,

$$r = 1; \quad \mu = 0.9; \quad e = 0.5; \tag{250}$$

and defining c as a tophat kernel, which is defined by two arguments "cIntegral" and "cRadius",

$$c = \text{tophat}[\text{cIntegral}, \text{cRadius}], \tag{251}$$

$$\text{cIntegral} = 2; \quad \text{cRadius} = 1, \tag{252}$$

the definition of the model can be written into the .txt file shown in Supplementary Figure 7.

```
Immigration[1, 1]
Infection[2, 1, tophat[2, 1]]
ChangeInType[1, 2, 0.5]
DensityIndependentDeath[1, 0.9]
DensityIndependentDeath[2, 0.9]
```

Supplementary Figure 7: A content of the file "modelCaseStudy1.txt" which defines the model for "The model simulator" toolbox. All model component are listed without commas.

Once the system is defined by the txt file, the toolbox can be used. For simplicity of this demonstration, all necessary commands needed to create a single simulation of the dynamics of the system discussed here are presented in the file "CaseStudy1.R" (available on request), partially shown in Supplementary Figure 8(a). The dimensionality of the space (here we consider a 2-dimensional space) is also defined in "CaseStudy1.R" file.

(a)

(b)

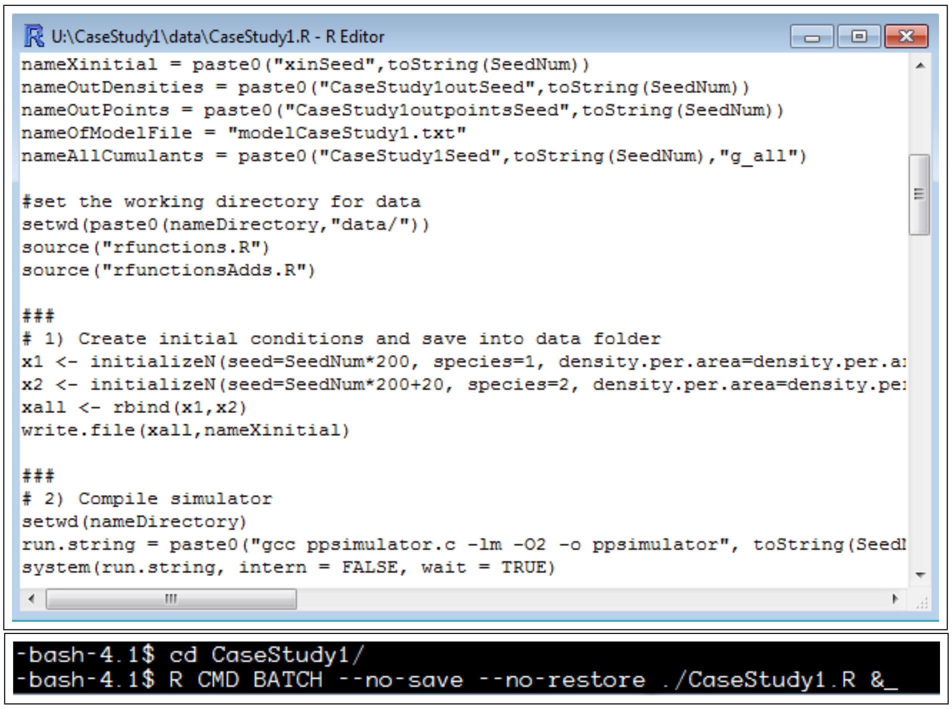

```
U:\CaseStudy1\data\CaseStudy1.R - R Editor
nameXinitial = paste0("xinSeed",toString(SeedNum))
nameOutDensities = paste0("CaseStudy1outSeed",toString(SeedNum))
nameOutPoints = paste0("CaseStudy1outpointsSeed",toString(SeedNum))
nameOfModelFile = "modelCaseStudy1.txt"
nameAllCumulants = paste0("CaseStudy1Seed",toString(SeedNum),"g_all")

#set the working directory for data
setwd(paste0(nameDirectory,"data/"))
source("rfunctions.R")
source("rfunctionsAdds.R")

###
# 1) Create initial conditions and save into data folder
x1 <- initializeN(seed=SeedNum*200, species=1, density.per.area=density.per.a
x2 <- initializeN(seed=SeedNum*200+20, species=2, density.per.area=density.pe
xall <- rbind(x1,x2)
write.file(xall,nameXinitial)

###
# 2) Compile simulator
setwd(nameDirectory)
run.string = paste0("gcc ppsimulator.c -lm -O2 -o ppsimulator", toString(Seed
system(run.string, intern = FALSE, wait = TRUE)
```

```
-bash-4.1$ cd CaseStudy1/
-bash-4.1$ R CMD BATCH --no-save --no-restore ./CaseStudy1.R &_
```

Supplementary Figure 8: (a) Screenshot of the file “CaseStudy1.R” containing necessary commands to use “The model simulator” toolbox. (b) Commands in UNIX terminal to run the R file shown in (a).

Simulations can be started by running the commands in the terminal shown in Supplementary Figure 8(b), also see the Tutorial in Supplementary Note 2, section 2.2. Some examples of how results can be visualised are presented in Supplementary Figure 9(a)-(b). Other ways to generate simulations using “The model simulator” toolbox and to analyze results using R are presented in the Tutorial in Supplementary Note 2, section 2.2.

(a) 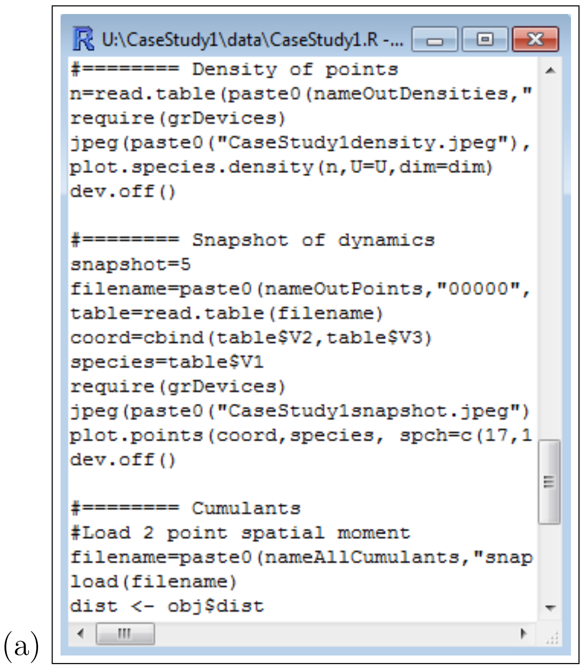

(b) 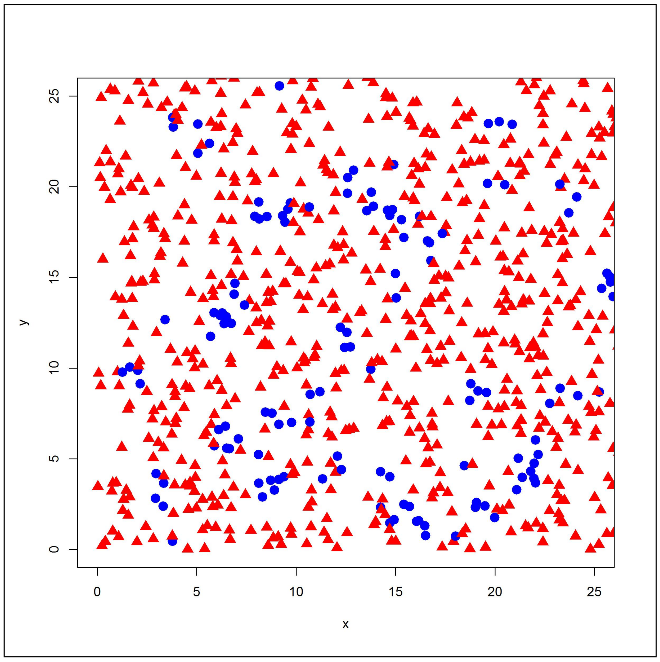

Supplementary Figure 9: Examples of visualization of data obtained from simulations: (a) generating a snapshot of dynamics using R; (b) the resulting snapshot of the system defined in Supplementary Figure 7.

3.3 Generating model equations from "The model constructor" toolbox.

Here we present the application of "The model constructor" toolbox to this case study. In "The model constructor" toolbox the model (249) is defined by the variable named "processes" and shown in Supplementary Figure 10.

```
processes ={Immigration[1, r, 1],
Infection[2, 1, c, $c, 1],
ChangeInType[1, 2, e, 1],
DensityIndependentDeath[1, mu, 1],
DensityIndependentDeath[2, mu, 1]};
```

Supplementary Figure 10: The variable 'processes' defines the model (249) in "The model constructor" toolbox. There is the following link between parameters here and in the definition (249): r = r, c = c, $c denotes $\tilde{c}$ the Fourier transform of c, e = e, mu = μ.

One can obtain model equations saved in .tex file by compiling commands shown in Supplementary Figure 11. As a result, the model equations in real and Fourier spaces are written in corresponding .tex files, resulting in .pdf files shown in the Supplementary Figure 12. Equations can be obtained in real and in Fourier spaces, for all model components together or for each model component separately, e.g. the contribution from a single model component "Infection" to the function H_{q_1} is shown in Supplementary Figure 12(c)-(d).

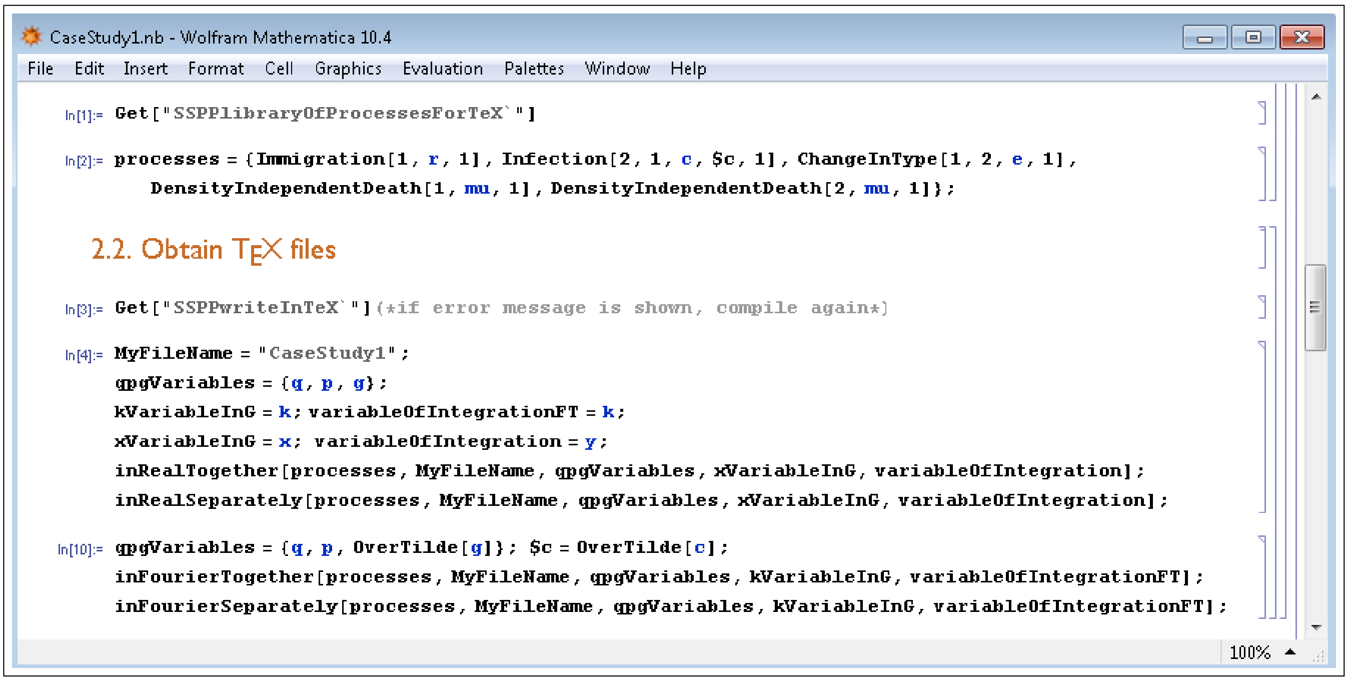

Supplementary Figure 11: Screenshot of some commands from the Mathematica file “CaseStudy1.nb” (available on request) used to create and save analytical expressions for model equations into .tex files. Here the function $\tilde{g}$ is the Fourier transform of the cumulant g.

Analytical expressions for model equations can be obtained for further transformations in Mathematica, for example see Supplementary Figure 13. Using numerical values (250) and (252) for parameters of the model, the toolbox “The model constructor” can be used to calculate numerical values of densities in all possible equilibria in the system, and to identify the linear stability of each equilibrium, as shown in the Supplementary Figure 14.

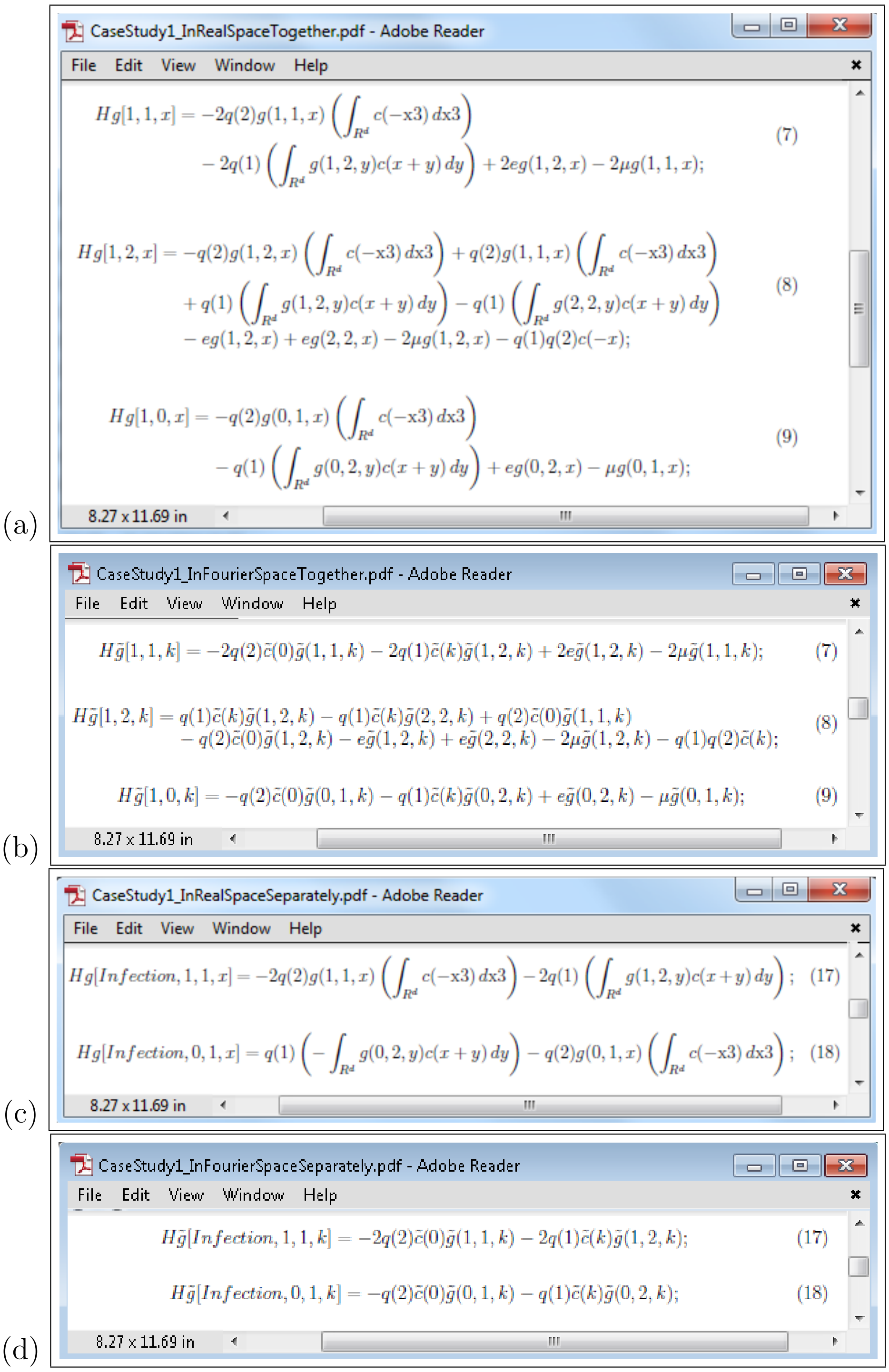

Supplementary Figure 12: Screenshots of .pdf files obtained from .tex files generated by the commands in Supplementary Figure 11. The contributions to the model equations from all model components are considered together in: (a) real space, (b) Fourier space; and separately in: (c) real space, (d) Fourier space. Here, $Hg[1,1,x]$ is $H_{g_{11}}(x)$ from the equation for the cumulant $g_{11}(x)$, $dg_{11}(x)/dt = H_{g_{11}}(x)$; functions $H\tilde{g}$ and $\tilde{g}$ are Fourier transforms of Hg and g respectively. Species denoted by 0 are considered as species which are not described by the model definition (249).

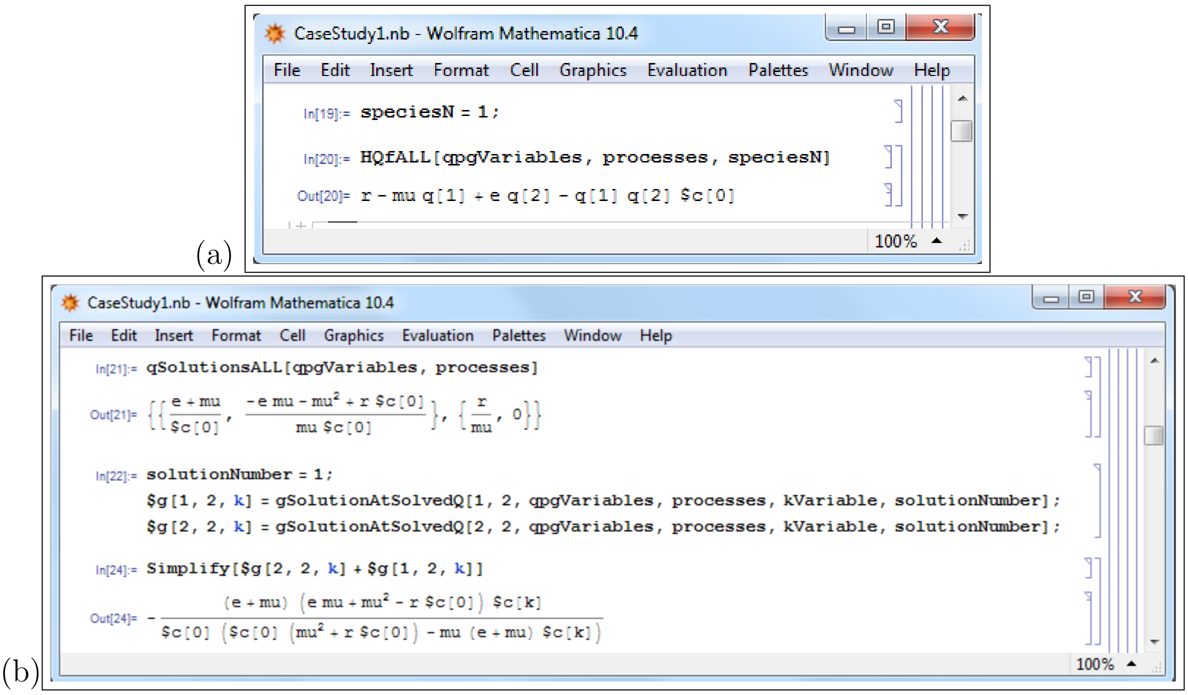

Supplementary Figure 13: Examples of analytical expressions for the model equations: (a) the function H_{q_1}; (b) the densities $\{q_1, q_2\}$ at two possible equilibria. Using "The model constructor" toolbox the analytical expressions can be obtained for all H functions, for corrections to mean field densities, and for cumulants in all possible equilibria. Here $\$c$ and $\$g$ denote Fourier transforms of c and g.

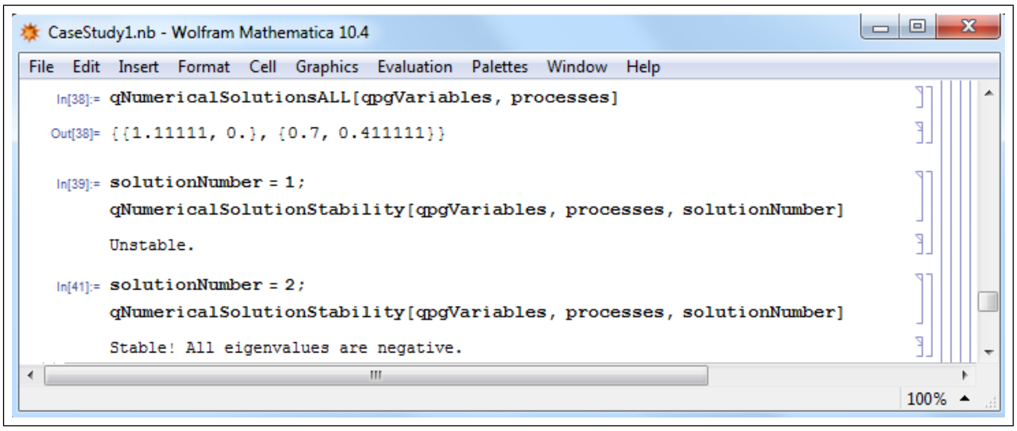

Supplementary Figure 14: Two equilibria are found, with densities shown in form of a vector $\{q_1, q_2\}$. Equilibrium with $q_2 = 0$ is unstable with respect to infinitesimally small perturbations. In the equilibrium with $q_1 = 0.7$ and $q_2 \approx 0.4$ all corresponding stability eigenvalues are negative, thus this equilibrium is stable.

Second spatial moments (found from cumulants and densities at equilibrium) and corrections to the mean field densities can be calculated in any equilibrium found by the toolbox. For a stable equilibrium this is demonstrated in the Supplementary Figure 15.

(a)

(b)

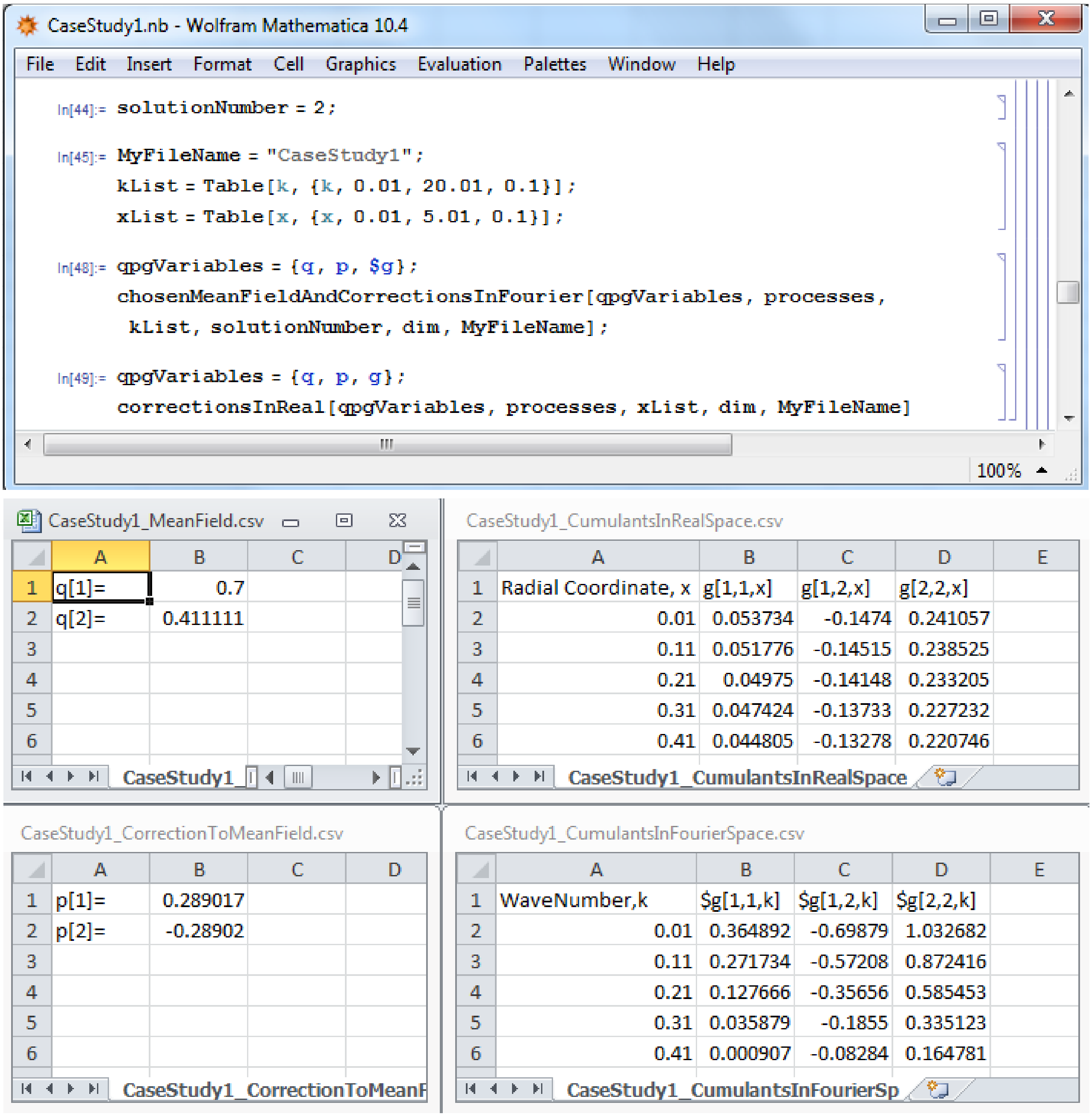

Supplementary Figure 15: Derivation of numerical results for a given equilibrium using "The model constructor" toolbox. (a) The stable equilibrium is chosen. Cumulants are calculated in points defined by "kList" variable in Fourier space and by "xList" variable in real space. The compilations of commands shown in (a) result in .csv files shown in (b). Here $\$g$ denotes Fourier transform of g.

3.4 The research question and results

We are interested in how well connectivity measures can predict whether a metapopulation patch will be occupied or not. We do this by first computing an expression for the correlation coefficient between a connectivity measure and the patch occupancy, and then exploring how this depends on the model parameters and the choice of connectivity measure. We are particularly

interested in finding the optimal choice of connectivity kernel (i.e. the one for which connectivity is the best predictor of occupancy), and in how much worse the correlation is for non-optimal choices of connectivity kernel.

3.4.1 Occupancy-connectivity correlation

In this section we define the occupancy-connectivity correlation, show how it can be expressed in general in terms of spatial moments, and then compute the leading behavior for our model from the quantities p, q, g in our perturbation expansion.

Let $\sigma(x)$ be the occupancy of a patch at position x, i.e. a quantity that is 0 if the patch is unoccupied and 1 if it is occupied, and let $S(x)$ be the connectivity measured at position x. For a metapopulation where the patches are located as a set of points γ_{A}, the product-moment correlation between the occupancy and connectivity is then

$$\begin{aligned} R &= \frac{\left\langle \left(\sigma(x) - \langle\sigma(x)\rangle_{x\in\gamma_{\mathrm{A}}}\right)\left(S(x) - \langle S(x)\rangle_{x\in\gamma_{\mathrm{A}}}\right)\right\rangle_{x\in\gamma_{\mathrm{A}}}}{\left[\left\langle(\sigma(x) - \langle\sigma(x)\rangle_{x\in\gamma_{\mathrm{A}}})^2\right\rangle_{x\in\gamma_{\mathrm{A}}}\left\langle(S(x) - \langle S(x)\rangle_{x\in\gamma_{\mathrm{A}}})^2\right\rangle_{x\in\gamma_{\mathrm{A}}}\right]^{1/2}} \\ &= \frac{\langle\sigma(x)S(x)\rangle_{x\in\gamma_{\mathrm{A}}} - \langle\sigma(x)\rangle_{x\in\gamma_{\mathrm{A}}}\langle S(x)\rangle_{x\in\gamma_{\mathrm{A}}}}{\left[(\langle\sigma^2(x)\rangle_{x\in\gamma_{\mathrm{A}}} - \langle\sigma(x)\rangle^2_{x\in\gamma_{\mathrm{A}}})(\langle S^2(x)\rangle_{x\in\gamma_{\mathrm{A}}} - \langle S(x)\rangle^2_{x\in\gamma_{\mathrm{A}}})\right]^{1/2}}, \end{aligned}$$

where $\langle\cdot\rangle_{x\in\gamma_{\mathrm{A}}}$ denotes an average over all patches in the landscape, i.e. $\langle Z(x)\rangle_{x\in\gamma_{\mathrm{A}}} = \frac{\sum_{x\in\gamma_{\mathrm{A}}} Z(x)}{|\gamma_{\mathrm{A}}|}$.

Each term in this expression can be computed in terms of the moments of the system. Following reference [11] we introduce the notation $k(\eta_{\mathrm{u}}, \eta_{\mathrm{o}})$, where η_{u} denotes a set of locations of unoccupied patches and η_{o} the location of a set of occupied patches, to represent a moment function of order $m = |\eta_{\mathrm{u}} \cup \eta_{\mathrm{o}}|$ (the sum of the numbers of points in η_{u} and η_{o}). Thus, $k(\{x\}, \varnothing)$ is the mean density of unoccupied patches measured at x, $k(\varnothing, \{x\})$ is the mean density of occupied patches measured at x, $k(\{x\},\{y\})$ is the second moment for unoccupied patches at position x and occupied patches at position y, etc. For brevity, we also define the moments $k_{\mathrm{A}}(\eta)$ for the unmarked process (i.e. disregarding whether patches are occupied or unoccupied), so that, for example,

$$\begin{aligned} k_{\mathrm{A}}(\{x\}) &= k(\{x\},\varnothing) + k(\varnothing,\{x\}) \\ k_{\mathrm{A}}(\{x,y\}) &= k(\{x,y\},\varnothing) + k(\{x\},\{y\}) + k(\{y\},\{x\}) + k(\varnothing,\{x,y\}) \\ k_{\mathrm{A}}(\{x,y,z\}) &= k(\{x,y,z\},\varnothing) + k(\{x,y\},\{z\}) + k(\{x,z\},\{y\}) + k(\{y,z\},\{x\}) \\ &\quad + k(\{x\},\{y,z\}) + k(\{y\},\{x,z\}) + k(\{z\},\{x,y\}) + k(\varnothing,\{x,y,z\}). \end{aligned}$$

We denote by γ the configuration of the system, i.e. the set of locations and point types for a given state. We denote by γ_{o} and γ_{u}, respectively, the sets of locations of occupied and unoccupied patches in configuration γ, and $\gamma_{\mathrm{A}} = \gamma_{\mathrm{o}} \cup \gamma_{\mathrm{u}}$ the set of locations of all patches. Therefore, $\sigma(x) = 1$ for $x \in \gamma_{\mathrm{o}}$, and $\sigma(x) = 0$ for $x \in \gamma_{\mathrm{u}}$. The mean occupancy is the number of occupied patches in a

large volume Ω, divided by the number of all patches in Ω, in the limit $\Omega \to 0$:

$$
\begin{aligned}
\langle \sigma \rangle_{\gamma_\mathrm{A}} &= \lim_{|\Omega|\to\infty} \frac{\int_\Gamma \sum_{x\in\gamma_\mathrm{o}} \mathbb{1}_\Omega(x) d\mu(\gamma)}{|\Omega| k_\mathrm{A}(\{x\})} \\
&= \frac{k(\varnothing, \{x\})}{k_\mathrm{A}(\{x\})} \\
\left\langle \sigma^2 \right\rangle_{x\in\gamma_\mathrm{A}} - \langle \sigma \rangle^2_{x\in\gamma_\mathrm{A}} &= \langle \sigma \rangle_{x\in\gamma_\mathrm{A}} \left(1 - \langle \sigma \rangle_{x\in\gamma_\mathrm{A}}\right),
\end{aligned}
$$

where we have used the fact that $\sigma \in \{0,1\}$ so $\sigma^2 = \sigma$.

The landscape connectivity for a landscape γ_A, measured at a point $x \in \gamma_\mathrm{A}$, is $S(x) = \sum_{y\in\gamma_\mathrm{A}\backslash x} h(x-y)$, so

$$
\begin{aligned}
\langle S(x) \rangle &= \langle \sum_{y\in\gamma_\mathrm{A}\backslash x} h(x-y) \rangle_{x\in\gamma_\mathrm{A}} \\
&= \lim_{|\Omega|\to\infty} \frac{\int_\Gamma \sum_{x\in\gamma_\mathrm{A}\cap\Omega} \sum_{y\in\gamma_\mathrm{A}\backslash x} h(x-y) d\mu(\gamma)}{|\Omega| k_\mathrm{A}(\{x\})} \\
&= \lim_{|\Omega|\to\infty} \frac{\int_\Gamma \sum_{x\in\gamma_\mathrm{A}} \sum_{y\in\gamma_\mathrm{A}\backslash x} \mathbb{1}_\Omega(x) h(x-y) d\mu(\gamma)}{|\Omega| k_\mathrm{A}(\{x\})} \\
&= \frac{1}{k_\mathrm{A}(\{x\})} \int_{\mathbb{R}^d} h(x-y) k_\mathrm{A}(\{x,y\}) dy \\
\langle S^2(x) \rangle &= \langle \sum_{y\in\gamma_\mathrm{A}\backslash x} h(x-y) \sum_{z\in\gamma_\mathrm{A}\backslash x} h(x-z) \rangle_{x\in\gamma_\mathrm{A}} \\
&= \lim_{|\Omega|\to\infty} \frac{\int_\Gamma \sum_{x\in\gamma_\mathrm{A}} \left(\sum_{y\in\gamma_\mathrm{A}\backslash x} \sum_{z\in\gamma_\mathrm{A}\backslash x\backslash y} h(x-y)h(x-z) + \sum_{y\in\gamma_\mathrm{A}\backslash x} (h(x-y))^2 \right) d\mu(\gamma)}{|\Omega| k_\mathrm{A}(\{x\})} \\
&= \frac{1}{k_\mathrm{A}(\{x\})} \int_{\mathbb{R}^d} \int_{\mathbb{R}^d} h(x-y)h(x-z) \left(k_\mathrm{A}(\{x,y,z\}) \, dy \, dz\right. \\
&+ \frac{1}{k_\mathrm{A}(\{x\})} \int_{\mathbb{R}^d} h^2(x-y) \left(k_\mathrm{A}(\{x,y\}) \, dy\right.
\end{aligned}
$$

$$
\begin{aligned}
\langle \sigma(x) S(x) \rangle &= \langle \sum_{y\in\gamma_\mathrm{A}\backslash x} h(x-y) \rangle_{x\in\gamma_\mathrm{o}} \\
&= \lim_{|\Omega|\to\infty} \frac{\int_\Gamma \sum_{x\in\gamma_\mathrm{o}} \left(\sum_{y\in\gamma_\mathrm{A}\backslash x} h(x-y) \right) d\mu(\gamma)}{|\Omega| k_\mathrm{A}(\{x\})} \\
&= \frac{1}{k_\mathrm{A}(\{x\})} \int_{\mathbb{R}^d} h(x-y) \left(k(\{y\},\{x\}) + k(\varnothing,\{x,y\})\right) dy \\
&= \frac{1}{k_\mathrm{A}(\{x\})} \int_{\mathbb{R}^d} h(x-y) \left(u(\{y\},\{x\}) + u(\varnothing,\{x,y\}) + k(\varnothing,\{x\}) k_\mathrm{A}(\{y\})\right) dy.
\end{aligned}
$$

The above expressions are valid for any dynamic landscape patch-occupancy metapopulation. We now show how to compute the leading contribution from the perturbation expansion in our

model. This expansion takes the form

$$
\begin{aligned}
k(\varnothing,\{x\}) &= q_{\mathrm{u}} + \epsilon^d p_{\mathrm{u}} + o(\epsilon^d)\\
k(\{x\},\varnothing) &= q_{\mathrm{o}} + \epsilon^d p_{\mathrm{o}} + o(\epsilon^d)\\
k(\{x,y\},\varnothing) &= k(\{x\},\varnothing)k(\{y\},\varnothing) + u(\{x,y\},\varnothing)\\
&= q_{\mathrm{u}}^2 + \epsilon^d g_{\mathrm{uu}}(x-y) + o(\epsilon^d)\\
k(\{x\},\{y\}) &= k(\{x\},\varnothing)k(\varnothing,\{y\}) + u(\{x\},\{y\})\epsilon^d g_{\mathrm{uo}}(x-y) + o(\epsilon^d)\\
&= q_{\mathrm{u}}q_{\mathrm{o}} + \epsilon^d g_{\mathrm{uo}}(x-y) + o(\epsilon^d)\\
k(\varnothing,\{x,y\}) &= k(\varnothing,\{x\})k(\varnothing,\{y\}) + u(\varnothing,\{x,y\})\\
&= q_{\mathrm{o}}^2 + \epsilon^d g_{\mathrm{oo}}(x-y) + o(\epsilon^d),
\end{aligned}
$$

where $u(\eta_{\mathrm{u}},\eta_{\mathrm{o}})$ is a cumulant of order $m = |\eta_{\mathrm{u}} \cup \eta_{\mathrm{o}}|$. Using the model constructor to evaluate (see Supplementary Figure 13b), we have

$$
\begin{aligned}
q_{\mathrm{u}} &= \frac{e+\mu}{\tilde{c}(0)}\\
&= P_0 Q_0\\
q_{\mathrm{o}} &= \frac{r\tilde{c}(0) - \mu(e+\mu)}{\mu\tilde{c}(0)}\\
&= (1-P_0)Q_0\\
\tilde{g}_{\mathrm{oe}}(\omega) + \tilde{g}_{\mathrm{oo}}(\omega) &= -\frac{(e+\mu)\left(\mu(e+\mu) - r\tilde{c}(0)\right)\tilde{c}(\omega)}{\tilde{c}(0)\left(\tilde{c}(0)\left(\mu^2 + r\tilde{c}(0)\right) - \mu(e+\mu)\tilde{c}(\omega)\right)}\\
&= \frac{Q_0 P_0 (1-P_0)}{\phi(\omega)(1+\rho) - (1-P_0)},
\end{aligned}
$$

where $\tilde{g}(\omega) = \int_{\mathbb{R}^d} \mathrm{e}^{2\pi \mathrm{i} x\cdot\omega} g(x)dx$ is the Fourier transform of $g(x)$, and

$$
P_0 = 1 - \frac{\mu(e+\mu)}{\tilde{c}(0)r} \tag{253}
$$

$$
\begin{aligned}
Q_0 &= \frac{r}{\mu}\\
\phi(\omega) &= \frac{\tilde{c}(0)}{\tilde{c}(\omega)}
\end{aligned}
$$

$$
\rho = \frac{1-P_0}{1+\frac{e}{\mu}}, \tag{254}
$$

where $\tilde{c}(\omega) = \int_{\mathbb{R}^d} \mathrm{e}^{2\pi \mathrm{i} x\cdot\omega} c(x)dx$. The quantities P_0 and Q_0 represent, respectively, the mean-field patch occupancy [10] and the density of patches in the landscape.

The moments k_{A} of the unmarked process take a particularly simple form, because the patch dynamics (disregarding whether they are occupied or unoccupied) is a simple immigration-death process that introduces no spatial correlations. Therefore, since the density of patches at equilib-

rium is Q_0, we get

$$k_{\mathrm{A}}(\{x\}) = Q_0$$
$$k_{\mathrm{A}}(\{x,y\}) = Q_0^2$$
$$k_{\mathrm{A}}(\{x,y,z\}) = Q_0^3$$

These results combine to form an expression for the connectivity-occupancy correlation to leading order:

$$R = \frac{\int_{\mathbb{R}^d} \frac{\tilde{h}(\omega) Q_0 P_0 (1-P_0)}{\phi(\omega)(1+\rho)-(1-P_0)} d\omega}{\left(Q_0^3 P_0 (1-P_0) \int_{\mathbb{R}^d} \tilde{h}^2(\omega) d\omega\right)^{\frac{1}{2}}}, \tag{255}$$

where $\tilde{h}(\omega) = \int_{\mathbb{R}^d} \mathrm{e}^{2\pi \mathrm{i} x \cdot \omega} h(x) dx$, and we have converted integrals in real space to integrals in Fourier space using the identity

$$\int_{\mathbb{R}^d} f(x) g(x) dx = \int_{\mathbb{R}^d} \tilde{f}(\omega) \tilde{g}(\omega) d\omega,$$

where $\tilde{f}$ and $\tilde{g}$ are the Fourier transforms of any functions f and g.

Proof that correlation weakens as landscape becomes more dynamic

From Eq. (255), the derivative of the connectivity-occupancy correlation with respect to ρ, keeping the patch density Q_0 and the mean-field patch occupancy P_0 constant, is

$$\frac{\partial R}{\partial \rho} = -\frac{1}{\left(Q_0^3 P_0 (1-P_0) \int_{\mathbb{R}^d} \tilde{h}^2(\omega) d\omega\right)^{\frac{1}{2}}} \int_{\mathbb{R}^d} \tilde{h}(\omega) \frac{\phi(\omega) Q_0 P_0 (1-P_0)}{\left(\phi(1+\rho)-(1-P_0)\right)^2} d\omega.$$

The expression on the right hand side is always negative, so R decreases when ρ is increased. From eqn. (254), the quantity ρ increases monotonically as the landscape is made more dynamic (keeping P_0 constant), being equal to 0 when the landscape is static (when $\mu = 0$) and $1-P_0$ when the landscape is maximally dynamic (when $e/\mu = 0$, i.e. patches only go extinct when they are destroyed). Therefore, the correlation R gets weaker when the landscape is made more dynamic, provided P_0 and Q_0 are kept constant.

3.4.2 Optimal connectivity kernel

Using equation (255) we can ask the question: for a given set of parameters (i.e. keeping P_0, Q_0, ρ constant), and a given colonisation kernel c, what choice of connectivity kernel h leads to the strongest correlation R? We restrict ourselves to the class of functions with spherical symmetry, i.e. $\tilde{h}(\omega) = \tilde{h}(w)$ where $w = |\omega|$. We adopt a variational approach to find the value of $\tilde{h}$ for which this expression is maximal.

We write $\tilde{h}(w) = \tilde{h}_0(w) + \nu\delta(w - w_1)$, with $\nu \ll 1$, and expand in powers of ν so that

$$\begin{aligned} R &\propto \frac{1}{\left(\int_{\mathbb{R}^d} \tilde{h}^2(w) w^{d-1} dw\right)^{\frac{1}{2}}} \int_{\mathbb{R}^d} \tilde{h}(w) \frac{1}{\phi(1+\rho) - (1-P_0)} w^{d-1} dw \\ &= \frac{1}{\left(\int_{\mathbb{R}^d} \tilde{h}_0^2(w) w^{d-1} dw\right)^{\frac{1}{2}}} \left[1 - \frac{\nu \tilde{h}_0(w_1) w_1^{d-1}}{\int_{\mathbb{R}^d} \tilde{h}_0^2(w) w^{d-1} dw}\right] \times \\ &\times \left[\int_{\mathbb{R}^d} \frac{\tilde{h}_0(w) w^{d-1}}{\phi(1+\rho) - (1-P_0)} dw + \frac{\nu w_1^{d-1}}{\phi(w_1)(1+\rho) - (1-P_0)}\right] + o(\epsilon). \end{aligned}$$

If $\tilde{h}_0 = \tilde{h}_\star$, the optimal choice of connectivity kernel, we must have $\frac{dR}{d\nu} = 0$, so the order ν term in the expansion of R must be zero. Therefore, the optimal choice $\tilde{h}_0$ satisfies

$$0 = -\frac{\tilde{h}_\star(w_1) w_1^{d-1}}{\int_{\mathbb{R}^d} \tilde{h}_\star^2(w) w^{d-1} dw} \int_{\mathbb{R}^d} \frac{\tilde{h}_\star(w) w^{d-1}}{\phi(1+\rho) - (1-P_0)} dw + \frac{1}{\phi(w_1)(1+\rho) - (1-P_0)} w_1^{d-1}.$$

This must be satisfied for all w_1, so since the integrals in this equation are independent of w_1 we deduce that the optimal kernel choice is

$$\tilde{h}_\star(\omega) = \frac{A}{\phi(\omega)(1+\rho) - (1-P_0)}, \tag{256}$$

where A is a constant. The choice of A is arbitrary — it only changes the connectivity measure S by an overall factor, and does not affect R.

3.4.3 Bessel function colonisation kernel

In most cases, $\tilde{h}_\star(\omega)$ will have a different functional form from $1/\phi(\omega)$ and, therefore, from $\tilde{c}(\omega)$. In dimension 2, the exception is when $c \propto K_0(x/l_c)$, where K_0 is a modified Bessel function of the second kind and order zero and l_c is the characteristic length scale of the colonisation process, in which case

$$\tilde{c}(\omega) = \frac{1}{1 + (2\pi\omega l_c)^2}.$$

This leads to

$$\begin{aligned} \tilde{h}_\star(\omega) &= \frac{A}{P_0 + \rho}\left(1 + \frac{(1+\rho)}{\rho + P_0}(2\pi\omega l_c)^2\right)^{-1} \\ \Rightarrow h_\star(x) &\propto K_0(x/\theta_\star), \end{aligned}$$

where

$$\theta_\star = l_c \left(\frac{1+\rho}{\rho + P_0}\right)^{1/2}. \tag{257}$$

Since $P_0 \leq 1$, we have $\theta_\star \geq \frac{1}{\epsilon}$, so the optimal connectivity kernel has a longer typical length scale than the colonisation kernel. This length scale decreases monotonically as P_0 is increased if ρ is kept fixed. For fixed P_0, ρ increases monotonically as patch turnover is made more rapid, with $\rho = 0$ when the landscape is static ($\mu \to 0$) and $\rho = 1 - P_0$ when the landscape is maximally

dynamic ($e = 0$). Therefore, we have

$$\theta_\star = \begin{cases} \frac{l_c}{P_0^{1/2}} & \text{for } \mu{=}0 \\ l_c(2-P_0)^{1/2} & \text{for } e = 0. \end{cases}$$

Thus, we see that, for a static landscape, the optimal connectivity kernel diverges when $P_0 \to 0$. This is because the correlation length of the system diverges when P_0 approaches zero, so the best predictor of occupancy needs to take account of the landscape structure at larger and larger scales. By contrast, when the landscape is maximally dynamic ($e = 0$) the length scale of the length scale of the optimal connectivity kernel is only between 1 and $\sqrt{2}$ times the length scale of the colonisation kernel. This is because the landscape turnover makes it impossible for very long range correlations to build up in the system.

To illustrate how sensitive the correlation R is to a non-optimal choice of connectivity kernel, we can compute R in closed form when the colonisation and connectivity kernels are both modified bessel functions of the second kind and order zero, i.e. $c(x) \propto K_0(x/l_c)$ and $h(x) = K_0(x/\theta)$, so that $\tilde{h}(\omega) \propto \frac{1}{1+(2\pi\omega\theta)^2}$ and $\phi(\omega) = 1 + (2\pi\omega l_c)^2$ Substituting these forms for $\tilde{h}$ and ϕ into eqn. (255) gives

$$\begin{aligned} R &= \left(\frac{P_0(1-P_0)}{\pi Q_0}\right) \frac{\theta}{(\rho+P_0)(\theta^2-\theta_\star^2)} \log\frac{\theta}{\theta_\star} \\ &= R_{\max}\psi\left(\frac{\theta}{\theta_\star}\right), \end{aligned}$$

where $\theta_\star$ is the optimal value of θ for this colonisation kernel, $R_{\max}$ is the value of R where $\theta = \theta_\star$, and

$$\psi(x) = \frac{2\log x}{x - \frac{1}{x}} \tag{258}$$

is a symmetric function of $\log x$ with maximum unity at $x = 1$. As shown in Supplementary Figure 16, if θ differs from its optimal value by a factor of about 10, then the correlation is reduced by a factor of about 0.4; if θ differs by a factor of about 100 from its optimal value then the correlation reduces by a factor of about 0.1.

3.4.4 Other colonisation kernels

For other colonisation kernels, the optimal connectivity kernel takes a different shape from the colonisation kernel. For example, for an exponential colonisation kernel $c(x) \propto e^{-x/\lambda}$, we have $\tilde{c}(\omega) \propto \left(1 + \left(\frac{2\pi\omega}{\lambda}\right)^2\right)^{-3/2}$, and

$$H_\star = \frac{A}{(1+\rho)\left(1+(\frac{2\pi\omega}{\lambda})^2\right)^{3/2} - (1-P_0)}.$$

This cannot be written in the form $(1+(b\omega)^2)^{-3/2}$ for some constant b, so $h_\star$ is not exponential in shape. Nevertheless, we expect that the the optimal connectivity length scale to depend on model parameters in a qualitatively similar way to $\theta_\star$ in Eq. (257), even for other kernel shapes.

Moreover, for a wide class of colonisation kernels the correlation R will take the same value as for a Bessel colonisation function K_0 with the same variance, in a particular parameter limit. To

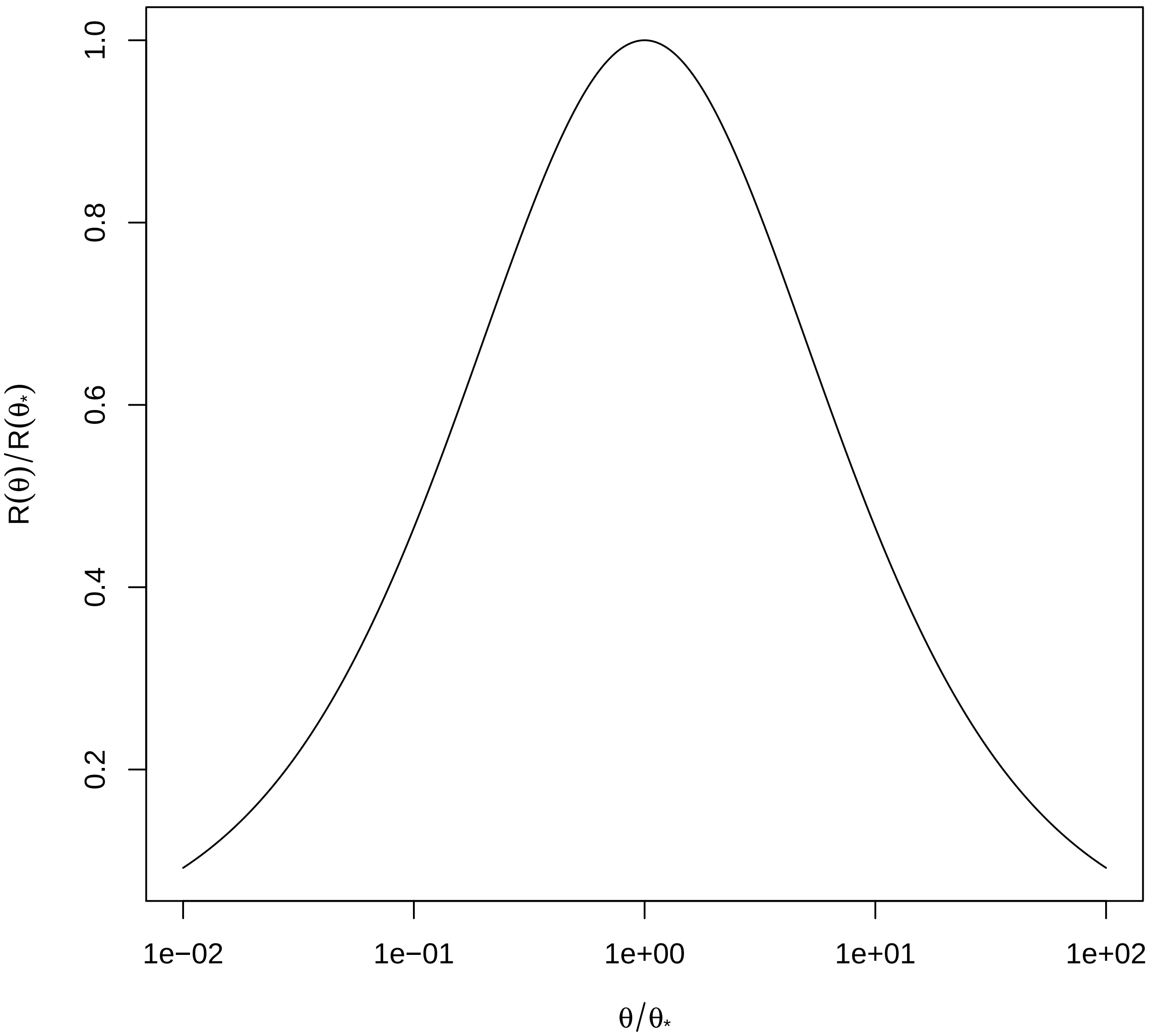

Supplementary Figure 16: Ratio of connectivity-occupancy correlation to that with the optimal kernel, from eqn. (258). Here, the colonisation kernel is of the form $c \propto K_0(x/l)$, $R(\theta)$ is the connectivity-occupancy correlation for the optimal kernel choice $h \propto K_0(x/\theta_\star)$, and $R(\theta)$ is for the connectivity kernel $h \propto K_0(x/\theta)$).

see this, note that the Fourier transform in 2D for circular symmetry takes the form

$$\begin{aligned}F(\omega) &= \int_0^\infty \int_0^{2\pi} e^{2\pi i\omega x\cos\theta} f(x) d\theta x\, dx \\ &= \int_0^\infty \int_0^{2\pi} \left(1 + 2\pi i\omega x\cos\theta - 2\pi^2\omega^2 x^2\cos^2\theta + \dots\right) f(x) d\theta x\, dx \\ &= \left(1 - \pi^2\omega^2 V_f + O(V_f^2\omega^4)\right) \int_0^\infty f(x) 2\pi x\, dx,\end{aligned}$$

where

$$V_f = \frac{\int_0^\infty f(x) 2\pi x^3\, dx}{\int_0^\infty f(x) 2\pi x\, dx}$$

is the variance (over 2D space) of the kernel f. Therefore,

$$\begin{aligned}\int_{\mathbb{R}^2} \frac{\tilde{h}(\omega) Q_0 P_0 (1 - P_0)}{\phi(\omega)(1+\rho) - (1 - P_0)} d\omega &= \int_0^\infty \frac{\tilde{h}(\omega) Q_0 P_0 (1 - P_0)}{\frac{(1+\rho)}{(1-\pi^2\omega^2 V_c + O(V_c^2\omega^4))} - (1 - P_0)} 2\pi\omega\, d\omega \\ &= \int_0^\infty \frac{\tilde{h}(\omega) Q_0 P_0 (1 - P_0)}{\rho + P_0 + (1+\rho)\left(\pi^2\omega^2 V_c + O(V_c^2\omega^4)\right)} 2\pi\omega\, d\omega \\ &= (1+\rho) V_c \int_0^\infty \frac{\tilde{h}\left(\omega' \left(\frac{\rho + P_0}{V_c(1+\rho)}\right)^{1/2}\right) Q_0 P_0 (1 - P_0)}{1 + \pi^2\omega'^2 V_c + O\left(\omega'^4 \left(\frac{\rho+P_0}{1+\rho}\right)\right)} 2\pi\omega\, d\omega.\end{aligned}$$

Thus, if $\left(\frac{\rho+P_0}{1+\rho}\right) \to 0$, the integral approaches the value

$$\int_{\mathbb{R}^2} \frac{\tilde{h}(\omega) Q_0 P_0 (1 - P_0)}{\phi(\omega)(1+\rho) - (1 - P_0)} d\omega \to (1+\rho) V_c \int_0^\infty \frac{\tilde{h}\left(\omega' \left(\frac{\rho + P_0}{V_c(1+\rho)}\right)^{1/2}\right) Q_0 P_0 (1 - P_0)}{1 + \pi^2\omega'^2 V_c} 2\pi\omega\, d\omega,$$

provided the integral exists. This integral is the same as if the colonisation kernel were $c(x) \propto K_0(2x/V_c^{1/2})$. This kernel has variance

$$\begin{aligned}\frac{\int_0^\infty K_0(2x/V_c^{1/2}) 2\pi x^3\, dx}{\int_0^\infty K_0(2x/V_c^{1/2}) 2\pi x\, dx} &= \frac{V_c}{4} \frac{\int_0^\infty K_0(z) z^3\, dz}{\int_0^\infty K_0(z) z\, dz} \\ &= V_c\end{aligned}$$

(Integrals evaluated using formula 11.4.22 in *Abramowitz M., Stegun I. A. Handbook of Mathematical Functions with Formulas, Graphs, and Mathematical Tables. Wiley—Interscience, New York, 1972*).

As discussed above, the regime where$\left(\frac{\rho+P_0}{1+\rho}\right) \ll 1$ when the landscape turnover is much slower than colonisation/extinction dynamics ($\mu \ll e$) and, in addition, the patch occupancy is low.

3.4.5 Optimal length scale for connectivity kernel of a given shape

In practice, the colonisation kernel is unlikely to be known in enough detail to compute the optimal connectivity kernel $h_\star$. Instead, the connectivity kernels is assumed to take a convenient shape such as the exponential form $e^{-x/\lambda}$, where λ is the typical length scale of the connectivity

kernel. One can still ask the question: how does the correlation R between connectivity and occupancy depend on the choice of λ, and for which value of λ is the correlation the strongest? This can be studied by numerically evaluating the integrals in Eqn. (255) (see the main text, Figure 3B).

Suppose that we have an exponential connectivity kernel $h(x) = \mathrm{e}^{-x/\lambda}$. The variance of this exponential kernel in 2D space is

$$\frac{\int_0^\infty \mathrm{e}^{-\frac{x}{\lambda}} 2\pi x^3\, dx}{\int_0^\infty \mathrm{e}^{-\frac{x}{\lambda}} 2\pi x\, dx} = 6\lambda^2.$$

Meanwhile, the variance of a top-hat kernel with lengthscale L, $\mathrm{TopHat}(x, L)$, is

$$\frac{\int_0^L 2\pi x^2 x\, dx}{\int_0^L 2\pi x\, dx} = \frac{1}{2} L^2.$$

Therefore, if we choose a top-hat connectivity kernel $\mathrm{TopHat}(x, l_\mathrm{c}\sqrt{12})$, it will have the same variance as the connectivity kernel when $l_\mathrm{c} = \lambda$.

If the optimal value of λ were sensitive to the variances of the kernels only, then it could be predicted from the analytical prediction (257) for modified Bessel functions of the second kind and order zero. The function $K_0(x(2/3)^{1/2}/\lambda)$ would have the same variance as the exponential connectivity kernel given above, and the function $K_0(x(2/3)^{1/2}/l_\mathrm{c})$ would have the same variance as this colonisation kernel. Therefore we would predict that the optimal value of λ would be

$$\lambda_\star = l_\mathrm{c} \left(\frac{1+\rho}{\rho + P_0} \right)^{1/2}.$$

As can be seen from Figure 3B in the main text, this formula does not typically give a good approximation to the observed optimal value of λ, showing that the full kernel shape is important in determining the optimal length scale for the connectivity kernel. The exception is the case where the landscape is static and where occupancy is low ($\mu/e = 0$, $P_0 = 0.167$, black solid line in the main text Figure 3B), which, as explained above, is the regime where R is expected to be well approximated by the result for Bessel function kernels.

Supplementary Note 4

The case study 2 “Genetic similarity”

Contents

4.1 The definition of the model **148**
4.2 Generating simulations from “The model simulator” toolbox **150**
4.3 Generating model equations from “The model constructor” toolbox **153**
4.4 The research question and results **159**

4.1 The definition of the model

The model involves two habitat types (a and b), and four species represented by haploid individuals with 4 different haploid genotypes. The genotypes involve a neutral locus (with alleles 1 and 2) and a non-neutral locus (with alleles A and B). Thus, four species are denoted as A1, A2, B1, B2. The detailed definition of dynamics of habitats and species is shown below.

Habitats

The two habitat types are assumed to follow identical immigration-extinction dynamics, where immigration occurs with rate κ, and density independent mortality has rate μ. Therefore, dynamics of habitat a is described by two model components, “Immigration” and “Density Independent Death”, which in terms of operators L is written as $L_{\mathrm{a}}^{\mathrm{IM}}(\kappa) + L_{\mathrm{a}}^{\mathrm{D}}(\mu)$. Denoting a set of habitat types by $\mathcal{H} = \{\mathrm{a}, \mathrm{b}\}$, the dynamics of all types of habitat is described by the following sum of operators:

$$\sum_{i\in\mathcal{H}} \left(L_i^{\mathrm{IM}}(\kappa) + L_i^{\mathrm{D}}(\mu)\right). \tag{259}$$

Species

The dynamics of the species is described by the logistic growth model, where the mortality and reproduction of species are modelled as follows.

Mortality

The mortality is assumed to be independent of habitat availability. It is modeled by two model components. The first model component is given by density independent death with rate

m which is identical for all genotypes. Denoting a set of all genotypes by $\mathcal{G} = \{\mathrm{A1}, \mathrm{A2}, \mathrm{B1}, \mathrm{B2}\}$, and performing summation over all genotypes, the contribution of this model component to the full model definition is given by the following sum:

$$\sum_{j \in \mathcal{G}} L_j^{\mathrm{D}}(m). \tag{260}$$

The second model component is given by density dependent mortality, where the density of all types of genotypes should be taken into account. This means that individuals with type k genotype induce death in individuals with type j genotype with certain kernel c (here notation c stands for "competition"). The kernel c is assumed to be a symmetric function, $c(-x) = c(x)$. When type k is the same as type j, such a process corresponds to the "Death By Competition" model component denoted as $L_j^{\mathrm{C}}(c)$. In a general case when types k and j can be any genotypes from the set $\mathcal{G}$ such a process corresponds to "Death By External Factor" model component and to the operator $L_{jk}^{\mathrm{DE}}(c)$. Performing summation over possible values of j and k, the contribution of the density-dependent mortality to the full definition of the model is given by the following sum:

$$\sum_{j \in \mathcal{G}} \sum_{k \in \mathcal{G}} L_{jk}^{\mathrm{DE}}(c), \tag{261}$$

where it is taken into account that $L_j^{\mathrm{C}}(c) = L_{jj}^{\mathrm{DE}}(c)$.

Reproduction

First, it is assumed that at the time of reproduction offspring species disperse, and the establishment takes place with probability one (i.e. the establishment is independent of the habitat type). Second, the fecundity is assumed to depend on habitat availability and the match between the habitat type and the genotype of the parent individual. Last, it is assumed that the reproduction is clonal with random mutation between the alleles at the two loci.

To take into account these assumptions, the appropriate model component is constructed. Particularly, at the time of reproduction the individual of type k_1 genotype can produce a new individual of any type i genotype. The offspring species disperse according to certain dispersal kernel d (here the notation d stands for "dispersal"). Also, the reproduction is facilitated by the type k_2 habitat which is located near the parent k_1 genotype within the range of the patch kernel r (r stands for "resource"). Both kernels are assumed to be symmetric, $d(-x) = d(x)$, $r(-x) = r(x)$. The model component for such a process is called "Birth To Another Type By Facilitation", and the corresponding operator is $L_{ik_1k_2}^{\mathrm{BTF}}(r, d)$. Notice, that in case when the genotypes k_1 and i are identical ($k_1 = i$) the process is called "Birth By Facilitation", and the corresponding operator is $L_{ik_2}^{\mathrm{BF}}(r, d)$, and $L_{ik_2}^{\mathrm{BF}}(r, d) = L_{iik_2}^{\mathrm{BTF}}(r, d)$. The rate of this process is defined by contributions from *dispersal, adaptation* and *mutation* components which are discussed below.

Dispersal The rate of the process depends on the dispersal kernel d which is included in the operator $L_{ik_1k_2}^{\mathrm{BTF}}(r, d)$ as a second argument.

Adaptation The dependence of the fecundity of individuals on habitat availability is described by the patch kernel r, which depends only on distance between the parent individual and a habitat. To model the dependence of the fecundity on the match between the type of habitat and type of the parent individual we use a parameter ϕ_{k_1,k_2}^{τ} which denotes the ability of an individual with genotype k_1 to use the habitat type k_2. The rate of reproduction described by the operator $L_{ik_1k_2}^{\mathrm{BTF}}(r, d)$ is multiplied by ϕ_{k_1,k_2}^{τ}. We assume that the allele A (from non-neutral locus) allows the individual to utilize the habitat a with efficiency $(1 + \tau) f_0$ and the habitat b with efficiency

$(1-\tau)f_0$ (and vice versa for allele B), where $0 < \tau < 1$ is a parameter measuring the strength of local adaptation, f_0 is the baseline fecundity rate. Thus, the parameter $\phi^{\tau}_{k_1,k_2}$ can have the following values, $\phi^{\tau}_{\mathrm{A1},a} = \phi^{\tau}_{\mathrm{A2},a} = \phi^{\tau}_{\mathrm{B1},b} = \phi^{\tau}_{\mathrm{B2},b} = (1+\tau)f_0$, $\phi^{\tau}_{\mathrm{A1},b} = \phi^{\tau}_{\mathrm{A2},b} = \phi^{\tau}_{\mathrm{B1},a} = \phi^{\tau}_{\mathrm{B2},a} = (1-\tau)f_0$.

Mutation It is assumed that the alleles can mutate to another allele from the same locus at the time of the reproduction. The mutation probability, denoted by ν, is assumed to be the same for both loci and symmetric between the alleles. Thus, the probability that allele in any locus will stay the same equals to $(1-\nu)$, and the probability that it will change into another allele from this locus equals to ν. The total probability of mutation of one genotype to another genotype equals to the product of probabilities for mutation for each allele. We use the symbol $\psi^{\nu}_{k_1,i}$ to denote the probability that the offspring has genotype i if the parent individual has genotype k_1. The values of $\psi^{\nu}_{(k_1,i)}$ form a 4×4 matrix, with values such as $\psi^{\nu}_{\mathrm{A1,A1}} = (1-\nu)^2$, $\psi^{\nu}_{(\mathrm{A1,A2})} = \psi^{\nu}_{(\mathrm{A1,B1})} = (1-\nu)\nu$. $\psi^{\nu}_{(\mathrm{A1,B2})} = \nu^2$. Thus, the rate of reproduction described by the operator $L^{\mathrm{BTF}}_{ik_1k_2}(r,d)$ has a pre-factor $\psi^{\nu}_{k_1,i}$.

Taking into account dispersal, adaptation and mutation, the reproduction described by the model component "Birth To Another Type By Facilitation" contributes the following term to the full definition of the model:

$$\sum_{i\in\mathcal{G}}\sum_{k_1\in\mathcal{G}}\sum_{k_2\in\mathcal{H}}\phi^{\tau}_{k_1,k_2}\psi^{\nu}_{k_1,i}L^{\mathrm{BTF}}_{ik_1k_2}(r,d). \tag{262}$$

The definition of the model

Contributions (259)-(262) form the following operator L which defines the model,

$$L = \sum_{i\in\mathcal{H}}\left(L^{\mathrm{IM}}_i(\kappa) + L^{\mathrm{D}}_i(\mu)\right) + \sum_{j\in\mathcal{G}}L^{\mathrm{D}}_j(m) + \sum_{j\in\mathcal{G}}\sum_{k\in\mathcal{G}}L^{\mathrm{DE}}_{jk}(c) + \sum_{i\in\mathcal{G}}\sum_{k_1\in\mathcal{G}}\sum_{k_2\in\mathcal{H}}\phi^{\tau}_{k_1,k_2}\psi^{\nu}_{k_1,i}L^{\mathrm{BTF}}_{ik_1k_2}(r,d). \tag{263}$$

4.2 Generating simulations from "The model simulator" toolbox

In this section we demonstrate how to use "The model simulator" toolbox to perform the individual-based simulations of the dynamics of the system defined by (263). First, species and habitats are denoted by the following numbers: $\mathcal{G} = \{1,2,3,4\}$, i.e. genotypes $\{\mathrm{A1,A2,B1,B2}\}$ correspond to species $\{1,2,3,4\}$, and $\mathcal{H} = \{5,6\}$, i.e. habitats $\{a,b\}$ are considered as species $\{5,6\}$. Second, all model components from (263) should be defined numerically, for example using the following values of parameters:

$$\nu = 0.01;\quad \kappa = \mu = 1;\quad \tau = 0.1;\quad f_0 = 1;\quad m = 1. \tag{264}$$

We choose to use tophat kernels, which are defined by two arguments, 'Integral' and 'Radius', where 'Integral' equals the integral of this kernel over the whole space, and 'Radius' defines the radius of area where the kernel has non-zero constant value (detailed definition is presented in the Tutorial in Supplementary Note 2, section 2.2),

$$\begin{aligned} r&= \mathrm{tophat}[\mathrm{rIntegral},\mathrm{rRadius}],\\ d&= \mathrm{tophat}[\mathrm{dIntegral},\mathrm{dRadius}],\\ c&= \mathrm{tophat}[\mathrm{cIntegral},\mathrm{cRadius}], \end{aligned} \tag{265}$$

where

$$\text{rIntegral} = 1; \qquad \text{rRadius} = 6;$$
$$\text{dIntegral} = 1; \qquad \text{dRadius} = 6;$$
$$\text{cIntegral} = 1; \qquad \text{cRadius} = 20. \tag{266}$$

Next, all model components are written in file with .txt extension. In this case study such a file is "modelCaseStudy2.txt", which is shown in the Supplementary Figure 17. When creating such

```
BirthToAnotherTypeByFacilitation[1,1,5,tophat[1,6],tophat[1.07811,6]]
BirthToAnotherTypeByFacilitation[1,2,5,tophat[1,6],tophat[0.01089,6]]
BirthToAnotherTypeByFacilitation[1,3,5,tophat[1,6],tophat[0.00891,6]]
BirthToAnotherTypeByFacilitation[1,4,5,tophat[1,6],tophat[0.00009,6]]
BirthToAnotherTypeByFacilitation[2,1,5,tophat[1,6],tophat[0.01089,6]]
BirthToAnotherTypeByFacilitation[2,2,5,tophat[1,6],tophat[1.07811,6]]
BirthToAnotherTypeByFacilitation[2,3,5,tophat[1,6],tophat[0.00009,6]]
BirthToAnotherTypeByFacilitation[2,4,5,tophat[1,6],tophat[0.00891,6]]
BirthToAnotherTypeByFacilitation[3,1,5,tophat[1,6],tophat[0.01089,6]]
BirthToAnotherTypeByFacilitation[3,2,5,tophat[1,6],tophat[0.00011,6]]
BirthToAnotherTypeByFacilitation[3,3,5,tophat[1,6],tophat[0.88209,6]]
BirthToAnotherTypeByFacilitation[3,4,5,tophat[1,6],tophat[0.00891,6]]
BirthToAnotherTypeByFacilitation[4,1,5,tophat[1,6],tophat[0.00011,6]]
BirthToAnotherTypeByFacilitation[4,2,5,tophat[1,6],tophat[0.01089,6]]
BirthToAnotherTypeByFacilitation[4,3,5,tophat[1,6],tophat[0.00891,6]]
BirthToAnotherTypeByFacilitation[4,4,5,tophat[1,6],tophat[0.88209,6]]
BirthToAnotherTypeByFacilitation[1,1,6,tophat[1,6],tophat[0.88209,6]]
BirthToAnotherTypeByFacilitation[1,2,6,tophat[1,6],tophat[0.00891,6]]
BirthToAnotherTypeByFacilitation[1,3,6,tophat[1,6],tophat[0.01089,6]]
BirthToAnotherTypeByFacilitation[1,4,6,tophat[1,6],tophat[0.00011,6]]
BirthToAnotherTypeByFacilitation[2,1,6,tophat[1,6],tophat[0.00891,6]]
BirthToAnotherTypeByFacilitation[2,2,6,tophat[1,6],tophat[0.88209,6]]
BirthToAnotherTypeByFacilitation[2,3,6,tophat[1,6],tophat[0.00011,6]]
BirthToAnotherTypeByFacilitation[2,4,6,tophat[1,6],tophat[0.01089,6]]
BirthToAnotherTypeByFacilitation[3,1,6,tophat[1,6],tophat[0.00891,6]]
BirthToAnotherTypeByFacilitation[3,2,6,tophat[1,6],tophat[0.00009,6]]
BirthToAnotherTypeByFacilitation[3,3,6,tophat[1,6],tophat[1.07811,6]]
BirthToAnotherTypeByFacilitation[3,4,6,tophat[1,6],tophat[0.01089,6]]
BirthToAnotherTypeByFacilitation[4,1,6,tophat[1,6],tophat[0.00009,6]]
BirthToAnotherTypeByFacilitation[4,2,6,tophat[1,6],tophat[0.00891,6]]
BirthToAnotherTypeByFacilitation[4,3,6,tophat[1,6],tophat[0.01089,6]]
BirthToAnotherTypeByFacilitation[4,4,6,tophat[1,6],tophat[1.07811,6]]
DensityIndependentDeath[1,1] DensityIndependentDeath[2,1]
DensityIndependentDeath[3,1] DensityIndependentDeath[4,1]
DeathByExternalFactor[1,1,tophat[1,20]] DeathByExternalFactor[1,2,tophat[1,20]]
DeathByExternalFactor[1,3,tophat[1,20]] DeathByExternalFactor[1,4,tophat[1,20]]
DeathByExternalFactor[2,1,tophat[1,20]] DeathByExternalFactor[2,2,tophat[1,20]]
DeathByExternalFactor[2,3,tophat[1,20]] DeathByExternalFactor[2,4,tophat[1,20]]
DeathByExternalFactor[3,1,tophat[1,20]] DeathByExternalFactor[3,2,tophat[1,20]]
DeathByExternalFactor[3,3,tophat[1,20]] DeathByExternalFactor[3,4,tophat[1,20]]
DeathByExternalFactor[4,1,tophat[1,20]] DeathByExternalFactor[4,2,tophat[1,20]]
DeathByExternalFactor[4,3,tophat[1,20]] DeathByExternalFactor[4,4,tophat[1,20]]
Immigration[5,1] Immigration[6,1]
DensityIndependentDeath[5,1] DensityIndependentDeath[6,1]
```

Supplementary Figure 17: A content of the txt file which defines the model for "The model simulator" toolbox. All model component are listed without comas.

a file, all pre-factors to the model components "BirthToAnotherTypeByFacilitation" are included in the definition of 'dIntegral' parameter of d kernel, i.e.

$$\sum_{i\in\mathcal{G}}\sum_{k_1\in\mathcal{G}}\sum_{k_2\in\mathcal{H}}\phi^{\tau}_{k_1,k_2}\psi^{\nu}_{k_1,i}L^{\text{BTF}}_{ik_1k_2}(r,d) = \sum_{i\in\mathcal{G}}\sum_{k_1\in\mathcal{G}}\sum_{k_2\in\mathcal{H}}L^{\text{BTF}}_{ik_1k_2}(r,d^{\tau\nu}_{ik_1k_2}), \tag{267}$$

where $d^{\tau\nu}_{ik_1k_2}$ is a new kernel, $d^{\tau\nu}_{ik_1k_2}(x) = \phi^{\tau}_{k_1,k_2}\psi^{\nu}_{k_1,i}d(x)$. The kernel $d^{\tau\nu}_{ik_1k_2}$ is therefore defined as

$$d^{\tau\nu}_{ik_1k_2} = \text{tophat}[\phi^{\tau}_{k_1,k_2}\psi^{\nu}_{k_1,i}\text{dIntegral}, \text{dRadius}]. \tag{268}$$

As one can see in Supplementary Figure 17, the first argument of tophat kernel can take one of the following 6 possible values due to different mutation and adaptation:

$$\begin{aligned}
\nu^2(1-\tau) &= 0.00009;\\
\nu^2(1+\tau) &= 0.00011;\\
(1-\nu)\nu(1-\tau) &= 0.00891;\\
(1-\nu)\nu(1+\tau) &= 0.01089;\\
(1-\nu)^2(1-\tau) &= 0.88209;\\
(1-\nu)^2(1+\tau) &= 1.07811.
\end{aligned} \tag{269}$$

Once the system is defined by the txt file, the toolbox can be used. For simplicity of this demonstration, all necessary commands needed to create a single simulation of the dynamics of the system discussed here are presented in the file "CaseStudy2.R" (available on request), partially shown in Supplementary Figure 18(a). The dimensionality of the space (here we consider a 2-dimensional space) is also defined in "CaseStudy2.R" file.

Simulations can be started running the commands in the terminal shown in Supplementary Figure 18(b), also see the Tutorial in Supplementary Note 2, section 2.2. Some examples of how results can be visualized are also presented in the file "CaseStudy2.R", see Supplementary Figure 19(a)-(b). Other ways to generate simulations using "The model simulator" toolbox and to analyze results using R are presented in the Tutorial in Supplementary Note 2, section 2.2.

(a)
(b)
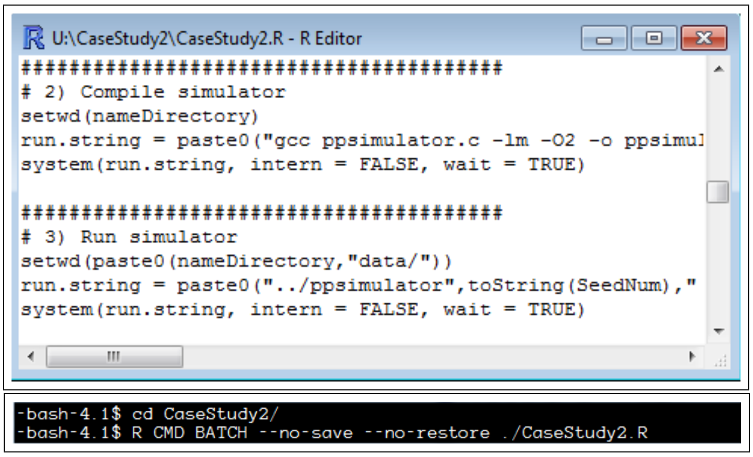

Supplementary Figure 18: (a) Screenshot of the file "CaseStudy2.R" containing necessary commands to use "The model simulator" toolbox. (b) Commands in terminal to run the R file shown in (a).

In the main text the snapshot in Figure 4 is produced using the following parameters: $\nu = 0.01$, $\kappa = 0.05$, $\mu = 1$, $\tau = 1$, $f_0 = 1$, $m = 1$; rIntegral = 3.5, rRadius = 2; dIntegral = 3, dRadius = 2; cIntegral = 1.25, cRadius = 20/3.

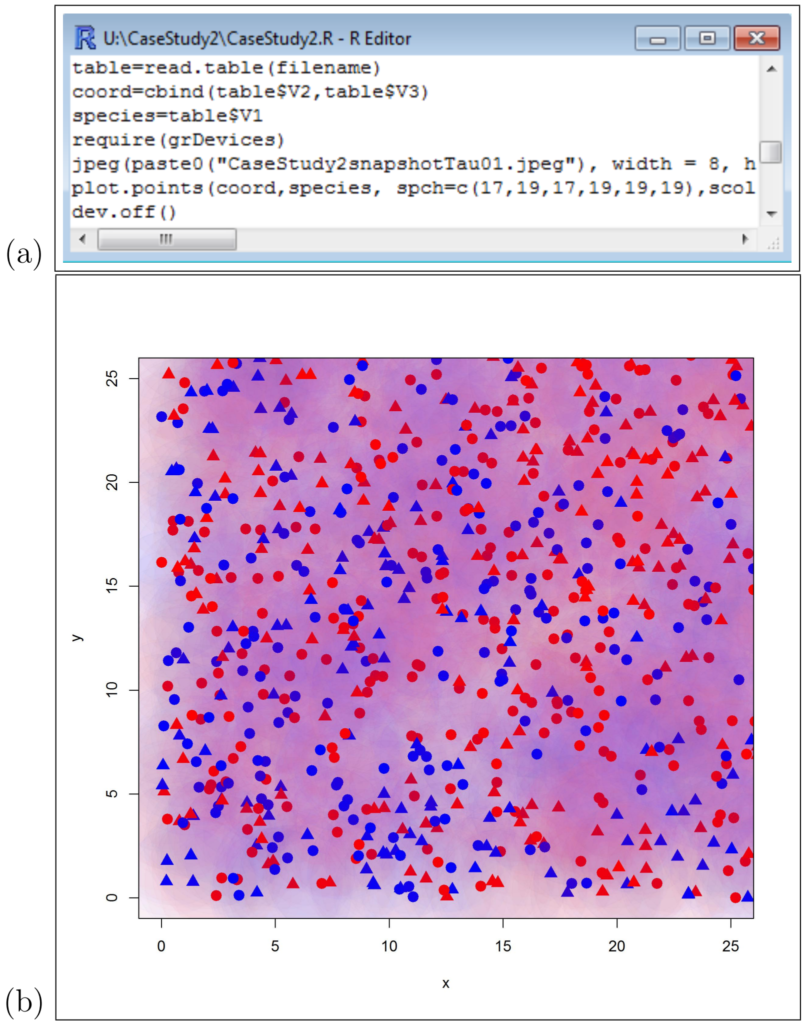

Supplementary Figure 19: Examples of visualization of data obtained from simulations: (a) generating a snapshot of dynamics using R; (b) the resulting snapshot of the system defined in Supplementary Figure 17. Habitat patches are shown as semi-transparent disks or red and blue color, thus the background colours reflect the distribution of habitat patches.

4.3 Generating model equations from “The model constructor” toolbox

Here we demonstrate the application of “The model constructor” toolbox to this case study. The model (263) is defined by the variable named ‘processes’ and shown in Supplementary Figure 20.

One can obtain model equations saved in .tex file by compiling commands shown in Supplementary Figure 21(a). As a result, the model equations are written in corresponding .tex files, see Supplementary Figures 21(b) and 21(c).

```
processes = {Immigration[5, k0, 1], Immigration[6, k0, 1],
DensityIndependentDeath[1, m, 1], DensityIndependentDeath[2, m, 1],
DensityIndependentDeath[3, m, 1], DensityIndependentDeath[4, m, 1],
DensityIndependentDeath[5, mu0, 1], DensityIndependentDeath[6, mu0, 1],
BirthToAnotherTypeByFacilitation[1, 1, 5, r, $r, d, $d, f0(1 - nu)^2(tau + 1)],
BirthToAnotherTypeByFacilitation[1, 2, 5, r, $r, d, $d, f0(1 - nu)nu(tau + 1)],
BirthToAnotherTypeByFacilitation[1, 3, 5, r, $r, d, $d, f0(1 - nu)nu(1 - tau)],
BirthToAnotherTypeByFacilitation[1, 4, 5, r, $r, d, $d, f0 nu^2(1 - tau)],
BirthToAnotherTypeByFacilitation[2, 1, 5, r, $r, d, $d, f0(1 - nu)nu(tau + 1)],
BirthToAnotherTypeByFacilitation[2, 2, 5, r, $r, d, $d, f0 (1 - nu)^2(tau + 1)],
BirthToAnotherTypeByFacilitation[2, 3, 5, r, $r, d, $d, f0 nu^2(1 - tau)],
BirthToAnotherTypeByFacilitation[2, 4, 5, r, $r, d, $d, f0(1 - nu)nu(1 - tau)],
BirthToAnotherTypeByFacilitation[3, 1, 5, r, $r, d, $d, f0(1 - nu)nu(tau + 1)],
BirthToAnotherTypeByFacilitation[3, 2, 5, r, $r, d, $d, f0 nu^2(tau + 1)],
BirthToAnotherTypeByFacilitation[3, 3, 5, r, $r, d, $d, f0(1 - nu)^2(1 - tau)],
BirthToAnotherTypeByFacilitation[3, 4, 5, r, $r, d, $d, f0(1 - nu)nu(1 - tau)],
BirthToAnotherTypeByFacilitation[4, 1, 5, r, $r, d, $d, f0 nu^2(tau + 1)],
BirthToAnotherTypeByFacilitation[4, 2, 5, r, $r, d, $d, f0(1 - nu)nu(tau + 1)],
BirthToAnotherTypeByFacilitation[4, 3, 5, r, $r, d, $d, f0(1 - nu)nu(1 - tau)],
BirthToAnotherTypeByFacilitation[4, 4, 5, r, $r, d, $d, f0(1 - nu)^2(1 - tau)],
BirthToAnotherTypeByFacilitation[1, 1, 6, r, $r, d, $d, f0(1 - nu)^2(1 - tau)],
BirthToAnotherTypeByFacilitation[1, 2, 6, r, $r, d, $d, f0(1 - nu)nu(1 - tau)],
BirthToAnotherTypeByFacilitation[1, 3, 6, r, $r, d, $d, f0(1 - nu)nu(tau + 1)],
BirthToAnotherTypeByFacilitation[1, 4, 6, r, $r, d, $d, f0 nu^2(tau + 1)],
BirthToAnotherTypeByFacilitation[2, 1, 6, r, $r, d, $d, f0(1 - nu)nu(1 - tau)],
BirthToAnotherTypeByFacilitation[2, 2, 6, r, $r, d, $d, f0(1 - nu)^2(1 - tau)],
BirthToAnotherTypeByFacilitation[2, 3, 6, r, $r, d, $d, f0 nu^2(tau + 1)],
BirthToAnotherTypeByFacilitation[2, 4, 6, r, $r, d, $d, f0(1 - nu)nu(tau + 1)],
BirthToAnotherTypeByFacilitation[3, 1, 6, r, $r, d, $d, f0(1 - nu)nu(1 - tau)],
BirthToAnotherTypeByFacilitation[3, 2, 6, r, $r, d, $d, f0 nu^2(1 - tau)],
BirthToAnotherTypeByFacilitation[3, 3, 6, r, $r, d, $d, f0(1 - nu)^2(tau + 1)],
BirthToAnotherTypeByFacilitation[3, 4, 6, r, $r, d, $d, f0(1 - nu)nu(tau + 1)],
BirthToAnotherTypeByFacilitation[4, 1, 6, r, $r, d, $d, f0 nu^2(1 - tau)],
BirthToAnotherTypeByFacilitation[4, 2, 6, r, $r, d, $d, f0(1 - nu)nu(1 - tau)],
BirthToAnotherTypeByFacilitation[4, 3, 6, r, $r, d, $d, f0(1 - nu)nu(tau + 1)],
BirthToAnotherTypeByFacilitation[4, 4, 6, r, $r, d, $d, f0(1 - nu)^2(tau + 1)],
DeathByExternalFactor[1, 1, c, $c, 1], DeathByExternalFactor[1, 2, c, $c, 1],
DeathByExternalFactor[1, 3, c, $c, 1], DeathByExternalFactor[1, 4, c, $c, 1],
DeathByExternalFactor[2, 1, c, $c, 1], DeathByExternalFactor[2, 2, c, $c, 1],
DeathByExternalFactor[2, 3, c, $c, 1], DeathByExternalFactor[2, 4, c, $c, 1],
DeathByExternalFactor[3, 1, c, $c, 1], DeathByExternalFactor[3, 2, c, $c, 1],
DeathByExternalFactor[3, 3, c, $c, 1], DeathByExternalFactor[3, 4, c, $c, 1],
DeathByExternalFactor[4, 1, c, $c, 1], DeathByExternalFactor[4, 2, c, $c, 1],
DeathByExternalFactor[4, 3, c, $c, 1], DeathByExternalFactor[4, 4, c, $c, 1]};
```

Supplementary Figure 20: The variable ‘processes’ defines the model (263) in “The model constructor” toolbox. Here, Fourier transforms of kernels r, d, c are denoted as \$r, \$d and \$c correspondingly. Also, k0 = κ, mu0 = μ, r = r, d = d, c = c, mu = μ, nu = ν, f0 = f_0.

Equations in Fourier space are obtained using commands shown in Supplementary Figure 22(a), the examples of resulting expressions are shown in Supplementary Figures 22(b) and 22(c).

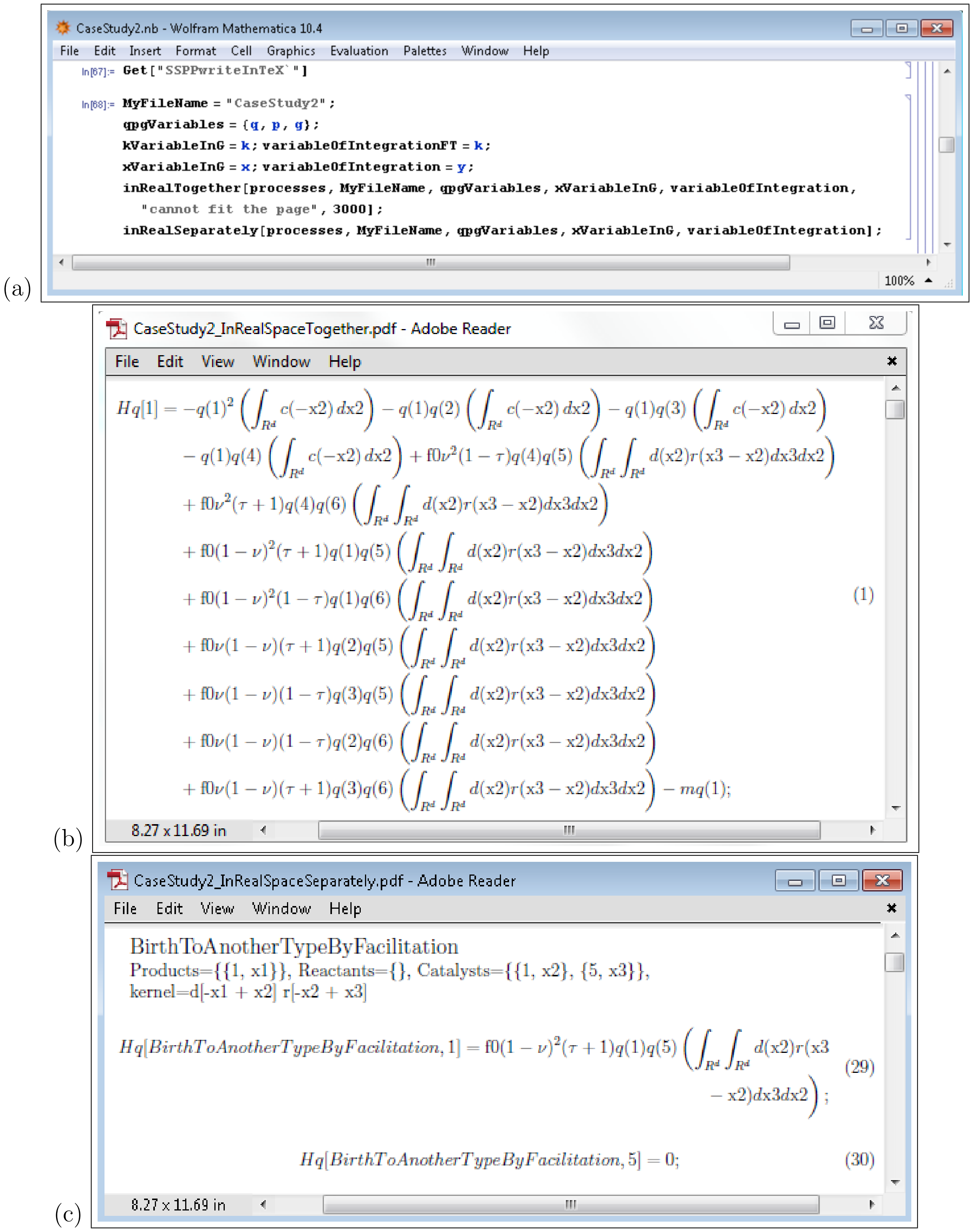

Supplementary Figure 21: (a) Screenshot of some commands from the Mathematica file "CaseStudy2.nb" (available on request). Screenshots of .pdf files obtained from .tex files generated by the commands in (a). The model equations are shown in real space. Contributions from model components are shown: (b) together, (c) separately.

(a) (b) (c)

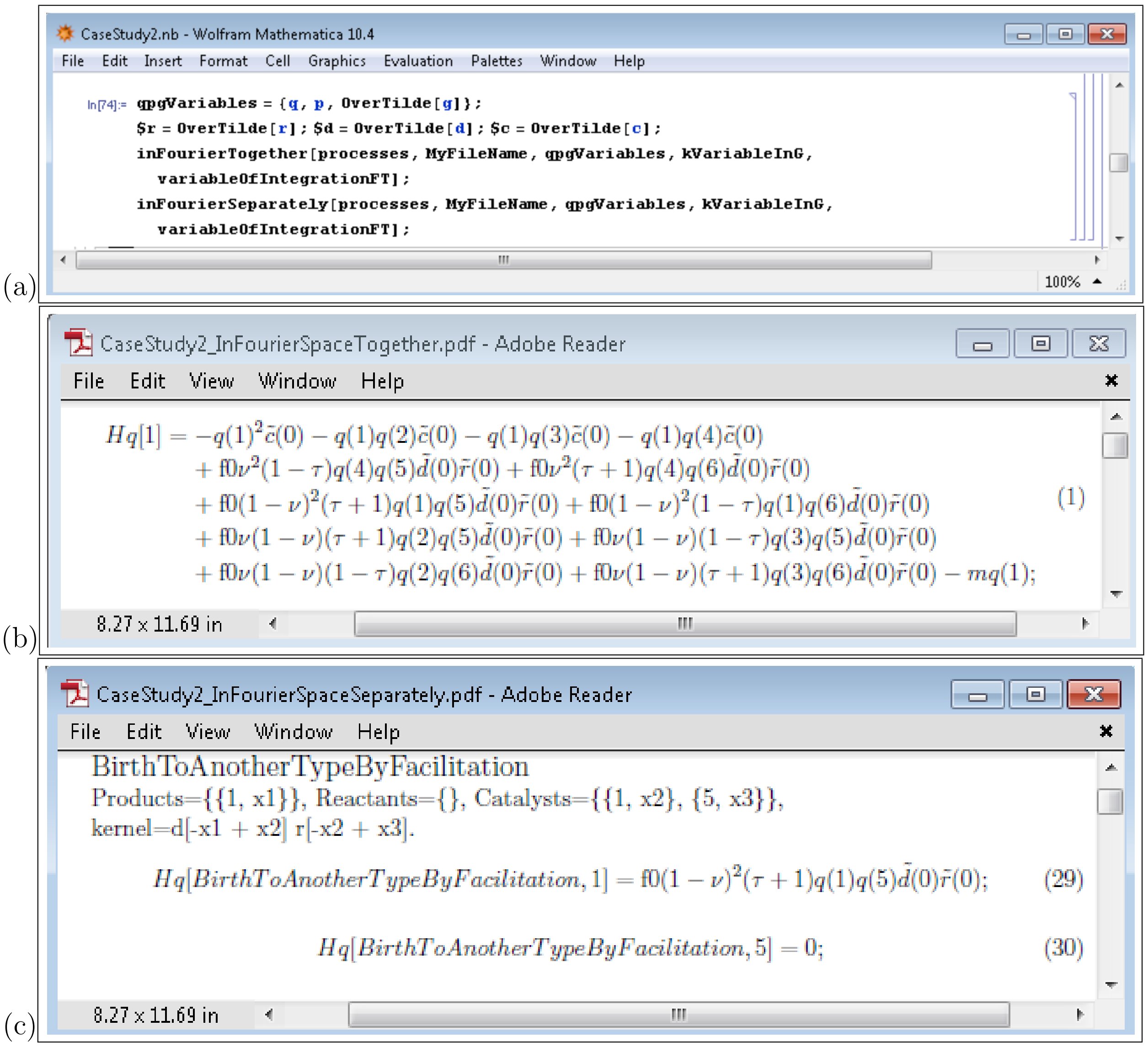

Supplementary Figure 22: (a) Screenshot of some commands from the Mathematica file "CaseStudy2.nb". The model equations are shown in Fourier space. Contributions from model components are shown: (b) together, (c) separately.

Analytical expressions for model equations can be obtained for further transformations in Mathematica, for example see Supplementary Figure 23.

(a) 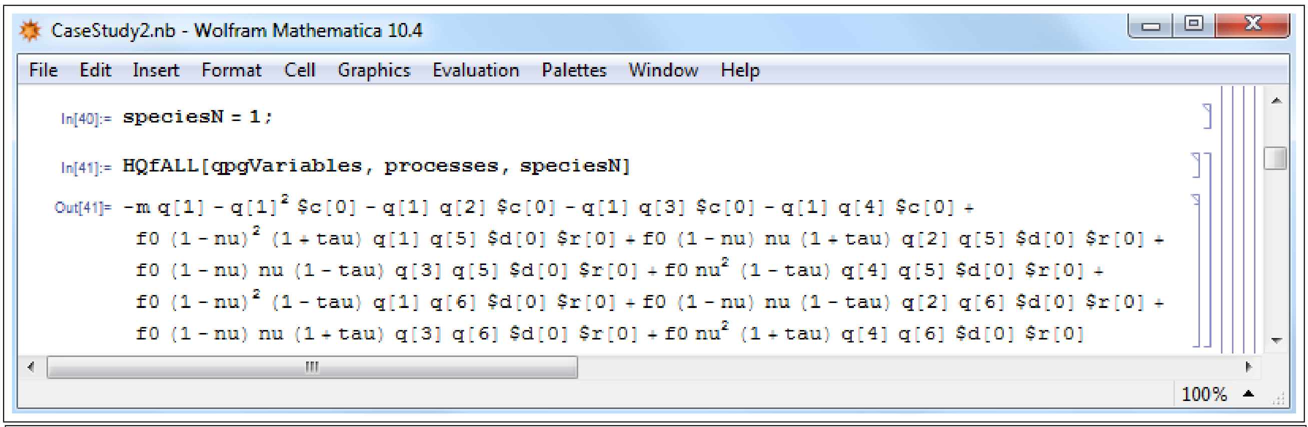

(b) 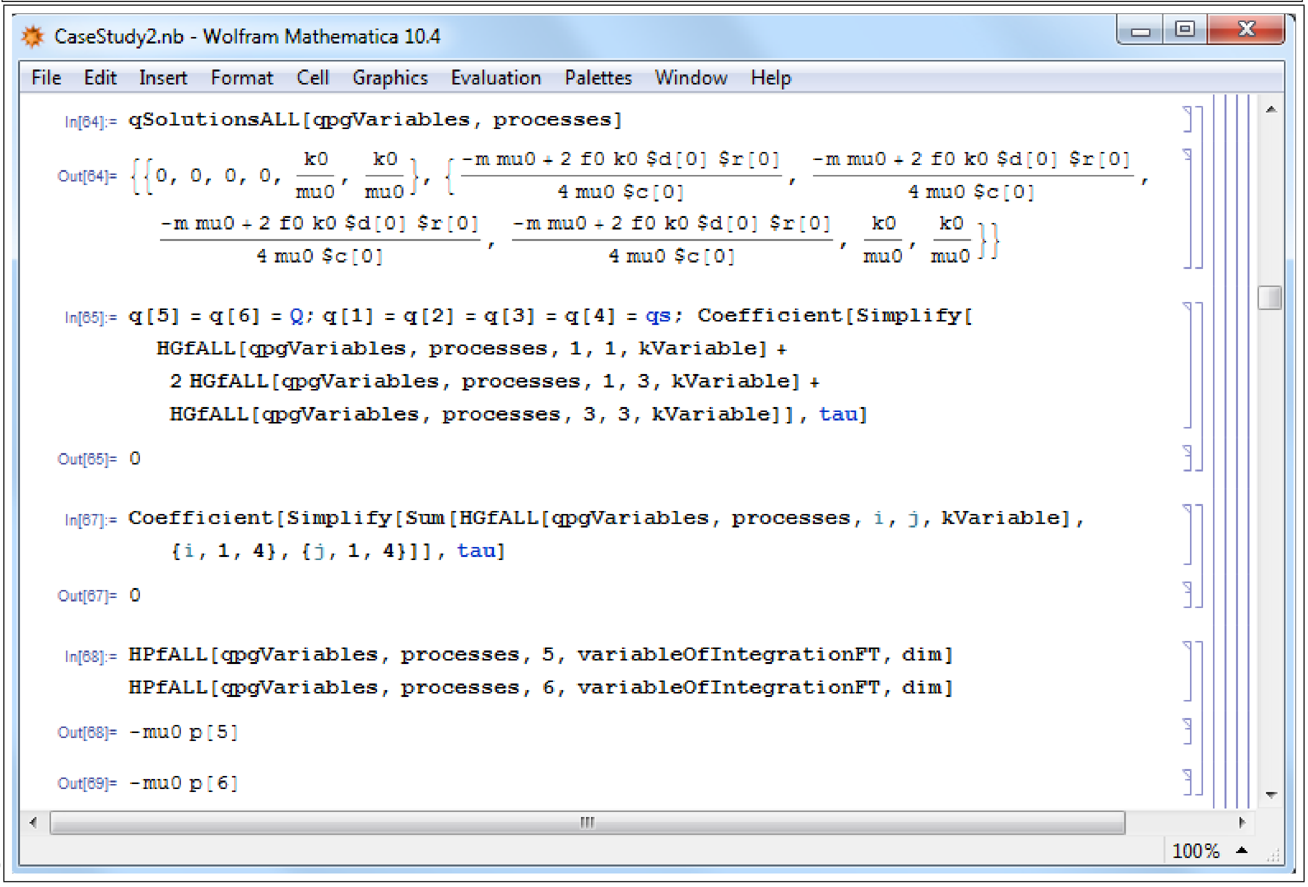

Supplementary Figure 23: Examples of results obtained analytically: (a)The analytical expression for the function H_{q_1} is obtained. Here, functions in Fourier space are denoted with the sign \$ in front of them, e.g. \$c$[k]$ denotes $\tilde{c}(k)$. (b) The analysis of the dependence of genetic differentiation for neutral locus on the strength of selection τ. First, expressions for densities in two possible equilibria are found. Next, it is found that the parameter τ is absent in the sum $H\tilde{g}_{11}(k) + 2H\tilde{g}_{13}(k) + H\tilde{g}_{33}(k)$, as well as in the sum $\sum_{i,j\in\mathcal{G}} H\tilde{g}_{ij}(k)$.

Using numerical values (264) for parameters of the model, the toolbox "The model constructor" can be used to calculate numerical values of densities in all possible equilibria in the system, and to identify the linear stability of each equilibrium, as shown in the Supplementary Figure 24.

Second spatial moments (found from cumulants and densities at equilibrium) and corrections to the mean field densities can be calculated numerically in any equilibrium found by the toolbox.

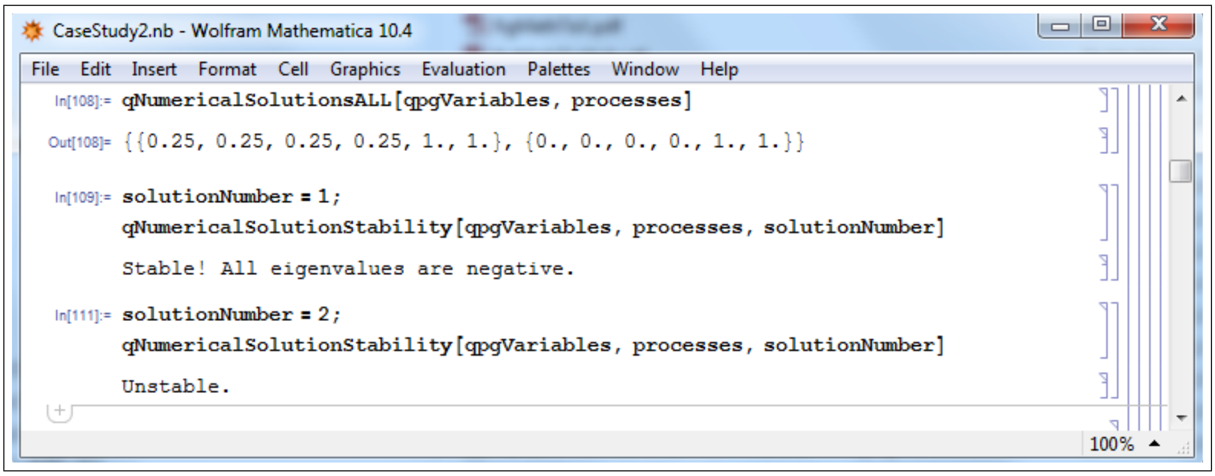

Supplementary Figure 24: Two equilibria are found, with densities shown in form of a vector $\{q_1, q_2, q_3, q_4, q_5, q_6\}$. Equilibrium with non-zero densities of species is stable with respect to infinitesimally small perturbations to the values of mean field densities, which results from the fact that all stability eigenvalues are negative. The equilibrium with zero densities of species is unstable in the same sense because at least one stability eigenvalue is positive.

For a stable equilibrium this is demonstrated in the Supplementary Figures 25-26. The resulting numerical values for the mean field densities, for corrections to the mean field densities, and for cumulants are written in files shown in Supplementary Figure 26.

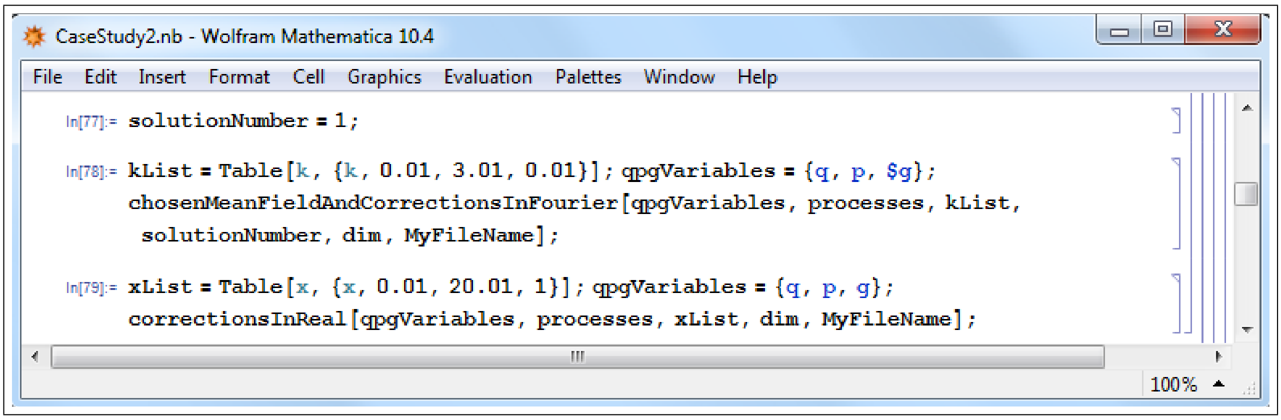

Supplementary Figure 25: Derivation of numerical results for a given equilibrium using "The model constructor" toolbox. For the chosen equilibrium, cumulants are calculated in points defined by "kList" variable in Fourier space and by "xList" variable in real space.

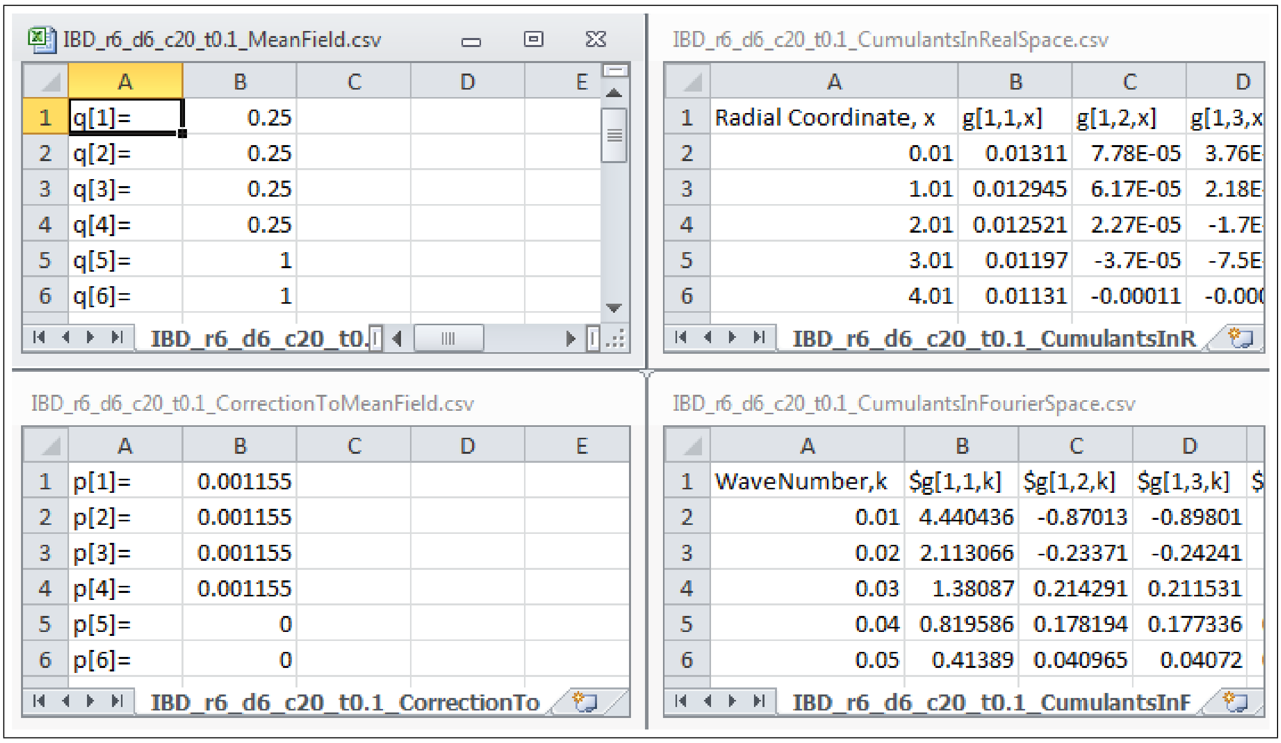

IBD_r6_d6_c20_t0.1_MeanField.csv

	A	B	C	D	E
1	q[1]=	0.25			
2	q[2]=	0.25			
3	q[3]=	0.25			
4	q[4]=	0.25			
5	q[5]=	1			
6	q[6]=	1			

IBD_r6_d6_c20_t0.1_CumulantsInRealSpace.csv

	A	B	C	D
1	Radial Coordinate, x	g[1,1,x]	g[1,2,x]	g[1,3,x
2	0.01	0.01311	7.78E-05	3.76E
3	1.01	0.012945	6.17E-05	2.18E
4	2.01	0.012521	2.27E-05	-1.7E
5	3.01	0.01197	-3.7E-05	-7.5E
6	4.01	0.01131	-0.00011	-0.00[illegible]

IBD_r6_d6_c20_t0.1_CorrectionToMeanField.csv

	A	B	C	D	E
1	p[1]=	0.001155			
2	p[2]=	0.001155			
3	p[3]=	0.001155			
4	p[4]=	0.001155			
5	p[5]=	0			
6	p[6]=	0			

IBD_r6_d6_c20_t0.1_CumulantsInFourierSpace.csv

	A	B	C	D
1	WaveNumber,k	$g[1,1,k]	$g[1,2,k]	$g[1,3,k]
2	0.01	4.440436	-0.87013	-0.89801
3	0.02	2.113066	-0.23371	-0.24241
4	0.03	1.38087	0.214291	0.211531
5	0.04	0.819586	0.178194	0.177336
6	0.05	0.41389	0.040965	0.04072

Supplementary Figure 26: Derivation of numerical results for a given equilibrium using "The model constructor" toolbox. The compilations of commands shown in Supplementary Figure 25 result in .csv files shown here. Here, $\$g$ denotes $\tilde{g}$.

4.4 The research question and results

Here we demonstrate how results obtained by the toolbox presented in this paper can be used to study genetic differentiation in populations. First, we consider the probability that two randomly picked individuals separated by distance x have the same allele in the locus of interest. Such probabilities, denoted as $F_{\rm n}(x)$ at the neutral locus and $F_{\rm s}(x)$ at the selective locus, are given by a sum of probabilities of getting specific alleles at the chosen locus. For the case when there are 2 alleles in neutral locus, we have:

$$F_{\rm n}(x) = F_1(x) + F_2(x), \quad (270)$$
$$F_{\rm s}(x) = F_{\rm A}(x) + F_{\rm B}(x), \quad (271)$$

where

$$F_i(x) = \frac{\text{probability density to find the same allele } i \text{ at distance } x}{\text{probability density to find any pair of alleles at distance } x}, \quad (272)$$

where $i = 1, 2, \mathrm{A}, \mathrm{B}$. The function $F_i(x)$ can be expressed in terms of two-point correlation functions $k^{(2)}_{i,j}(x_i, x_j)$ which measure the probability density to find the individual of type i at location x_i and the individual of type j at location x_j. Due to spatial homogeneity of the system, the correlation functions are symmetric functions which depend on the distance between individuals, i.e. $k^{(2)}_{i,j}(x_i, x_j) = k^{(2)}_{i,j}(x_i - x_j) = k^{(2)}_{i,j}(x_j - x_i)$. Thus, the probability density to find any pair of alleles at distance x is considered as $\sum_{i,i' \in \mathcal{G}} k^{(2)}_{i,i'}(x)$, where the sum is taken over all possible pairs (i, i'). Here, $\mathcal{G} = \{\mathrm{A1}, \mathrm{A2}, \mathrm{B1}, \mathrm{B2}\}$, whereas in the toolboxes this corresponds to $\mathcal{G} = \{1, 2, 3, 4\}$. The

probability density to find a particular allele in two individuals separated by distance x equals to a sum of two-point correlation functions over all pairs (i,i') where both i and i' contain the considered allele. For example, "the probability density to find allele 1 in two individuals separated by distance x" equals to $k^{(2)}_{\mathrm{A1,A1}}(x)+k^{(2)}_{\mathrm{A1,B1}}(x)+k^{(2)}_{\mathrm{B1,A1}}(x)+k^{(2)}_{\mathrm{B1,B1}}(x)$. Thus, the probability to find allele 1 or 2 from the neutral locus equals to $F_1(x)$ or $F_2(x)$ respectively:

$$F_j(x) = \frac{k^{(2)}_{\mathrm{A}j,\mathrm{A}j}(x)+k^{(2)}_{\mathrm{A}j,\mathrm{B}j}(x)+k^{(2)}_{\mathrm{B}j,\mathrm{A}j}(x)+k^{(2)}_{\mathrm{B}j,\mathrm{B}j}(x)}{\sum_{i,i'\in\mathcal{G}} k^{(2)}_{i,i'}(x)}, \quad j=1,2. \tag{273}$$

The probability to find allele A or B at the non-neutral locus is the following:

$$F_\beta(x) = \frac{k^{(2)}_{\beta 1,\beta 1}(x)+k^{(2)}_{\beta 1,\beta 2}(x)+k^{(2)}_{\beta 2,\beta 1}(x)+k^{(2)}_{\beta 2,\beta 2}(x)}{\sum_{i,i'\in\mathcal{G}} k^{(2)}_{i,i'}(x)}, \quad \beta=\mathrm{A},\mathrm{B}. \tag{274}$$

According to the approximation of long-ranged interactions, discussed in this paper and introduced in Refs. [11,12], the following expansion holds:

$$k^{(2)}_{i,j}(x) = q_i q_j + \epsilon^d\left(g_{i,j}(x)+q_i p_j + p_i q_j\right) + o\left(\epsilon^{2d}\right). \tag{275}$$

Substituting (275) into the definition of $F(x)$, and keeping only leading and sub-leading contributions, we obtain:

$$F(x) = F^{(0)} + \epsilon^d F^{(1)}(x), \tag{276}$$

where $F^{(0)}$ and $F^{(1)}$ do not depend on ϵ^d. In the mean field limit $\epsilon^d \to 0$ we find $F^{(0)} = 0.5$ for both loci, which corresponds to a probability to find allele from one or another locus.

To test the convergence of the expansion in the small parameter ϵ^d, we consider three copies of the same system with parameters (264) and (266) but with interaction kernels scaled by a common pre-factor. Three considered systems differ by the following parameters, {rRadius,dRadius,cRadius}, which take the following values: $\{3,3,10\}$, $\{6,6,20\}$, $\{9,9,30\}$. Results are shown in Supplementary Figure 27. Denoting these three systems by the value of their parameters {rRadius,dRadius,cRadius}, one may consider results for systems $\{6,6,20\}$ and $\{9,9,30\}$ as such that can be obtained from the system $\{3,3,10\}$ using scaling with $\epsilon^d = (1/2)^2 = 1/4$, and $\epsilon^d = (1/3)^2 = 1/9$ respectively. One can notice the better agreement between theoretical and numerical simulations in case of interaction kernels with larger range.

Using "The model constructor" toolbox, analytical expressions for mean-field densities q^* were obtained,

$$q_1^* = q_2^* = q_3^* = q_4^* \equiv q^*; \quad q^* = \frac{2f_0\kappa\widetilde{d}(0)\widetilde{r}(0) - m\mu}{4\mu\widetilde{c}(0)}; \quad q_5^* = q_6^* = \frac{\kappa}{\mu}. \tag{277}$$

As one can see, mean-field densities do not depend on the strength of local adaptation. Using "The model constructor" toolbox, we also derived the analytical expressions for genetic differentiation for the neutral and selective locus:

$$F_{\mathrm{n}}(x) = \frac{1}{2} + \frac{\pi}{4q^*}\int_0^\infty \frac{(1-2\nu)\widetilde{d}(k)}{\widetilde{d}(0)-(1-2\nu)\widetilde{d}(k)} J_0(2\pi xk)k\,dk, \tag{278}$$

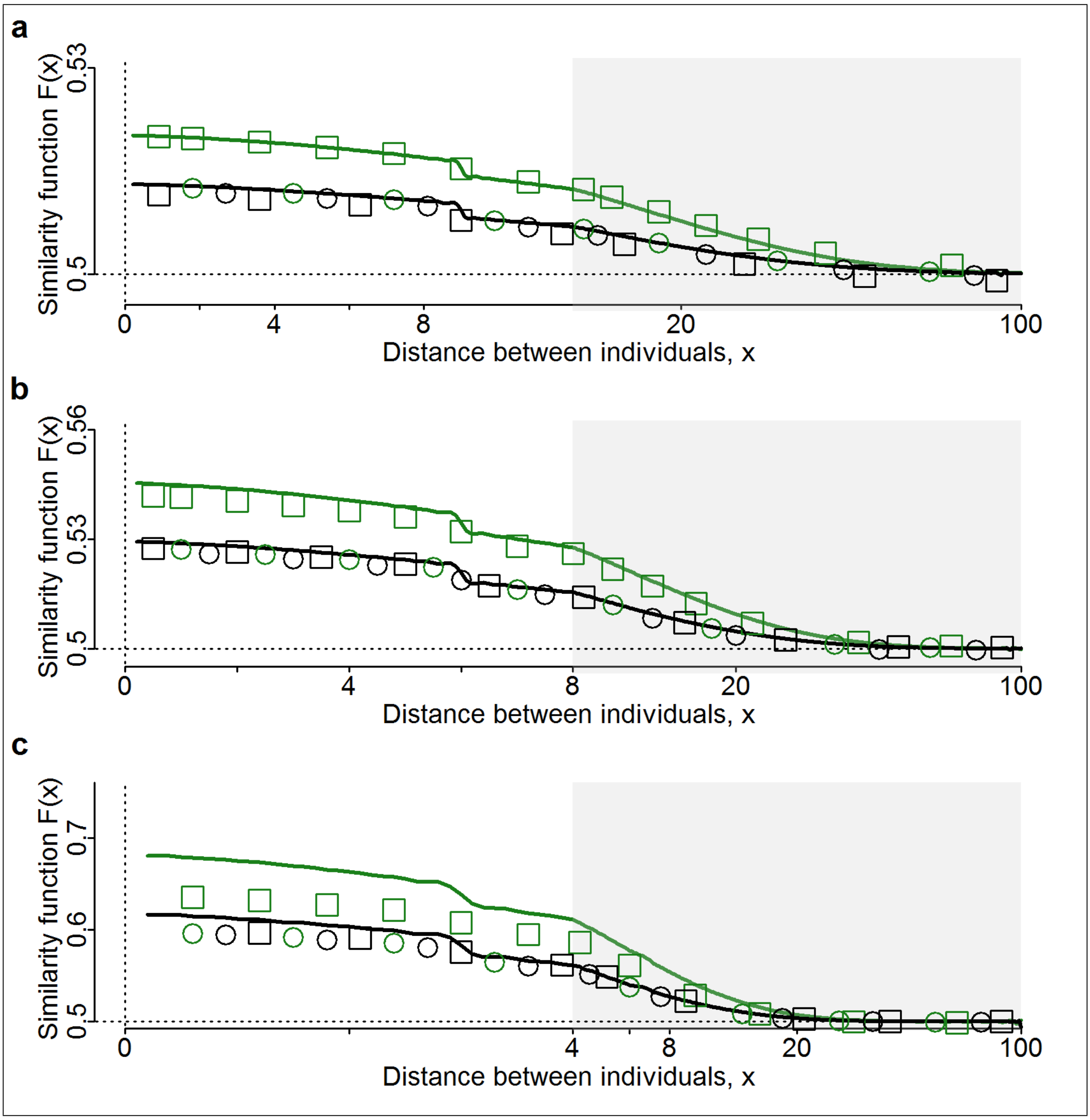

Supplementary Figure 27: The comparison of results obtained from numerical simulations by "The model simulator" toolbox, and results from analytical equations obtained by "The model constructor" toolbox for the system defined by parameters (264) and (266) but with the parameters {rRadius,dRadius,cRadius} equal to the following values: **a** {9, 9, 30}, **b** {6, 6, 20}, **c** {3, 3, 10}. Colors and lines are explained in Figure 4 in the main text.

$$F_s(x) = F_n(x) + \\ + \tau^2 \int_0^\infty \left(\frac{(1-2\nu)\widetilde{d}(k)}{\widetilde{d}(0) - (1-2\nu)\widetilde{d}(k)} \right) \frac{\pi(1-2\nu) f_0 \mu \widetilde{d}(k) \widetilde{r}^2(k)/\widetilde{r}(0)}{2 f_0 \kappa [\widetilde{d}(0) - (1-2\nu)\widetilde{d}(k)]\widetilde{r}(0) + \mu^2} J_0(2\pi x k) k dk, \quad (279)$$

where $J_0(k)$ is Bessel function of the first kind of order zero.

The integration in expressions for similarity functions can be performed analytically in the

limit of extremely rare mutations, i.e. $\nu \to 0$. In that case the leading contribution to the integrals comes from the region of small k. If σ^2 is the variance of the kernel $d(x)$, defined as

$$\sigma^2 = \frac{\int_{R^2} x^2 d(\sqrt{x^2+y^2})dx\,dy}{\int_{R^2} d(\sqrt{x^2+y^2})dx\,dy} = \frac{1}{2}\frac{\int_0^\infty r^3 d(r)dr}{\int_0^\infty r d(r)dr},$$

then the Taylor expansion for $\tilde{d}(k)$ takes the form $\widetilde{d}(k) \approx \widetilde{d}(0)\,(1-2\pi^2\sigma^2 k^2) + o(k^3)$. This can be seen because $\tilde{d}(k) = \int_0^\infty d(r)J_0(2\pi rk)2\pi r\,dr$,

$$\begin{aligned}
\tilde{d}'(k) &= \frac{d}{dk}\int_0^\infty d(r)J_0(2\pi rk)2\pi r\,dr \\
&= 2\pi\int_0^\infty d(r)J_0'(2\pi rk)2\pi r^2\,dr \\
\Rightarrow \tilde{d}'(0) &= 2\pi\int_0^\infty d(r)J_0'(0)2\pi r^2\,dr \\
&= 0, \\
\tilde{d}''(k) &= \frac{d^2}{dk^2}\int_0^\infty d(r)J_0(2\pi rk)2\pi r\,dr \\
&= 4\pi^2\int_0^\infty r^2 d(r)J_0''(2\pi rk)2\pi r\,dr \\
\Rightarrow \tilde{d}''(0) &= 4\pi^2\int_0^\infty r^2 d(r)J_0''(0)2\pi r\,dr \\
&= -4\pi^2\int_0^\infty r^2 d(r)\pi r\,dr \\
&= -4\pi^2\sigma^2\widetilde{d}(0).
\end{aligned}$$

Substituting the Taylor expansion for $\tilde{d}(k)$ into Eqn. (278) gives

$$\begin{aligned}
F_{\mathrm{n}}(x) &\approx \frac{1}{2} + \frac{1}{8\pi q^*\sigma^2}\int_0^\infty \frac{J_0(2\pi xk)kdk}{R_\nu^2+k^2} \\
&= \frac{1}{2} + \frac{1}{8\pi q^*\sigma^2}K_0(2\pi x R_\nu),
\end{aligned} \tag{280}$$

where $x \gg \sigma$ and

$$R_\nu^2 = \frac{\nu}{\pi^2\sigma^2}. \tag{281}$$

Thus, the expression for the similarity function $F_{\mathrm{n}}(x)$ in the neutral locus becomes:

$$F_{\mathrm{n}}(x) = \frac{1}{2} + \frac{1}{2N_{\mathrm{e}}\pi\sigma^2}K_0(x/x_{\mathrm{n}}), \quad x_{\mathrm{n}} = \frac{\sigma}{2\sqrt{\nu}}, \tag{282}$$

where $N_{\mathrm{e}} = 4q^*$ is the total density of all species, $K_0(x)$ is the modified Bessel function of the second kind. Under the same conditions ($\nu \to 0$) for the similarity function $F_{\mathrm{s}}(x)$ in selective locus

we obtain:

$$F_{\rm s}(x) = F_{\rm n}(x) + \frac{\tau^2}{8\nu q_{\rm h}\pi\sigma^2}\frac{K_0(x/x_{\rm n}) - K_0(x/x_{\rm s})}{\mu/M}, \quad x_{\rm s} = \frac{x_{\rm n}}{\sqrt{1+\mu/M}}, \tag{283}$$

where $q_{\rm h} = \kappa/\mu$ is the density of habitat patches of a single type, $M = 4\nu q_{\rm h} f_0 \widetilde{r}(0)\widetilde{d}(0)$ is the rate with which an existing individual produces a new mutant.

One can define a length scale l of a similarity function $F(x)$ as

$$l = \frac{\int_0^\infty x\left(F(x) - \frac{1}{2}\right)dx}{\int_0^\infty \left(F(x) - \frac{1}{2}\right)dx}, \tag{284}$$

and in the similar way the length scale $l_{\rm s}$ of the similarity function $F_{\rm s}(x)$ in selective locus. Using the following notation, $I = \int_0^\infty xK_0(x)dx/\left(\int_0^\infty K_0(x)dx\right)$, and considering equation (282), we obtain:

$$l_{\rm n} = x_{\rm n}I. \tag{285}$$

For simplicity of the following calculation, we re-write equation (283) as

$$F_{\rm s}(x) = \frac{1}{2} + c_{\rm n}K_0(x/x_{\rm n}) + c_{\rm s}\left[K_0(x/x_{\rm n}) - K_0(x/x_{\rm s})\right], \tag{286}$$

where

$$\begin{aligned} c_{\rm n} &= \frac{1}{2N_{\rm e}\pi\sigma^2}, \\ c_{\rm s} &= \frac{\tau^2}{8\nu q_{\rm h}\pi\sigma^2}\frac{M}{\mu}. \end{aligned} \tag{287}$$

According to the definition of a length scale (284), we obtain:

$$l_{\rm s} = l_{\rm n}\frac{1 + \frac{c_{\rm s}}{c_{\rm n}}\left(1 - \frac{x_{\rm s}^2}{x_{\rm n}^2}\right)}{1 + \frac{c_{\rm s}}{c_{\rm n}}\left(1 - \frac{x_{\rm s}}{x_{\rm n}}\right)}, \tag{288}$$

where

$$\frac{c_{\rm s}}{c_{\rm n}} = \left(\frac{\tau^2 N_{\rm e}}{4\nu q_{\rm h}}\right)\frac{M}{\mu}; \tag{289}$$

$$\frac{x_{\rm s}}{x_{\rm n}} = \left(1 + \frac{\mu}{M}\right)^{-1/2}. \tag{290}$$

Thus, the ratio $l_{\rm s}/l_{\rm n}$ depends only on two parameters: μ/M, and $\tau^2 N_{\rm e}/(4\nu q_{\rm h})$. The dependence of $l_{\rm s}/l_{\rm n}$ on these two parameters is shown in Figure 4D in the main text.

Supplementary Note 5

The case study 3 “Optimal foraging”

Contents

5.1 The definition of the model **164**

5.2 Generating simulations from “The model simulator” toolbox **165**

5.3 Generating model equations from “The model constructor” toolbox **167**

5.4 The research question and results **171**

5.1 The definition of the model

The model involves moving individuals (searchers) and resources (targets), their dynamics is discussed below.

Resources (targets)

It is assumed, that resources follow immigration-extinction dynamics, but also that they form clusters. To model such a dynamics, we introduce two different species: target generators as auxiliary species denoted by 1, and targets denoted as species 2.

Target generators appear randomly due to immigration at rate b per unit area, which is described by the model component “Immigration” and the operator $L_1^{\mathrm{IM}}(b)$. Each target generator creates a cluster of targets around itself. Such a cluster consists of a Poisson distributed number of targets, spatially distributed around the cluster center according to a kernel λr, where λ is a prefactor, and $\int_{R^d} r(x)dx = 1$, also $r(-x) = r(x)$.

It is assumed, that from the searcher's point of view each cluster is created instantaneously. This can be modeled by making each target generator to exist only during infinitesimally short time interval, during which it should produce all targets in the cluster. Thus, target generators experience density independent death at rate h, described by the model component “Density Independent Death” and the operator $L_1^{\mathrm{D}}(h)$, where we will consider limit $h \to \infty$. Simultaneously, the rate of creation of targets should increase when the mortality rate increases. Therefore, the rate of creation of targets is multiplied by h. Hence, targets are generated by target generators according to the model component “Birth To Another Type” and the operator $L_{21}^{\mathrm{B}}(h\lambda r)$.

Existing targets disappear at rate μ, which is described by the model component “Density Independent Death” and the operator $L_2^{\mathrm{D}}(\mu)$. Thus, dynamics of targets is determined by the

following sum of operators:

$$L_1^{\mathrm{IM}}(b) + L_1^D(h) + L_{21}^{\mathrm{BT}}(h\lambda r) + L_2^D(\mu). \tag{291}$$

Foragers (searchers)

It is considered that a single forager can make jumps with symmetric kernel c, $c(-x) = c(x)$, and at rate m_{S} (when in slow mode) or m_{F} (when in fast mode), with $m_{\mathrm{S}} < m_{\mathrm{F}}$. Slowly moving searchers are denoted as species 3, and fast moving searchers are denoted as species 4. The movement of searchers is described by model component "Jump", correspondent to the operator $L_3^{\mathrm{J}}(m_{\mathrm{S}}c)$ for slow searchers and the operator $L_4^{\mathrm{J}}(m_{\mathrm{F}}c)$ for fast searchers.

The searchers encounter targets with kernel f multiplied by rate γ. The following normalization is assumed: $\int_{R^d} f(x)dx = 1$. The kernel f is also assumed to be symmetric, $f(-x) = f(x)$. When encountering a target (denoted by 2), the searcher consumes it and switches to the slow mode, i.e. the fast moving searcher 4 changes into slowly moving searcher 3. The dynamics of searchers due to this process is described by the model component "Change In Type By Consumption" and by the operator $L_{342}^{\mathrm{CTC}}(\gamma f)$.

Once in the slow mode, the searcher 3 switches from the slow mode to the fast mode spontaneously at rate α. This corresponds to the model component "Change In Type" and the operator $L_{43}^{\mathrm{CT}}(\alpha)$.

Also, searchers in slow mode consume targets with the same ability as in the fast mode, i.e. the consumption occurs with the same kernel f multiplied by the same rate γ. Such a consumption of targets corresponds to the model component "Death By External Factor" and the operator $L_{23}^{\mathrm{DE}}(\gamma f)$.

Thus, the presence of searchers in the system is described by the following sum of operators:

$$L_3^{\mathrm{J}}(m_{\mathrm{S}}c) + L_4^{\mathrm{J}}(m_{\mathrm{F}}c) + L_{43}^{\mathrm{CT}}(\alpha) + L_{23}^{\mathrm{DE}}(\gamma f) + L_{342}^{\mathrm{CTC}}(\gamma f). \tag{292}$$

The definition of the model

Contributions (291) and (292) form the following operator L which defines the model,

$$\begin{aligned} L =& L_1^{\mathrm{IM}}(b) + L_1^D(h) + L_{21}^{\mathrm{BT}}(h\lambda r) + L_2^D(\mu) \\ &+ L_3^{\mathrm{J}}(m_{\mathrm{S}}c) + L_4^{\mathrm{J}}(m_{\mathrm{F}}c) + L_{43}^{\mathrm{CT}}(\alpha) + L_{23}^{\mathrm{DE}}(\gamma f) + L_{342}^{\mathrm{CTC}}(\gamma f), \end{aligned} \tag{293}$$

where the limit $h \to \infty$ should be taken into account.

5.2 Generating simulations from "The model simulator" toolbox

In this section we demonstrate how to use "The model simulator" toolbox to perform the individual-based simulations of the dynamics of the system defined by (293). All model components from (293) should be defined numerically, for example using the following values of parameters:

$$b = 0.00003; \quad h = 100; \quad \lambda = 40; \quad \mu = 0.01; \quad m_{\mathrm{S}} = 0.1; \quad m_{\mathrm{F}} = 20; \quad \alpha = 0.5; \quad \gamma = 100; \tag{294}$$

and using kernels r, c and f as normalized tophat kernels,

$$r = \text{tophat}[\text{rIntegral}, \text{rRadius}], \quad \text{rIntegral} = 1; \quad \text{rRadius} = 7, \tag{295}$$
$$c = \text{tophat}[\text{cIntegral}, \text{cRadius}], \quad \text{cIntegral} = 1; \quad \text{cRadius} = 10, \tag{296}$$
$$f = \text{tophat}[\text{fIntegral}, \text{fRadius}], \quad \text{fIntegral} = 1; \quad \text{fRadius} = 5. \tag{297}$$

Next, all model components are written in a file with .txt extension. In this case study such a file is "modelCaseStudy3.txt", which is shown in the Supplementary Figure 28. All prefactors to the model components are included in the definition of 'Integral' parameter of corresponding kernels.

```
Immigration[1, 0.00003]
DensityIndependentDeath[1, 100]
BirthToAnotherType[2, 1, tophat[4000, 7]]
DensityIndependentDeath[2, 0.01]
Jump[3, tophat[0.1, 10]]
Jump[4, tophat[20, 10]]
ChangeInType[4, 3, 0.5]
DeathByExternalFactor[2, 3, tophat[100, 5]]
ChangeInTypeByConsumption[3, 4, 2, tophat[100, 5]]
```

Supplementary Figure 28: The content of the file "modelCaseStudy3.txt" which defines the model.

Once the system is defined by the txt file, the toolbox can be used. For simplicity of this demonstration, all necessary commands needed to create a single simulation of the dynamics of the system discussed here are presented in the file "CaseStudy3.R" (available on request), partially shown in Supplementary Figure 29(a). The dimensionality of the space (here we consider a 2-dimensional space) is also defined in "CaseStudy3.R" file.

Simulations can be started by running the commands in the terminal shown in Supplementary Figure 29(b), also see the Tutorial in Supplementary Note 2, section 2.2. Some examples of how results can be visualized are also presented the file "CaseStudy3.R", see Supplementary Figure 30(a)-(b). Other ways to generate simulations using "The model simulator" toolbox and to analyze results using R are presented in the Tutorial in Supplementary Note 2, section 2.2.

(a)
(b)

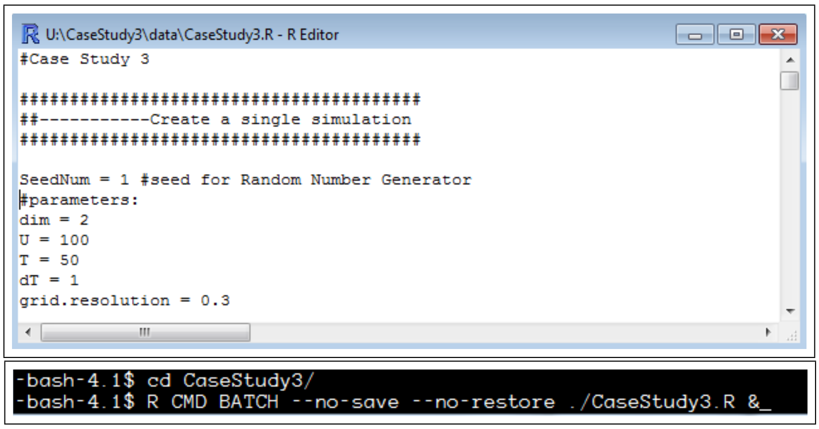

Supplementary Figure 29: (a) Screenshot of the file "CaseStudy3.R" containing necessary commands to use "The model simulator" toolbox. (b) Commands in terminal to run the R file shown in (a).

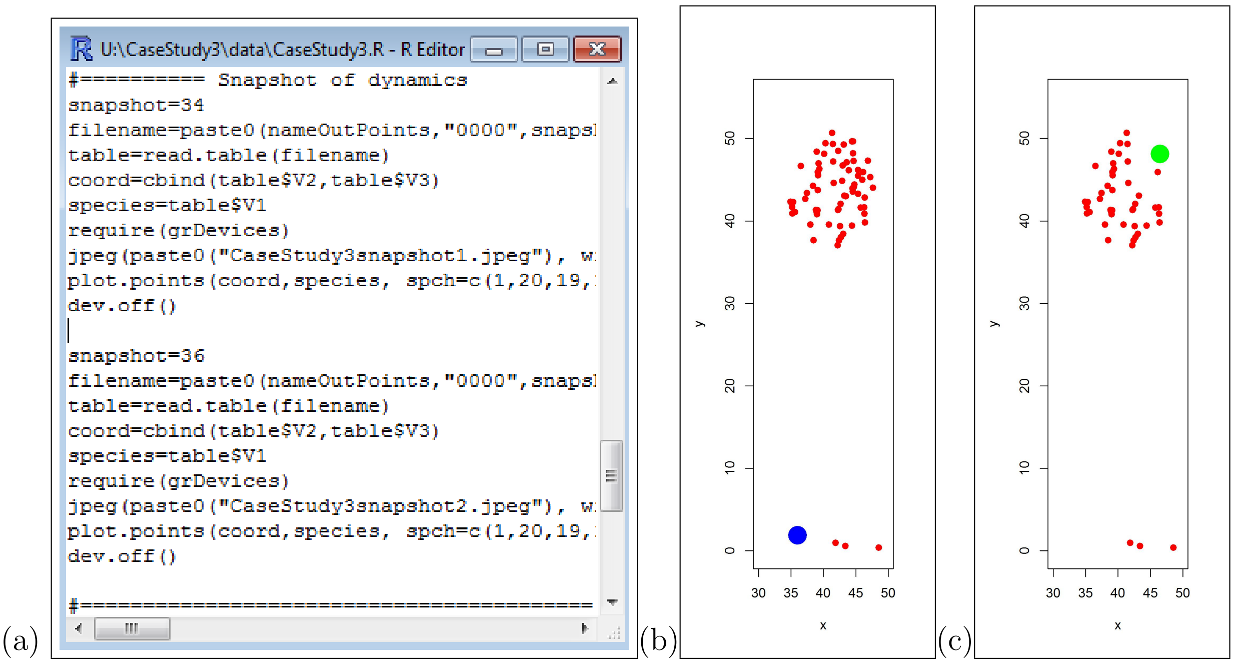

Supplementary Figure 30: Examples of visualization of data obtained from simulations: (a) generating a snapshot of dynamics using R; (b), (c) the resulting snapshots of the system defined in Supplementary Figure 28, a forager in fast mode (blue dot) switches to slow mode (green dot) near cluster or resources (red dots).

5.3 Generating model equations from “The model constructor” toolbox

In this section we present the application of “The model constructor” toolbox to this case study. In “The model constructor” toolbox the model (293) is defined by the variable named “processes” and shown in Supplementary Figure 31.

```
processes ={ Immigration[1, b, 1],
DensityIndependentDeath[1, h, 1],
BirthToAnotherType[2, 1, r, $r, h lambda],
DensityIndependentDeath[2, mu, 1],
Jump[3, c, $c, ms],
Jump[4, c, $c, mf],
ChangeInType[4, 3, alpha, 1],
DeathByExternalFactor[2, 3, f, $f, gamma],
ChangeInTypeByConsumption[3, 4, 2, f, $f, gamma] };
```

Supplementary Figure 31: The variable “processes” defines the model (293) in “The model constructor” toolbox. There is the following link between parameters here and in the definition (293): b = b, h = h, lambda = λ, r = r,c = c, f = f, $r and $c and $f denote Fourier transforms of kernels r and c and f respectively. Other parameters: mu = μ, ms = m_S, mf = m_F, alpha = α, gamma = γ.

One can obtain model equations saved in .tex file by compiling commands shown in Supplementary Figure 32. As a result, the model equations in real and Fourier spaces are written in

corresponding .tex files, resulting in .pdf files shown in the Supplementary Figure 33. Equations can be obtained in real and in Fourier spaces, for all model components together or for each model component separately, e.g. the contribution from a single model component "Infection" to the function H_{q_1} is shown in Supplementary Figure 33(c)-(d).

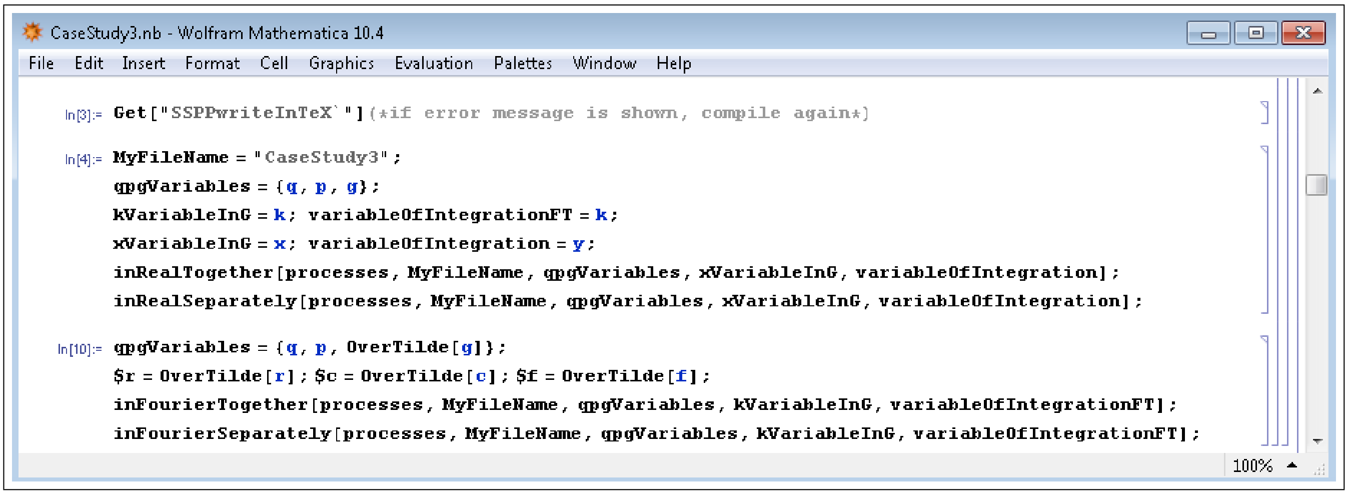

Supplementary Figure 32: Screenshot of some commands from the Mathematica file "CaseStudy3.nb" (available on request). The compilation of these commands creates and saves analytical expressions for model equations into .tex files.

Analytical expressions for model equations can be obtained for further transformations in Mathematica, for example see Supplementary Figure 34 which shows the functions H_{q_i} from equations $dq_i/dt = H_{q_i}$, $i = 1, 2, 3, 4$.

In this case study, as shown in Supplementary Figure 34, $H_{q_3} = -H_{q_4}$ and therefore the system of equations $\{H_{q_i} = 0, i = 1, 2, 3, 4\}$ is degenerate. Using the condition that the total number of searchers is constant, i.e. $d(q_3 + q_4)/dt = 0$, $q_3 + q_4 = A$, A = Const, the equilibrium can be found as shown in Supplementary Figure 35. Using functions H_q, H_p and H_g generated by the toolbox, one can use Mathematica to find numerical values and analytical expressions for cumulants and corrections to the mean field density in a given equilibrium for given numerical values of parameters.

5.4 The research question and results

We extend the model of Gurarie and Ovaskainen (*Gurarie E., Ovaskainen O. Theor. Ecol.* ***6****, 189 – 202 (2013)*) to include behavioral switching between slow and fast movement modes. As shown by the graphical model definition (Figure 5A), we consider a single searcher which makes jumps with kernel $c(x)$ and at rate m_{S} (when in slow mode) or m_{F} (when in fast mode), with $m_{\mathrm{S}} < m_{\mathrm{F}}$. Existing targets disappear at rate μ. New clusters of targets are generated at rate b per unit area, each cluster consisting of a Poisson distributed (mean λ) number of targets, spatially distributed around the cluster center according to kernel $r(x)$. This is achieved by assuming that the targets are produced target generators that appear to random locations and that generate targets around them during their infinitesimally short lifetime. The searchers encounters targets at detection efficiency γ and kernel $f(x)$. When encountering a target, the searcher consumes it and switches to the slow mode, as it allows the searcher to spend more time in areas where there are potentially more targets (Figure 5B). The searcher is assumed to switch from the slow mode back to the fast mode spontaneously at rate α.

Our interest is at the stationary rate ρ^* by which each searcher consumes targets. This can be computed by solving the stationary state of the system under the condition that the density of searchers $q_3 + q_4$ is constant, denoted here by A. This yields $\rho^* = (\beta/k_2^* - \mu)k_2^*/A$, where $\beta = \lambda b$ is the appearance rate of targets, k_2^* is the stationary density of targets, and the expression in brackets is the rate at which a randomly selected target disappears due to consumption. As we are not interested in resource competition among multiple searchers, we consider the case of a single searcher, obtained technically by taking the limit $A \to 0$.

In the mean-field approximation, the stationary density of targets is $k_2^* \approx q_2^* = \beta/(A\gamma + \mu)$, and thus the consumption rate of targets by a single searcher is $\rho^* \approx \rho_0 = \gamma\beta/\mu$ independently of the parameter α.

In the first-order approximation, the stationary density of targets is $k_2^* \approx q_2^* + \epsilon^d p_2^*$. While the general expression for p_2^* is found using "The model constructor" toolbox, we simplify here by considering the limit where in the fast movement mode the movements are very fast ($m_{\mathrm{F}} \to \infty$), and the slow movements are very slow ($m_{\mathrm{S}} = 0$). At this limit, the first order approximation for the consumption rate of targets is $\rho^* \approx \rho_0 + \epsilon^d \rho_1$, where

$$\rho_1 = \frac{\beta\gamma^2(\alpha\lambda\mu I_1 - \beta\gamma I_2)}{\mu(\beta\gamma + \alpha\mu)(\alpha + \mu)}, \tag{298}$$

and $I_1 = \int \tilde{f}(\omega)^2 \tilde{r}(\omega)^2 d\omega$ and $I_2 = \int \tilde{f}(\omega)^2 d\omega$. Note that at the limit of $\alpha \to \infty$ (shown by gray dashed line in Figure 5C), it holds that $\rho_1 \to 0$, which is expected, as at these limit the searcher samples random locations of space, thus corresponding to the assumptions of the mean-field model. At the limit of $\alpha = 0$ (shown by gray continuous line in Figure 5C), the searcher remains stationary, and thus relies on new targets that appear in its neighborhood. The consumption rate is maximized at an intermediate switching rate α^* (Figure 5C, vertical line). This is because a consumer that remains continuously in the slow mode is not efficient in finding new resource aggregates, whereas a consumer that remains continuously in the fast mode misses the opportunity of elevated resource availability within aggregates. At the optimal switching rate α^* the searcher remains stationary until it has depleted so many targets that it becomes beneficial to search for a new cluster.

The simple closed-form expression for the consumption rate of targets (298) makes it possible to compute analytically the optimal switching rate α^*, and thus to examine how it depends on

the properties of the model:

$$\alpha^* = \rho_0 \frac{I_2}{\lambda I_1} + \sqrt{\mu\rho_0 \left(1 + \frac{\rho_0}{\mu}\frac{I_2}{\lambda I_1}\right)\left(1 + \frac{I_2}{\lambda I_1}\right)}. \tag{299}$$

As one example, the optimal switching rate decreases (and thus the time that the searcher spends stationary increases) with the increasing level of target clustering and with decreasing level of target detection efficiency (Figure 5D).

The above analysis has assumed that the energy requirements of the consumer are the same in the slow as in the fast mode, but in reality the fast movement mode is likely to be more energeticaly costly. During the fast foraging mode, the resource acquisition rate is ρ_0, so it takes a time $1/\rho_0$ before the consumer finds a resource and switches to the slow mode. If the fast mode costs κ resource units per unit time, then the rate of resource expenditure due to fast foraging is $\alpha\kappa/\rho_0$. The net resource gain is

$$\rho_0 + \epsilon^d \rho_1 - \alpha\kappa/\rho_0 = \rho_0 + \epsilon^d \frac{\rho_0\gamma(\alpha\lambda I_1 - \rho_0 I_2)}{(\rho_0 + \alpha)(\alpha + \mu)} - \frac{\alpha\kappa}{\rho_0},$$

where we used $\rho_0 = \gamma\beta/\mu$. This function has a maximum at $\alpha > 0$ (and, correspondingly, its derivative with respect to α at $\alpha = 0$ is positive) unless

$$\kappa > \epsilon^d \frac{\rho_0\gamma}{\mu}\left[\lambda \int_{R^d} \left\{\tilde{f}(\omega)\tilde{r}(\omega)\right\}^2 d\omega + \left(1 + \frac{\rho_0}{\mu}\right)\int_{R^d} \tilde{f}^2(\omega) d\omega\right], \tag{300}$$

in which case the fastest net resource acquisition is at $\alpha = 0$. Note that, in the main text, we have set $\epsilon = 1$, i.e. the length scale of the kernels f and r is the true biological one and has not been rescaled by ϵ.

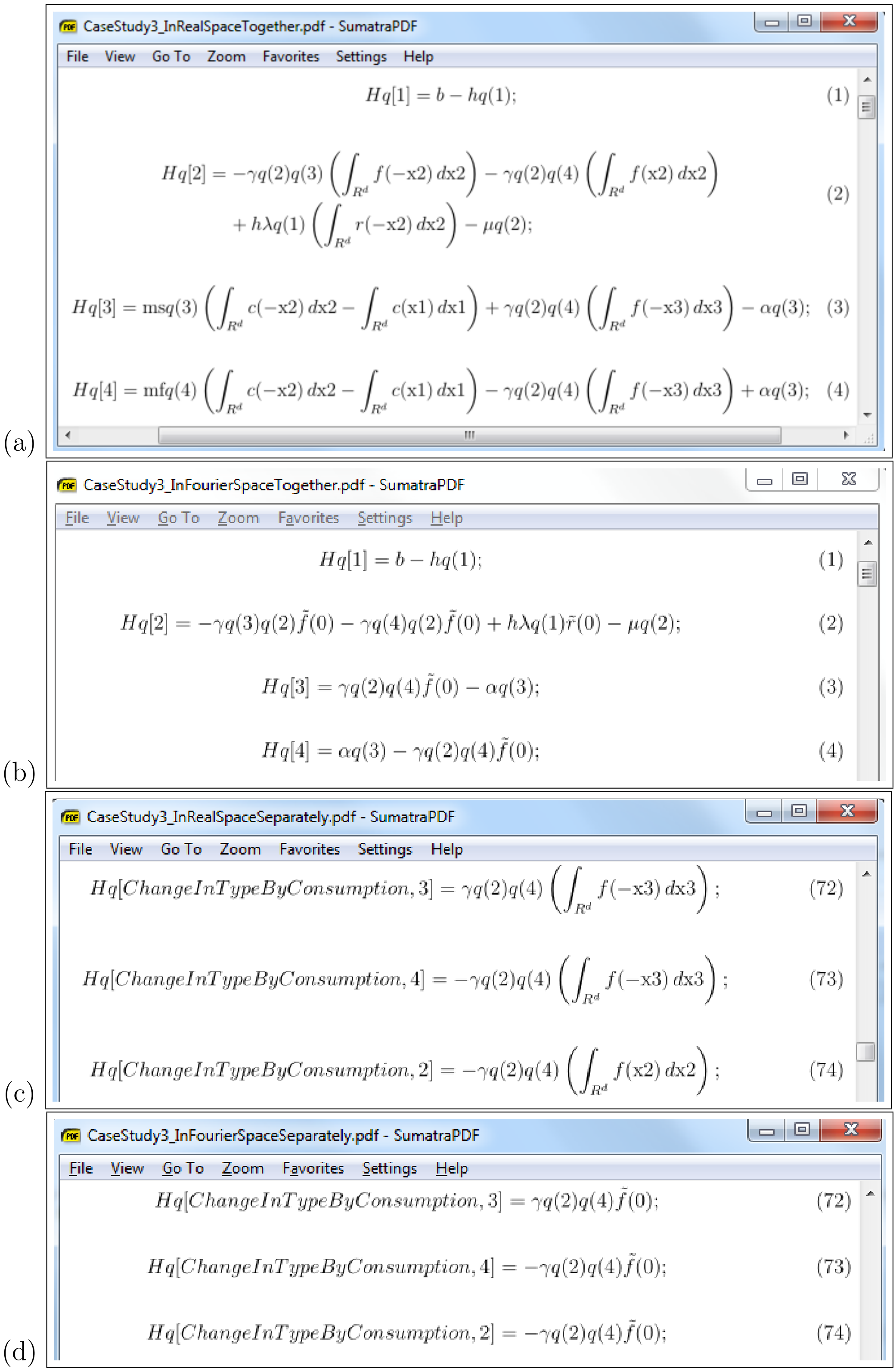

Supplementary Figure 33: Screenshots of .pdf files obtained from .tex files generated by the commands in Supplementary Figure 32. The contributions to the model equations from all model components are considered together in: (a) real space, (b) Fourier space; and separately in: (c) real space, (d) Fourier space. Here, $q(1)$ is q_1, and $Hq[1]$ is H_{q_1} from the equation for the density q_1, $dq_1/dt = H_{q_1}$.

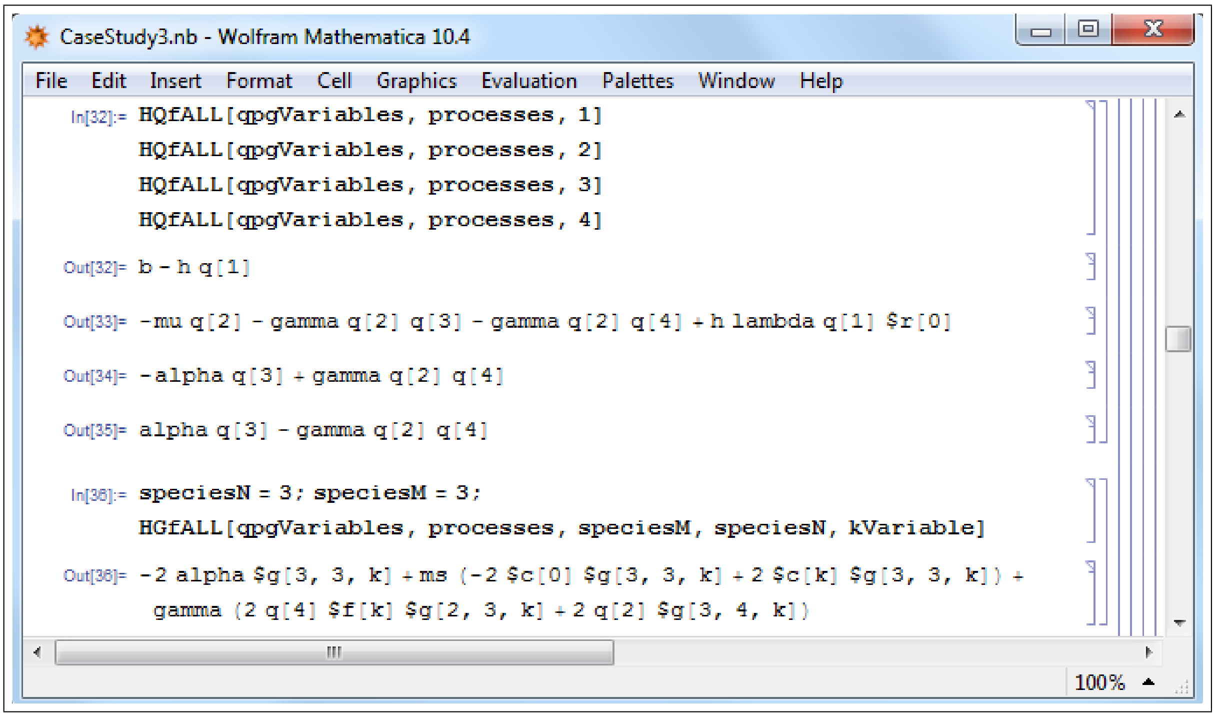

Supplementary Figure 34: Examples of analytical expressions for the model equations: the functions H_{q_i} and the function $H\tilde{g}_{33}(k)$ from the equation $d\tilde{g}_{33}(k,t)/dt = H\tilde{g}_{33}(k)$. In Mathematica codes we denote functions in Fourier space by using the sign \$ in front of them, e.g. \$c$[k]$ and \$g$[1,2,\mathrm{k}]$ are $\tilde{c}$ and $\tilde{g}$, i.e. the Fourier transforms of $c(x)$ and $g_{12}(x)$ respectively. For details see the Tutorial in Supplementary Note 2, section 2.3.

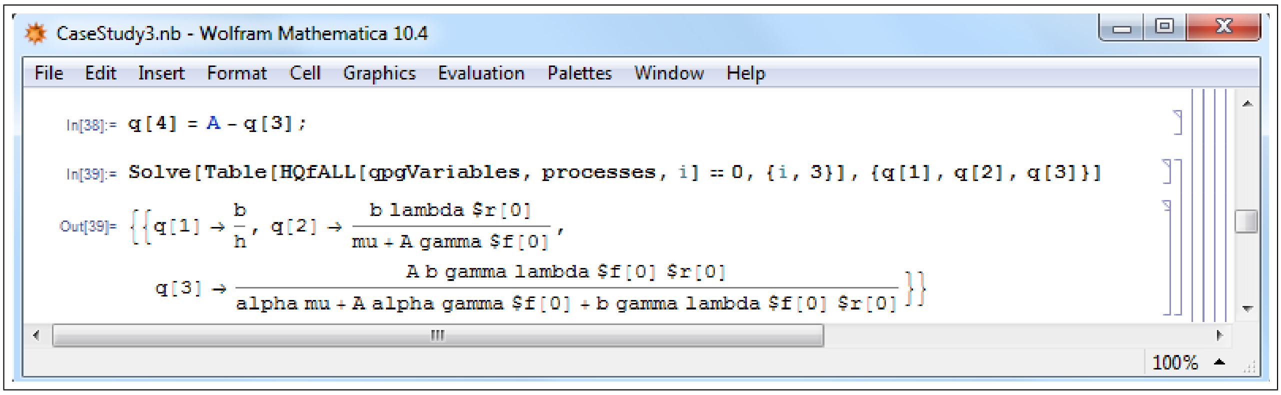

Supplementary Figure 35: Finding mean field densities at the equilibrium using Mathematica.
